# Visible light-induced halogen-atom transfer by N-heterocyclic carbene-ligated boryl radicals for diastereoselective C(sp^3^)–C(sp^2^) bond formation†

**DOI:** 10.1039/d4sc02962c

**Published:** 2024-08-13

**Authors:** Luca Capaldo, Ting Wan, Robin Mulder, Jonas Djossou, Timothy Noël

**Affiliations:** a Flow Chemistry Group, Van 't Hoff Institute for Molecular Sciences (HIMS), University of Amsterdam Science Park 904 1098 XH Amsterdam The Netherlands t.noel@uva.nl; b Department of Chemistry, SynCat Lab, Life Sciences and Environmental Sustainability, University of Parma 43124 Parma Italy luca.capaldo@unipr.it; c The Research Center of Chiral Drugs, Innovation Research Institute of Traditional Chinese Medicine, Shanghai University of Traditional Chinese Medicine Shanghai 201203 China

## Abstract

Photoinduced halogen-atom transfer (XAT) has rapidly emerged as a programmable approach to generate carbon-centered radical intermediates, mainly relying on silyl and α-aminoalkyl radicals as halogen abstractors. More recently, ligated boryl radicals have also been proposed as effective halogen abstractors under visible-light irradiation. In this study, we describe the use of this approach to enable C(sp^3^)–C(sp^2^) bond formation *via* radical addition of carbon-centered radicals generated *via* XAT onto chloroalkynes. Our mechanistic investigation reveals a complex interplay of highly reactive radical intermediates which, under optimized conditions, delivered the targeted vinyl chlorides in excellent yields and *Z* : *E* ratios. Finally, we demonstrated the synthetic value of these products in transition metal-based cross-coupling reactions.

## Abstract 2

In this work, we report the synthesis of vinyl chlorides from chloroalkynes *via* ligated boryl radicals-mediated XAT under visible light. This study introduces the first protocol using XAT to establish a C(sp^3^)–C(sp^2^) bond through radical addition.

## Graphical abstract

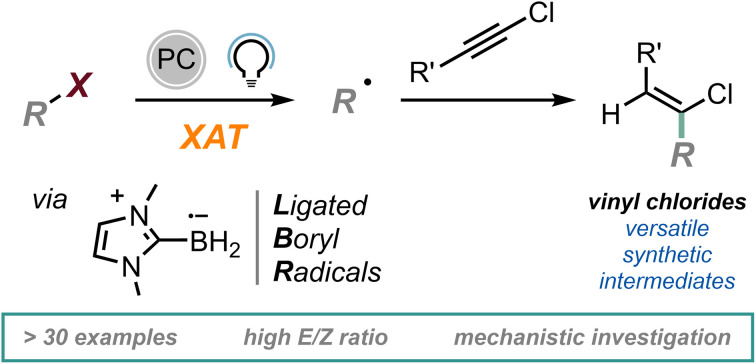

## Introduction

Over the past decade, the field of chemistry has experienced a notable expansion in synthetic methodologies, marked by the development of increasingly selective techniques focused on the use of carbon-centered radicals.^1^ This advancement is attributed to the accumulation of knowledge over the past century regarding these fleeting intermediates,^2^ coupled with the emergence of gentler approaches for their generation, such as electro-^3^ and photo-catalysis.^4^ As a specific example, light-mediated halogen-atom transfer (XAT) has emerged as a versatile and controllable method for generating radical intermediates from readily available haloalkanes, predominantly relying on silyl and α-aminoalkyl radicals as halogen abstractors.^5^ In these processes, the excited state of a photocatalyst oxidizes silicon-containing compounds (*e.g.*, supersilane and supersilanol)^6–8^ and amines^9,10^ to unveil the halogen abstractor.

**Fig. 1:**
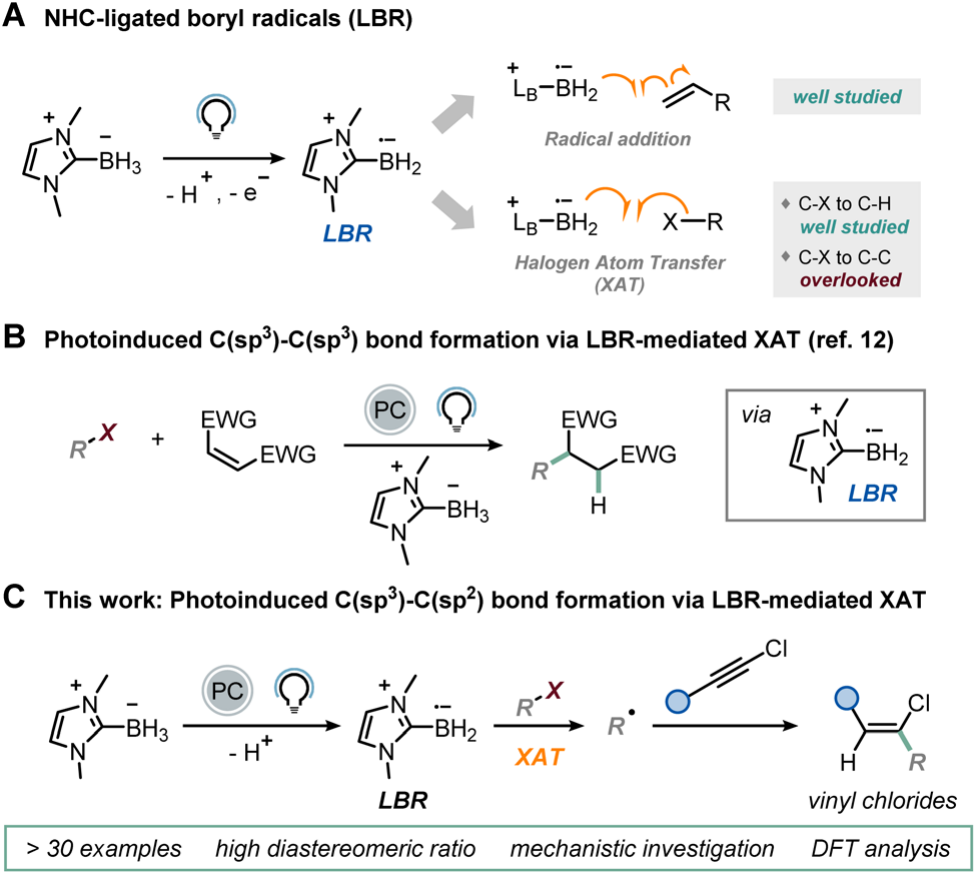
(A) Use of NHC-ligated boryl radicals (LBRs) in light-mediated synthesis. (B) C(sp^3^)–C(sp^3^) bond formation *via* LBR-mediated XAT. (C) This work: C(sp^3^)–C(sp^2^) bond formation *via* LBR-mediated XAT.

More recently, N-heterocyclic carbene (NHC)-ligated boryl radicals (LBRs) have also been proposed as halogen abstractors under visible-light irradiation.^11–13^ While the mild nucleophilicity of these boron-centered radicals^14^ has traditionally been exploited for B–C bond formation,^15–19^ their affinity for halogens can also be utilized for the homolytic activation of a C–X bond, generating carbon-centered radicals *via* XAT (Fig. 1A).^20–24^ We have recently exploited this capability, triggering the reactivity under visible-light irradiation in the presence of an organic photocatalyst to realize C(sp^3^)–C(sp^3^) bond formation through a classical Giese-type reaction mechanism (Fig. 1B).^12,25^ In this process, the excited state of the photocatalyst is reductively quenched by an NHC-ligated borane,^26^ leading to the spontaneous deprotonation and generation of the corresponding LBR.

This species is then responsible for the XAT with a variety of alkyl iodides, producing carbon-centered radicals that subsequently participate in a radical addition step with electron-poor olefins. We wondered whether an LBR-based approach could be viable for C(sp^3^)–C(sp^2^) bond formation. Drawing upon the findings from our initial report, we surmised that an effective approach to tackle this challenge would entail exploring a radical addition pathway onto carbon–carbon triple bonds. Unlike acetylenic bromides and sulfones,^27^ which typically yield alkynylation products *via* a radical addition/β-fragmentation pathway, their chlorinated analogues retain the halogen atom in the final product, as demonstrated by Hashmi in a seminal work based on HAT.^28^ Consequently, the resulting vinyl chlorides, which are valuable intermediates for transition metal-based cross-coupling reactions,^29–31^ contain the coveted C(sp^3^)–C(sp^2^) bond. This reactivity difference arises from the higher carbon–halogen bond dissociation energy (BDE) for a C–Cl bond, which inhibits the β-fragmentation step.^28,32^ Since this chlorine radical elimination is hindered, we hypothesized that the vinyl radical, deriving from the radical addition of the alkyl radical generated *via* XAT onto the triple bond, could abstract a hydrogen atom from another molecule of NHC-ligated borane, thus initiating a LBR-sustained radical chain mechanism. Given the mild nucleophilicity of LBRs, competitive direct addition onto chloroalkynes should be unfavorable. Herein, we present the realization of this synthetic protocol (Fig. 1C).

## Results and discussion

We started our investigations by optimizing the reaction between iodocyclohexane 1a (2 equiv.) and chloroalkyne 2a (0.1 mmol) in the presence of ligated borane B1 (1.2 equiv.). When 4CzIPN (5 mol%) served as the photocatalyst (PC), product 3 was obtained in 26% ^1^H NMR yield (Table 1, entry 1) after 12 hours of blue LED irradiation. Employing a more oxidizing acridinium catalyst (Mes-AcrClO_4_, *E*(PC*/PC_red_) = +2.06 V *vs.* SCE)^33^ resulted in similar yields (30%, entry 2), while photocatalysts such as Ru(bpy)_3_(PF_6_)_2_ (*E*(PC*/PC_red_) = +0.77 V *vs.* SCE)^34^ and Eosin Y (*E*(PC*/PC_red_) = +0.83 V *vs.* SCE)^35^ afforded lower NMR yields (entries 3 and 4). This trend can be explained based on the oxidation potential of B1 (*E*_p/2_ = +0.89 V *vs.* SCE):^36^ reductive quenching becomes inefficient with the latter two photocatalysts. The addition of water and a slight increase in the stoichiometry of B1 (1.5 equiv.) proved beneficial for the transformation (entries 5 and 6), allowing to obtain product 3 in 68% yield. We found that the addition of K_3_PO_4_ boosted reactivity (6 h *vs.* 12 h), presumably by facilitating deprotonation subsequent to the oxidation of B1 by PC* to reveal the desired LBR (entry 7). Notably, the product could be obtained with a very good ^1^H NMR yield (∼80%) even with a reduced loading of 4CzIPN (0.5 mol%, entry 8). Under these conditions, an excellent *Z* : *E* ratio of 92 : 8 was measured (see ESI for additional info†). Control experiments (entries 9 and 10) showed that light, 4CzIPN and B1 are all necessary for initiating the desired reactivity. Moreover, when we tried to execute the same transformation with silyl radicals or α-aminoalkyl radicals, we could not obtain product 3 in satisfactory yields (Section 9 in the ESI†).

**Table 1:** Optimization of reaction conditions

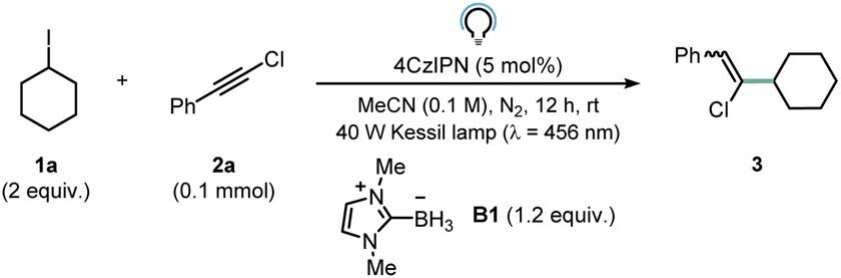		
Entry	Variation from conditions	Yield^a^ (%)
1	None	26
2	Mes-AcrClO_4_ instead of 4CzIPN	30
3	Ru(bpy)_3_(PF_6_)_2_ instead of 4CzIPN	10
4	Eosin Y instead of 4CzIPN	14
5	As entry 1, MeCN/H_2_O 9 : 1 instead of MeCN	56
6	As entry 5, 1a : 2a : B1 2 : 1 : 1.5	68
7	As entry 6, 4CzIPN (2 mol%), K_3_PO_4_ (0.5 equiv.), 6 h	80
**8**	**As entry 7, 4CzIPN (0.5 mol%), 6 h**	**82 (*Z* : *E* 92 : 8)** b **; 73** c
9	As entry 8, no PC, no B1 or no light	n.d.
10	As entry 8, *Δ* (80 °C, dark)	n.d.

a Yields were determined *via*^1^H-NMR by using CH_2_Br_2_ as external standard.

b The *Z* : *E*-ratio was determined *via*^1^H-NMR of the reaction crude.

c Yield of the isolated product.

**Fig. 2:**
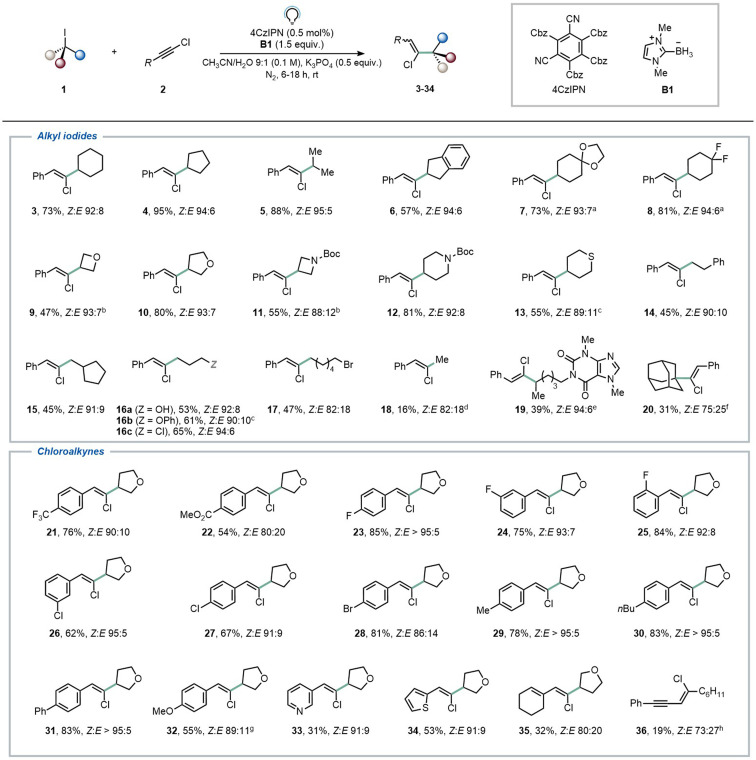
Scope of the transformation. Reported yields of compounds reflect values obtained after purification *via* column chromatography; displayed *Z* : *E* ratios were determined *via*^1^H-NMR of the reaction crude. Major isomers (*Z*) are shown. For secondary organic halides (GP3 in the ESI†): 2 (0.5 mmol), 1 (2 equiv.), B1 (1.5 equiv.), K_3_PO_4_ (0.5 equiv.) in CH_3_CN/H_2_O 9 : 1 (5 mL) in the presence of 4CzIPN (0.5 mol%), 6 h. For primary organic halides (GP4 in the ESI†): 2 (1.5 equiv.), 1 (0.5 mmol), B1 (1.5 equiv.), K_3_PO_4_ (0.5 equiv.) in CH_3_CN/H_2_O 9 : 1 (5 mL) in the presence of 4CzIPN (0.5 mol%), 18 h. Solutions were bubbled with N_2_ (5 min) prior to irradiation (40 W Kessil lamp, *λ* = 456 nm). ^*a*^1 was used in 1.5 equiv. ^*b*^Reaction time: 8 h. ^*c*^Reaction time: 16 h. *^d^*10 equivalents of methyl iodide were used. *^e^*0.5 mmol of 1 and 2 equiv., of 2 were used. ^*f*^0.5 mmol of 1 and 1.2 equiv., of 2 were used. ^*g*^34% of the corresponding alkyne (32′) was isolated as well. ^*h*^Reaction carried out on 0.1 mmol scale.

With the optimized conditions in hand (Table 1, entry 8), we proceeded to explore the scope of the transformation (Fig. 2). First, various secondary halides were subjected to the reaction conditions, revealing that both cyclic and acyclic iodoalkanes delivered the anticipated products in good to excellent yields and diastereomeric ratios (3–13, 47–95%). Both acetal and CF_2_ moieties were successfully tolerated under the reaction conditions, yielding the desired products (7–8, 73–81%). Saturated N- and S-containing heterocycles were also well accommodated, encompassing pharmaceutically relevant azetidine and piperidine derivatives (9–13, 47–81%). Subsequently, we explored the option of employing primary iodides under our reaction conditions. This necessitated slight modifications to the reaction conditions, as we found in our previous report,^12^ including a different organic halide/chloroalkyne ratio (1 : 2 1 : 1.5) and prolonged light exposure (18 h *vs.* 6 h). With these conditions, complete conversion of the halide was observed, affording compounds 14–18 in satisfactory yields (45–65%). Intriguingly, our approach proved chemoselective towards C–I bonds (see compound 17) leaving C–Br unaffected. This shows complementary reactivity compared to the more common XAT manifolds based on silyl and α-aminoalkyl radicals. Also methylation could be realized using our methodology (18). A caffeine derivative could be functionalized as well, albeit in modest yield (19, 39%). One tertiary iodide also proved to be an effective coupling partner, yielding the desired compound in a synthetically useful yield and modest diastereoselectivity (20, 31%, *Z* : *E* 75 : 25). However, alkyl bromides continued to present a limitation for this XAT methodology, consistent with observations in our previous work.^12^

Regarding the scope of the chloroalkynes counterpart, we observed that the reaction remained efficient across a wide array of functional groups. Notably, strongly electron-withdrawing groups on the aromatic ring facilitated the formation of the desired products in good yields (21–22, 54–76%). Similarly, halogen substituents on the aromatic ring were well accommodated under our conditions (23–28, 62–85%), without any interference observed during the XAT step. Chloroalkynes bearing mildly electron-donating substituents on the aromatic ring afforded products 29–31 in excellent yields (78–83%). Interestingly, the presence of a strong electron-donating methoxy substituent partially redirected reactivity, resulting in the desired product 32 with a 55% isolated yield, along with the formation of 34% of the corresponding alkyne (32′), wherein the chlorine atom underwent formal replacement by the alkyl radical. It is noteworthy that the formation of alkynes was not observed under other conditions, and to the best of our knowledge, this reactivity is much more commonly observed for brominated analogues featuring weak C–Br bonds (see Section 8 in the ESI†).^27^ Finally, heterocycles (such as pyridine and thiophene) and conjugated carbon–carbon double bonds were well tolerated, yielding the corresponding products 33–35 in yields ranging from 31% to 53%. Similarly, when using a chlorodiyne as radical trap, the expected product 36 was formed, albeit in lower yield. On the contrary, fully aliphatic chloroalkynes remain a limitation of the scope, in accordance with the literature.^28^ More in detail, 1-chlorooctyne did not deliver the expected product and resulted in decomposition.

**Fig. 3:**
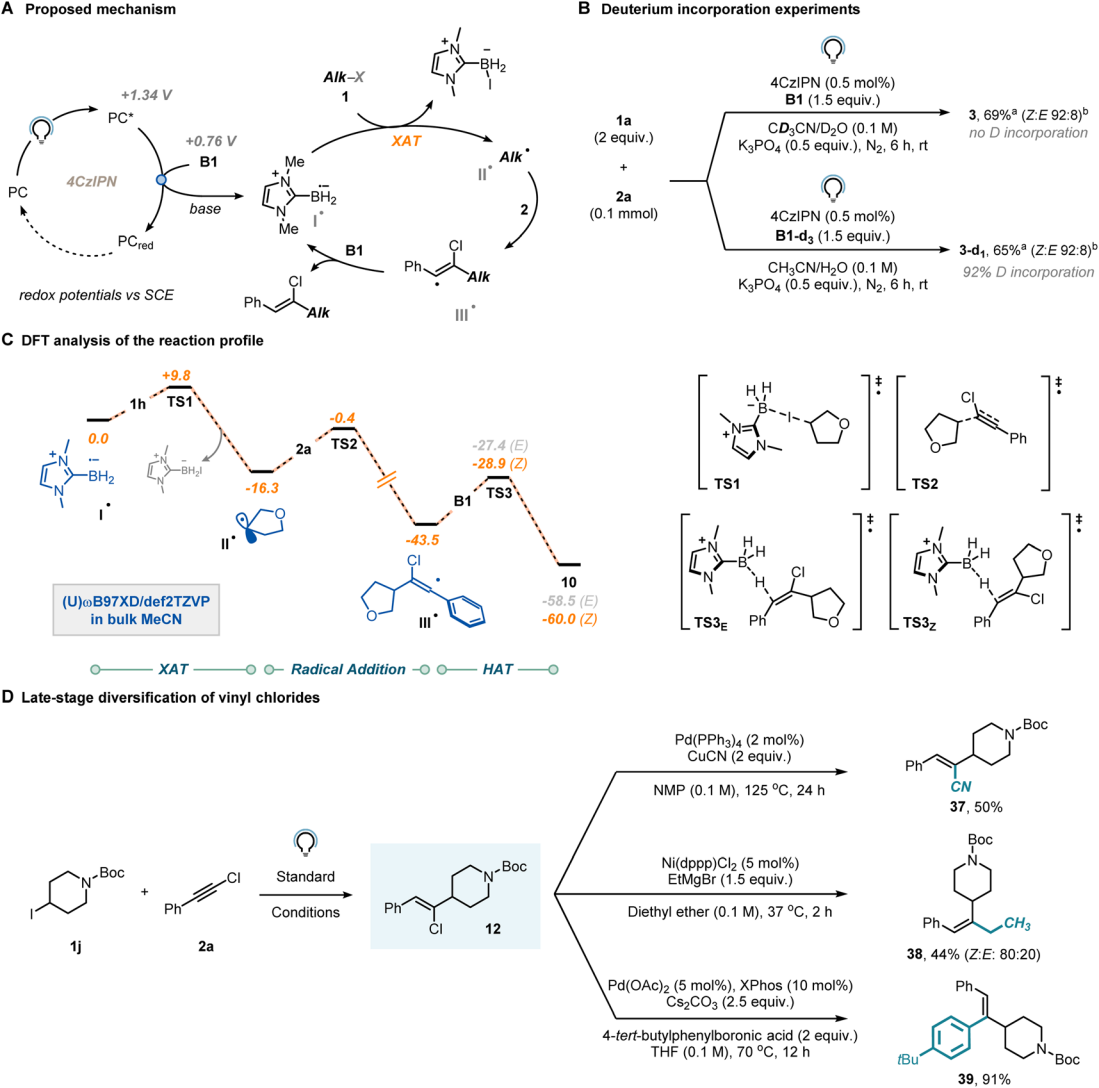
(A) Proposed mechanism. (B) Deuterium labelling experiments. (C) DFT analysis of the reaction pathway. (D) Late-stage diversification campaign for vinyl chlorides. ^*a*^Yields were determined *via*^1^H-NMR by using CH_2_Br_2_ as external standard. ^*b*^The *Z* : *E*-ratio was determined *via*^1^H-NMR of the reaction crude.

We then shifted our attention towards elucidating the mechanism to gain a more comprehensive understanding of the reaction. Our initial hypothesis (Fig. 3A) proposed that reductive quenching of the photocatalyst excited state would lead, upon base-aided deprotonation, to the crucial ligated boryl radical (I˙). This species would subsequently engage with organohalides *via* XAT to generate a carbon-centered radical (II˙), which would then be readily intercepted by the chloroalkyne (2). The regioselectivity of this step is known to be influenced by both thermodynamic and kinetic factors.^28^ The resulting vinyl radical (III˙) is expected to exhibit high reactivity and would abstract a hydrogen atom from a molecule of B1, yielding the anticipated vinyl chloride and regenerating the LBR, thereby initiating a radical chain mechanism.

To further investigate the occurrence of a radical chain mechanism, we conducted deuteration experiments (Fig. 3B). When the model reaction was performed in the presence of B1-d_3_ instead of B1, complete incorporation of deuterium was observed, strongly suggesting the operation of a radical chain mechanism. In contrast, when deuterated solvent was used (CD_3_CN/D_2_O 9 : 1), no deuterium atom was incorporated into the product. Additionally, we measured the Kinetic Isotope Effect (KIE) of the reaction using the parallel reaction method^37^ and found a value of 1.2 (see Section 9 in the ESI†), which suggests that the halogen atom transfer might be limiting the rate of the reaction.

To get further insights, we conducted a computational investigation aimed at modelling the entire reaction profile between substrates 1h and 2a, simulating all key steps including XAT, radical addition, and HAT. We adopted Density Functional Theory (DFT) at the (U)ωB97xD/def2TZVP level of theory to optimize the relevant stationary points, also including the effect of the solvent (CH_3_CN) using an implicit model (Fig. 3C). Initially, we considered the reaction between the LBR (I˙) and 1a*via*TS1 (Δ*G*^‡^ = +9.8 kcal mol^−1^) to afford alkyl radical II˙, which is then trapped by chloroalkyne 2a*via*TS2 (Δ*G*^‡^ = +15.9 kcal mol^−1^). Vinyl radicals are known to adopt either a linear or a bent geometry, depending on the substituent attached to the carbon bearing the unpaired electron. Our DFT analysis confirmed that when this substituent is a π-type group (*e.g.*, a phenyl ring), the vinyl radical adopts a linear geometry, thus suggesting that stereochemical control does not occur in the radical addition step to yield III˙.^38–40^ Moreover, it has been reported^39^ that the barrier for inversion of bent vinyl radicals is typically low, further diminishing the likelihood of the radical addition step being the key determinant of the observed stereoselectivity. From III˙, the targeted vinyl chloride 10 is formed through reaction with NHC-ligated borane B1*via*TS3*_Z_* (Δ*G*^‡^ = +14.6 kcal mol^−1^) or TS3*_E_* (Δ*G*^‡^ = +16.1 kcal mol^−1^). We propose that *Z*-diasteroselectivity originates in this step, despite the marginal thermodynamic (ΔΔ*G*_*E*–*Z*_ = 1.5 kcal mol^−1^) and kinetic (ΔΔ*G*^‡^ = +1.5 kcal mol^−1^) driving forces. It is crucial to note that due to the conjugated structure of the prepared vinyl chlorides, triplet–triplet sensitization might be an underlying process. While we did observe photoisomerization, it is noteworthy that this process was identified to be comparatively slower than the formation of the vinyl chloride itself. Consequently, one can halt the reaction before photoisomerization occurs (see Section 9 in the ESI†).^41,42^ A quantum yield for vinyl chloride formation was determined to be 10% *via* ferrioxalate actinometry. Such a modest value is consistent with a process being supported by either short-lived radical chain propagations or an inefficient initiation process.^43^

Finally, we demonstrated the synthetic value of the synthesized vinyl chlorides (Fig. 3D). The chlorine atom in *Z*-12 could be readily substituted with cyanide under Pd-catalysis to afford product 37 in 50% yield. Similarly, the same substrate exhibited compatibility for a nickel-mediated Kumada–Corriu-type coupling (38, 44%) and was also effective in the Pd-catalyzed Suzuki–Miyaura coupling (39, 91%).

## Conclusions

In summary, this work elucidates the effective utilization of NHC-ligated boryl radicals for promoting the formation of C(sp^3^)–C(sp^2^) bonds under visible light irradiation through XAT. The boron-centered radicals served as halogen abstractors for a diverse array of organoiodides, generating carbon-centered radicals that readily underwent radical addition with chloroalkynes, resulting in the formation of synthetically valuable vinyl chlorides. Despite being previously reported by Hashmi *via* HAT,^28^ the current methodology offers several advantages, including reduced excess of the alkyl coupling partner, broader substrate scope, minimal photocatalyst loading, and improved diastereoselectivity. While the reduced nucleophilicity of the ligated boryl radical limits the activation of alkyl bromides, it concurrently enhances selectivity by discouraging direct addition to the triple bond. This study represents the first protocol employing XAT for establishing a C(sp^3^)–C(sp^2^) bond through a radical addition mechanism and demonstrates the synthetic value of ligated boryl radicals.

## Author contributions

L. C.: conceptualization, investigation, writing – original draft, writing – review & editing, project administration. T. W. & R. M.: investigation, data curation, writing – review & editing. T. N.: supervision, writing – review & editing.

## Conflicts of interest

There are no conflicts to declare.

## Supplementary Material

SC-015-D4SC02962C-s001

## Data Availability

The data supporting this article have been included as part of the ESI.†
