# Supplementary material for: Visible light-induced halogen-atom transfer by N-heterocyclic carbene-ligated boryl radicals for diastereoselective C(sp3)–C(sp2) bond formation
Source: Chem Sci. 2024 Aug 13;15(36):14844–50. doi: 10.1039/d4sc02962c (PMC11340342; doi:10.1039/d4sc02962c)
Supplement: SC-015-D4SC02962C-s001 [file SC-015-D4SC02962C-s001.pdf]

Supplementary Information (SI) for Chemical Science.
This journal is © The Royal Society of Chemistry 2024

Supporting Information

Visible Light-Induced Halogen-Atom Transfer by N-Heterocyclic Carbene-Ligated Boryl Radicals for Diastereoselective $C(sp^3)–C(sp^2)$ Bond Formation

Luca Capaldo,*[,a,b,†] Ting Wan,[a,c,†] Robin Mulder,[a] Jonas Djossou[a] and Timothy Noël*[,a]

[a] *Flow Chemistry Group, van 't Hoff Institute for Molecular Sciences (HIMS), University of Amsterdam, Science Park 904, 1098 XH Amsterdam, The Netherlands.*

[b] *SynCat Lab, Department of Chemistry, Life Sciences and Environmental Sustainability, University of Parma, 43124 Parma, Italy.*

[c] *The Research Center of Chiral Drugs, Innovation Research Institute of Traditional Chinese Medicine, Shanghai University of Traditional Chinese Medicine, Shanghai 201203, China.*

[†] *These authors contributed equally to this work.*

.

1. General information S4

2. Reactor design S5

UFO reactor S5

3. Charts of starting materials S7

Alkyl iodides S7

Chloroalkynes S7

4. Synthesis of starting materials S8

GP1: Synthesis of alkyl iodides S8

GP2: Synthesis of chloroalkynes S9

5. Optimization of reaction conditions S15

6. NOESY experiments to determine double bond geometry S18

7. General Procedures for the Synthesis of Vinyl Chlorides S20

GP3: Procedure for secondary alkyl iodides S20

GP4: Procedure for primary alkyl iodides S20

8. Characterization data S21

9. Mechanistic investigation S35

Quantum yield measurements S35

Deuterium labelling experiments S36

Experiment with deuterated solvent S37

Experiment with deuterated ligated borane S37

Additional control experiments S39

Determination of Kinetic Isotope Effect (KIE) via parallel reaction method. S39

*Photoisomerization of vinyl chloride **3**. S39*

*Reaction profile of the reaction between **1h** and **2m**. S40*

Comparison with other XAT methodologies. S42

10. Computational details S43

11. References S55

12. NMR Spectra S57

1. General information

Reagents and consumables. All reagents and solvents were bought from Sigma Aldrich, TCI, Flurochem, VWR International and Biosolv and used as received. Disposable syringes were purchased from Laboratory Glass Specialist. Product isolation was performed manually, using silica (P60, SILICYCLE), or automatically, by a Biotage® Isolation Four, with Biotage® SNAP KP-Sil 20 or 50 g flash chromatography cartridges. TLC analysis was performed using Silica on aluminum foils TLC plates (F254, SILICYCLE) with visualization under ultraviolet light (254 nm and 365 nm) or appropriate TLC staining (potassium permanganate or cerium ammonium molybdate).

NMR spectroscopy. ^{1}H (400 MHz or 300 MHz), ^{13}C (101 MHz or 75 MHz) and ^{19}F (376 MHz or 282 MHz) spectra were recorded unless stated otherwise on ambient temperature using a Bruker AV400 or a Bruker AV300. ^{1}H NMR spectra are reported in parts per million (ppm) downfield relative to $CDCl_3$ (7.26 ppm) or CD_2Cl_2 (5.32 ppm) and all ^{13}C NMR spectra are reported in ppm relative to $CDCl_3$ (77.2 ppm) or CD_2Cl_2 (53.8 ppm) unless stated otherwise. The multiplicities of signals are designated by the following abbreviations: s (singlet), d (doublet), t (triplet), q (quartet), m (multiplet), dd (doublet of doublets), dt (doublet of triplets), td (triplet of doublets), tt (triplets of triplets), ddd (doublet of doublet of doublets), qd (quartet of doublet). Coupling constants (*J*) are reported in hertz (Hz). NMR data was processed using the MestReNova 14 software package. Known products were characterized by comparing ^{1}H NMR and ^{13}C NMR spectra with those available in the literature.

Mass spectrometry. High resolution mass spectra (HRMS) were collected on an AccuTOF LC, JMS-T100LP Mass spectrometer (JEOL, Japen).

2. Reactor design

UFO reactor

For all experiments a homemade, 3D-printed reactor was adopted.[1] The reactor was designed to fit reaction vials and to be equipped with a Kessil lamp PR160L series (λ_{em} = 456 nm).

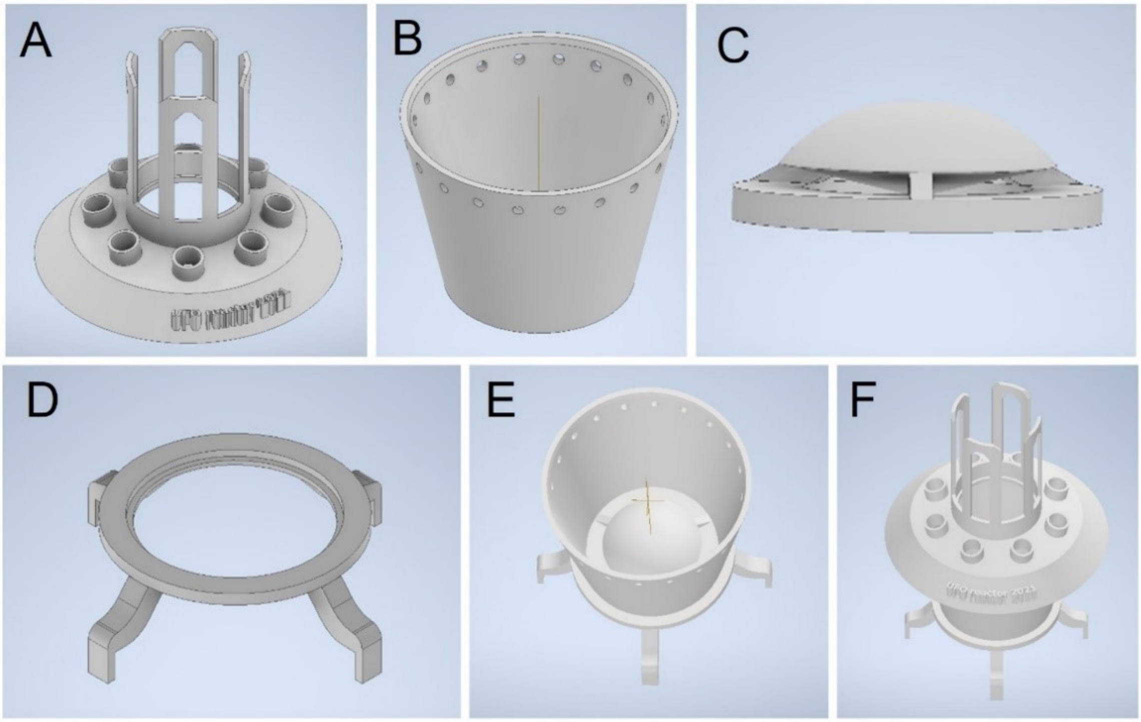

Figure S1: Overview of the 3D-printed reactor: A) lid designed to host up to 8 reactions vials and hold the Kessil lamp in the center; B) body of the reactor; C) light reflector: it is coated with reflective tape; D) adapter for stirring plate; E) inside of the reactor; G) overall reactor. Reprinted with permission from S5 Wan, T.; Capaldo, L.; Ravelli, D.; Vitullo, W.; de Zwart, F. J.; de Bruin, B.; Noël, T. Photoinduced Halogen-Atom Transfer by N-Heterocyclic Carbene-Ligated Boryl Radicals for C(sp^3)-C(sp^3) Bond Formation. J. Am. Chem. Soc. 2023, 145, 991–999. DOI: 10.1021/jacs.2c10444. Copyright © 2022 The Authors.

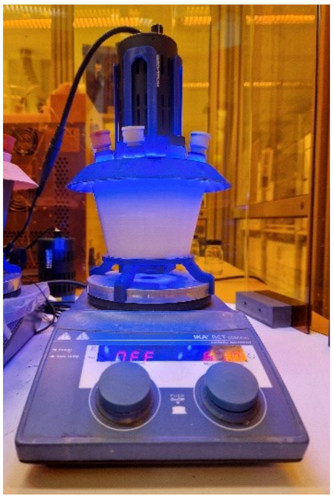

Figure S2: Picture of the assembled reactor equipped with a Kessil lamp (λ_{em} = 456 nm). Reprinted with permission from S5 Wan, T.; Capaldo, L.; Ravelli, D.; Vitullo, W.; de Zwart, F. J.; de Bruin, B.; Noël, T. Photoinduced Halogen-Atom Transfer by N-Heterocyclic Carbene-Ligated Boryl Radicals for C(sp^3)-C(sp^3) Bond Formation. J. Am. Chem. Soc. 2023, 145, 991–999. DOI: 10.1021/jacs.2c10444. Copyright © 2022 The Authors.

For kinetic experiments, the UFO kinetics reactor was used: the only variation compared to the classic UFO reactor is the lid. In particular, the new lid hosts 4 reaction vials (instead of 8) and holds the Kessil lamp in the center. This design was made to ensure that, with a proper alignment of Kessil lamps of the PR160L series (equipped with the linear reflector), the 4 vials experienced the same photonic flux (**Figure S3**). This was found to be crucial to avoid reproducibility issues.

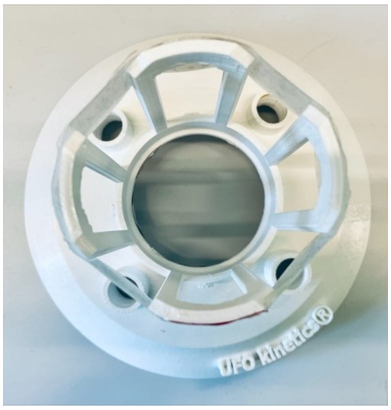

Figure S3: Picture of the lid for the UFO kinetics reactor.

3. Charts of starting materials

Alkyl iodides

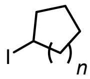

1a, n = 2
1b, n = 1

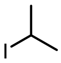

1c

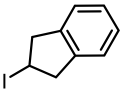

1d

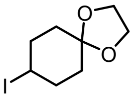

1e

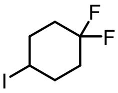

1f

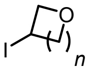

1g, n = 1
1h, n = 2

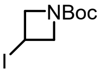

1i

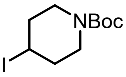

1j

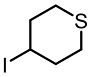

1k

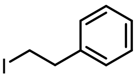

1l

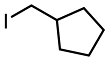

1m

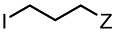

1n (Z = OH)
1o (Z = OPh)
1p (Z = Cl)

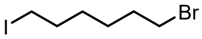

1q

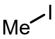

1r

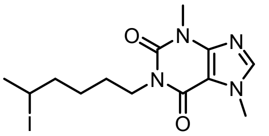

1s

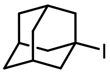

1t

Chloroalkynes

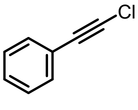

2a

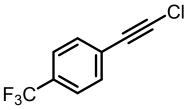

2b

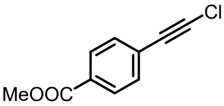

2c

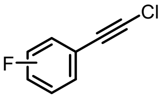

2d, 4-F
2e, 3-F
2f, 2-F

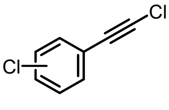

2g, 3-Cl
2h, 4-Cl

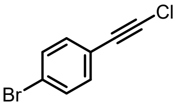

2i

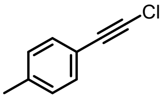

2j

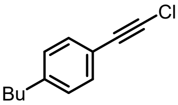

2k

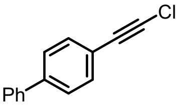

2l

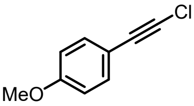

2m

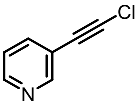

2n

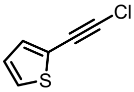

2o

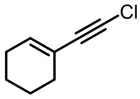

2p

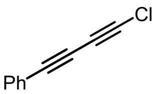

2q

4. Synthesis of starting materials

GP1: Synthesis of alkyl iodides

Alkyl iodides were synthesizing according to a procedure reported in the literature.[2]
In particular, under inert atmosphere (N_2), PPh_3 (1.5 equiv.) and imidazole (1.5 equiv.) were dissolved in CH_2Cl_2 (0.25 M) and the mixture was cooled at 0 °C by means of an ice bath. Next, I_2 (1.5 equiv.) was added and the resulting suspension was stirred for 30 min. Alcohol (1 equiv.) was added dropwise to the reaction mixture and the orange suspension was left stirring for 4 h. After the reaction was judged to be complete (via ^{1}H-NMR), the reaction was quenched by the addition of H_2O; the aqueous layer was extracted twice with CH_2Cl_2 (2×0.75 reaction volume) and the combined organic layers were washed with an aqueous solution of sodium thiosulphate, dried over Na_2SO_4, filtered, and the solvent removed under reduced pressure. The desired compounds was obtained via flash chromatography using pure pentane as eluent.[2]

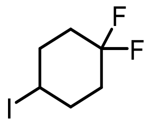

1,1-difluoro-4-iodocyclohexane (1f). Following **GP1** from 4,4-difluorocyclohexan-1-ol (0.50 g, 3.7 mmol). Colorless oil (0.40 g, 44%). ^{1}H NMR (300 MHz, $CDCl_3$) δ 4.53 – 4.40 (m, 1H), 2.27 – 2.00 (m, 6H), 2.00 – 1.80 (m, 2H). ^{13}C NMR (75 MHz, $CDCl_3$) δ: 122.1 (t, J = 242 Hz), 34.7 (t, J = 5 Hz), 33.2 (t, J = 24 Hz), 27.4. ^{19}F NMR (282 MHz, $CDCl_3$) δ -94.27 – -98.53 (m). Spectroscopic data are in accordance with the literature.[3]

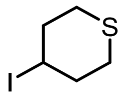

4-Iodotetrahydro-2H-thiopyran (1k). Following **GP1** from tetrahydro-2H-thipyran-4-ol (0.83 g, 7.0 mmol). Colorless oil (1.1 g, 71%). ^{1}H NMR (300 MHz, $CDCl_3$) δ 4.48 (ddd, J_1 = 11 Hz, J_2 = 7 Hz, J_3 = 5 Hz, 1H), 2.87 – 2.69 (m, 2H), 2.63 – 2.49 (m, 2H), 2.43 – 2.20 (m, 4H). ^{13}C NMR (75 MHz, $CDCl_3$) δ 38.8, 31.0, 28.1.
Spectroscopic data are in accordance with the literature.[2]

GP2: Synthesis of chloroalkynes

Chloroalkynes, except **2p**, were prepared by adapting a previously reported procedure in the literature.[4] In particular, an oven-dried (T: 130 °C, overnight) 100 mL Schlenk flask equipped with a magnetic stirrer was charged with *N*-chlorosuccinimide (NCS) (2.67 g, 20.0 mmol, 2.0 equiv.), K_2CO_3 (690 mg, 5.0 mmol, 0.5 equiv.) and Ag_2CO_3 (276 mg, 1.0 mmol, 0.1 equiv.) under inert atmosphere (N_2). Finally, propanol (15 mL) was added. Then, starting material acetylene (10.0 mmol, 1.0 equiv.) was added and the mixture was refluxed for 4 hours. Reaction was monitored via NMR and ultimately quenched with brine at 0 °C. Next, the crude was filtered through a Celite pad using Büchner filtration and washed with pentane (3x30 mL). The crude was extracted with ethyl acetate (1×50 mL), the organic phases were combined, washed with water, dried over Na_2SO_4 and then the solvent was removed under reduced pressure. Purification by flash chromatography on silica gel using pure pentane as eluent gave the desired chloroalkyne product, which was stored in the fridge. **2p** was purified via distillation under vacuum as described in the literature.[5]

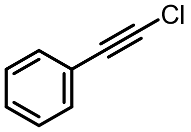

(Chloroethynyl)benzene (2a). Following **GP2** from ethynylbenzene. Colorless oil (0.72 g, 53%). ^{1}H NMR (300 MHz, $CDCl_3$) δ: 7.47 – 7.42 (m, 2H), 7.35 – 7.28 (m, 3H). ^{13}C NMR (75 MHz, $CDCl_3$) δ: 132.1, 128.7, 128.5, 122.3, 69.5, 68.1. Spectroscopic data are in accordance with the literature.[6]

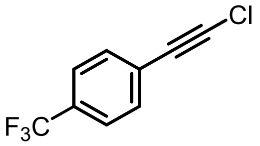

1-(Chloroethynyl)-4-(trifluoromethyl)benzene (2b). Following **GP2** from 1-ethynyl-4-(trifluoromethyl)benzene. Colorless oil (1.04 g, 51%). ^{1}H NMR (400 MHz, $CDCl_3$) δ: 7.59 – 7.53 (m, 4H). ^{13}C NMR (101 MHz, $CDCl_3$) δ: 132.4, 130.6 (q, J = 33 Hz), 126.1, 125.5 (q, J = 4 Hz), 124.0 (q, J = 272 Hz), 71.0, 68.3. ^{19}F NMR (376 MHz, $CDCl_3$) δ: -62.99. Spectroscopic data are in accordance with the literature.[7]

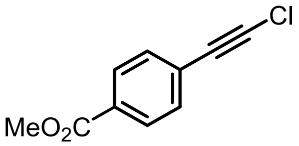

Methyl 4-(chloroethynyl)benzoate (2c). Following **GP2** from methyl 4-ethynylbenzoate. Colorless oil (1.36 g, 70%). ^{1}H NMR (300 MHz, $CDCl_3$) δ 8.04 – 7.93 (m, 2H), 7.60 – 7.44 (m, 2H), 3.91 (s, 3H). ^{13}C NMR (75 MHz, $CDCl_3$) δ 166.5, 132.1, 130.0, 129.6, 126.9, 71.39, 68.9, 52.4. Spectroscopic data are in accordance with the literature.[8]

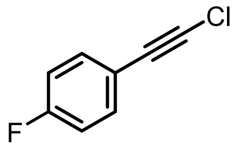

1-(Chloroethynyl)-4-fluorobenzene (2d). Following **GP2** from 1-ethynyl-4-fluorobenzene. Colorless oil (0.61 g, 40%). ^{1}H NMR (300 MHz, $CDCl_3$) δ: 7.42 (dd, J_1 = 9 Hz, J_2 = 5 Hz, 2H), 7.01 (t, J = 9 Hz, 2H). ^{13}C NMR (75 MHz, $CDCl_3$) δ: 162.8 (d, J = 251 Hz), 133.3 (d, J = 8 Hz), 118.34 (d, J = 4 Hz), 115.8 (d, J = 22 Hz), 68.5, 67.9 (d, J = 2 Hz). ^{19}F NMR (282 MHz, $CDCl_3$) δ: -110.18 (tt, J_1 = 8 Hz, J_2 = 5 Hz). Spectroscopic data are in accordance with the literature.[4]

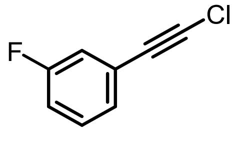

1-(Chloroethynyl)-3-fluorobenzene (2e). Following **GP2** from 1-ethynyl-3-fluorobenzene. Volatile colorless oil (0.40 g, 26%). ^{1}H NMR (400 MHz, $CDCl_3$) δ 7.28 – 7.14 (m, 2H), 7.09 (ddd, J_1 = 9 Hz, J_2 = 3 Hz, J_3 = 2 Hz, 1H), 7.00 (tdd, J_1 = 8 Hz, J_2 = 3 Hz, J_3 = 1 Hz, 1H). ^{13}C NMR (101 MHz, $CDCl_3$) δ 162.4 (d, J = 247 Hz), 130.1 (d, J = 9 Hz), 128.0 (d, J = 3 Hz), 124.08 (d, J = 10 Hz), 119.0 (d, J = 23.0 Hz), 116.2 (d, J = 21 Hz), 69.4, 68.4 (d, J = 4 Hz). ^{19}F NMR (376 MHz, $CDCl_3$) δ -112.65 (td, J_1 = 9 Hz, J_2 = 6 Hz). HRMS (EI) m/z calcd for C_8H_4ClF: 153.9986; found: 153.9996.

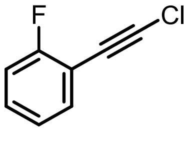

1-(Chloroethynyl)-2-fluorobenzene (2f). Following **GP2** from 1-ethynyl-2-fluorobenzene. Colorless oil (0.65 g, 42%). ^{1}H NMR (400 MHz, $CDCl_3$) δ: 7.47 – 7.41 (m, 1H), 7.36 – 7.28 (m, 1H), 7.12 – 7.04 (m, 2H). ^{13}C NMR (101 MHz, $CDCl_3$) δ: 163.5 (d, J = 252 Hz), 134.0 (d, J = 1 Hz), 130.4 (d, J = 8 Hz), 124.1 (d, J = 4 Hz), 115.7 (d, J = 21 Hz), 110.9 (d, J = 16 Hz), 73.1 (d, J = 3 Hz), 63.3 (d, J = 1 Hz). ^{19}F NMR (376 MHz, $CDCl_3$) δ: -110.23 (ddd, J_1 = 10 Hz, J_2 = 7 Hz, J_3 = 5 Hz). Spectroscopic data are in accordance with the literature.[9]

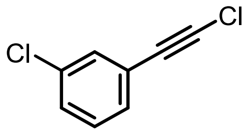

1-Chloro-3-(chloroethynyl)benzene (2g). Following **GP2** from 1-chloro-3-ethynylbenzene. Colorless oil (1.21 g, 71%). ^{1}H NMR (300 MHz, $CDCl_3$) δ 7.43 (t, J = 2 Hz, 1H), 7.32 (ddd, J_1 = 7 Hz, J_2 = 2 Hz, J_3 = 1 Hz, 2H), 7.27 – 7.19 (m, 1H). ^{13}C NMR (75 MHz, $CDCl_3$) δ 134.4, 132.0, 130.2, 129.7, 129.1, 124.0, 69.7, 68.2. Spectroscopic data are in accordance with the literature.[4]

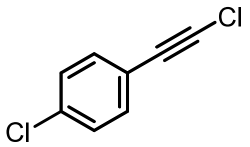

1-Chloro-4-(chloroethynyl)benzene (2h). Following **GP2** from 1-chloro-4-ethynylbenzene. White solid (1.29 g, 75%). ^{1}H NMR (400 MHz, $CDCl_3$) δ 7.37 (d, J = 9 Hz, 2H), 7.29 (d, J = 9 Hz, 2H). ^{13}C NMR (101 MHz, $CDCl_3$) δ 134.8, 133.3, 128.9, 120.8, 69.3, 68.5. Spectroscopic data are in accordance with the literature.[7]

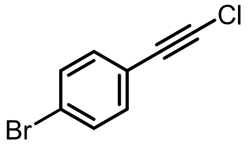

1-Bromo-4-(chloroethynyl)benzene (2i). Following **GP2** from 1-bromo-4-ethynylbenzene. White solid (1.24 g, 58%). ^{1}H NMR (300 MHz, $CDCl_3$) δ 7.45 (d, J = 9 Hz, 2H), 7.30 (d, J = 9 Hz, 2H). ^{13}C NMR (75 MHz, $CDCl_3$) δ 133.5, 131.8, 123.1, 121.2, 69.5, 68.6. Spectroscopic data are in accordance with the literature.[6]

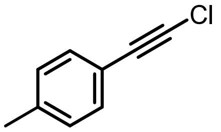

1-(Chloroethynyl)-4-methylbenzene (2j). Following **GP2** from 1-ethynyl-4-methylbenzene. Colorless oil (0.72 g, 48%). ^{1}H NMR (300 MHz, $CDCl_3$) δ 7.98 (d, J = 9 Hz, 2H), 7.49 (d, J = 9 Hz, 2H), 3.91 (s, 3H). ^{13}C NMR (75 MHz, $CDCl_3$) δ: 138.9, 132.0, 129.3, 119.2, 69.6, 67.3, 21.6. Spectroscopic data are in accordance with the literature.[6]

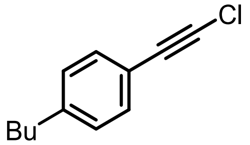

1-Butyl-4-(chloroethynyl)benzene (2k). Following **GP2** from 1-butyl-4-ethynylbenzene. Colorless oil (1.45 g, 75%). ^{1}H NMR (300 MHz, $CDCl_3$) δ 7.39 (d, J = 8 Hz, 2H), 7.15 (d, J =

8 Hz, 2H), 2.79 – 2.50 (m, 2H), 1.69 – 1.54 (m, 2H), 1.46 – 1.29 (m, 2H), 1.05 – 0.83 (m, 3H). ^{13}C NMR (75 MHz, $CDCl_3$) δ 143.8, 132.0, 128.6, 119.4, 69.7, 67.2, 35.7, 33.5, 22.5, 14.0. Spectroscopic data are in accordance with the literature.[9]

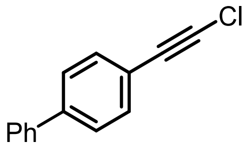

4-(Chloroethynyl)-1,1'-biphenyl (2l). Following **GP2** from 4-ethynyl-1,1'-biphenyl. White solid (1.42 g, 67%). ^{1}H NMR (300 MHz, $CDCl_3$) δ 7.72 – 7.38 (m, 9H). ^{13}C NMR (75 MHz, $CDCl_3$) δ 141.4, 140.3, 132.5, 129.0, 127.8, 127.1, 127.1, 121.1, 69.5, 68.7. Spectroscopic data are in accordance with the literature.[7]

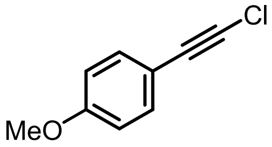

1-(Chloroethynyl)-4-methoxybenzene (2m). Following **GP2** from 1-ethynyl-4-methoxybenzene. Colorless oil (0.73 g, 44%). ^{1}H NMR (300 MHz, $CDCl_3$) δ: 7.40 – 7.35 (m, 2H), 6.86 – 6.81 (m, 2H), 3.81 (s, 3H). ^{13}C NMR (75 MHz, $CDCl_3$) δ: 159.9, 133.5, 114.3, 114.1, 69.4, 66.5, 55.4. Spectroscopic data are in accordance with the literature.[6]

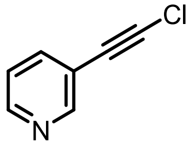

3-(Chloroethynyl)pyridine (2n). Prepared by following a procedure reported in the literature[6] from 3-ethynylpyridine. Yellowish liquid (0.82 g, 60%). ^{1}H NMR (300 MHz, $CDCl_3$) δ 8.68 (dd, J_1 = 2 Hz, J_2 = 1 Hz, 1H), 8.55 (dd, J_1 = 5 Hz, J_2 = 2 Hz, 1H), 7.72 (dt, J_1 = 8 Hz, J_2 = 2 Hz, 1H), 7.30 – 7.19 (m, 1H). ^{13}C NMR (75 MHz, $CDCl_3$) δ 152.8, 149.1, 139.1, 123.2, 119.6, 71.8, 66.4. Spectroscopic data are in accordance with the literature.[6]

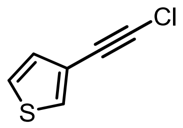

3-(Chloroethynyl)thiophene (2o). To a solution of 3-ethynylthiophene (1 g, 9.2 mmol) in tetrahydrofuran (12 mL) was added dropwise 2.5 M n-butyl lithium/ hexane solution (4.4 mL, 11 mmol) at -78°C, and the mixture was stirred at -78°C for 1 h. To the reaction mixture was added dropwise a suspension of *N*-chlorosuccinimide (1.4 g, 10 mmol) in tetrahydrofuran (30 mL), and the mixture was stirred at -78°C to room temperature for 12 h. To the reaction mixture was added hexane, and the mixture was washed with saturated brine and 1.0 M aqueous sodium

thiosulfate, dried over anhydrous magnesium sulfate and concentrated. The concentrated residue was purified by column chromatography (pentane) to give **2o**, light yellow oil (0.70 g, 54%). ^{1}H NMR (300 MHz, $CDCl_3$) δ 7.49 (dd, J_1 = 3 Hz, J_2 = 1 Hz, 1H), 7.35 – 7.24 (m, 1H), 7.13 (dd, J_1 = 5 Hz, J_2 = 1 Hz, 1H). ^{13}C NMR (75 MHz, $CDCl_3$) δ 130.0, 129.7, 125.5, 121.1, 67.8, 64.9. HRMS (EI) m/z calcd for C_6H_3ClS: 141.9644; found: 141.9653.

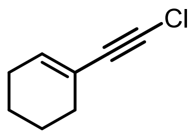

1-(Chloroethynyl)cyclohex-1-ene (2p). Prepared by following a procedure reported in the literature[5] from 1-ethynylcyclohex-1-ene on 10 mmol scale. Colorless liquid (0.37 g, 26%). ^{1}H NMR (400 MHz, $CDCl_3$) δ 6.17 – 6.08 (m, 1H), 2.14 – 2.02 (m, 4H), 1.70 – 1.51 (m, 4H). ^{13}C NMR (101 MHz, $CDCl_3$) δ 136.2, 119.9, 71.3, 65.1, 29.1, 25.7, 22.3, 21.5. Spectroscopic data are in accordance with the literature.[5]

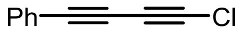

(Chlorobuta-1,3-diyn-1-yl)benzene (2q). Prepared via a three-steps procedure adopted from the literature.

Step 1:[10] Phenylethynyl chloride (1.0 equiv., 500 mg, 3.66 mmol) was dissolved in THF (50 mL) and diisopropylamine (20 mL). To the solution were added $Pd(PPh_3)_4$ (5 mol%, 0.18 mmol, 208 mg), CuI (5 mol%, 0.18 mmol, 34 mg) and trimethylsilylacetylene (3 equiv., 1.56 mL, 11 mmol). The reaction was stirred at rt for 24 h and at 50 °C for 4 h. Saturated aqueous NH_4Cl (25 mL) was added, and the mixture was extracted with dichloromethane, washed with brine, dried over Na_2SO_4, filtered and concentrated under reduced pressure. Purification by flash column chromatography on silica gel (Heptane) gave the title compound as a white solid (359 mg, 49% yield). ^{1}H NMR (300 MHz, $CDCl_3$) δ 7.51 – 7.46 (m, 2H), 7.38 – 7.29 (m, 3H), 0.23 (s, 9H). ^{13}C NMR (75 MHz, $CDCl_3$) δ 132.8, 129.5, 128.6, 121.5, 90.8, 87.9, 76.9, 74.3, -0.2. Spectroscopic data are in accordance with the literature.[10]

Step 2:[11] A 50 mL round-bottomed flask was charged with trimethyl(phenylbuta-1,3-diyn-1-yl)silane (1.0 equiv., 359 mg, 1.81 mmol), MeOH (5.5 mL), and CsF (3.0 equiv., 825 mg, 5.4 mmol). The mixture was stirred for 2 h at room temperature, then evaporated under reduced pressure to remove MeOH. Purification by flash column chromatography on silica gel (Heptane) gave the title compound as a white solid (192 mg, 85% yield). ^{1}H NMR (300 MHz, $CDCl_3$) δ 7.57 – 7.47 (m, 2H), 7.43 – 7.29 (m, 3H), 2.48 (s, 1H). ^{13}C NMR (75 MHz, CDCl3)

δ 132.9, 129.7, 128.6, 121.2, 75.5, 73.6, 71.4, 68.3. Spectroscopic data are in accordance with the literature.[11]

Step 3:[12] A flame-dried Schlenk flask was charged with K_2CO_3 (0.5 equiv., 0.4 mmol, 55 mg), Ag_2CO_3 (0.1 equiv., 0.8 mmol, 22 mg) and NCS (2.0 equiv., 0.8 mmol, 212 mg). The flask was evacuated and backfilled with N_2 and the solids were dispersed in *n*-propanol (1.6 mL) and buta-1,3-diyn-1-ylbenzene (1.0 equiv., 0.79 mmol, 100 mg) was added dropwise. The flask was stirred at 50 °C for 4 h. The mixture was allowed to cool to room temperature, quenched with brine at 0 °C and extracted with ethyl ether (3×). The combined organic phase was washed water and dried with Na_2SO_4 and the solvent was removed under reduced pressure. Purification by flash column chromatography on silica gel (Heptane) to afford the product as a brown oil (35 mg, 27% yield). ^{1}H NMR (300 MHz, $CDCl_3$) δ 7.55 – 7.47 (m, 2H), 7.42 – 7.31 (m, 3H). ^{13}C NMR (75 MHz, $CDCl_3$) 132.9, 129.6, 128.6, 121.3, 74.3, 73.7, 61.6, 55.5.
Spectroscopic data are in accordance with the literature.[12]

5. Optimization of reaction conditions

1. Optimization of reaction conditions

The optimization of the reaction conditions was carried out by studying the radical addition of iodocyclohexane (**1a**) onto (chloroethynyl)benzene (**2a**) to give (2-chloro-2-cyclohexylvinyl)benzene (**3**) on a 0.1 mmol scale (see Table S1-7). In a 7 mL vial equipped with a screw cap **2a** (0.1 mmol), **1a** (n equiv.), **B1** (n equiv.) and the chosen photocatalyst (n mol%) were dissolved in 1.0 mL of the chosen solvent. The mixture was bubbled with N_2 (1 min) and irradiated for the indicated time with a 40 W Kessil lamp (λ = 456 nm, full intensity) for the required time in the UFO reactor (**Figure S1**). After irradiation, dibromomethane was added to the reaction crude and the sample was analyzed via ^{1}H-NMR.

Table S 1: Screening of photocatalysts.

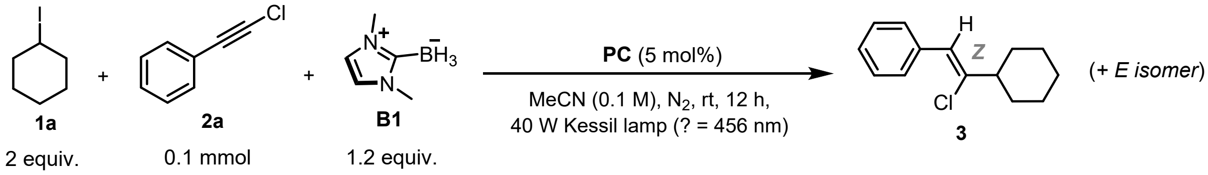

Entry	Photocatalyst	Yield (%)[a]
**1**	Mes-AcrClO$_4$	26 (*Z*:*E* 93:7)
**2**	4CzIPN	30 (*Z*:*E* 92:8)
**3**	Ru(bpy)$_3$(PF$_6$)$_2$	10 (*Z*:*E* > 95:5)
**4**	Eosin Y	14 (*Z*:*E* >95:5)

[a] Yields determined by ^{1}H-NMR, dibromomethane as external standard.

Table S 2: Screening of substrates ratio.

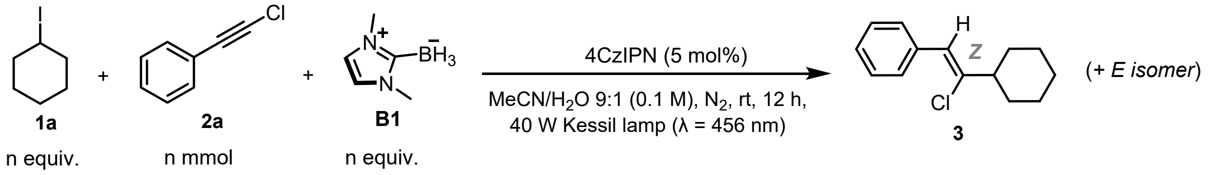

Entry	Substrates ratio (1a:2a:B1)	Yield (%)[a]
**1**	2:1:1.2	56 (*Z*:*E* 93:7)
**2**	1:1:1	40 (*Z*:*E* 91:9)
**3**	1.5:1:1.2	52 (*Z*:*E* 92:8)
**4**	1:1.2:1.2	48 (*Z*:*E* 92:8)

**5**	2:1:1.5	68 (*Z*:*E* 89:11)

[a] Yields determined by ^{1}H-NMR, dibromomethane as external standard.

Table S 3: Screening of atmosphere.

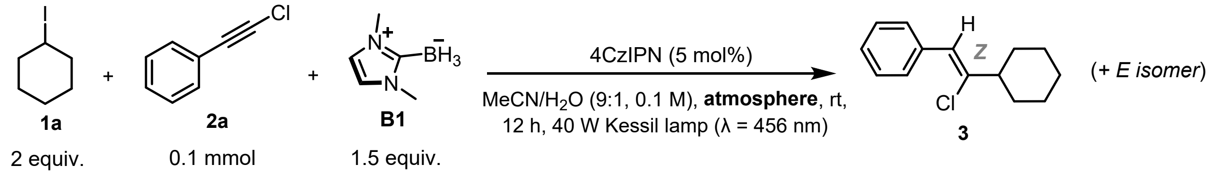

Entry	Atmosphere	Yield (%)[a]
**1**	N_2	68 (*Z*:*E* 89:11)
**2**	Air-equilibrated	58 (*Z*:*E* 93:7)

[a] Yields determined by ^{1}H-NMR, dibromomethane as external standard.

Table S 4: Screening of solvents and photocatalyst loading.

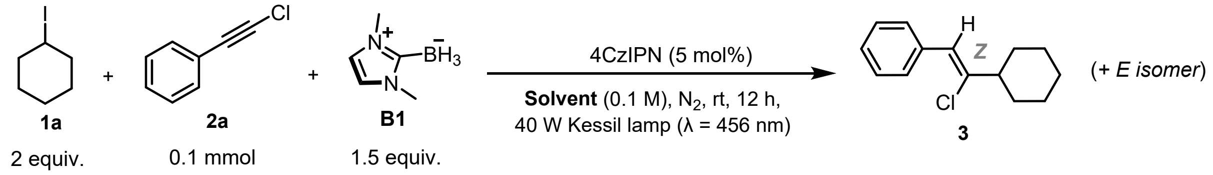

Entry	Solvent	Yield (%)[a]
**1**	MeCN/H_2O (9:1)	68 (*Z*:*E* 89:11)
**2**	EtOAc	54 (*Z*:*E* 76:14)
**3**	CH_3CN	32 (*Z*:*E* 94:6)
**4**	$PhCH_3$	n.d.
**5**	CH_3OH	46 (*Z*:*E* 91:9)
**6**	DMSO	50 (*Z*:*E* >95:5)
**8**	As entry 1, 4CzIPN (2 mol%)	70 (*Z*:*E* 86:14)

[a] Yields determined by ^{1}H-NMR, dibromomethane as external standard.

Table S 5: Screening of bases.

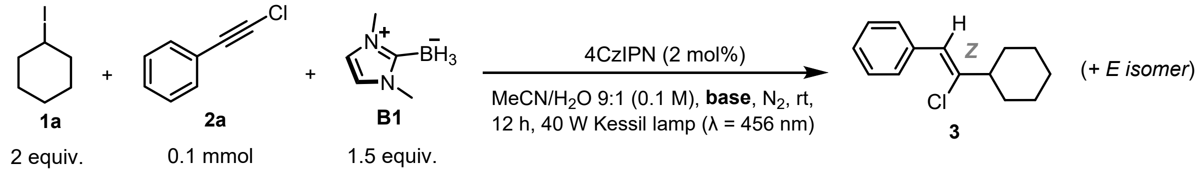

Entry	Base	Yield (%)a
**1**	K_3PO_4 (1.2 equiv.)	81 (*Z*:*E* 80:20)
**2**	NaOAc (1.2 equiv.)	82 (*Z*:*E* 66:33)

**3**	Cs_2CO_3 (1.2 equiv.)	79 (Z:E 80:20)
**4**	K_3PO_4 (0.5 equiv.)	82 (*Z:E* 75:25)

[a] Yields determined by ^{1}H-NMR, dibromomethane as external standard

Table S 6: Screening of photocatalyst loading and reaction time.

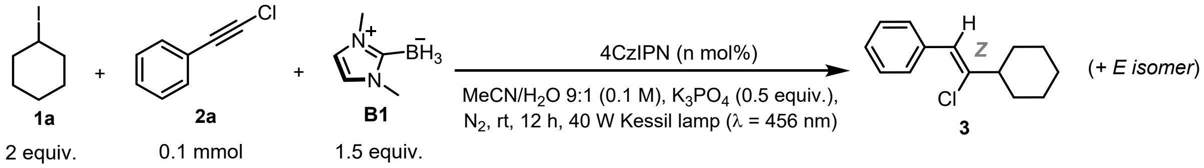

Entry	Photocatalyst loading	Yield (%)[a]
**1**	4CzIPN (2 mol%)	82 (*Z*:*E* 75:25)
**2**	4CzIPN (0.5 mol%)	86 (*Z*:*E* 80:20)
**3**	As entry 2, 6 h	82 (*Z*:*E* 92:8)

[a] Yields determined by ^{1}H-NMR, dibromomethane as external standard.

Table S 7: Control experiments.

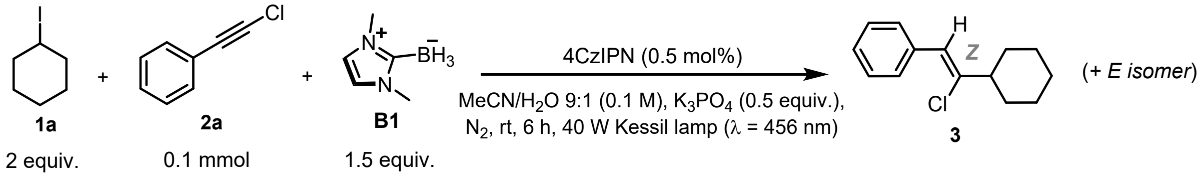

Entry	Variations from conditions	Yield (%)[a]
**1**	Without light	n.d.
**2**	Without 4CzIPN	n.d.
**3**	Without **B1**	n.d.
**4**	Heating at 80°C in the dark	n.d.

[a] Yields determined by ^{1}H-NMR, dibromomethane as external standard.

6. NOESY experiments to determine double bond geometry

We performed NOESY experiments on model substrates to ascertain the geometry of C=C double bonds in vinyl chlorides. Geometry of the other compounds was assigned based on these results.

**Structure *Z*-isomer:**	**NOESY:**	**Structure *E*-isomer:**	**NOESY:**
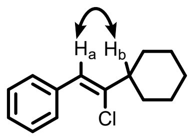	Correlation between: H_a/H_b	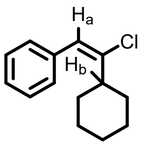	No correlation between: H_a/H_b
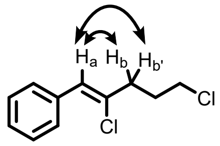	Correlation between: H_a/H_b, $H_a/H_{b'}$	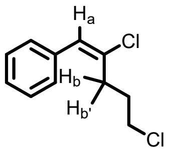	No correlation between: H_a/H_b, $H_a/H_{b'}$

^{1}H-^{1}H NOESY (400 MHz, $CDCl_3$) of compound **3** (major, Z):

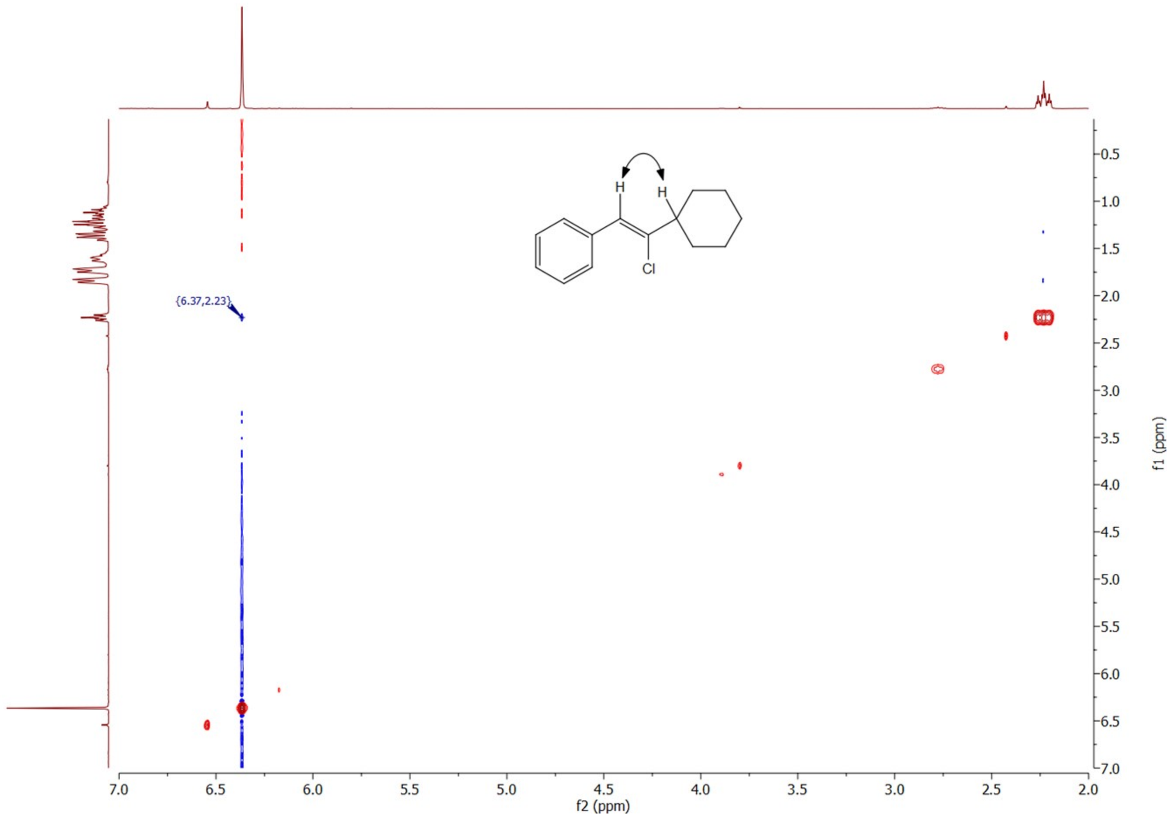

^{1}H-^{1}H NOESY (400 MHz, $CDCl_3$) of compound **18** (major, Z):

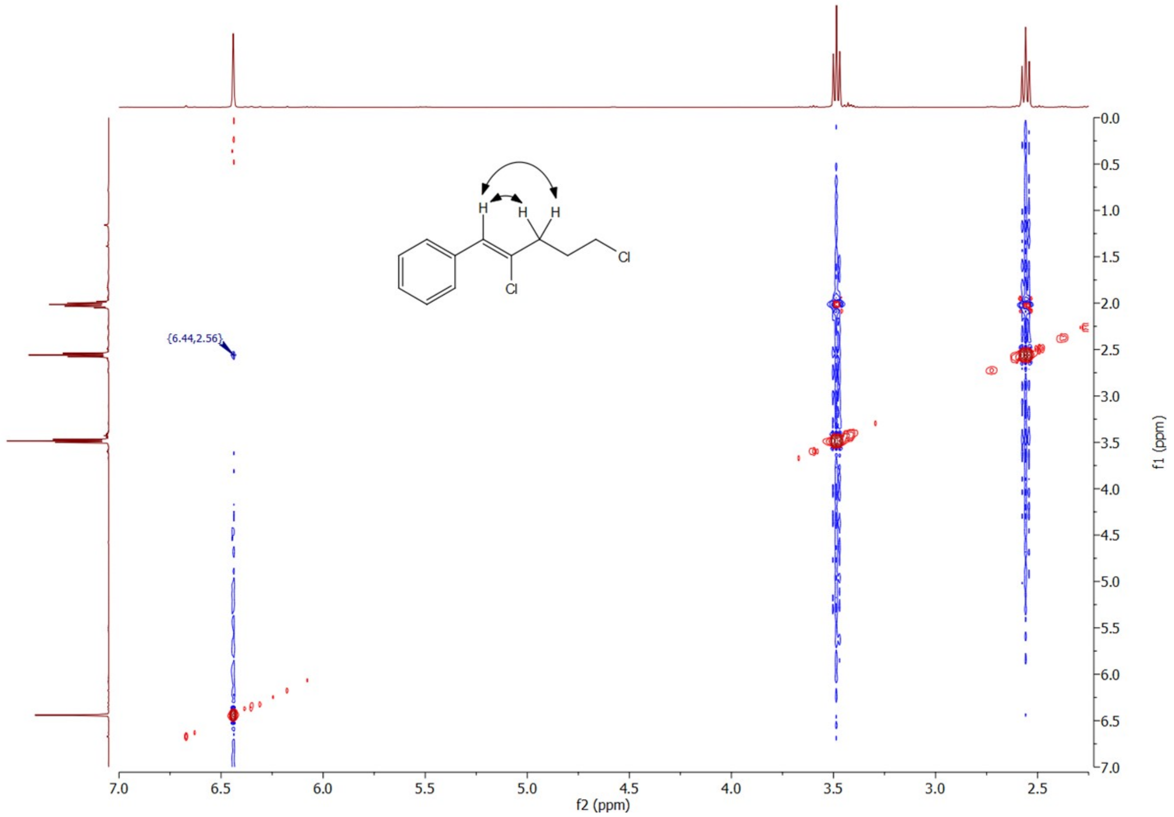

7. General Procedures for the Synthesis of Vinyl Chlorides

GP3: Procedure for secondary alkyl iodides

A CH_3CN/H_2O 9:1 (0.1 M) solution containing the chloroalkyne **2** (0.5 mmol), alkyl iodide **1** (2.0 equiv.), **B1** (1.5 equiv.), K_3PO_4 (0.5 equiv) and 4CzIPN (0.5 mol%) was prepared in a 7 mL vial equipped with a screw cap and a stirring bar. The solution was bubbled with N_2 (for 5 min) and then irradiated by adopting the UFO reactor setup equipped with a 40 W Kessil lamp (λ = 456 nm, full intensity) for 6 h. The solutions were collected, solvent was removed under reduced pressure and the crude was purified via column chromatography on silica gel to provide the expected product.

GP4: Procedure for primary alkyl iodides

A CH_3CN/H_2O 9:1 (0.1 M) solution containing the chloroalkyne **2** (1.5 equiv.), organic halide **1** (0.5 mmol), **B1** (1.5 equiv.), K_3PO_4 (0.5 equiv.) and 4CzIPN (0.5 mol%) was prepared in a 7 mL vial equipped with a screw cap and a stirring bar. The solution was bubbled with N_2 (for 5 min) and then irradiated by adopting the UFO reactor setup equipped with a 40 W Kessil lamp (λ = 456 nm, full intensity) for 18 h. The solutions were collected, solvent was removed under reduced pressure and the crude was purified via column chromatography on silica gel to provide the expected product.

8. Characterization data

N.B.: A complete characterization of the two isomers was reported when Z:E ratio was found to be lower than 80:20 as determined by ^{1}H-NMR of the crude.

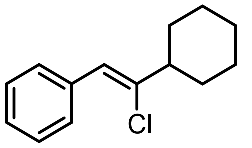

(2-Chloro-2-cyclohexylvinyl)benzene (3). Prepared according to **GP3**. Purified by flash column chromatography on silica gel (Pentane) to afford the product as a colourless liquid (80 mg, 73% combined yield). (*Z*:*E* = 92:8) *Major (Z)*: ^{1}H NMR (300 MHz, $CDCl_3$) δ: 7.61 (d, *J* = 8 Hz, 2H), 7.38 – 7.24 (m, 3H), 6.49 (s, 1H), 2.36 (tt, J_1 = 11 Hz, J_2 = 3 Hz, 1H), 2.03 – 1.64 (m, 5H), 1.54 – 1.15 (m, 5H). ^{13}C NMR (75 MHz, $CDCl_3$) δ: 140.7, 135.6, 129.3 (2C), 128.2 (2C), 127.4, 122.4, 48.9, 31.8 (2C), 26.3 (2C), 26.1. Spectroscopic data are in accordance with the literature.[S7]

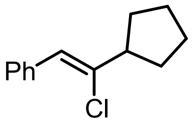

(2-Chloro-2-cyclopentylvinyl)benzene (4). Prepared according to **GP3**. Purified by flash column chromatography on silica gel (Heptane) to afford the product as a greenish clear liquid (98 mg, 95% combined yield). (*Z*:*E* = 94:6) *Major (Z)*: ^{1}H NMR (300 MHz, $CDCl_3$) δ: 7.61 (d, *J* = 7 Hz, 2H), 7.40 – 7.23 (m, 3H), 6.57 (s, 1H), 2.99 – 2.89 (m, 1H), 1.99 – 1.58 (m, 8H). ^{13}C NMR (75 MHz, $CDCl_3$) δ: 139.1, 135.5, 129.2 (2C), 128.2 (2C), 127.4, 122.9, 50.2, 31.6 (2C), 25.7 (2C). HRMS (FI) m/z calcd for $C_{13}H_{15}Cl$: 206.0862; found: 206.0863.

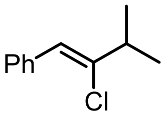

(2-Chloro-3-methylbut-1-en-1-yl)benzene (5). Prepared according to **GP3**. Purified by flash column chromatography on silica gel (Pentane) to afford the product as a colourless liquid (79 mg, 88% combined yield). (*Z*:*E* = 95:5) *Major (Z)*: ^{1}H NMR (300 MHz, $CDCl_3$) δ: 7.62 (d, *J* = 7 Hz, 2H), 7.39 – 7.25 (m, 3H), 6.52 (s, 1H), 2.83 – 2.69 (m, 1H), 1.25 (d, *J* = 3 Hz, 6H). ^{13}C NMR (75 MHz, $CDCl_3$) δ: 141.6, 135.4, 129.3 (2C), 128.2 (2C), 127.5, 122.2, 39.0, 21.4 (2C). HRMS (FI) m/z calcd for $C_{11}H_{13}Cl$: 180.0706; found: 180.0713.

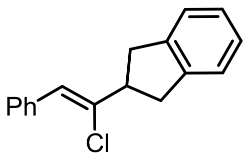

2-(1-Chloro-2-phenylvinyl)-2,3-dihydro-1H-indene (6). Prepared according to **GP3**. Purified by flash column chromatography on silica gel (Heptane) to afford the product as a colourless liquid (73 mg, 57% combined yield). (*Z*:*E* = 94:6) *Major (Z)*: ^{1}H NMR (300 MHz, $CDCl_3$) δ: 7.67 (d, *J* = 7 Hz, 2H), 7.43 – 7.16 (m, 7H), 6.69 (s, 1H), 3.67 – 3.55 (m, 1H), 3.35 – 3.16 (m, 4H). ^{13}C NMR (75 MHz, $CDCl_3$) δ: 142.3 (2C), 137.4, 135.1, 129.3 (2C), 128.3 (2C), 127.7, 126.6 (2C), 124.5 (2C), 124.2, 50.1, 38.0 (2C). HRMS (FI) m/z calcd for $C_{11}H_{13}Cl$: 254.0862; found: 254.0862.

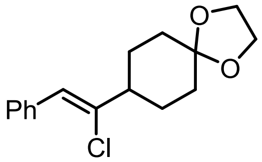

8-(1-Chloro-2-phenylvinyl)-1,4-dioxaspiro[4.5]decane (7). Prepared according to **GP3**, **1e** (1.5 equiv.). Purified by flash column chromatography on silica gel (Pentane:Ethyl Acetate 100:0 → 90:10) to afford the product as a colourless solid (102 mg, 73% combined yield), m.p. 61.9~63.5 °C. (*Z*:*E* = 93:7) *Major (Z)*: ^{1}H NMR (300 MHz, $CDCl_3$) δ: 7.58 (d, *J* = 7 Hz, 2H), 7.37 – 7.22 (m, 3H), 6.52 (s, 1H), 3.97 (s, 4H), 2.40 (tt, J_1 = 11 Hz, J_2 = 3 Hz, 1H) 1.99 – 1.76 (m, 6H), 1.68 – 1.58 (m, 2H). ^{13}C NMR (75 MHz, $CDCl_3$) δ: 139.1, 135.3, 129.3 (2C), 128.2 (2C), 127.6, 123.0, 108.4, 64.5 (2C), 47.5, 34.6 (2C), 28.9 (2C). HRMS (FI) m/z calcd for $C_{16}H_{19}ClO_2$: 278.1074; found: 278.1077.

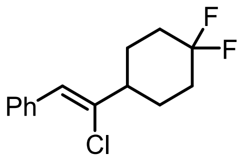

(2-Chloro-2-(4,4-difluorocyclohexyl)vinyl)benzene (8). Prepared according to **GP2**, **1l** (1.5 equiv.). Purified by flash column chromatography on silica gel (Pentane:Ethyl Acetate 100:0 → 90:10) to afford the product as a colourless solid (104 mg, 81% combined yield), m.p. 50.9~53.6 °C. (*Z*:*E* = 94:6) *Major (Z)*: ^{1}H NMR (300 MHz, $CDCl_3$) δ: 7.61 (d, *J* = 7 Hz, 2H), 7.41 – 7.27 (m, 3H), 6.55 (s, 1H), 2.50 – 2.41 (m, 1H), 2.27 – 2.17 (m, 2H), 2.04 – 1.71 (m, 6H). ^{13}C NMR (75 MHz, $CDCl_3$) δ: 137.7 (d, *J* = 3 Hz), 135.0, 129.3 (2C), 128.3 (2C), 127.8, 123.7, 122.9 (dd, J_1 = 243 Hz, J_2 = 240 Hz, 2C), 46.7 (d, *J* = 2 Hz), 33.5 (dd, J_1 = 26 Hz, J_2 = 23 Hz, 2C), 27.7 (d, *J* = 10 Hz). ^{19}F NMR (376 MHz, $CDCl_3$) δ: -92.0 (d, *J* = 236 Hz, 1F), -

102.4 (dtt, J_1 = 237 Hz, J_2 = 34 Hz, J_3 = 10 Hz, 1F). HRMS (FI) m/z calcd for $C_{14}H_{15}ClF_2$: 256.0830; found: 256.0829.

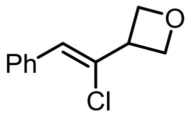

3-(1-Chloro-2-phenylvinyl)oxetane (9). Prepared according to **GP3**. Reaction time: 8 h. Purified by flash column chromatography on silica gel (Heptane:Ethyl Acetate 100:0 → 93:7) to afford the product as a colourless oil (46 mg, 47% combined yield). (*Z*:*E* = 93:7) *Major (Z)*: ^{1}H NMR (300 MHz, $CDCl_3$) δ: 7.62 (d, *J* = 7 Hz, 2H), 7.40 – 7.27 (m, 3H), 6.54 (s, 1H), 4.95 – 4.84 (m, 4H), 4.21 – 4.11 (m, 1H). ^{13}C NMR (75 MHz, $CDCl_3$) δ: 134.4, 132.8, 129.4 (2C), 128.4 (2C), 128.2, 125.6, 75.1 (2C), 44.2. HRMS (FI) m/z calcd for $C_{11}H_{11}ClO$: 194.0498; found: 194.0508.

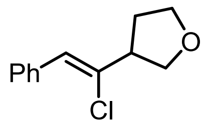

3-(1-Chloro-2-phenylvinyl)tetrahydrofuran (10). Prepared according to **GP3**. Purified by flash column chromatography on silica gel (Heptane:Ethyl Acetate 100:0 → 90:10) to afford the product as a colourless liquid (83 mg, 80% combined yield). (*Z*:*E* = 93:7) *Major (Z)*: ^{1}H NMR (300 MHz, $CDCl_3$) δ: 7.60 (d, *J* = 7 Hz, 2H), 7.39 – 7.25 (m, 3H), 6.60 (s, 1H), 4.06 – 3.82 (m, 4H), 3.38 – 3.27 (m, 1H), 2.18 (q, *J* = 7 Hz, 2H). ^{13}C NMR (75 MHz, $CDCl_3$) δ: 134.8, 134.5, 129.2 (2C), 128.3 (2C), 127.9, 125.1, 71.4, 68.6, 49.2, 31.4. HRMS (FI) m/z calcd for $C_{12}H_{13}ClO$: 208.0655; found: 208.0661.

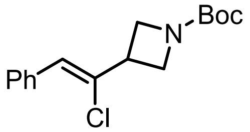

***tert*-Butyl 3-(1-chloro-2-phenylvinyl)azetidine-1-carboxylate (11).** Prepared according to **GP2**. Reaction time: 8 h. Purified by flash column chromatography on silica gel (Heptane:Ethyl Acetate 100:0 → 90:10) to afford the product as a colourless oil (81 mg, 55% combined yield). (*Z*:*E* = 88:12) *Major (Z)*: ^{1}H NMR (300 MHz, $CDCl_3$) δ: 7.60 (d, *J* = 7 Hz, 2H), 7.39 – 7.28 (m, 3H), 6.57 (s, 1H), 4.13 (d, *J* = 4 Hz, 4H), 3.68 – 3.59 (m, 1H), 1.46 (s, 9H). ^{13}C NMR (75 MHz, $CDCl_3$) δ: 156.3, 134.4, 133.5, 129.2 (2C), 128.4 (2C), 128.2, 125.7, 79.8, 53.1 (2C), 38.0, 28.5 (3C). HRMS (FI) m/z calcd for $C_{16}H_{20}ClNO_2$: 293.1183; found: 293.1195.

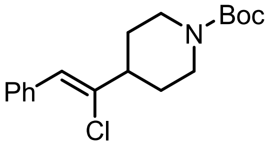

***tert*-Butyl 4-(1-chloro-2-phenylvinyl)piperidine-1-carboxylate (12).** Prepared according to **GP3**. Purified by flash column chromatography on silica gel (Heptane:Ethyl Acetate 100:0 → 90:10) to afford the product as a colourless oil (130 mg, 81% combined yield). (*Z*:*E* = 92:8) *Major (Z)*: ^{1}H NMR (300 MHz, $CDCl_3$) δ: 7.58 (d, *J* = 7 Hz, 2H), 7.37 – 7.23 (m, 3H), 6.50 (s, 1H), 4.24 (d, *J* = 5 Hz, 2H), 2.75 (t, *J* = 13 Hz, 2H), 2.48 (tt, J_1 = 12 Hz, J_2 = 4 Hz, 1H), 1.89 (d, *J* = 6 Hz, 2H), 1.65 (qd, J_1 = 13 Hz, J_2 = 4 Hz, 2H), 1.48 (s, 9H). ^{13}C NMR (75 MHz, $CDCl_3$) δ: 154.8, 138.4, 135.1, 129.3 (2C), 128.3 (2C), 127.7, 123.4, 79.7, 47.0, 43.8 (2C), 30.7 (2C), 28.6 (3C). HRMS (FI) m/z calcd for $C_{18}H_{24}ClNO_2$: 321.1496; found: 321.1497.

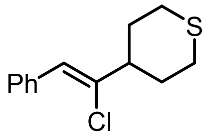

4-(1-Chloro-2-phenylvinyl)tetrahydro-2H-thiopyran (13). Prepared according to **GP3**. Reaction time: 16 h. Purified by flash column chromatography on silica gel (Pentane:Ethyl Acetate 100:0 → 90:10) to afford the product as a colourless oil (66 mg, 55% combined yield). (*Z*:*E* = 89:11) *Major (Z)*: ^{1}H NMR (300 MHz, $CDCl_3$) δ: 7.60 (d, *J* = 7 Hz, 2H), 7.38 – 7.21 (m, 3H), 6.49 (s, 1H), 2.99 – 2.64 (m, 4H), 2.38 (tt, J_1 = 12 Hz, J_2 = 3 Hz, 1H), 2.24 (dd, J_1 = 14 Hz, J_2 = 2 Hz, 2H), 1.92 (qd, J_1 = 12 Hz, J_2 = 4 Hz, 2H). ^{13}C NMR (75 MHz, $CDCl_3$) δ: 139.1, 135.1, 129.2 (2C), 128.3 (2C), 127.7, 123.4, 48.6, 32.8 (2C), 28.8 (2C). HRMS (FI) m/z calcd for $C_{13}H_{15}ClS$: 238.0583; found: 238.0584.

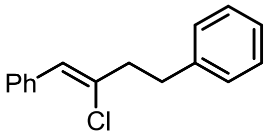

(2-Chlorobut-1-ene-1,4-diyl)dibenzene (14). Prepared according to **GP4**. Purified by flash column chromatography on silica gel (Pentane) to afford the product as a colourless liquid (54 mg, 45% combined yield). (*Z*:*E* = 90:10) *Major (Z)*: ^{1}H NMR (300 MHz, $CDCl_3$) δ: 7.55 (d, *J* = 7 Hz, 2H), 7.38 – 7.20 (m, 8H), 6.40 (s, 1H), 3.01 (t, *J* = 8 Hz, 2H), 2.79 (t, *J* = 8 Hz, 2H). ^{13}C NMR (75 MHz, $CDCl_3$) δ: 140.8, 135.2, 133.9, 129.1 (2C), 128.7 (2C), 138.6 (2C), 128.3 (2C), 127.6, 126.3, 125.3, 43.3, 34.2. HRMS (FI) m/z calcd for $C_{16}H_{15}Cl$: 242.0862; found: 242.0857.

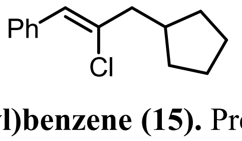

(2-Chloro-3-cyclopentylprop-1-en-yl)benzene (15). Prepared according to **GP4**. Purified by flash column chromatography on silica gel (Pentane) to afford the product as a colourless liquid (49 mg, 45% combined yield). (*Z*:*E* = 91:9) *Major (Z)*: ^{1}H NMR (300 MHz, $CDCl_3$) δ: 7.61 (d,

J = 7 Hz, 2H), 7.39 – 7.24 (m, 3H), 6.47 (s, 1H), 2.47 (d, J = 4 Hz, 2H), 2.40 – 2.25 (m, 1H), 1.88 – 1.78 (m, 2H), 1.69 – 1.55 (m, 4H), 1.29 – 1.17 (m, 2H). ^{13}C NMR (75 MHz, $CDCl_3$) δ: 135.4, 135.0, 129.1 (2C), 128.3 (2C), 127.5, 124.7, 47.5, 38.0, 32.1 (2C), 25.2 (2C). HRMS (FI) m/z calcd for $C_{14}H_{17}Cl$: 220.1019; found: 220.1011.

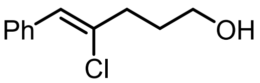

4-Chloro-5-phenylpent-4-en-1-ol (16a). Prepared according to **GP4**. Purified by flash column chromatography on silica gel (Pentane:Ethyl Acetate 80:20) to afford the product as a colourless liquid (52 mg, 53% combined yield). (*Z*:*E* = 92:8) *Major (Z)*: ^{1}H NMR (300 MHz, $CDCl_3$) δ: 7.60 (d, J = 8 Hz, 2H), 7.39 – 7.25 (m, 3H), 6.53 (s, 1H), 3.73 (t, J = 6 Hz, 2H), 2.61 (t, J = 7 Hz, 2H), 1.98 – 1.89 (m, 2H), 1.24 (s, 1H). ^{13}C NMR (75 MHz, $CDCl_3$) δ: 135.1, 134.2, 129.1 (2C), 128.3 (2C), 127.7, 125.0, 61.6, 37.6, 30.6. HRMS (FI) m/z calcd for $C_{11}H_{13}ClO$: 196.0655; found: 196.0656.

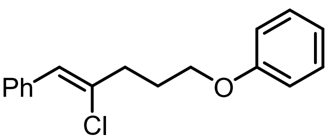

(2-Chloro-5-phenoxypent-1-en-1-yl)benzene (16b). Prepared according to **GP4**. Reaction time: 16 h. Purified by flash column chromatography on silica gel (Pentane:Ethyl Acetate 100:0 → 90:10) to afford the product as a colourless oil (83 mg, 61% combined yield). (*Z*:*E* = 90:10) *Major (Z)*: ^{1}H NMR (300 MHz, $CDCl_3$) δ: 7.60 (d, J = 7 Hz, 2H), 7.43 – 7.25 (m, 5H), 7.04 – 6.96 (m, 3H), 6.57 (s, 1H), 4.08 (t, J = 6 Hz, 2H), 2.77 (t, J = 7 Hz, 2H), 2.25 – 2.16 (m, 2H). ^{13}C NMR (75 MHz, $CDCl_3$) δ: 159.1, 135.1, 133.8, 129.6 (2C), 129.1 (2C), 128.3 (2C), 127.7, 125.3, 120.8, 114.6 (2C), 66.3, 37.8, 27.4. HRMS (FI) m/z calcd for $C_{17}H_{17}ClO$: 272.0968; found: 272.0963.

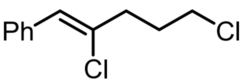

(2,5-Dichloropent-1-en-1-yl)benzene (16c). Prepared according to **GP4**. Purified by flash column chromatography on silica gel (Pentane) to afford the product as a colourless liquid (70 mg, 65% combined yield). (*Z*:*E* = 94:6) *Major (Z)*: ^{1}H NMR (300 MHz, $CDCl_3$) δ: 7.63 (d, J = 7 Hz, 2H), 7.41 – 7.27 (m, 3H), 6.58 (s, 1H), 3.63 (t, J = 6 Hz, 2H), 2.70 (t, J = 7 Hz, 2H), 2.20 – 2.11 (m, 2H). ^{13}C NMR (75 MHz, $CDCl_3$) δ: 134.9, 132.8, 129.1 (2C), 128.3 (2C), 127.8, 125.9, 43.7, 38.2, 30.2. HRMS (FI) m/z calcd for $C_{11}H_{12}Cl_2$: 214.0316; found: 214.0327.

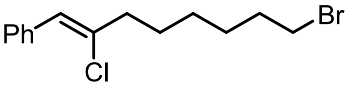

(2-Chloro-8-iodooct-1-en-1-yl)benzene (**17**). Prepared according to **GP4**. Purified by flash column chromatography on silica gel (Pentane) to afford the product as a colourless liquid (69 mg, 47% combined yield). (Z:E = 82:18) *Major (Z)*: ^{1}H NMR (300 MHz, $CDCl_3$) δ 7.67 – 7.54 (m, 2H), 7.42 – 7.22 (m, 3H), 6.47 (s, 1H), 3.42 (t, *J* = 7 Hz, 2H), 2.49 (t, *J* = 7 Hz, 2H), 1.88 (p, *J* = 7 Hz, 2H), 1.68 (p, *J* = 7 Hz, 2H), 1.52 – 1.26 (m, 4H). ^{13}C NMR (75 MHz, $CDCl_3$) δ: 135.3, 134.8, 129.1, 128.3, 127.6, 124.6, 41.1, 34.0, 32.8, 28.0, 27.8, 27.5. HRMS (EI) m/z calcd for $C_{14}H_{18}BrCl$: 302.0258; found: 302.0268.

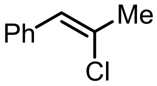

2-Chloro-1-phenyl-1-propene (18). Prepared according to **GP4** using 10 equiv. MeI. Purified by flash column chromatography on silica gel (Pentane) to afford the product as a mixture of diastereomers as a colourless liquid (12 mg, 16% combined yield). *Z/E* mixture: ^{1}H NMR (300 MHz, $CDCl_3$) δ 7.61 – 7.54 (m, 1.6H), 7.40 – 7.19 (m, 3.4H), 6.72 (s, 0.18H, *minor*), 6.47 (s, 0.82H, *major*), 2.30 (d, *J* = 1 Hz, 2.5H, *major*), 2.29 (d, *J* = 1 Hz, 0.5H, *minor*).

Major (Z): ^{13}C NMR (75 MHz, $CDCl_3$) δ 135.4, 130.5, 129.0 (2C), 128.3 (2C), 127.5, 124.9, 28.2.

Minor (Z): ^{13}C NMR (75 MHz, $CDCl_3$) δ 136.1, 133.0, 128.6 (2C), 128.6 (2C), 128.2, 127.3, 22.6.

The spectra are in accordance with the literature.[13]

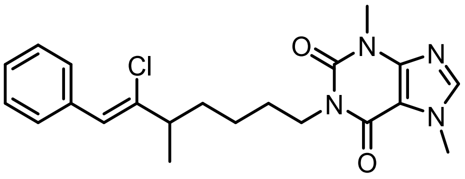

(Z)-1-(6-Chloro-5-methyl-7-phenylhept-6-en-1-yl)-3,7-dimethyl-3,7-dihydro-1H-purine-2,6-dione (19). Prepared according to **GP3**, 0.5 mmol **1q**, 2 equiv. **2a**. Purified by flash column chromatography on silica gel (Ethyl Acetate:dichloromethane 67:33) to afford the product as a light yellow liquid (79 mg, 39% combined yield). (*Z*:*E* = 94:6) *Major (Z)*: ^{1}H NMR (300 MHz, $CDCl_3$) δ 7.60 – 7.50 (m, 2H), 7.45 (s, 1H), 7.36 – 7.25 (m, 2H), 7.27 – 7.15 (m, 1H), 6.47 (s, 1H), 4.04 – 3.95 (m, 2H), 3.92 (s, 3H), 3.53 (s, 3H), 2.56 (ddt, *J* = 12, 9, 7 Hz, 1H), 1.76 – 1.58 (m, 3H), 1.51 – 1.28 (m, 3H), 1.17 (d, *J* = 7 Hz, 3H). ^{13}C NMR (75 MHz, $CDCl_3$) δ 155.3, 151.5, 148.8, 141.4, 139.8, 135.3, 129.2 (2C), 128.1 (2C), 127.4, 123.8, 107.7, 44.2, 41.3, 34.2,

33.6, 29.7, 27.9, 24.7, 19.6. HRMS (FD) m/z calcd for $C_{21}H_{25}ClN_4O_2$: 400.1666; found: 400.1674.

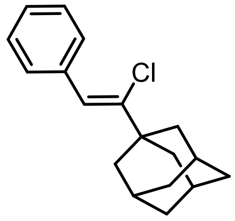

(3r,5r,7r)-1-((Z)-1-Chloro-2-phenylvinyl)adamantane (20). Prepared according to **GP3**, **1r** (0.5 mmol), **2a** (1.2 equiv.). Purified by flash column chromatography on silica gel (100% Pentane) to afford the product as a white solid (42 mg, 31% major). (*Z*:*E* = 75:25) *Major (Z)*: ^{1}H NMR (300 MHz, $CDCl_3$) δ 7.63 – 7.53 (m, 2H), 7.41 – 7.31 (m, 2H), 7.30 – 7.22 (m, 1H), 6.49 (s, 1H), 2.15 – 2.07 (m, 3H), 1.97 – 1.91 (m, 6H), 1.83 – 1.68 (m, 6H). ^{13}C NMR (75 MHz, $CDCl_3$) δ 145.8, 136.0, 129.5 (2C), 128.1 (2C), 127.3, 121.3, 41.1 (3C), 40.9, 36.8 (3C), 28.6 (3C). HRMS (FD) m/z calcd for $C_{18}H_{21}Cl$: 272.1332; found: 272.1332. A pure fraction of the minor diastereomer could not be isolated due to co-elution with remaining organic halide.

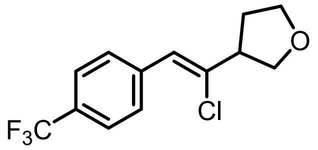

3-(1-Chloro-2-(4-(trifluoromethyl)phenyl)vinyl)tetrahydrofuran (21). Prepared according to **GP3**. Purified by flash column chromatography on silica gel (Pentane:Ethyl Acetate 100:0 → 90:10) to afford the product as a colourless liquid (105 mg, 76% combined yield). (*Z*:*E* = 90:10) *Major (Z)*: ^{1}H NMR (300 MHz, $CDCl_3$) δ: 7.69 – 7.58 (m, 4H), 6.62 (s, 1H), 4.06 – 3.82 (m, 4H), 3.39 – 3.28 (m, 1H), 2.19 (q, *J* = 7 Hz, 2H). ^{13}C NMR (75 MHz, $CDCl_3$) δ: 138.5 (d, *J* = 2 Hz), 137.1, 129.6 (q, *J* = 33 Hz, 2C), 129.5, 125.2 (q, *J* = 4 Hz, 2C), 124.2 (q, *J* = 272 Hz), 123.9, 71.4, 68.6, 49.3, 31.4. ^{19}F NMR (282 MHz, $CDCl_3$) δ: -62.6 (s, 3F). HRMS (FI) m/z calcd for $C_{13}H_{12}ClF_3O$: 276.0529; found: 276.0534.

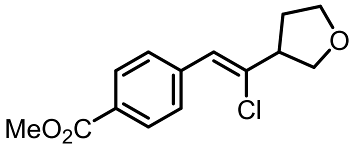

Methyl (Z)-4-(2-chloro-2-(tetrahydrofuran-3-yl)vinyl)benzoate (22). Prepared according to **GP3**. Purified by flash column chromatography on silica gel (Pentane:Ethyl Acetate 100:0 → 90:10) to afford the product as a colourless liquid (72 mg, 54% combined yield). (*Z*:*E* = 80:20) *Major (Z)*: ^{1}H NMR (300 MHz, $CDCl_3$) δ 8.07 – 7.96 (m, 2H), 7.70 – 7.60 (m, 2H), 6.63 (s, 1H), 4.01 (tt, J_1 = 9 Hz, J_2 = 7 Hz, 2H), 3.92 (s, 3H), 3.90 – 3.78 (m, 2H), 3.34 (p, *J* = 8 Hz,

1H), 2.26 – 2.12 (m, 2H). ^{13}C NMR (75 MHz, $CDCl_3$) δ 166.9, 139.3, 136.9, 129.6 (2C), 129.3, 129.2 (2C), 124.4, 71.4, 68.7, 52.3, 49.4, 31.5. HRMS (EI) m/z calcd for $C_{14}H_{15}ClO_3$: 266.0710; found: 266.0714.

Minor (E): ^{1}H NMR (400 MHz, $CDCl_3$) δ 8.03 (d, J = 8 Hz, 2H), 7.25 (d, J = 8 Hz, 2H), 6.81 (s, 1H), 4.01 – 3.89 (m, 5H), 3.87 – 3.77 (m, 2H), 3.65 (p, J = 8 Hz, 1H), 2.28 – 2.14 (m, 1H), 2.11 – 1.96 (m, 1H). ^{13}C NMR (101 MHz, $CDCl_3$) δ 166.7, 140.1, 139.6, 130.0 (2C), 129.3, 129.2, 128.5 (2C), 71.2, 68.9, 52.4, 41.9, 31.6. HRMS (EI) m/z calcd for $C_{14}H_{15}ClO_3$: 266.0710; found: 266.0700.

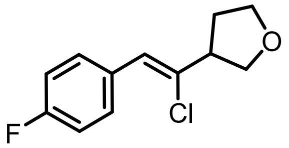

3-(1-Chloro-2-(4-fluorophenyl)vinyl)tetrahydrofuran (23). Prepared according to **GP3**. Purified by flash column chromatography on silica gel (Pentane:Ethyl Acetate 100:0 → 90:10) to afford the product as a greenish clear liquid (96 mg, 85% combined yield). (*Z*:*E* >95:5)

Major (Z): ^{1}H NMR (300 MHz, $CDCl_3$) δ: 7.57 (dd, J_1 = 9 Hz, J_2 = 6 Hz, 2H), 7.03 (t, J = 9 Hz, 2H), 6.54 (s, 1H), 4.04 – 3.80 (m, 4H), 3.35 – 3.25 (m, 1H), 2.16 (q, J = 7 Hz, 2H). ^{13}C NMR (75 MHz, $CDCl_3$) δ: 162.1 (d, J = 248 Hz), 134.3 (d, J = 2 Hz), 131.0 (d, J = 8 Hz, 2C), 130.8 (d, J = 3 Hz), 124.0, 115.2 (d, J = 21 Hz, 2C), 71.3, 68.6, 49.1, 31.4. ^{19}F NMR (282 MHz, $CDCl_3$) δ: -113.2 (tt, J_1 = 9 Hz, J_2 = 5 Hz, 1F). HRMS (FI) m/z calcd for $C_{12}H_{12}ClFO$: 226.0561; found: 226.0555.

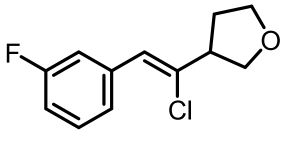

3-(1-Chloro-2-(3-fluorophenyl)vinyl)tetrahydrofuran (24). Prepared according to **GP3**. Purified by flash column chromatography on silica gel (Pentane:Ethyl Acetate 95:5) to afford the product as a colorless oil (85 mg, 75% combined yield). (*Z*:*E* = 93:7)

Major (Z): ^{1}H NMR (300 MHz, $CDCl_3$) δ 7.44 – 7.35 (m, 1H), 7.35 – 7.27 (m, 2H), 7.05 – 6.89 (m, 1H), 6.55 (s, 1H), 4.22 – 3.92 (m, 2H), 3.92 – 3.78 (m, 2H), 3.31 (p, J = 8 Hz, 1H), 2.17 (dt, J_1 = 8 Hz, J_2 = 7 Hz, 2H). ^{13}C NMR (75 MHz, $CDCl_3$) δ 162.6 (d, J = 245 Hz), 136.8 (d, J = 8 Hz), 135.9, 129.7 (d, J = 8 Hz), 125.1 (d, J = 3 Hz), 124.0 (d, J = 3 Hz), 115.8 (d, J = 23 Hz), 114.8 (d, J = 21 Hz), 71.3, 68.6, 49.2, 31.4. HRMS (EI) m/z calcd for $C_{12}H_{12}ClFO$: 226.0561; found: 226.0562.

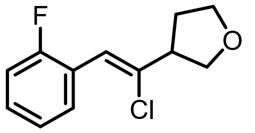

3-(1-Chloro-2-(2-fluorophenyl)vinyl)tetrahydrofuran (25). Prepared according to **GP3**. Purified by flash column chromatography on silica gel (Pentane:Ethyl Acetate 100:0 → 90:10) to afford the product as a colourless liquid (95 mg, 84% combined yield). (*Z*:*E* = 92:8) *Major (Z)*: ^{1}H NMR (400 MHz, $CDCl_3$) δ: 7.81 (td, J_1 = 8 Hz, J_2 = 1 Hz, 1H), 7.24 – 7.19 (m, 1H), 7.09 (t, J = 8 Hz, 1H), 7.00 (t, J = 9 Hz, 1H), 6.66 (s, 1H), 4.01 – 3.70 (m, 4H), 3.35 – 3.27 (m, 1H), 2.13 (q, J = 7 Hz, 2H). ^{13}C NMR (101 MHz, $CDCl_3$) δ: 160.2 (d, J = 249 Hz), 137.1 (d, J = 2 Hz) 130.2 (d, J = 3 Hz), 129.5 (d, J = 8 Hz), 123.7 (d, J = 4 Hz), 122.7 (d, J = 13 Hz), 117.6 (d, J = 5 Hz), 115.3 (d, J = 22 Hz), 71.3, 68.6, 49.1, 31.4. ^{19}F NMR (376 MHz, $CDCl_3$) δ: -115.20 – -115.3 (m, 1F). HRMS (FI) m/z calcd for $C_{12}H_{12}ClFO$: 226.0561; found: 226.0572.

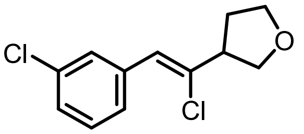

3-(1-Chloro-2-(3-chlorophenyl)vinyl)tetrahydrofuran (26). Prepared according to **GP3**. Purified by flash column chromatography on silica gel (Pentane:Ethyl Acetate 98:2 → 90:10) to afford the product as a colourless liquid (75 mg, 62% combined yield). (*Z*:*E* = 95:5) *Major (Z):* ^{1}H NMR (300 MHz, $CDCl_3$) δ 7.63 – 7.56 (m, 1H), 7.51 – 7.37 (m, 1H), 7.33 – 7.17 (m, 2H), 6.52 (s, 1H), 3.99 (dq, J_1 = 8 Hz, J_2 = 7 Hz, 2H), 3.92 – 3.79 (m, 2H), 3.30 (p, J = 8 Hz, 1H), 2.25 – 2.10 (m, 2H). ^{13}C NMR (75 MHz, $CDCl_3$) δ 136., 136.1, 134.1, 129.5, 129.1, 127.8, 127.4, 123.8, 71.3, 68.6, 49.2, 31.4. HRMS (EI) m/z calcd for $C_{12}H_{12}Cl_2O$: 242.0265; found: 242.0267.

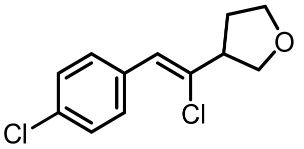

3-(1-Chloro-2-(4-chlorophenyl)vinyl)tetrahydrofuran (27). Prepared according to **GP3**. Purified by flash column chromatography on silica gel (Pentane:Ethyl Acetate 95:5 → 90:10) to afford the product as a colourless liquid (81 mg, 67% combined yield). (*Z*:*E* = 91:9) *Major (Z)*: ^{1}H NMR (300 MHz, $CDCl_3$) δ 7.52 (d, J = 9 Hz, 2H), 7.31 (d, J = 9 Hz, 2H), 6.53 (s, 1H), 3.99 (dt, J_1 = 14 Hz, J_2 = 8 Hz, 2H), 3.92 – 3.78 (m, 2H), 3.30 (p, J = 8 Hz, 1H), 2.17 (dt, J_1 = 8 Hz, J_2 = 7 Hz, 2H). ^{13}C NMR (75 MHz, $CDCl_3$) δ 135.3, 133.5, 133.2, 130.5 (2C), 128.5 (2C), 124.0, 71.3, 68.6, 49.2, 31.4. HRMS (EI) m/z calcd for $C_{12}H_{12}Cl_2O$: 242.0265; found: 242.0273.

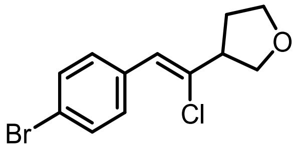

3-(2-(4-Bromophenyl)-1-chlorovinyl)tetrahydrofuran (28). Prepared according to **GP3**. Purified by flash column chromatography on silica gel (Pentane:Ethyl Acetate 95:5 → 90:10) to afford the product as a colourless liquid (116 mg, 81% combined yield). (*Z*:*E* = 86:14)

Major (Z): ^{1}H NMR (300 MHz, $CDCl_3$) δ 7.45 (s, 4H), 6.50 (s, 1H), 4.09 – 3.92 (m, 2H), 3.91 – 3.78 (m, 2H), 3.29 (p, J = 8 Hz, 1H), 2.16 (dt, J_1 = 8 Hz, J_2 = 7 Hz, 2H). ^{13}C NMR (75 MHz, $CDCl_3$) δ 135.4, 133.6, 131.4 (2C), 130.7 (2C), 124.0, 121.7, 71.3, 68.5, 49.2, 31.3. HRMS (EI) m/z calcd for $C_{12}H_{12}BrClO$: 285.9760; found: 285.9749.

Minor (E): ^{1}H NMR (300 MHz, $CDCl_3$) δ 7.49 (d, J = 8 Hz, 2H), 7.19 – 6.98 (m, 2H), 6.71 (s, 1H), 4.01 – 3.86 (m, 2H), 3.86 – 3.75 (m, 2H), 3.69 – 3.54 (m, 1H), 2.29 – 2.11 (m, 1H), 2.11 – 1.96 (m, 1H). ^{13}C NMR (75 MHz, $CDCl_3$) δ 138.5, 134.4, 131.9 (2C), 130.1 (2C), 128.9, 121.7, 71.08, 68.9, 41.8, 31.5. HRMS (EI) m/z calcd for $C_{12}H_{12}BrClO$: 285.9760; found: 285.9748.

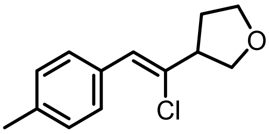

3-(1-Chloro-2-(4-methoxyphenyl)vinyl)tetrahydrofuran (29). Prepared according to **GP3**. Purified by flash column chromatography on silica gel (Pentane:Ethyl Acetate 95:5) to afford the product as a colourless liquid (87 mg, 78% combined yield). (*Z*:*E* >95:5) *Major (Z)*: ^{1}H NMR (300 MHz, $CDCl_3$) δ: 7.51 (d, J = 8 Hz, 2H), 7.17 (d, J = 8 Hz, 2H), 6.56 (s, 1H), 4.06 – 3.81 (m, 4H), 3.36 – 3.26 (m, 1H), 2.36 (s, 3H), 2.22 – 2.14 (m, 2H). ^{13}C NMR (75 MHz, $CDCl_3$) δ: 137.8, 133.6, 131.9, 129.1 (2C), 129.0 (2C), 125.0, 71.4, 68.6, 49.2, 31.4, 21.4. HRMS (FI) m/z calcd for $C_{13}H_{15}ClO$: 222.0811; found: 222.0819.

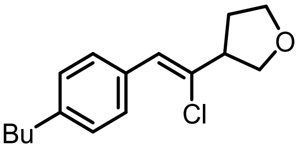

3-(2-(4-Butylphenyl)-1-chlorovinyl)tetrahydrofuran (30). Prepared according to **GP3**. Purified by flash column chromatography on silica gel (Pentane:Ethyl Acetate 95:5) to afford the product as a colourless liquid (110 mg, 83% combined yield). (*Z*:*E* >95:5) *Major (Z):* ^{1}H NMR (300 MHz, $CDCl_3$) δ 7.55 (d, J = 8 Hz, 2H), 7.20 (d, J = 8 Hz, 2H), 6.59 (s, 1H), 4.11 – 3.95 (m, 2H), 3.95 – 3.82 (m, 2H), 3.34 (p, J = 8 Hz, 1H), 2.64 (t, J = 8 Hz, 2H), 2.29 – 2.11 (m, 2H), 1.70 – 1.56 (m, 2H), 1.49 – 1.32 (m, 2H), 0.96 (t, J = 7 Hz, 3H). ^{13}C NMR (75 MHz,

$CDCl_3$) δ 142.8, 133.5, 132.1, 129.2 (2C), 128.4 (2C), 125.1, 71.4, 68.6, 49.2, 35.5, 33.6, 31.4, 22.5, 14.1. HRMS (EI) m/z calcd for $C_{16}H_{21}ClO$: 264.1281; found: 264.1272.

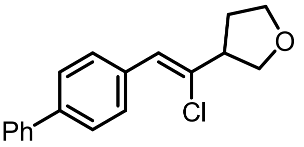

3-(2-([1,1'-Biphenyl]-4-yl)-1-chlorovinyl)tetrahydrofuran (31). Prepared according to **GP3**. Purified by flash column chromatography on silica gel (Pentane:Ethyl Acetate 98:2) to afford the product as a colourless liquid (110 mg, 83% combined yield). (*Z*:*E* = >95:5)

Major (Z): ^{1}H NMR (300 MHz, $CDCl_3$) δ 7.71 (d, *J* = 8 Hz, 2H), 7.67 – 7.56 (m, 4H), 7.47 (t, *J* = 8 Hz, 2H), 7.37 (t, *J* = 7 Hz, 1H), 6.64 (s, 1H), 4.14 – 3.96 (m, 2H), 3.96 – 3.80 (m, 2H), 3.35 (p, *J* = 8 Hz, 1H), 2.30 – 2.12 (m, 2H). ^{13}C NMR (75 MHz, $CDCl_3$) δ 140.6, 140.5, 134.6, 133.8, 129.7 (2C), 128.9 (2C), 127.5, 127.1 (2C), 126.9 (2C), 124.7, 71.4, 68.6, 49.3, 31.4. HRMS (EI) m/z calcd for $C_{18}H_{17}ClO$: 284.0968; found: 264.1272.

Minor (E): ^{1}H NMR (300 MHz, $CDCl_3$) δ 7.60 (d, *J* = 8 Hz, 4H), 7.46 (t, *J* = 7 Hz, 2H), 7.37 (t, *J* = 7 Hz, 1H), 7.27 (d, *J* = 8 Hz, 2H), 6.84 (s, 1H), 4.07 – 3.92 (m, 2H), 3.88 (s, 3H), 2.38 – 2.15 (m, 1H), 2.15 – 1.98 (m, 1H). ^{13}C NMR (75 MHz, $CDCl_3$) δ 140.5, 140.5, 137.8, 134.5, 129.7, 129.0 (2C), 128.9 (2C), 127.7, 127.4 (2C), 127.1 (2C), 71.2, 68.9, 41.9, 31.6. HRMS (EI) m/z calcd for $C_{18}H_{17}ClO$: 284.0968; found: 284.0964.

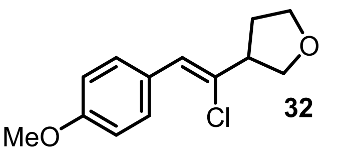

32

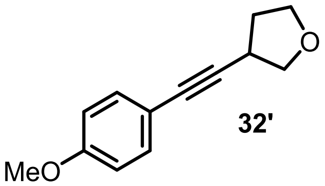

32'

3-(1-Chloro-2-(p-tolyl)tetrahydrofuran (32). Prepared according to **GP3**. Purified by flash column chromatography on silica gel (Cyclohexane:Ethyl Acetate 100:0 → 90:10) to afford the product as a colourless oil (50 mg, 42% combined yield) (*Z*:*E* = 89:11) along with the alkyne **32′** (34 mg, 34%). *Major (Z)*: ^{1}H NMR (300 MHz, $CDCl_3$) δ: 7.59 – 7.55 (m, 2H), 6.91 – 6.86 (m, 2H), 6.52 (s, 1H), 4.04 – 3.80 (m, 7H), 3.82 (s, 3H), 3.35 – 3.24 (m, 1H), 2.20 – 2.13 (q, *J* = 8 Hz, 2H). ^{13}C NMR (75 MHz, $CDCl_3$) δ: 159.2, 132.4, 130.6 (2C), 127.4, 124.6, 113.7 (2C), 71.4, 68.6, 55.4, 49.2, 31.4. HRMS (FI) m/z calcd for $C_{13}H_{15}ClO_2$: 238.0761; found: 238.0755. Characterization for **32′** is in accordance with the literature.[14] **3-((4-Methoxyphenyl)ethynyl)tetrahydrofuran (32')**. ^{1}H NMR (300 MHz, $CDCl_3$) δ = 7.37 – 7.28 (m, 2H), 6.85 – 6.77 (m, 2H), 4.07 (t, *J* = 8 Hz, 1H), 4.00 – 3.91 (m, 1H), 3.91 – 3.84 (m, 1H), 3.80 (s, 3H), 3.71 (t, *J* = 8 Hz, 1H), 3.26 – 3.10 (m, 1H), 2.34 – 2.21 (m, 1H), 2.11 – 1.99 (m, 1H).

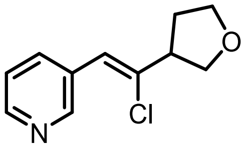

3-(2-Chloro-2-(tetrahydrofuran-3-yl)vinyl)pyridine (33). Prepared according to **GP3**. Purified by flash column chromatography on silica gel (Cyclohexane:Ethyl Acetate 50:50 → 10:90) to afford the product as a colourless oil (33 mg, 31% combined yield). (*Z*:*E* = 91:9) *Major (Z)*: ^{1}H NMR (300 MHz, $CDCl_3$) δ 8.67 (d, J = 2 Hz, 1H), 8.48 (dd, J_1 = 5 Hz, J_2 = 2 Hz, 1H), 8.08 – 7.98 (m, 1H), 7.34 – 7.21 (m, 1H), 6.55 (s, 1H), 4.08 – 3.92 (m, 2H), 3.92 – 3.79 (m, 2H), 3.34 (p, J = 8 Hz, 1H), 2.27 – 2.09 (m, 2H). ^{13}C NMR (75 MHz, $CDCl_3$) δ 150.6, 148.68, 137.4, 135.8, 130.8, 123.2, 121.8, 71.4, 68.6, 49.2, 31.4. HRMS (EI) m/z calcd for $C_{11}H_{12}ClNO$: 209.0607; found: 209.0600.

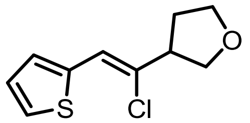

3-(1-Chloro-2-(thiophen-2-yl)vinyl)tetrahydrofuran (34). Prepared according to **GP3**. Purified by flash column chromatography on silica gel (Cyclohexane:Ethyl Acetate 90:10) to afford the product as a colourless oil (57 mg, 53% combined yield). (*Z*:*E* = 91:9) *Major (Z):* ^{1}H NMR (400 MHz, $CDCl_3$) δ 7.67 (d, J = 3 Hz, 1H), 7.38 (dd, J_1 = 5 Hz, J_2 = 1 Hz, 1H), 7.29 (dd, J_1 = 5 Hz, J_2 = 3 Hz, 1H), 6.61 (s, 1H), 3.98 (dt, J_1 = 15 Hz, J_2 = 8 Hz, 2H), 3.92 – 3.77 (m, 2H), 3.29 (p, J = 8 Hz, 1H), 2.16 (q, J = 7 Hz, 2H). ^{13}C NMR (101 MHz, $CDCl_3$) δ 135.7, 133.4, 128.7, 125.0, 124.9, 119.7, 71.4, 68.6, 48.9, 31.4. HRMS (EI) m/z calcd for $C_{10}H_{11}ClOS$: 214.0219; found: 214.0213.

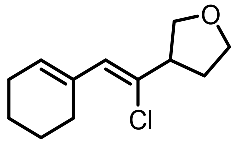

3-(1-Chloro-2-(cyclohex-1-en-1-yl)vinyl)tetrahydrofuran (35). Prepared according to **GP3**. Purified by flash column chromatography on silica gel (Cyclohexane:Ethyl Acetate 100:0 → 90:10) to afford the product as a colourless oil (34 mg, 32% combined yield). (*Z*:*E* = 80:20) *Major (Z)*: ^{1}H NMR (400 MHz, $CDCl_3$) δ 5.99 (s, 1H), 5.95 – 5.88 (m, 1H), 3.97 – 3.88 (m, 2H), 3.88 – 3.77 (m, 1H), 3.72 (t, J = 8 Hz, 1H), 3.14 (p, J = 7.9 Hz, 1H), 2.39 – 2.28 (m, 2H), 2.16 – 2.03 (m, 4H), 1.75 – 1.52 (m, 4H). ^{13}C NMR (101 MHz, $CDCl_3$) δ 133.6, 131.3, 130.2, 128.2, 71.4, 68.6, 49.2, 31.3, 28.5, 26.0, 22.9, 22.0. HRMS (EI) m/z calcd for $C_{12}H_{17}ClO$: 212.0968; found: 212.0965.

Minor (E): ^{1}H NMR (400 MHz, $CDCl_3$) δ 6.08 (s, 1H), 5.68 – 5.36 (m, 1H), 3.96 – 3.79 (m, 3H), 3.76 (s, 2H), 2.23 – 1.91 (m, 6H), 1.70 – 1.53 (m, 4H). ^{13}C NMR (101 MHz, $CDCl_3$) δ 134.5, 133.6, 132.9, 128.0, 71.3, 68.9, 42.1, 31.5, 29.0, 25.7, 22.7, 22.0. HRMS (EI) m/z calcd for $C_{12}H_{17}ClO$: 212.0968; found: 212.0978.

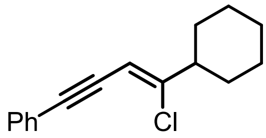

(4-Chloro-4-cyclohexylbuta-1,2,3-trien-1-yl)benzene (36). Prepared according to **GP3** on 100 micromole scale with irradiation for 18 h. Purified by preparative thin layer chromatography on silica gel (Heptane) to afford the product as a colourless oil (4.7 mg, 19% combined yield). (*Z*:*E* = 73:27) *Major (Z)*: ^{1}H NMR (300 MHz, $CDCl_3$) δ 7.53 – 7.42 (m, 2H), 7.34 – 7.27 (m, 3H), 5.83 (d, *J* = 1 Hz, 1H), 2.38 – 2.24 (m, 1H), 1.98 – 1.63 (m, 5H), 1.47 – 1.09 (m, 5H). ^{13}C NMR (75 MHz, $CDCl_3$) δ: 152.0, 131.7 (2C), 128.4 (3C), 123.4, 104.9, 94.6, 85.3, 47.4, 31.5, 26.1, 26.0. HRMS (EI) m/z calcd for $C_{16}H_{17}Cl$: 244.1019; found: 244.1017.

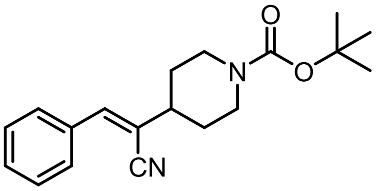

***tert*-Butyl (Z)-4-(1-cyano-2-phenylvinyl)piperidine-1-carboxylate (37).** Prepared by following a procedure reported in the literature,[15] compound **12** have been used on 0.1 mmol scale. Purified by flash column chromatography on silica gel (Cyclohexane:Ethyl Acetate 80:20) to afford the product as a colourless oil (16 mg, 50% yield). ^{1}H NMR (400 MHz, $CDCl_3$) δ 7.76 – 7.67 (m, 2H), 7.45 – 7.35 (m, 3H), 6.96 (s, 1H), 4.25 (s, 2H), 2.77 (d, *J* = 14 Hz, 2H), 2.45 (tt, J_1 = 12 Hz, J_2 = 4 Hz, 1H), 1.90 – 1.80 (m, 2H), 1.62 (td, J_1 = 13 Hz, J_2 = 4 Hz, 2H), 1.47 (s, 9H). ^{13}C NMR (101 MHz, $CDCl_3$) δ 154.7, 142.4, 133.6, 130.2, 128.9, 128.8, 118.0, 115.6, 79.9, 43.4, 42.7, 30.9, 28.6. HRMS (FD) m/z calcd for $C_{19}H_{24}N_2O_2$: 312.1838; found: 312.1839.

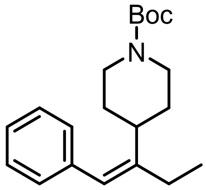

***tert*-Butyl (Z)-4-(1-phenylbut-1-en-2-yl)piperidine-1-carboxylate (38).** Prepared by following a procedure reported in the literature,[16] compound **12** have been used on 0.1 mmol

scale. Purified by flash column chromatography on silica gel (Cyclohexane:Ethyl Acetate 92:8) to afford the product as a colourless oil (14 mg, 44% yield). (*Z*:*E* = 80:20)

Z/*E* mixture: ^{1}H NMR (400 MHz, $CDCl_3$) δ 7.35 – 7.28 (m, 2H), 7.24 – 7.12 (m, 3H), 6.27 (s, 0.8H), 6.23 (s, 0.2H), 4.13 (s, 2H), 2.84 (tt, J_1 = 12 Hz, J_2 = 4 Hz, 0.8H), 2.77 – 2.50 (m, 2H), 2.27 (q, J = 8 Hz, 0.4H), 2.22 – 2.11 (m, 0.2H), 2.12 (qd, J = 7, 2 Hz, 1.6H), 1.80 – 1.42 (m, 13H), 1.13 (t, J = 7 Hz, 2.4H), 1.07 (t, J = 8 Hz, 0.6H).

Major (Z): ^{13}C NMR (101 MHz, $CDCl_3$) δ 155.0, 147.6, 138.7, 128.8 (2C), 128.3 (2C), 126.1, 124.1, 79.5, 44.2 (2C), 38.8, 30.5 (2C), 28.4 (3C), 24.5, 13.6.

Minor (E): ^{13}C NMR (101 MHz, $CDCl_3$) δ 155.0, 148.3, 138.6, 128.7 (2C), 128.2 (2C), 126.2, 123.8, 79.5, 44.2 (2C), 42.9, 31.9 (2C), 28.6 (3C), 23.5, 13.8.

HRMS (FD) m/z calcd for $C_{20}H_{29}N_1O_2$: 315.2198; found: 315.2205.

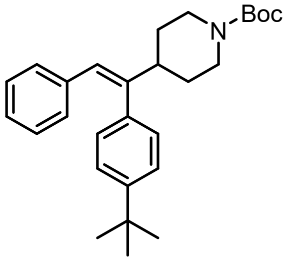

***tert*-Butyl (Z)-4-(1-(4-(tert-butyl)phenyl)-2-phenylvinyl)piperidine-1-carboxylate (39).** Prepared by following a procedure reported in the literature,[17] compound **12** have been used on 0.1 mmol scale. Purified by flash column chromatography on silica gel (Cyclohexane:Ethyl Acetate 95:5 → 80:20) to afford the product as a light yellow oil (38 mg, 91% yield). ^{1}H NMR (300 MHz, $CDCl_3$) δ 7.37 – 7.29 (m, 2H), 7.12 – 6.98 (m, 5H), 6.90 – 6.81 (m, 2H), 6.37 (s, 1H), 4.19 (d, J = 13 Hz, 2H), 2.71 (t, J = 13 Hz, 2H), 2.52 – 2.39 (m, 1H), 1.80 (dt, J = 13, 2 Hz, 2H), 1.46 (s, 11H), 1.34 (s, 9H). ^{13}C NMR (75 MHz, $CDCl_3$) δ 154.9, 150.0, 146.9, 137.8, 137.5, 129.2 (2C), 128.5 (2C), 127.9 (2C), 126.3, 125.5 (2C), 125.3, 79.4, 45.8, 44.4, 34.6, 31.5, 31.3, 28.6. HRMS (FD) m/z calcd for $C_{28}H_{37}NO_2$: 419.2824; found: 419.2814.

9. Mechanistic investigation

Quantum yield measurements

A fluorometer (HORIBA Fluorolog 3) was used for irradiation of the sample: its photon flux was determined by standard ferrioxalate actinometry.[18, 19]

A 0.15 M solution of ferrioxalate was prepared (in a dark room) by dissolving 736 mg of potassium ferrioxalate hydrate in 10 mL of 0.05 M H_2SO_4. A buffered solution of phenanthroline was prepared by dissolving 50 mg of phenanthroline and 11.25 g of NaOAc in 50 mL of 0.5 M H_2SO_4 (stored in the dark). Three vials were charged with 2 mL of the ferrioxalate solution, and each vial was transferred to a cuvette and irradiated for the indicated time (see *Table S 8*) at λ = 456 nm (emission slit width: 10 nm). After irradiation, 0.35 mL of the phenanthroline solution was added to the cuvette. The solution was then left equilibrating for 1 h to allow the ferrous ions to completely coordinate to the phenanthroline. The absorbance of the solutions was measured at 510 nm using a spectrophotometer.

Table S 8: Results obtained for ferrioxalate actinometry

**Entry**	**T_{irr} (s)**	**$A_{510\ nm}$ (corr)**	**mol Fe^{2+}**
Blank	0	0	0
1	10	0.50871	$1.1 \cdot 10^{-7}$
2	30	1.41614	$3.0 \cdot 10^{-7}$

mol Fe^{2+} was calculated using the following equation (eq. S1).

$$mol\,Fe^{2+} = \frac{V * \Delta A}{l * \varepsilon}$$, where

V: total volume (0.00235 L)

ΔA: difference in absorbance at 510 nm between irradiated and non-irradiated samples

l: optical path (1 cm)

ε: molar absorptivity at 510 nm (11100 M^{-1} cm^{-1})

Given that the number of moles of produced Fe^{2+} are directly proportional to the photon flux according the following equation:

$$mol\,Fe^{2+} = [\Phi * f * photon\,flux] * t$$

The average photon flux can be calculated by dividing the slope of the curve shown in **Figure S4** by the quantum yield (Φ) and fraction of light absorbed by ferrioxalate (*f*) at λ = 456 nm (0.93 and ≈1 for a 0.15 M solution at λ = 464 nm).[19] An average photon flux of $1.12 \cdot 10^{-6}$ E s^{-1} can be estimated.

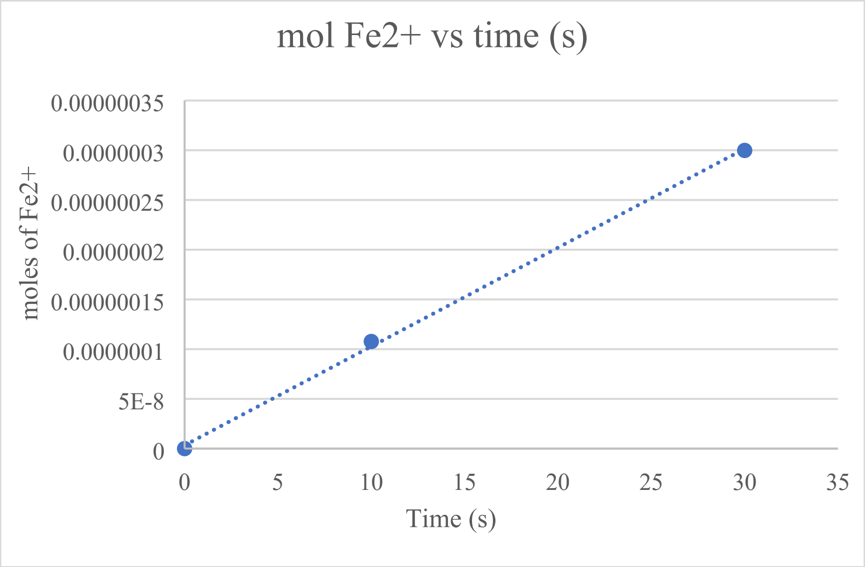

Figure S4: Plot of the moles of Fe^{2+} vs time (s)

Considering that the yield for compound **3** after 6h 7m 14s (22034 s) in the same setup is 12%, the quantum yield for the reaction is calculated to be ~10%. This value is in accordance with a process being supported by either short-lived radical chain propagations or an inefficient initiation process.[20]

Deuterium labelling experiments

The reactions for these experiments were performed following procedure **GP3**. A solution of **2a** (0.5 mmol), **1a** (2.0 equiv.), **B1** or **B1-*d*₃** (1.5 equiv.) and 4CzIPN (0.5 mol%) in CH_3CN/H_2O 9:1 or CD_3CN/D_2O 9:1 (0.1 M) was prepared in a 7 mL vial equipped with a screw cap and a stirring bar. The solution was bubbled with N_2 (for 5 min) and then irradiated by adopting the UFO reactor setup equipped with a 40 W Kessil lamp (λ = 456 nm, full intensity) for 6 h. The solution was then collected, solvent was removed under reduced pressure and the crude was purified via column chromatography on silica gel (Pure Pentane) to afford product **3**, **3-*d*₁** or a mixture of the two.

Characterization data for **3**:

^{1}H NMR (300 MHz, $CDCl_3$) δ: 7.61 (d, J = 7.4 Hz, 2H), 7.38 – 7.24 (m, 3H), 6.49 (s, 1H), 2.36 (tt, J_1 = 11 Hz, J_2 = 3 Hz, 1H), 1.99 – 1.71 (m, 5H), 1.55 – 1.15 (m, 5H). ^{13}C NMR (75 MHz, $CDCl_3$) δ: 140.7, 135.6, 129.3 (2C), 128.2 (2C), 127.4, 122.4, 48.9, 31.8 (2C), 26.3 (2C), 26.1.

Characterization data for **3-*d*₁**:

^{1}H NMR (300 MHz, $CDCl_3$) δ: 7.63 – 7.59 (m, 2H), 7.38 – 7.23 (m, 3H), 6.49 (s, 0.08H), 2.35 (tt, J_1 = 11 Hz, J_2 = 3 Hz, 1H), 1.99 – 1.71 (m, 5H), 1.55 – 1.15 (m, 5H). ^{13}C NMR (75 MHz, $CDCl_3$) δ: 140.6, 135.5, 129.3 (2C), 128.3 (2C), 127.4, 122.4, 48.9, 31.8 (2C), 26.3 (2C), 26.1.

Experiment with deuterated solvent

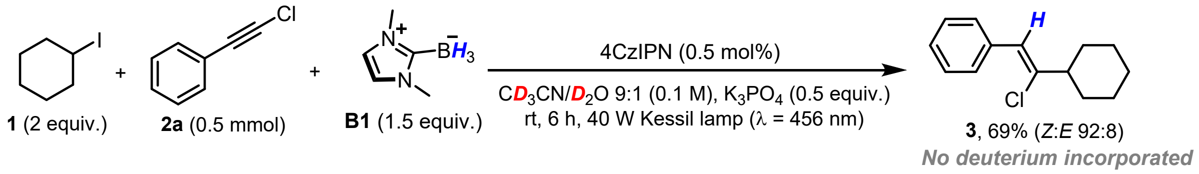

Product **3** was isolated in 69% yield (*Z*:*E* 92:8 determined from the crude via ^{1}H-NMR); no deuterium incorporation was observed, see ^{1}H-NMR spectra below.

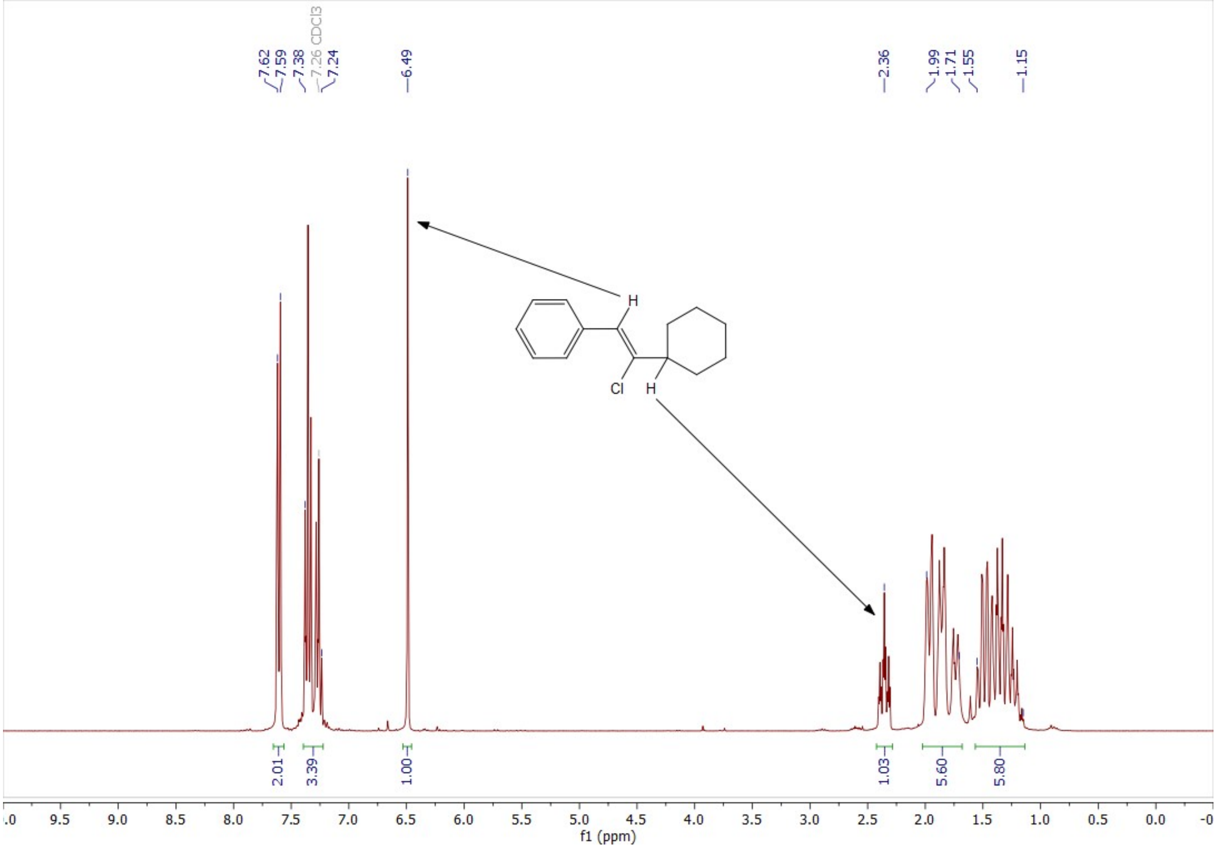

Experiment with deuterated ligated borane

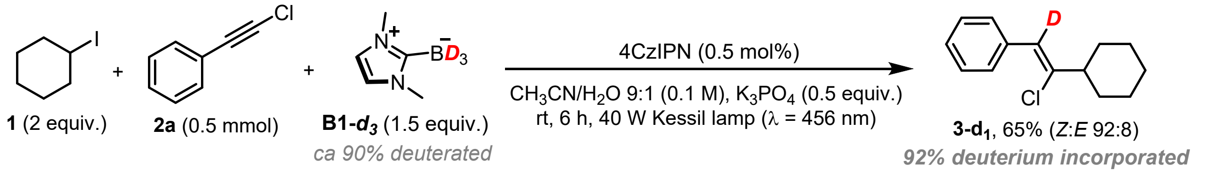

The product was isolated in 65% yield as a mixture of product **3** (5%) and **3-*d*$_1$** (60%); 92% of deuterium incorporation was calculated on the benzylic position, see ^{1}H-NMR spectra below.

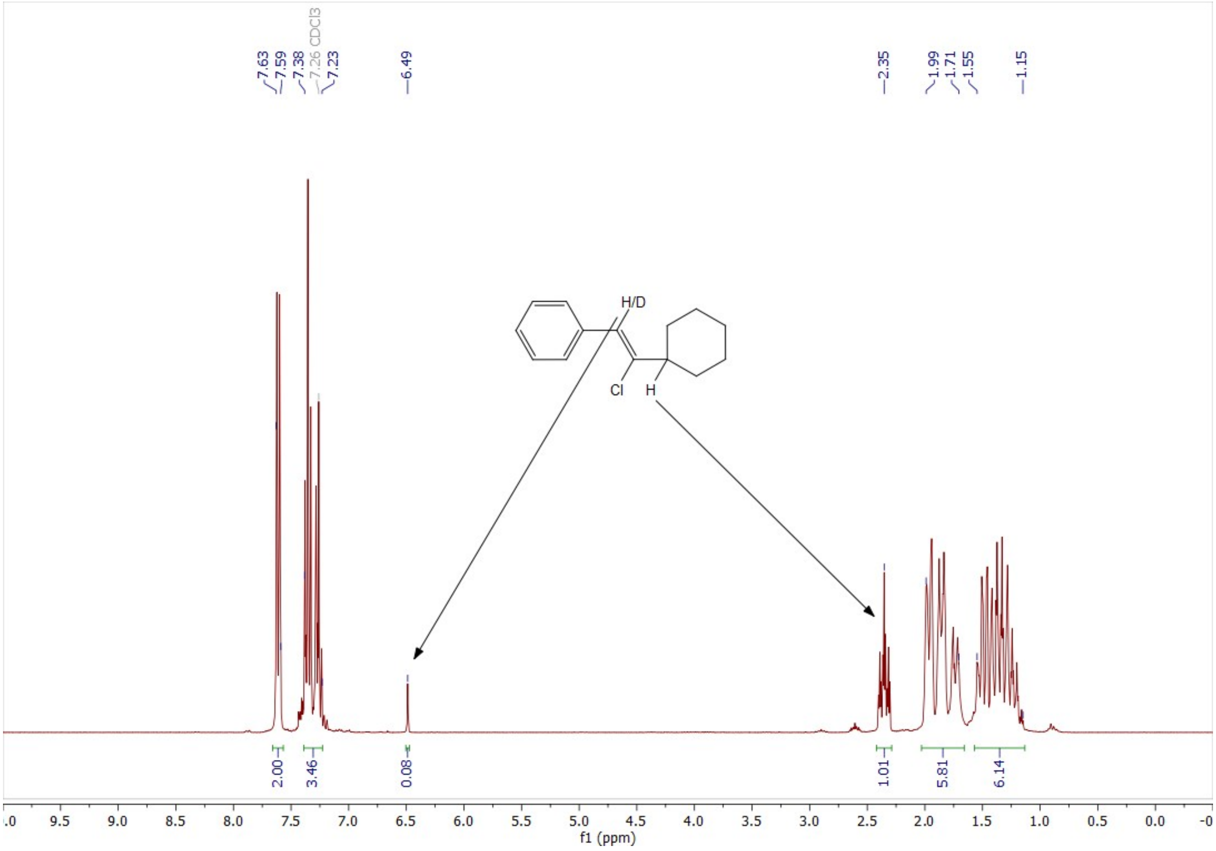

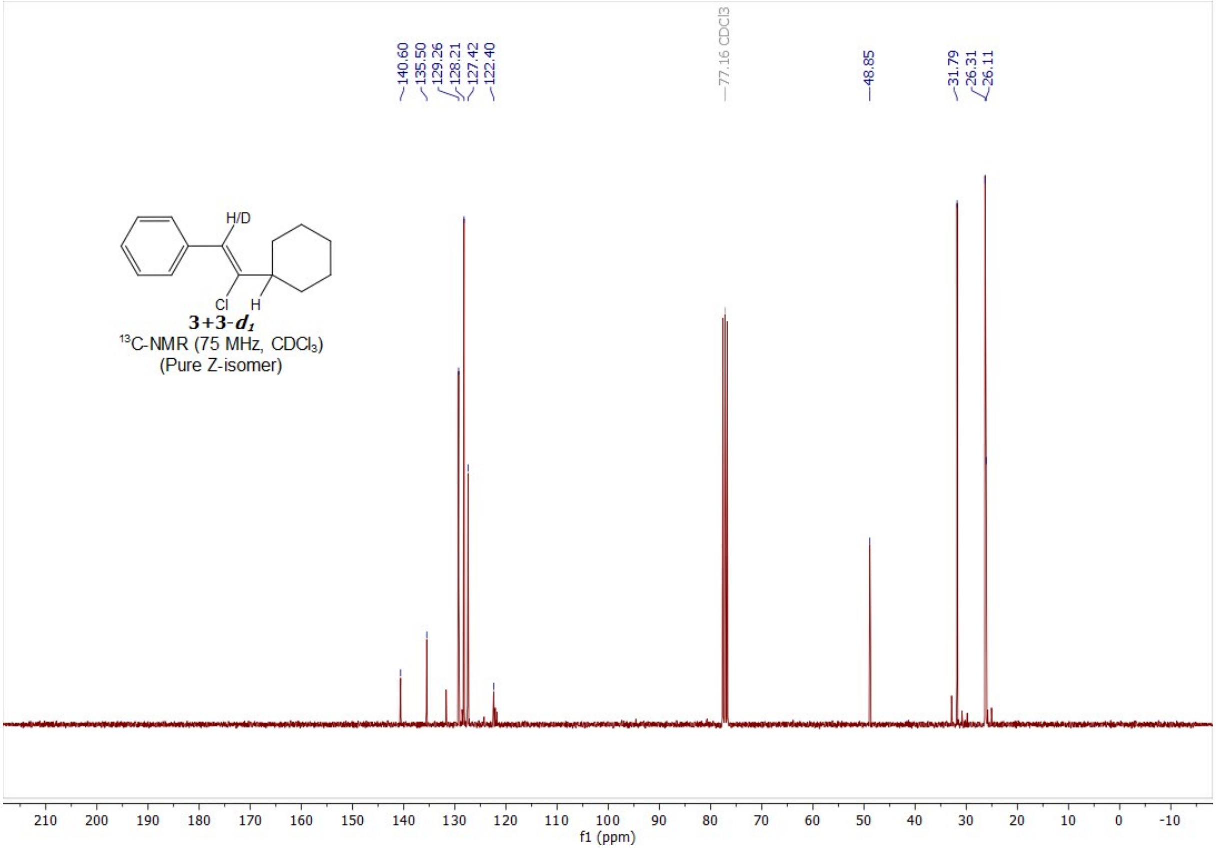

These experiments strongly suggest the occurrence of a radical chain mechanism as the major pathway of this reaction.

Additional control experiments

Determination of Kinetic Isotope Effect (KIE) via parallel reaction method.

A CH_3CN/H_2O 9:1 (0.1 M) stock solution containing the chloroalkyne **2a**, alkyl iodide **1a** (2.0 equiv.), **B1** (1.5 equiv.), K_3PO_4 (0.5 equiv) and 4CzIPN (0.5 mol%) was prepared in a 5 mL volumetric flask. The solution was then split in four 7 mL vials (1 mL each), the so obtained solutions were bubbled with N_2 (for 1 min each) and then irradiated by adopting the UFO reactor setup equipped with a 40 W Kessil lamp (λ = 456 nm, full intensity) for the indicated time. The same experiment was performed using **B1-d$_3$** (1.5 equiv.) in the place of **B1**. After irradiation, dibromomethane was added to the reaction crude and the sample was analyzed via ^{1}H-NMR (**Figure S5**).

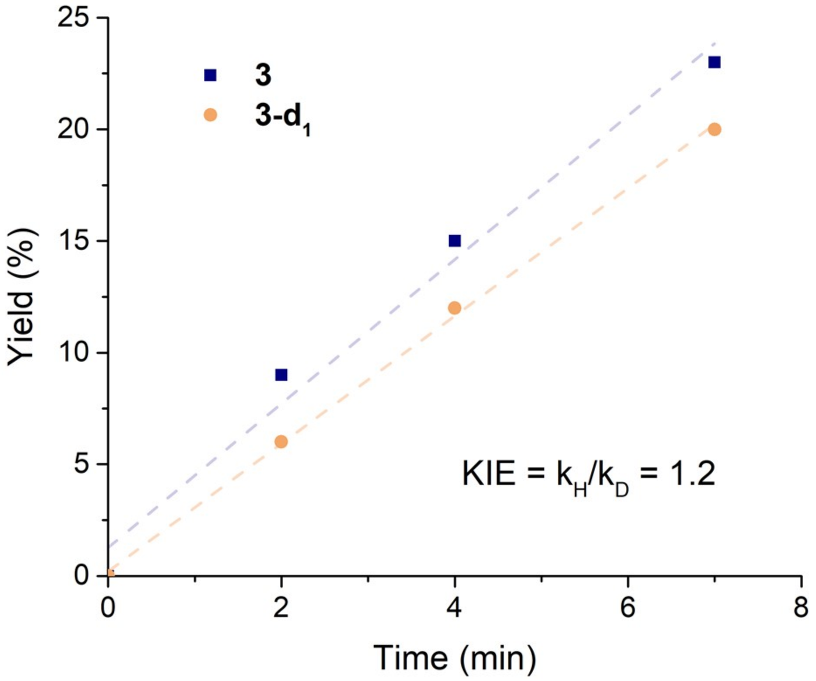

Figure S5. Parallel reactions method for KIE determination.

The KIE was determined to be 1.2. As this value is relatively low, this analysis suggests that the HAT might not be the rate-determining step (RDS). We identified the XAT step as the RDS based on our DFT analysis.

*Photoisomerization of vinyl chloride **3**.*

A CH_3CN/H_2O 9:1 (0.1 M) solution containing the chloroalkyne **2a** (0.5 mmol), alkyl iodide **1a** (2.0 equiv.), **B1** (1.5 equiv.), K_3PO_4 (0.5 equiv) and 4CzIPN (0.5 mol%) was prepared in a 7 mL vial equipped with a screw cap and a stirring bar. The solution was split in five 7 mL

vials and the obtained solutions were bubbled with N_2 (for 1 min each) and then irradiated by adopting the UFO reactor setup equipped with a 40 W Kessil lamp (λ = 456 nm, full intensity) for the indicated time. After irradiation, dibromomethane was added to the reaction crude and the sample was analyzed via ^{1}H-NMR (**Figure S6**).

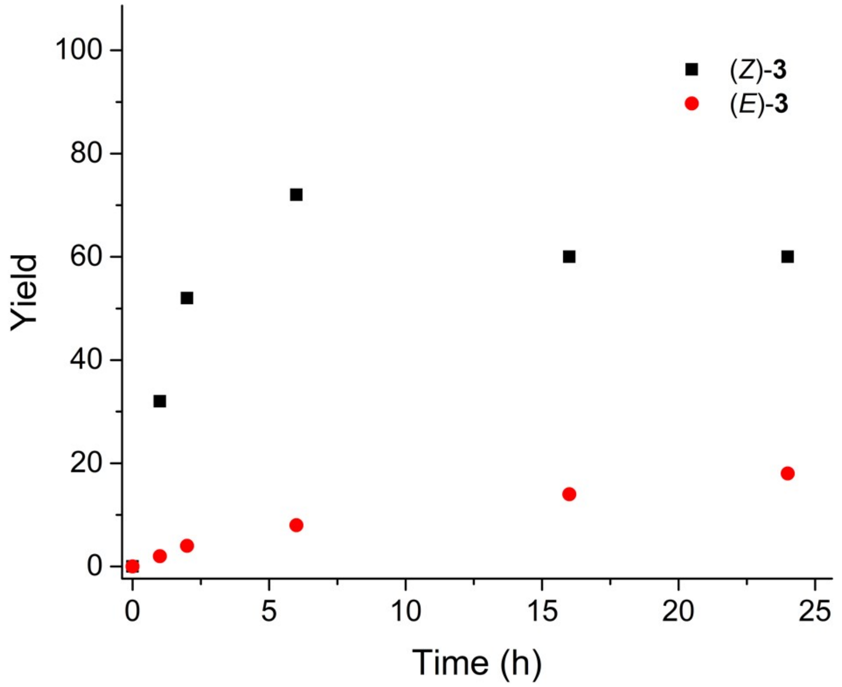

Figure S6. Photoisomerization of (*Z*)-**3** upon prolonged light exposition

The results obtained in **Figure S6** showed that the formation of vinyl chloride **3** is significantly faster than the *Z*→*E* photoisomerization via triplet-triplet energy transfer. In fact, a maximum yield of 80% was already reached after 6 h and remained constant upon prolonged reaction times. At the same time, it can be noted that the concentration of the *Z*-isomer reached a maximum value after 6 hours of irradiation (dr 90:10), after which it started declining slowly due to conversion to the *E*-isomer and less *Z*-isomer. For this reason, we elected 6 hours as our ideal reaction time, which proved a valid choice throughout the scope.

*Reaction profile of the reaction between **1h** and **2m**.*

A CH_3CN/H_2O 9:1 (0.1 M) stock solution containing the chloroalkyne **2m**, alkyl iodide **1h** (2.0 equiv.), **B1** (1.5 equiv.), K_3PO_4 (0.5 equiv) and 4CzIPN (0.5 mol%) was prepared in a 5 mL volumetric flask. The solution was then split in four 3 mL vials (1 mL each), the so-obtained solutions were bubbled with N_2 (for 1 min each) and then irradiated by adopting the UFO reactor setup equipped with a 40 W Kessil lamp (λ = 456 nm, full intensity) for the indicated time. After irradiation, dibromomethane was added to the reaction crude and the sample was

analyzed via ^{1}H-NMR and products **32** (vinyl chloride) and **32′** (alkyne) were quantified (**Figure S7**).

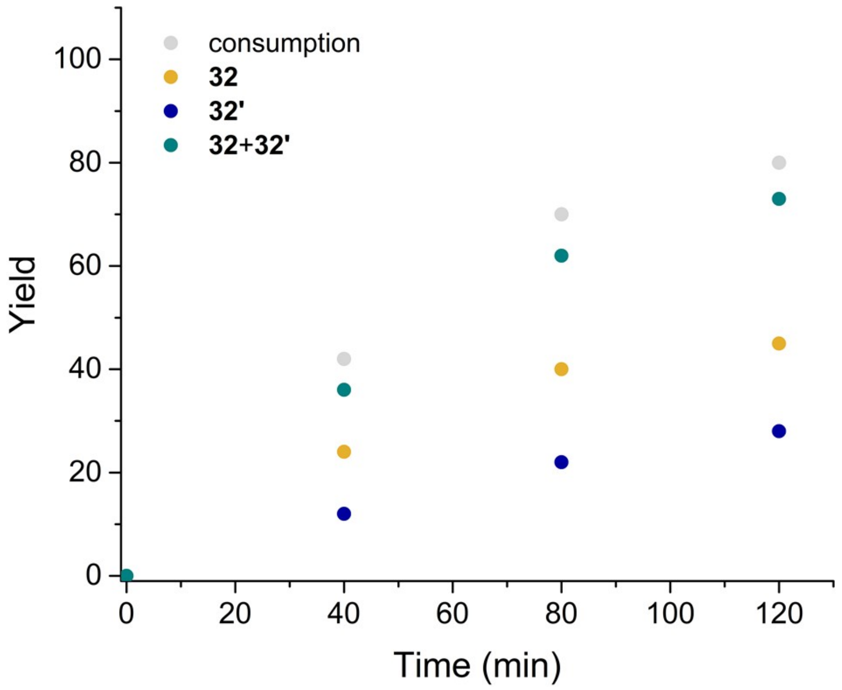

Figure S7. Kinetic profile of the reaction between **1h** and **2m**.

It can be immediately noted that the formation of **32** and **32′** are two parallel processes and not consecutive ones, showing overall a good mass balance. In order to get further evidence, we performed the following control experiments:

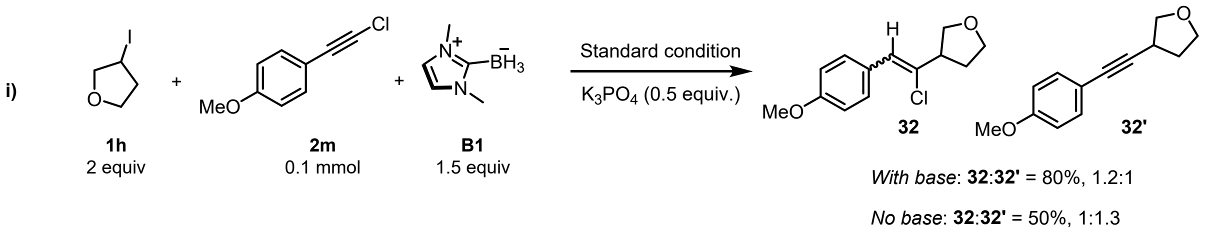

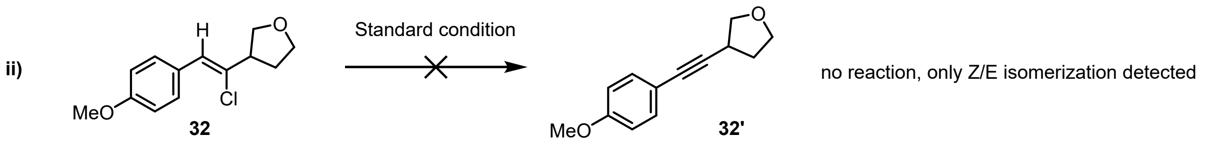

Taken together, these experiments show that **32′** is not formed from **32** because of the basic conditions. At present, we are still investigating a possible mechanism leading to **32′**.

Comparison with other XAT methodologies.

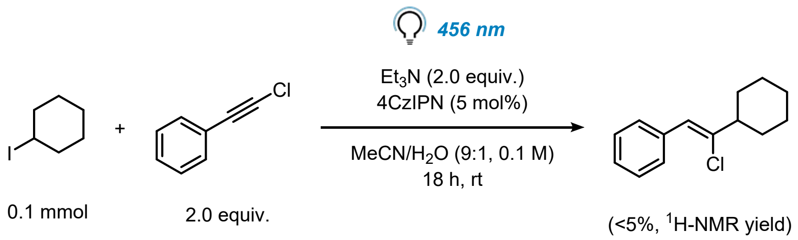

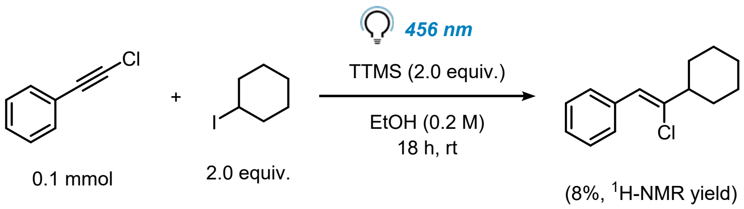

The reaction performed with other XAT methodologies did not lead to the desired product in satisfying yields. For example, only traces of the expected alkene were detected when using α-aminoalkyl radicals as XAT agents (<5%, ^{1}H-NMR yield). When employing a silyl radical-mediated radical chain scenario, only negligible product formation was observed (8%, 1H-NMR). We propose that the reduced nucleophilicity of boryl radicals makes these compounds uniquely suited to perform the title transformation.

10. Computational details

All the calculations were carried out using the Gaussian 16 program package, revision C.01 installed on the HPC infrastructure Dutch National Supercomputer Snellius (NL). In our investigation, the level of theory chosen for the optimization of the reported stationary points was DFT (Density Functional Theory) by using the ωB97xD functional and the def2TZVP basis set in the gas phase. When appropriate, an unrestricted formalism (U prefix) has been adopted via the UωB97xD/def2TZVP keyword. No symmetry constraint was applied to the structures investigated and a thorough conformers search has been performed to locate the absolute minimum for each species. The structures and data used for this work correspond to those of the absolute minimum. Frequency calculations were performed in the gas phase to check that minima and transition states (TS) had 0 or 1 imaginary frequencies, respectively.

Solvent effect was included by single-point calculations at the same level of theory (ωB97xD/def2TZVP) adopting the standard implicit solvent model implemented in Gaussian 16 via the keyword "scrf=solvent=acetonitrile" on the optimized geometries obtained in vacuo. The DFT Gibbs free energies reported in the main text (see Figure 3C) have been calculated by means of Eq. S1 reported below:

$$G_{DFT} = E_0(DFT,MeCN) + \Delta G_{CORR}(vacuo)$$

where E_0(DFT,MeCN) is the total electronic energy calculated at the SCRF-ωB97xD/def2TZVP level (acetonitrile bulk), while ΔG_{CORR}(vacuo) is the unscaled thermal correction to Gibbs Free Energy as from the output of the frequency calculation in vacuo, also including the zero-point vibrational energy (ZPVE).

When dealing with transition states (TSs), Intrinsic Reaction Coordinate (IRC) calculations were performed in both directions at the same level of theory adopted for optimizations (ωB97xD/def2TZVP in the gas phase) in order to confirm the nature of the TS itself. When indicated, the "LQA" option for the IRC keyword has been specified, in order to adopt the local quadratic approximation for the predictor step.

Optimized geometry listed in cartesian format (coordinates are given in Å), minimum energies and thermochemical data (in Hartree; the default options were adopted in the latter case, viz. temperature: 298.150 K and pressure: 1.00000 atm) are reported below.

The conversion factor adopted between Hartree and kcal·mol^{-1} is: 1 Hartree = 627.509 kcal·mol^{-1}.

Data for NHC-BH_3 (**B1**), the ligated boryl radical (NHC-$BH_2^{\bullet -}$, **I**) and NHC-BH_2I were taken from the literature.[21]

1. **XAT step**

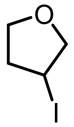

C	1.4702940	-1.1911510	-0.0670500
C	0.6817690	-0.0042780	0.4713170
C	1.4481960	1.1676550	-0.1215210
C	2.9023120	-0.6326150	-0.0869420
H	1.3692860	-2.0811590	0.5512300
H	1.1372630	-1.4371840	-1.0757540
H	0.7132880	0.0345570	1.5578680
H	1.1792470	1.3120090	-1.1762010
H	1.2945680	2.1037460	0.4144130
H	3.4262370	-0.8998690	-1.0084060
H	3.4930100	-0.9860680	0.7604920
O	2.7987320	0.7858430	0.0168730
I	-1.3965690	-0.0069900	-0.0251590

E(wB97XD, vacuo)	-529.651935
Zero-point correction	0.107755
Thermal correction to Energy	0.113957
Thermal correction to Enthalpy	0.114901
Thermal correction to Gibbs Free Energy	0.075054
E(wB97XD, MeCN)	-529.656547

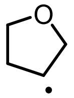

C	1.2474060	0.1124670	0.0670840
O	0.5115610	-1.0627120	-0.2236340
C	-0.8280110	-0.8429470	0.1765230
C	-1.1080520	0.6363900	-0.1106900
C	0.2524870	1.2172450	0.0359100
H	1.7343260	0.0281100	1.0543910
H	2.0438700	0.2108880	-0.6782960
H	-1.4608790	-1.5304680	-0.3831980
H	-0.9447270	-1.0501360	1.2490870
H	-1.4976120	0.7703210	-1.1288050
H	-1.8440980	1.0666110	0.5732130
H	0.4936570	2.2674380	0.0897190

E(UwB97XD, vacuo)	-231.799560
Zero-point correction	0.102522
Thermal correction to Energy	0.107579
Thermal correction to Enthalpy	0.108523
Thermal correction to Gibbs Free Energy	0.074129
E(UwB97XD, MeCN)	-231.802922

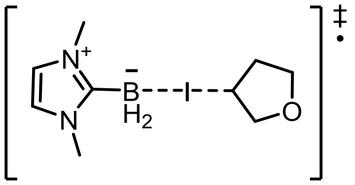

C	-2.9725930	0.0394330	1.5358560
C	-2.8445500	-0.5437550	0.1342810
C	-3.4709590	0.4933590	-0.7805990
C	-3.7738320	1.3242220	1.2943420
H	-3.4781870	-0.6413290	2.2221650
H	-1.9951020	0.2725800	1.9611170
H	-3.2150620	-1.5542300	0.0022610
H	-2.9217980	0.6728060	-1.7043340
H	-4.4979320	0.1878770	-1.0421210
H	-3.4847150	2.1514760	1.9418000
H	-4.8506330	1.1395850	1.4202260
O	-3.4984370	1.6953850	-0.0398280
I	-0.5257460	-0.8682510	-0.4421710
C	3.2321850	1.0503300	1.5092610
C	3.0416560	1.9402800	0.5130930
C	2.5413790	-0.0888960	-0.2908720
N	2.9328380	-0.1901250	1.0024890
H	3.5582180	1.1936080	2.5236460
H	3.1680880	3.0077130	0.4939140
N	2.6297070	1.2302860	-0.5871660
B	2.0071850	-1.2190610	-1.1973230
H	2.1949140	-2.3409260	-0.8298570
H	1.9194410	-0.9802780	-2.3650690
C	2.9192450	-1.4269690	1.7535620
H	1.9033150	-1.8208820	1.7929260
H	3.5639970	-2.1630450	1.2766790
H	3.2758130	-1.2272780	2.7615060
C	2.2236270	1.8010170	-1.8538670
H	2.4170910	2.8710320	-1.8326540
H	2.7840260	1.3435500	-2.6670610
H	1.1598560	1.6220520	-2.0124330

E(UwB97XD, vacuo)	-860.527799
Zero-point correction	0.258311
Thermal correction to Energy	0.274398
Thermal correction to Enthalpy	0.275342
Thermal correction to Gibbs Free Energy	0.208733
E(UwB97XD, MeCN)	-860.543436

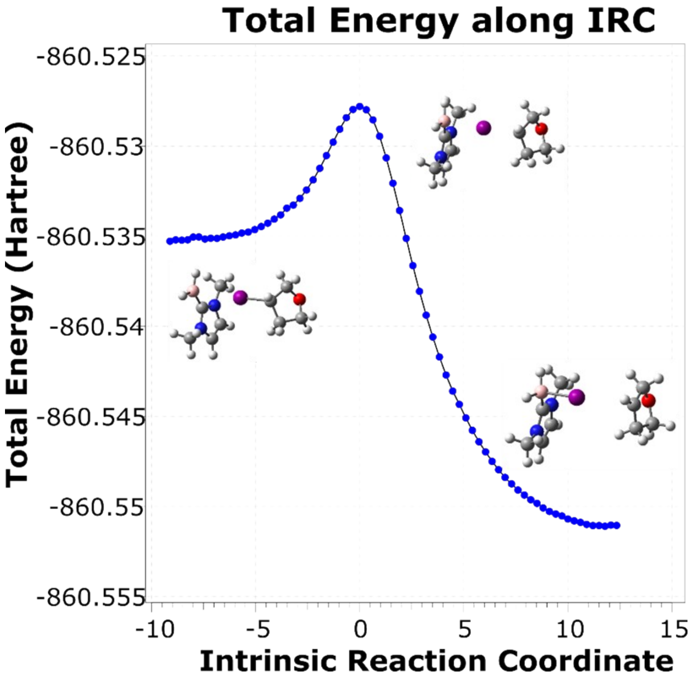

2. Radical addition step

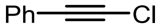

C	-2.6203120	-1.2004020	-0.0000060
C	-1.2363780	-1.2045850	0.0000050
C	-0.5308980	-0.0000950	0.0000010
C	-1.2362050	1.2045100	0.0000030
C	-2.6201290	1.2005350	-0.0000030
C	-3.3153830	0.0001140	-0.0000130
H	-3.1589080	-2.1394040	-0.0000100
H	-0.6900430	-2.1385900	0.0000150
H	-0.6897160	2.1384270	0.0000120
H	-3.1585970	2.1396110	-0.0000080
H	-4.3978810	0.0002000	-0.0000180
C	0.8988700	-0.0001690	0.0000220
C	2.0981520	-0.0000740	0.0000390
Cl	3.7334610	0.0000440	-0.0000170

E(UwB97XD, vacuo)	-767.991845
Zero-point correction	0.102345
Thermal correction to Energy	0.109797
Thermal correction to Enthalpy	0.110741
Thermal correction to Gibbs Free Energy	0.069181
E(UwB97XD, MeCN)	-767.995009

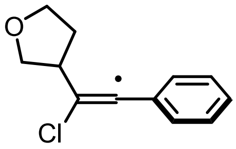

C	3.7889420	0.1147410	0.9925840
C	2.4492940	0.4088590	1.1157190
C	1.5483700	0.0955710	0.0683570
C	2.0607800	-0.5293730	-1.0950490
C	3.4038300	-0.8148720	-1.1974300
C	4.2759330	-0.4972910	-0.1593570
H	4.4669010	0.3633230	1.7991830
H	2.0676440	0.8856140	2.0088680
H	1.3798470	-0.7767970	-1.8985560
H	3.7821650	-1.2906080	-2.0932550
C	0.2014980	0.3654230	0.1885000
C	-0.9982900	0.8324480	0.2066750
C	-2.2584740	0.1476340	0.6744190
C	-3.1582460	-0.2558390	-0.5034110
C	-1.9913310	-1.2160430	1.3186550
H	-2.7744380	0.8201860	1.3605200
C	-2.5534370	-1.5918980	-0.9052590
H	-3.1639610	0.4771010	-1.3082360
H	-4.1816980	-0.3905680	-0.1493420
H	-2.7325470	-1.4143120	2.0996690
H	-0.9950190	-1.2790450	1.7619070
H	-3.2677090	-2.2589040	-1.3897230
H	-1.7006210	-1.4469140	-1.5800090
Cl	-1.2878890	2.4850040	-0.4113060
O	-2.1161940	-2.1955100	0.3002820
H	5.3298850	-0.7262210	-0.2474980

E(UwB97XD, vacuo)	-999.857742
Zero-point correction	0.210213
Thermal correction to Energy	0.223011
Thermal correction to Enthalpy	0.223955
Thermal correction to Gibbs Free Energy	0.166636
E(UwB97XD, MeCN)	-999.864623

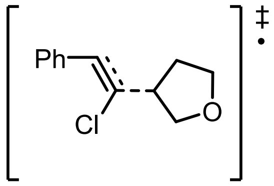

C	-3.5976120	-0.6056230	0.9087600
C	-2.2951060	-1.0676480	0.8889430
C	-1.4211090	-0.6714360	-0.1316610
C	-1.8896980	0.1916390	-1.1296480
C	-3.1939160	0.6483830	-1.0999940
C	-4.0518650	0.2532740	-0.0830770
H	-4.2638030	-0.9165630	1.7033910
H	-1.9369400	-1.7364270	1.6606460
H	-1.2177100	0.5018870	-1.9187460

H	-3.5429330	1.3205270	-1.8733490
C	-0.0807600	-1.1317160	-0.1497650
C	1.1204490	-1.3287610	-0.0650120
C	2.1389410	0.5721200	0.9124250
C	2.7874400	1.2074320	-0.2726950
C	1.0236000	1.4676990	1.3353550
H	2.6938310	-0.0092290	1.6360020
C	1.6378300	2.0497420	-0.8176390
H	3.1831650	0.4940590	-0.9969410
H	3.6190030	1.8501610	0.0442630
H	1.2409010	1.9815950	2.2796620
H	0.0815480	0.9206360	1.4668250
H	1.9616170	2.9506630	-1.3386690
H	1.0197310	1.4510390	-1.4986520
Cl	2.4751660	-2.2648160	-0.3173730
O	0.8858290	2.4460680	0.3147150
H	-5.0720270	0.6143520	-0.0627570

E(UwB97XD, vacuo)	-999.785819
Zero-point correction	0.206444
Thermal correction to Energy	0.219628
Thermal correction to Enthalpy	0.220572
Thermal correction to Gibbs Free Energy	0.162796
E(UwB97XD, MeCN)	-999.792004

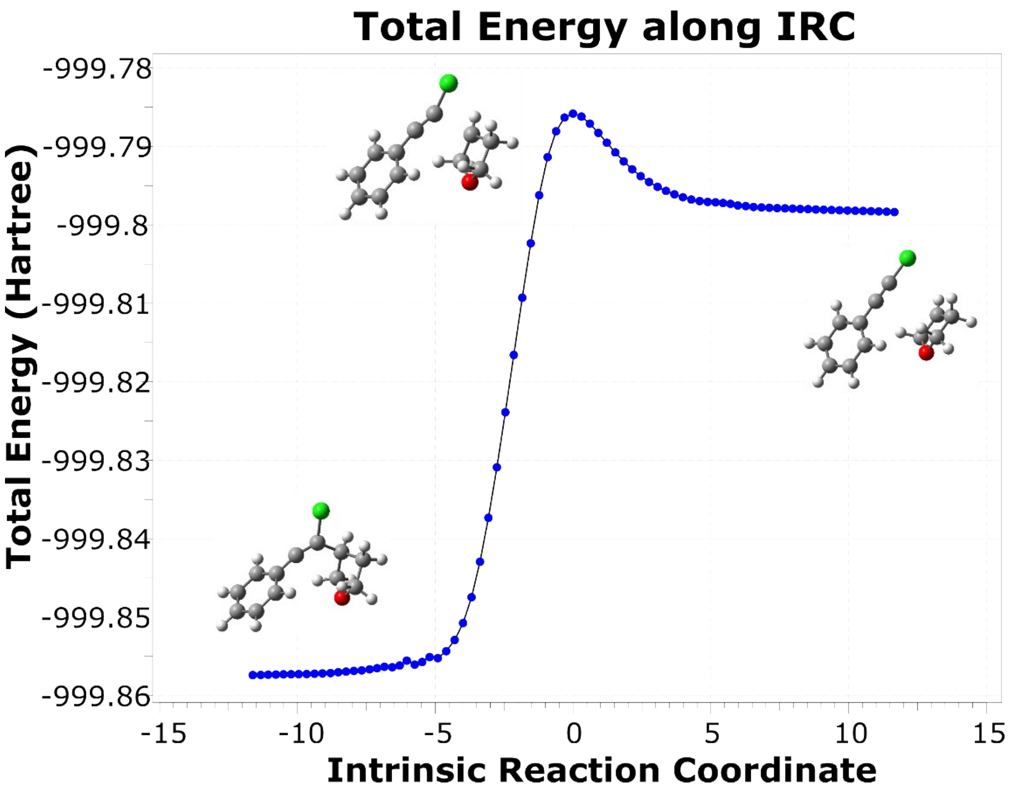

3. HAT step

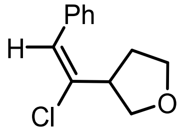

C	4.0223900	0.2790340	0.7492570
C	2.8616990	1.0182270	0.5985670
C	1.7898360	0.5234590	-0.1444590
C	1.9285820	-0.7180410	-0.7626930
C	3.0908840	-1.4580800	-0.6140470
C	4.1391950	-0.9651860	0.1467750
H	4.8398310	0.6762100	1.3376550
H	2.7756570	1.9897870	1.0707290
H	1.1280440	-1.0963760	-1.3856100
H	3.1804960	-2.4193560	-1.1040680
C	0.5744370	1.3468130	-0.2846190
C	-0.6802170	0.9299850	-0.1404710
C	-1.1521570	-0.4388650	0.2391370
C	-2.1834910	-0.4893280	1.3942120
C	-1.8792640	-1.1858100	-0.9031760
H	-0.2702150	-1.0061920	0.5294840
C	-3.3996440	-1.1607770	0.7546940
H	-2.4188070	0.4967290	1.7902840
H	-1.7906810	-1.0976550	2.2076760
H	-1.2199320	-1.8531880	-1.4579330
H	-2.3270900	-0.4686790	-1.6031290
H	-3.9538220	-1.8072760	1.4334710
H	-4.0839830	-0.4070960	0.3436400
Cl	-1.9593260	2.1026530	-0.4023850
O	-2.8760400	-1.9618960	-0.2851590
H	5.0477300	-1.5424500	0.2609440
H	0.7361150	2.3970150	-0.5003830

E(wB97XD, vacuo)	-1000.530759
Zero-point correction	0.224685
Thermal correction to Energy	0.236927
Thermal correction to Enthalpy	0.237872
Thermal correction to Gibbs Free Energy	0.184146
E(wB97XD, MeCN)	-1000.536767

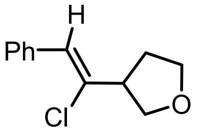

C	-3.8011270	1.0663000	0.3513300
C	-2.4384410	0.8403540	0.4555740
C	-1.9078790	-0.4237750	0.2009980
C	-2.7858620	-1.4554590	-0.1359180

C	-4.1460290	-1.2271050	-0.2500060
C	-4.6589220	0.0384270	-0.0087150
H	-4.1951510	2.0534710	0.5574260
H	-1.7848840	1.6486380	0.7505140
H	-2.3911290	-2.4483800	-0.3180960
H	-4.8072260	-2.0398050	-0.5229190
C	-0.4778060	-0.7455690	0.3152410
C	0.6064830	-0.0396730	0.0032500
C	2.0025790	-0.5534940	0.1900980
C	2.8996660	-0.5767100	-1.0507200
C	2.8655940	0.2485880	1.1756080
H	1.9097070	-1.5754960	0.5657930
C	4.2773570	-0.6929080	-0.4176940
H	2.8081050	0.3606660	-1.6007580
H	2.6651560	-1.4009320	-1.7232750
H	2.7199590	-0.0865600	2.2052050
H	2.6337790	1.3165860	1.1207870
H	4.5256690	-1.7406660	-0.2056020
H	5.0708500	-0.2716330	-1.0365200
Cl	0.5006500	1.5597650	-0.6894290
O	4.2140830	0.0399890	0.7952900
H	-5.7228110	0.2204220	-0.0921690
H	-0.2694300	-1.7460860	0.6833110

E(wB97XD, vacuo)	-1000.529274
Zero-point correction	0.224148
Thermal correction to Energy	0.236727
Thermal correction to Enthalpy	0.237671
Thermal correction to Gibbs Free Energy	0.181753
E(wB97XD, MeCN)	-1000.536006

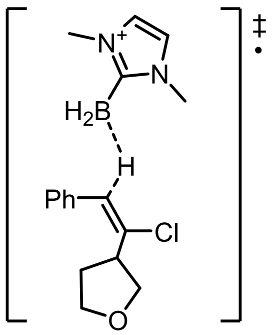

C	1.1227150	2.5072010	-1.2445170
C	0.5892120	1.2439750	-1.0672200
C	-0.2140200	0.9525520	0.0456400
C	-0.4302620	1.9662590	0.9899020
C	0.1003660	3.2287030	0.8026250
C	0.8785830	3.5094290	-0.3140670
H	1.7307850	2.7128180	-2.1170940
H	0.7879260	0.4626510	-1.7908530
H	-1.0281840	1.7491920	1.8650890
H	-0.0947380	4.0031620	1.5338970

C	-0.7015200	-0.3705630	0.2585280
C	-1.7297260	-1.1139620	-0.0406550
C	-3.0143710	-0.7535570	-0.7543370
C	-4.1811280	-0.6084910	0.2373380
C	-3.0329790	0.6319730	-1.3979700
H	-3.2188400	-1.5290820	-1.4934280
C	-4.0179690	0.8332130	0.6967660
H	-4.1407490	-1.3271960	1.0535740
H	-5.1267530	-0.7379760	-0.2923040
H	-3.7334540	0.6284460	-2.2416900
H	-2.0591850	0.9586450	-1.7609530
H	-4.9609630	1.2954630	0.9941470
H	-3.3194350	0.8979100	1.5391650
Cl	-1.7513030	-2.8210030	0.4713080
O	-3.4865300	1.5432800	-0.4113050
H	1.2903200	4.5001600	-0.4573410
C	3.9144780	0.1375980	-0.7005590
C	3.5408380	-0.9732720	-1.3674340
C	2.5388140	-1.0164380	0.6345850
N	3.2975830	0.0915920	0.5269890
H	4.5583550	0.9485090	-0.9892970
H	3.8043570	-1.3272990	-2.3478810
N	2.6974180	-1.6688640	-0.5347940
B	1.5474440	-1.4465960	1.7826790
H	1.5450040	-2.6436530	1.9500530
H	1.6462380	-0.7738260	2.7798620
H	0.4097170	-1.1409740	1.2154460
C	3.3413280	1.1589130	1.5067220
H	3.2906660	0.7310410	2.5035940
H	4.2729220	1.7085660	1.3860860
H	2.4953480	1.8311350	1.3614190
C	2.0119800	-2.9041800	-0.8602280
H	2.2331510	-3.1638860	-1.8931860
H	2.3433460	-3.7026590	-0.1985510
H	0.9382860	-2.7725720	-0.7310230

E(UwB97XD, vacuo)	-1331.370488
Zero-point correction	0.370741
Thermal correction to Energy	0.392926
Thermal correction to Enthalpy	0.393870
Thermal correction to Gibbs Free Energy	0.316144
E(UwB97XD, MeCN)	-1331.388017

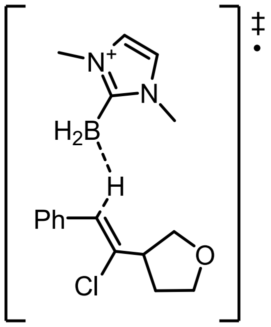

C	-2.3270140	3.0449300	0.8789860
C	-1.0520520	2.5132950	0.9496030
C	-0.6282590	1.5476280	0.0260450
C	-1.5315370	1.1276570	-0.9579340
C	-2.8072340	1.6592060	-1.0205480
C	-3.2135110	2.6187540	-0.1026660
H	-2.6332620	3.7980620	1.5944020
H	-0.3629210	2.8415390	1.7169950
H	-1.2182530	0.3780010	-1.6741160
H	-3.4880680	1.3254510	-1.7939040
C	0.6644890	0.9484190	0.1379390
C	1.9000740	1.2261040	-0.1738200
C	3.1309850	0.3968150	0.0709150
C	3.2704370	-0.2199280	1.4774040
C	3.2257240	-0.8441140	-0.8277400
H	4.0025310	1.0180000	-0.1301530
C	3.8648970	-1.6148260	1.2068170
H	2.3028930	-0.3019230	1.9666110
H	3.9199800	0.3901230	2.1038460
H	3.5924450	-0.6258690	-1.8306350
H	2.2404420	-1.3240190	-0.8921240
H	4.7958800	-1.8003570	1.7423640
H	3.1421520	-2.3927230	1.4721100
Cl	2.2344800	2.7365480	-1.0735270
O	4.1539370	-1.6784030	-0.1833010
H	-4.2105270	3.0371140	-0.1555580
C	-2.8382820	-2.3324780	-1.1932150
C	-3.4925820	-1.7127800	-0.1912320
C	-1.3497460	-1.9055720	0.4261890
N	-1.5260760	-2.4462340	-0.7981540
H	-3.1898450	-2.7066300	-2.1378560
H	-4.5242640	-1.4264370	-0.0955300
N	-2.5693670	-1.4660220	0.7957960
B	0.0248680	-1.7421160	1.1779530
H	-0.0949170	-1.6085190	2.3730390
H	0.8253570	-2.5808890	0.8390640
H	0.4483830	-0.5908540	0.7251730
C	-0.4575300	-3.0048620	-1.6022600
H	0.0611860	-3.7829440	-1.0470170
H	-0.8869390	-3.4228090	-2.5100270
H	0.2598240	-2.2271580	-1.8625420

C	-2.8454310	-0.6949670	1.9911420
H	-2.7617820	0.3700980	1.7717000
H	-3.8521360	-0.9228720	2.3371890

E(wB97XD, vacuo)	-1331.373922
Zero-point correction	0.371213
Thermal correction to Energy	0.393469
Thermal correction to Enthalpy	0.394413
Thermal correction to Gibbs Free Energy	0.316320
E(wB97XD, MeCN)	-1331.389913

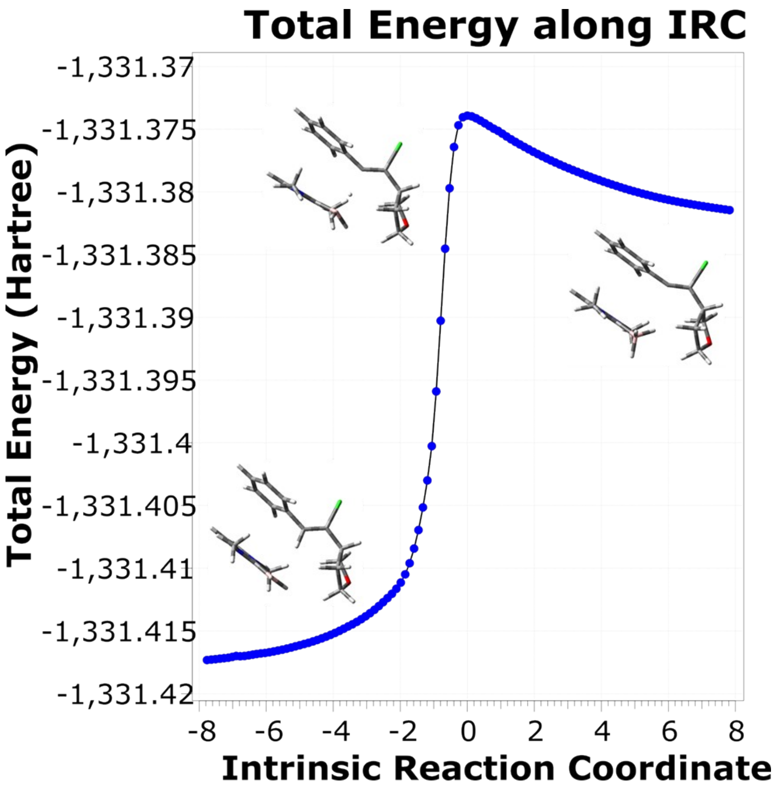

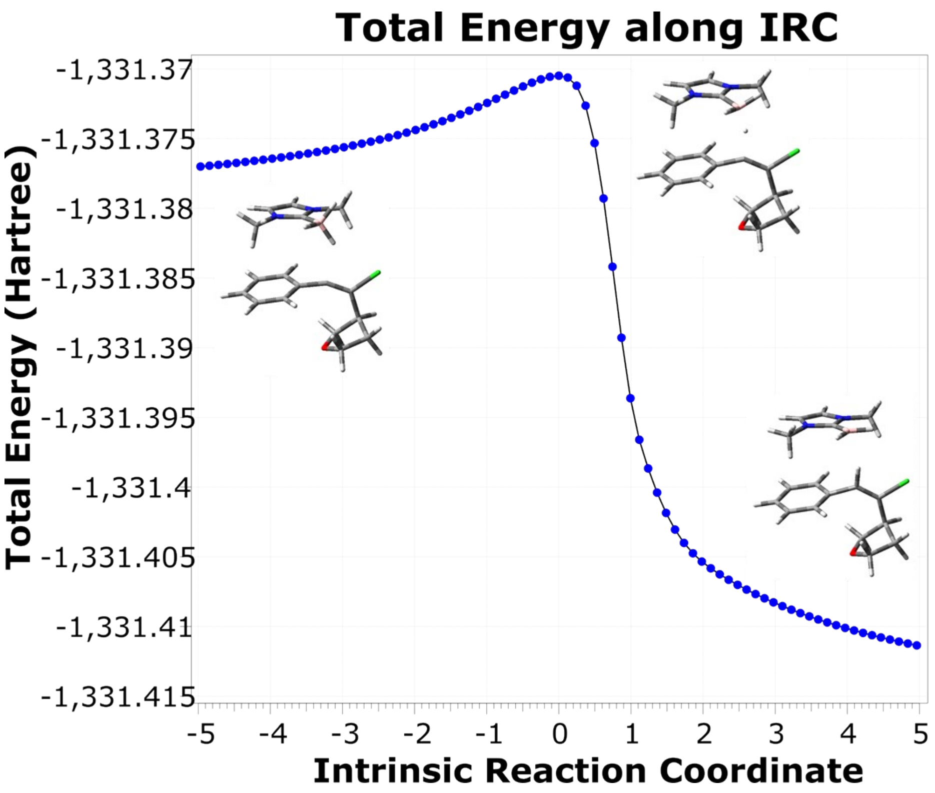
Total Energy along IRC
Total Energy (Hartree)
-1,331.37
-1,331.375
-1,331.38
-1,331.385
-1,331.39
-1,331.395
-1,331.4
-1,331.405
-1,331.41
-1,331.415
-5
-4
-3
-2
-1
0
1
2
3
4
5
Intrinsic Reaction Coordinate

11. References

1. T. M. Masson, S. D. A. Zondag, J. H. A. Schuurmans and T. Noël, Open-source 3D printed reactors for reproducible batch and continuous-flow photon-induced chemistry: design and characterization, *React. Chem. Eng.*, 2024, DOI: 10.1039/d4re00081a.
2. C. D. Matier, J. Schwaben, J. C. Peters and G. C. Fu, Copper-Catalyzed Alkylation of Aliphatic Amines Induced by Visible Light, *J. Am. Chem. Soc.*, 2017, **139**, 17707-17710.
3. B. Górski, A.-L. Barthelemy, J. J. Douglas, F. Juliá and D. Leonori, Copper-catalysed amination of alkyl iodides enabled by halogen-atom transfer, *Nat. Catal.*, 2021, **4**, 623-630.
4. D. Shi, Z. Liu, Z. Zhang, W. Shi and H. Chen, Silver-Catalyzed Synthesis of 1-Chloroalkynes Directly from Terminal Alkynes, *ChemCatChem*, 2015, **7**, 1424-1426.
5. F. Bellina, A. Carpita, L. Mannocci and R. Rossi, First Total Synthesis of Naturally Occurring (−)-Nitidon and Its Enantiomer, *Eur. J. Org. Chem.*, 2004, **2004**, 2610-2619.
6. R. M. Chowdhury and J. D. Wilden, An improved transition-metal-free synthesis of aryl alkynyl sulfides via substitution of a halide at an sp-centre, *Org. Biomol. Chem.*, 2015, **13**, 5859-5861.
7. T. Adak, J. Schulmeister, M. C. Dietl, M. Rudolph, F. Rominger and A. S. K. Hashmi, Gold-Catalyzed Highly Chemo- and Regioselective C-H Bond Functionalization of Phenols with Haloalkynes, *Eur. J. Org. Chem.*, 2019, **2019**, 3867-3876.
8. F. Yang, S. Zhang, T. Shen, J. Ni, J. Zhang, X. Cheng, J. Z. Sun, Z. Fu and B. Z. Tang, Polymerization of 1-chloro-2-phenylacetylene derivatives by using a Brookhart-type catalyst, *Polym. Chem.*, 2019, **10**, 4801-4809.
9. Y. Deng, X. J. Wei, X. Wang, Y. Sun and T. Noel, Iron-Catalyzed Cross-Coupling of Alkynyl and Styrenyl Chlorides with Alkyl Grignard Reagents in Batch and Flow, *Chemistry*, 2019, **25**, 14532-14535.
10. M. Nielsen, M. Christensen and M. Rimmen, Unsymmetrical Coupling of 1-Chloroalkynes and Terminal Alkynes under -Experimental Sonogashira Conditions, *Synlett*, 2013, **24**, 2715-2719.
11. X. F. Wei, X. W. Xie, Y. Shimizu and M. Kanai, Copper(I)-Catalyzed Enantioselective Addition of Enynes to Ketones, *J. Am. Chem. Soc.*, 2017, **139**, 4647-4650.
12. D. Shi, Z. Liu, Z. Zhang, W. Shi and H. Chen, Silver-Catalyzed Synthesis of 1-Chloroalkynes Directly from Terminal Alkynes, *ChemCatChem*, 2015, **7**, 1424-1426.
13. a) Z. Tan and E. Negishi, Widely applicable Pd-catalyzed trans-selective monoalkylation of unactivated 1,1-dichloro-1-alkenes and Pd-catalyzed second substitution for the selective synthesis of E or Z trisubstituted alkenes, *Angew. Chem. Int. Ed.*, 2006, **45**, 762-765. b) R. Ding, S. Huang, Q. Wang, Y. Liu, B. Sun, H. Tian, Dichlorination of olefins with diphenyl sulfoxide/oxalyl chloride. *Synth. Commun.,* 2020, **50**, 2319-2330.
14. A. R. Tripathy, A. Kumar, A. R. Rahmathulla, A. K. Jha and V. R. Yatham, Visible-Light-Driven alpha-Aminoalkyl Radical-Mediated C(sp(^{3}))-C(sp) Cross-Coupling of Iodoalkanes and Alkynyl Bromides, *Org. Lett.*, 2022, **24**, 5186-5191.
15. L. Huo, X. Li, Y. Zhao, L. Li and L. Chu, Site- and Stereoselective Synthesis of Alkenyl Chlorides by Dual Functionalization of Internal Alkynes via Photoredox/Nickel Catalysis, *J. Am. Chem. Soc.*, 2023, **145**, 9876-9885.
16. Z. Tan and E. Negishi, Widely applicable Pd-catalyzed trans-selective monoalkylation of unactivated 1,1-dichloro-1-alkenes and Pd-catalyzed second substitution for the

selective synthesis of E or Z trisubstituted alkenes, *Angew. Chem. Int. Ed.*, 2006, **45**, 762-765.

17. L. Zhang, Modular Synthesis of α-Substituted Alkenyl Acetals by a Palladium-Catalyzed Suzuki Reaction of α-Haloalkenyl Acetals with Organoboranes, *Synlett*, 2020, **32**, 723-727.
18. M. A. Cismesia and T. P. Yoon, Characterizing chain processes in visible light photoredox catalysis, *Chem. Sci.*, 2015, **6**, 5426-5434.
19. H. C. G. and C. A. Parker, A new sensitive chemical actinometer - II. Potassium ferrioxalate as a standard chemical actinometer, *Proc. Math. Phys. Eng. Sci.*, 1997, **235**, 518-536.
20. S. P. Pitre, C. D. McTiernan, W. Vine, R. DiPucchio, M. Grenier and J. C. Scaiano, Visible-Light Actinometry and Intermittent Illumination as Convenient Tools to Study Ru(bpy)3Cl2 Mediated Photoredox Transformations, *Sci. Rep.*, 2015, **5**, 16397.
21. T. Wan, L. Capaldo, D. Ravelli, W. Vitullo, F. J. de Zwart, B. de Bruin and T. Noël, Photoinduced Halogen-Atom Transfer by N-Heterocyclic Carbene-Ligated Boryl Radicals for C(sp^3)-C(sp^3) Bond Formation, *J. Am. Chem. Soc.*, 2023, **145**, 991-999.

12. NMR Spectra

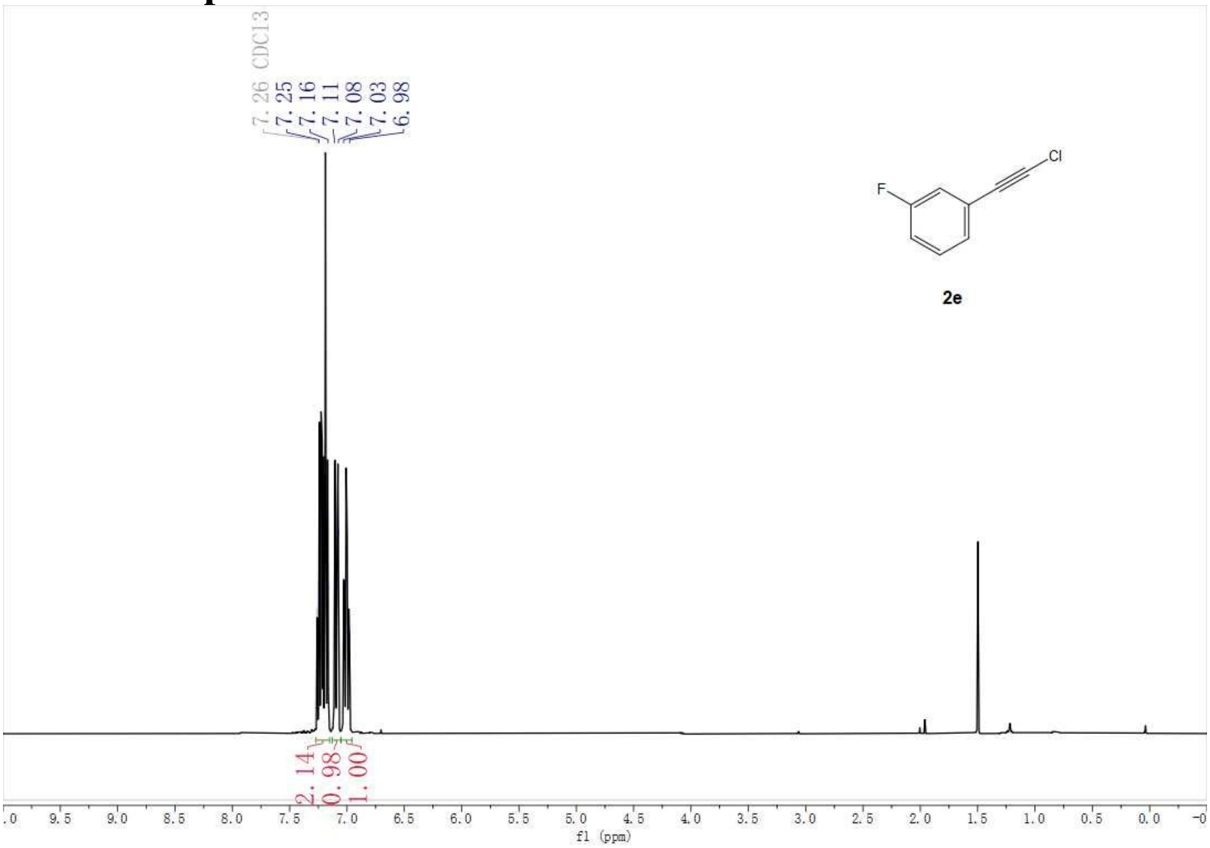

^{1}H-NMR (400 MHz, $CDCl_3$) of **2e**.

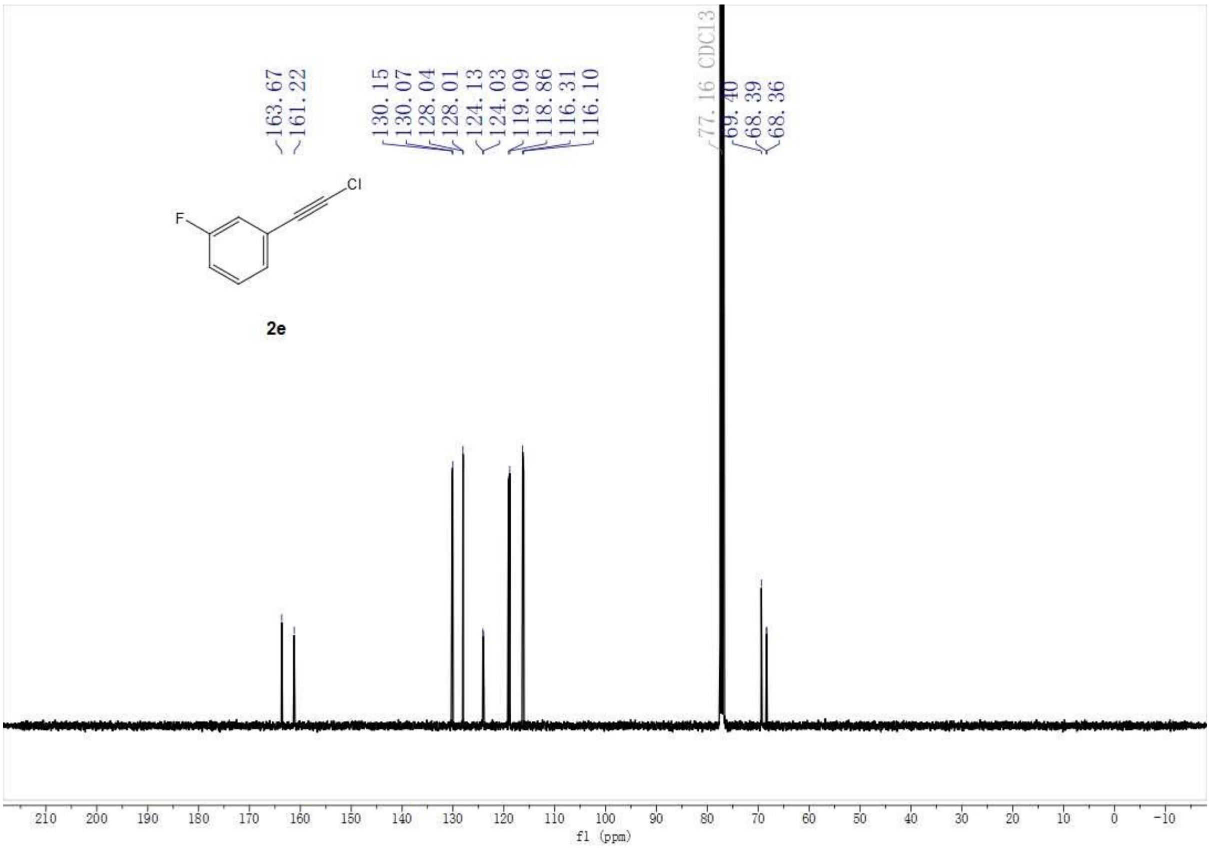

^{13}C-NMR (101 MHz, $CDCl_3$) of **2e**.

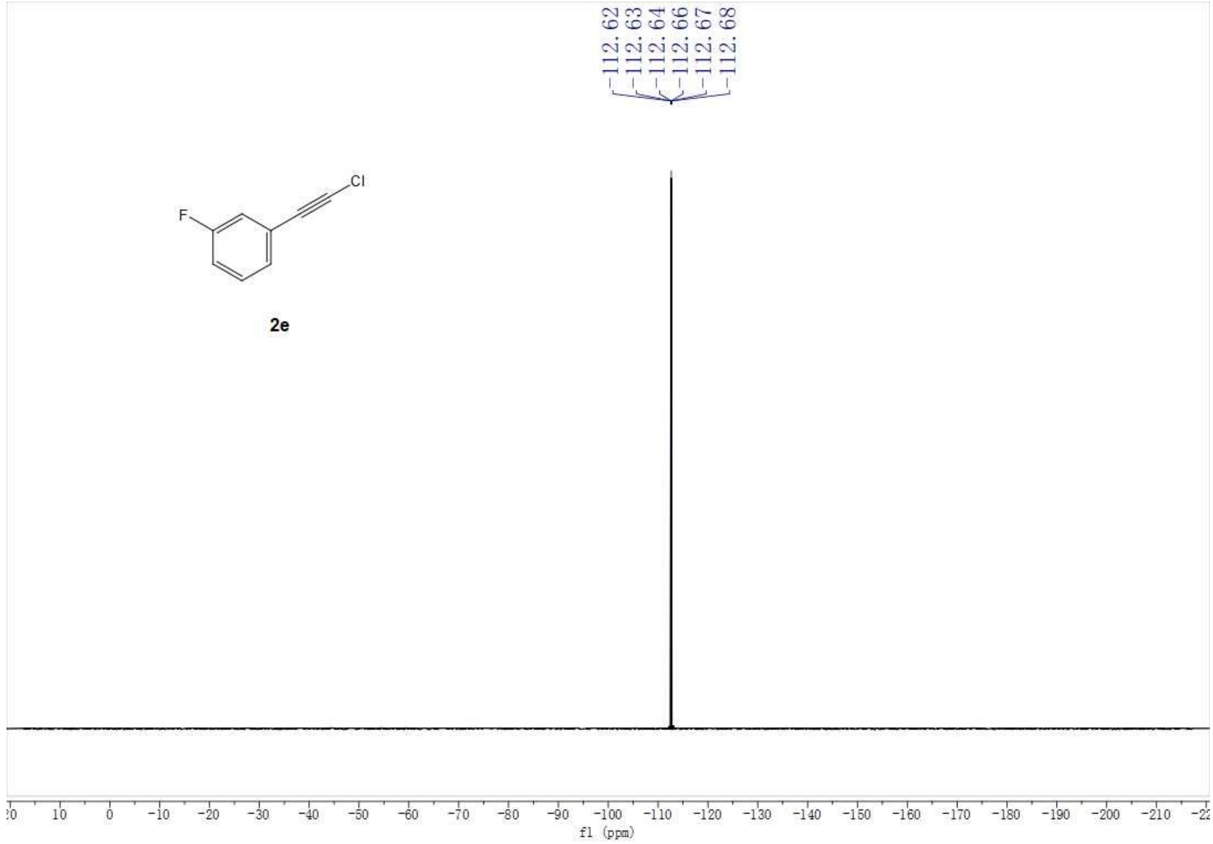

^{19}F-NMR (376 MHz, $CDCl_3$) of **2e**.

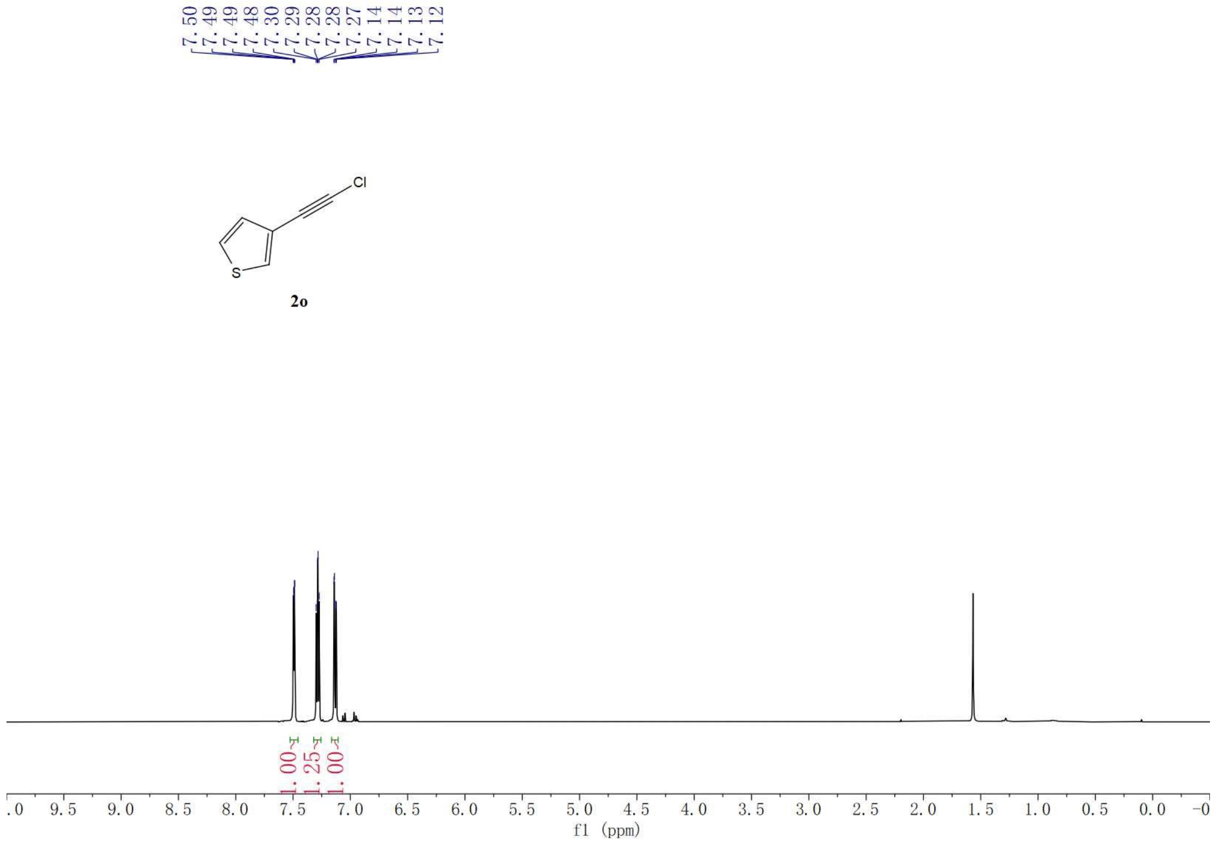

^{1}H-NMR (300 MHz, $CDCl_3$) of **2o**.

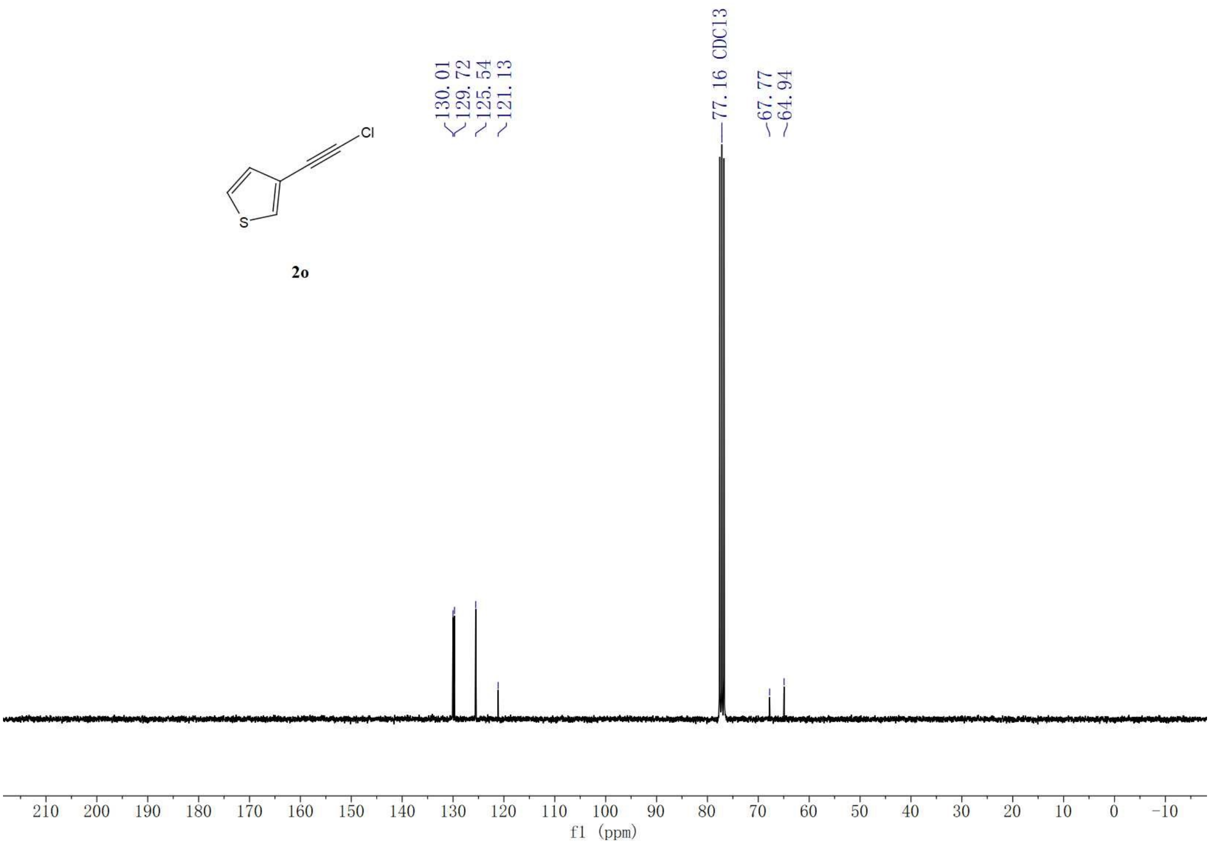

^{13}C-NMR (75 MHz, $CDCl_3$) of **2o**.

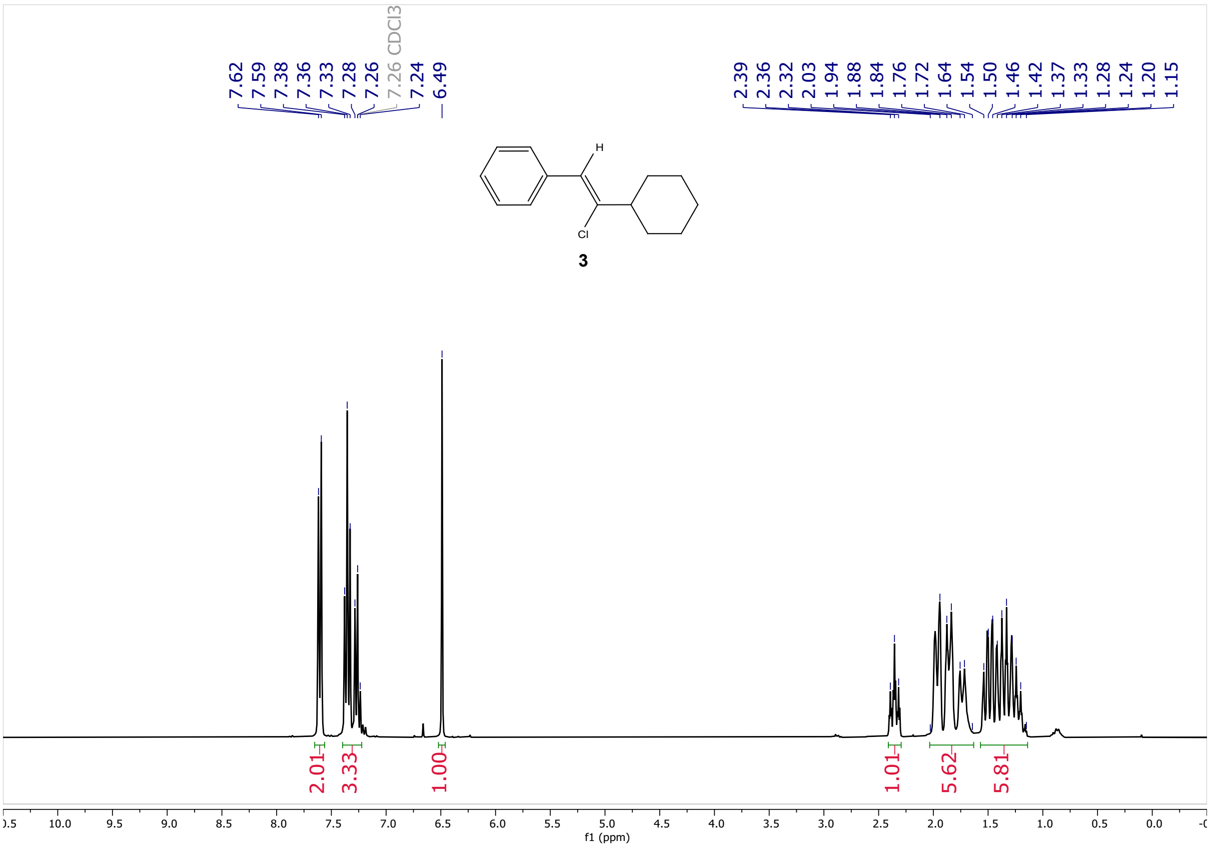

^{1}H-NMR (300 MHz, $CDCl_3$) of **3**, *Z* isomer.

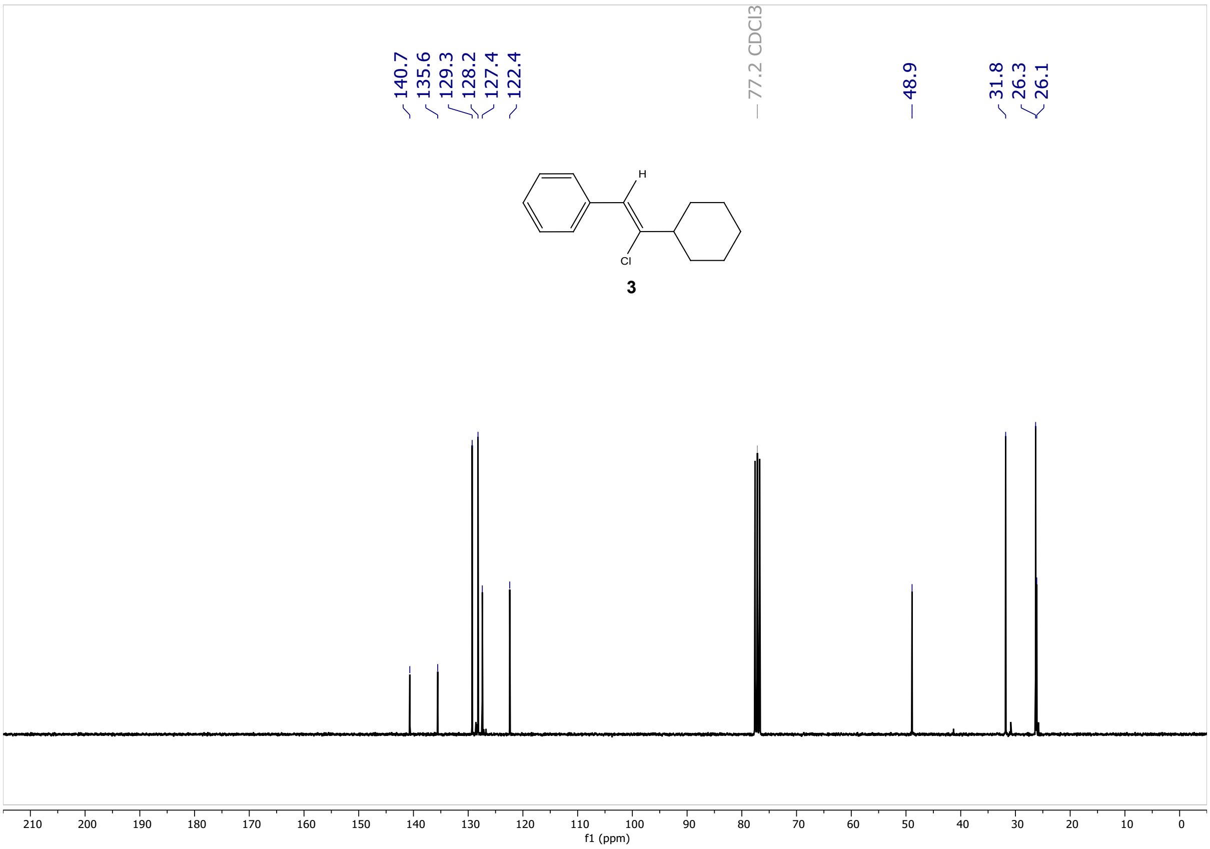

^{13}C-NMR (75 MHz, $CDCl_3$) of **3**, *Z* isomer.

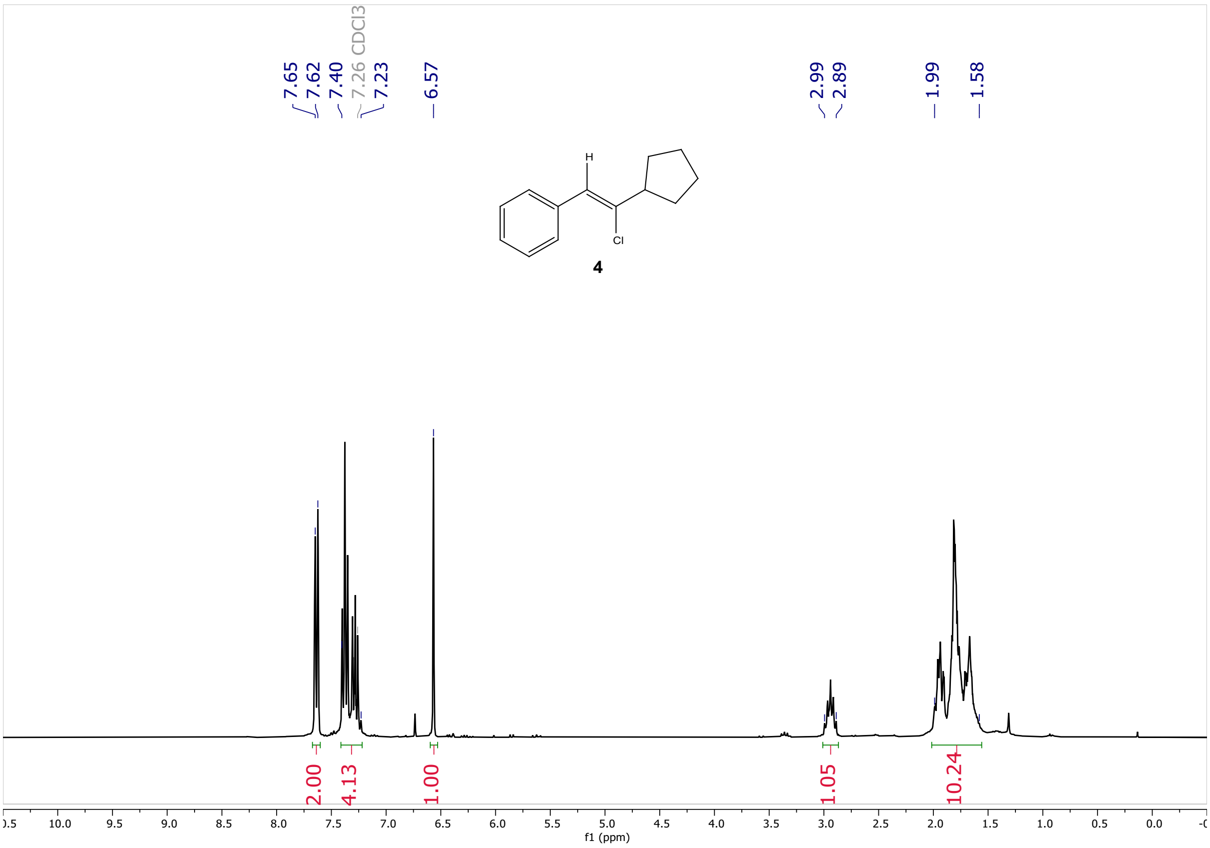

^{1}H-NMR (300 MHz, $CDCl_3$) of **4**, *Z* isomer.

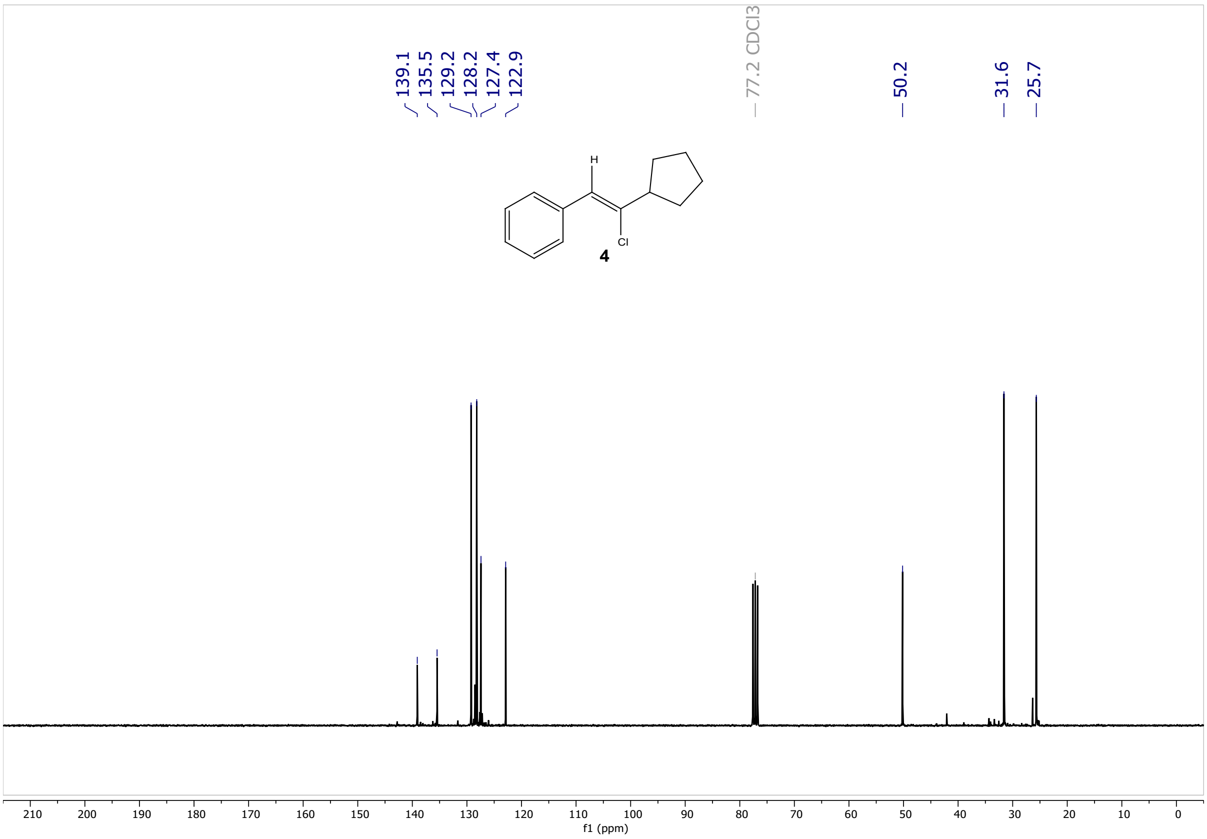

^{13}C-NMR (75 MHz, $CDCl_3$) of **4**, *Z* isomer.

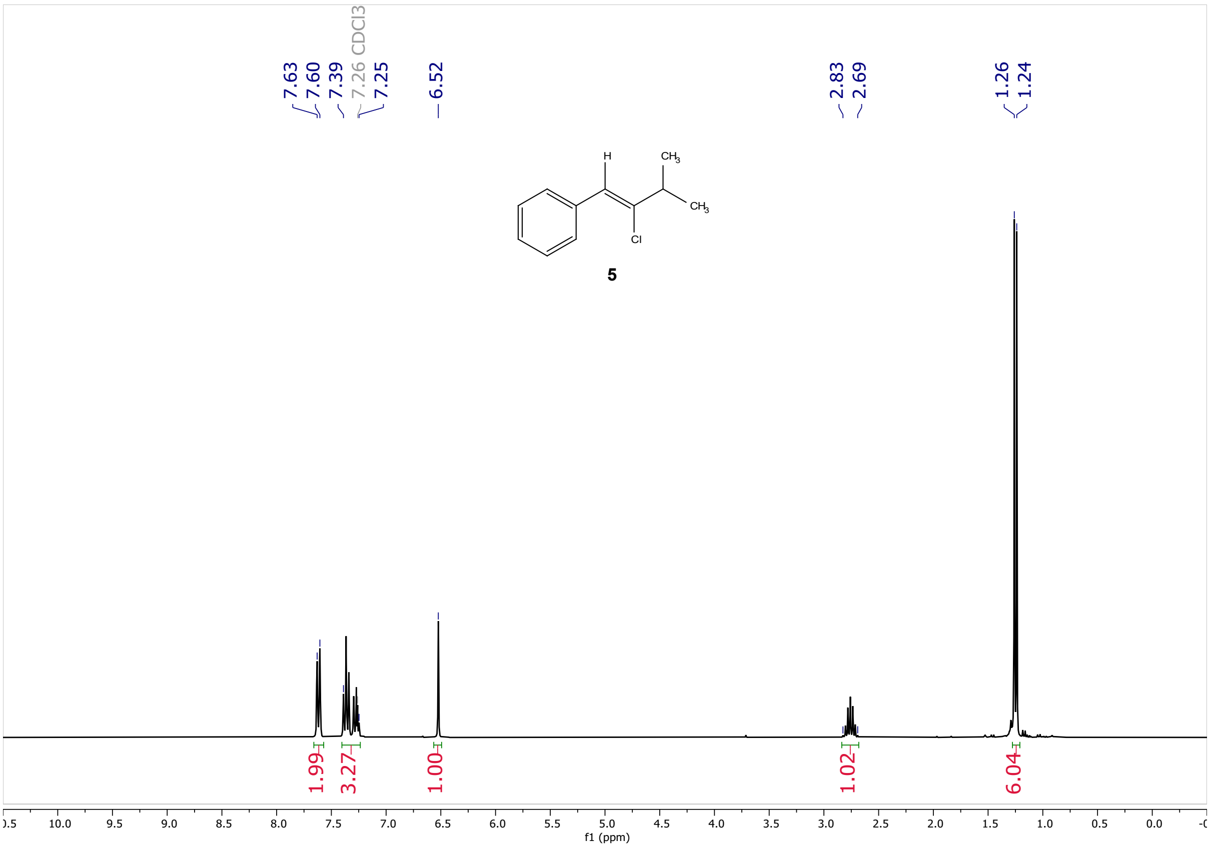

^{1}H-NMR (300 MHz, $CDCl_3$) of **5**, *Z* isomer.

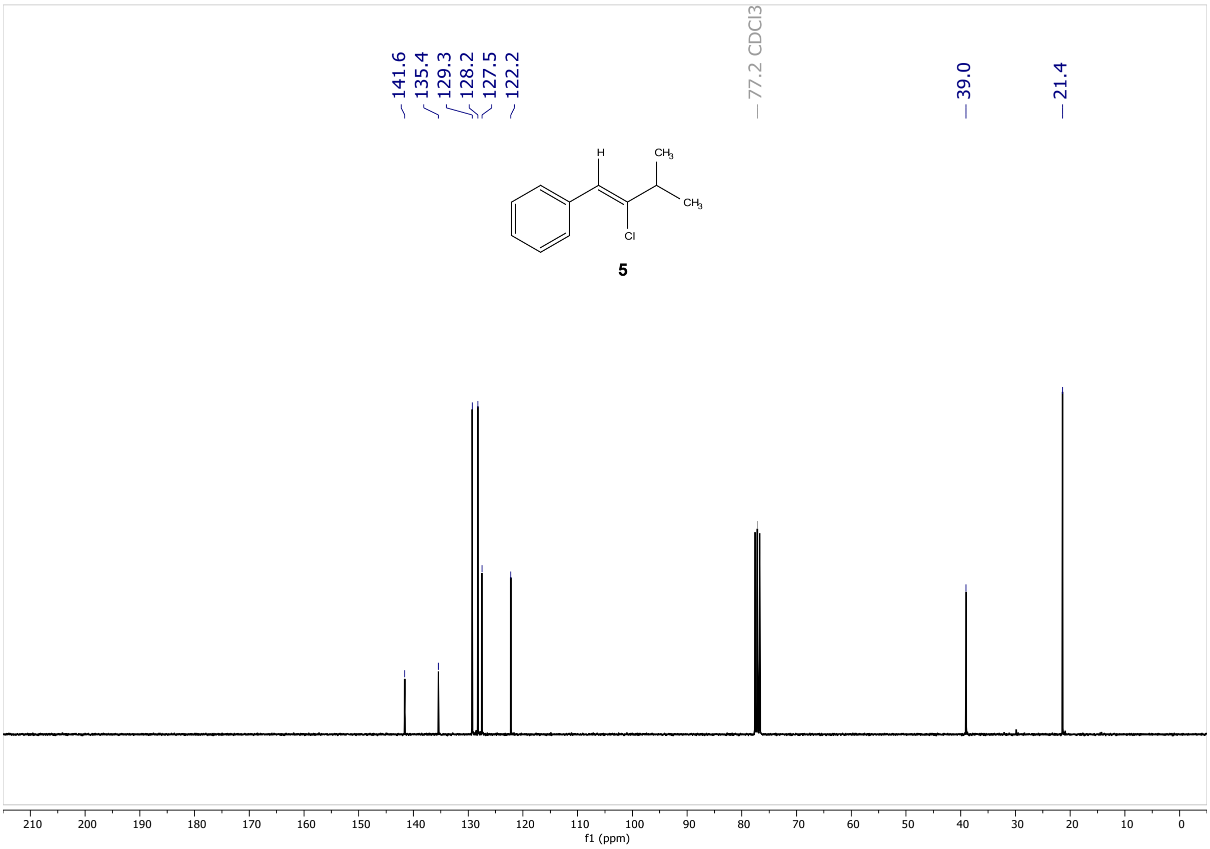

^{13}C-NMR (75 MHz, $CDCl_3$) of **5**, *Z* isomer.

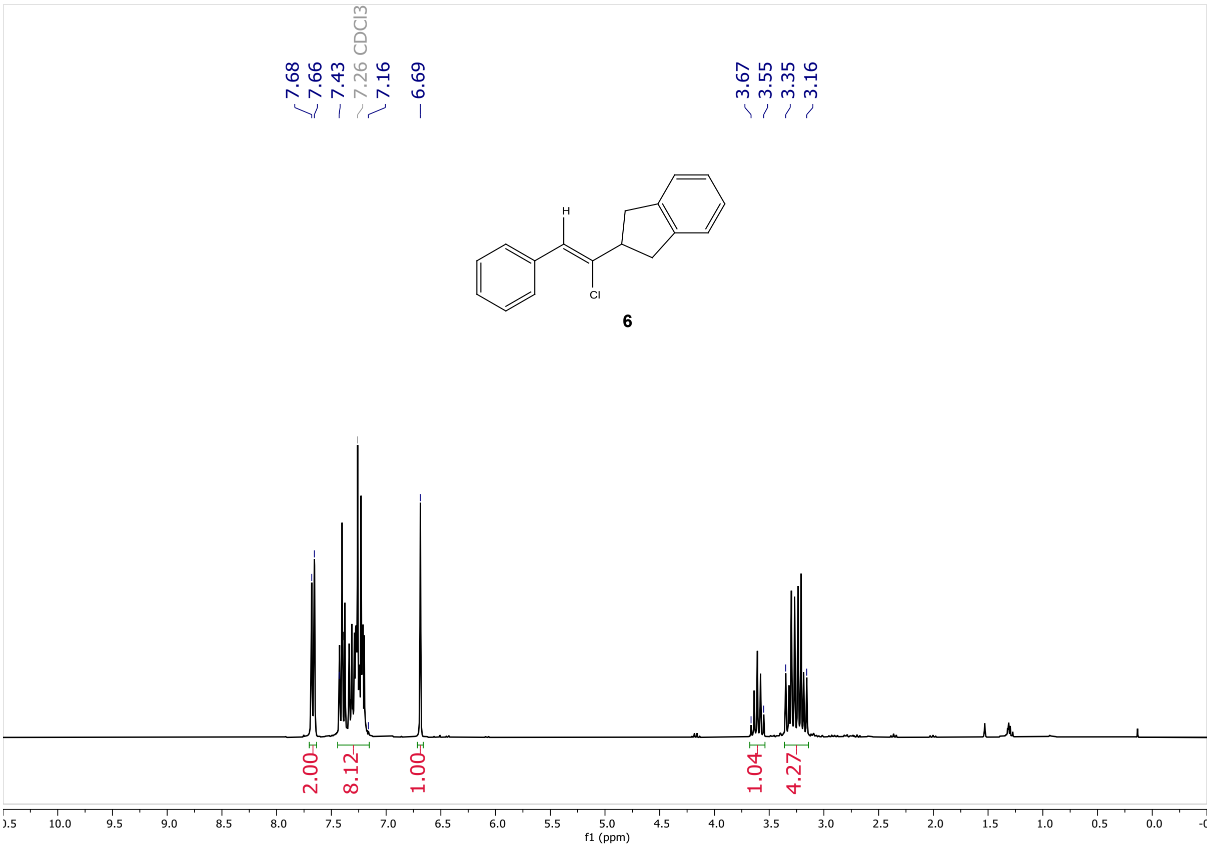

^{1}H-NMR (300 MHz, $CDCl_3$) of **6**, *Z* isomer.

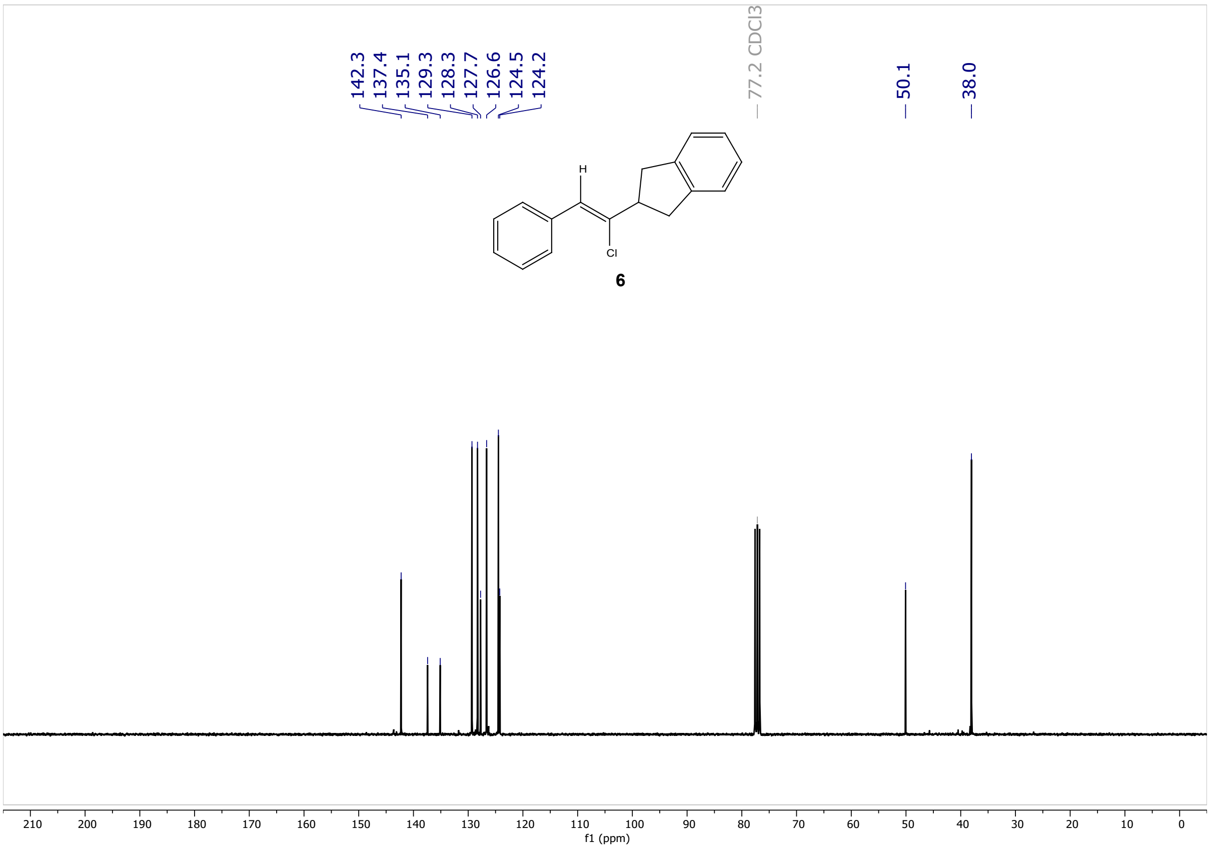

^{13}C-NMR (75 MHz, $CDCl_3$) of **6**, *Z* isomer.

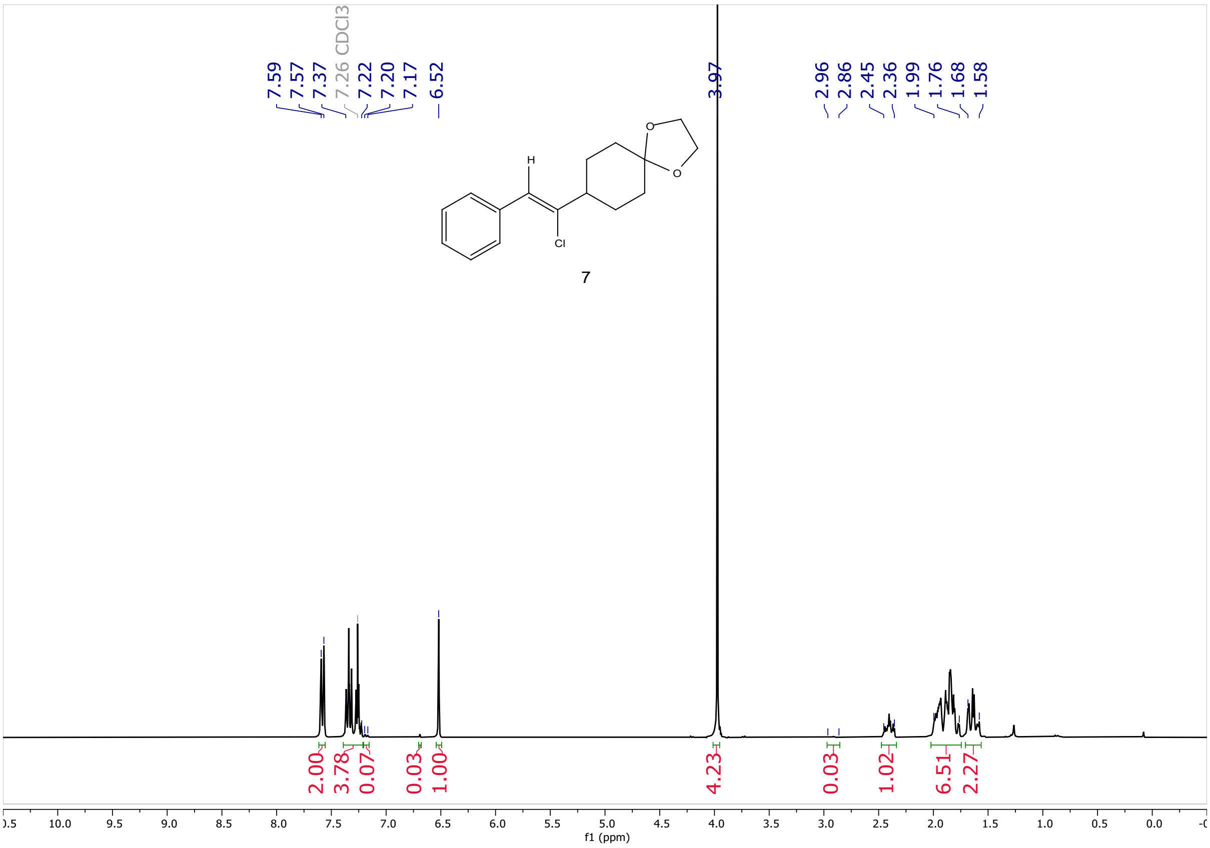

^{1}H-NMR (300 MHz, $CDCl_3$) of **7**, *Z* isomer.

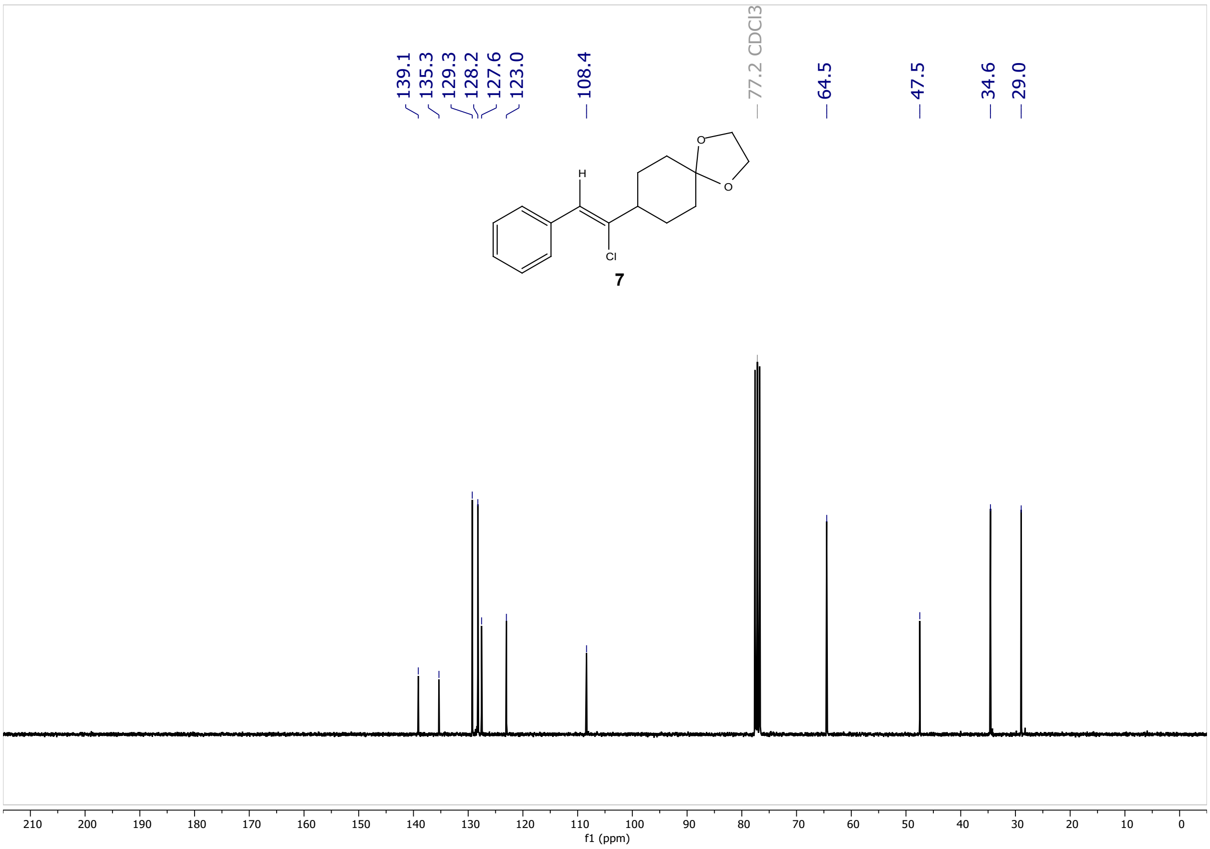

^{13}C-NMR (75 MHz, $CDCl_3$) of **7**, *Z* isomer.

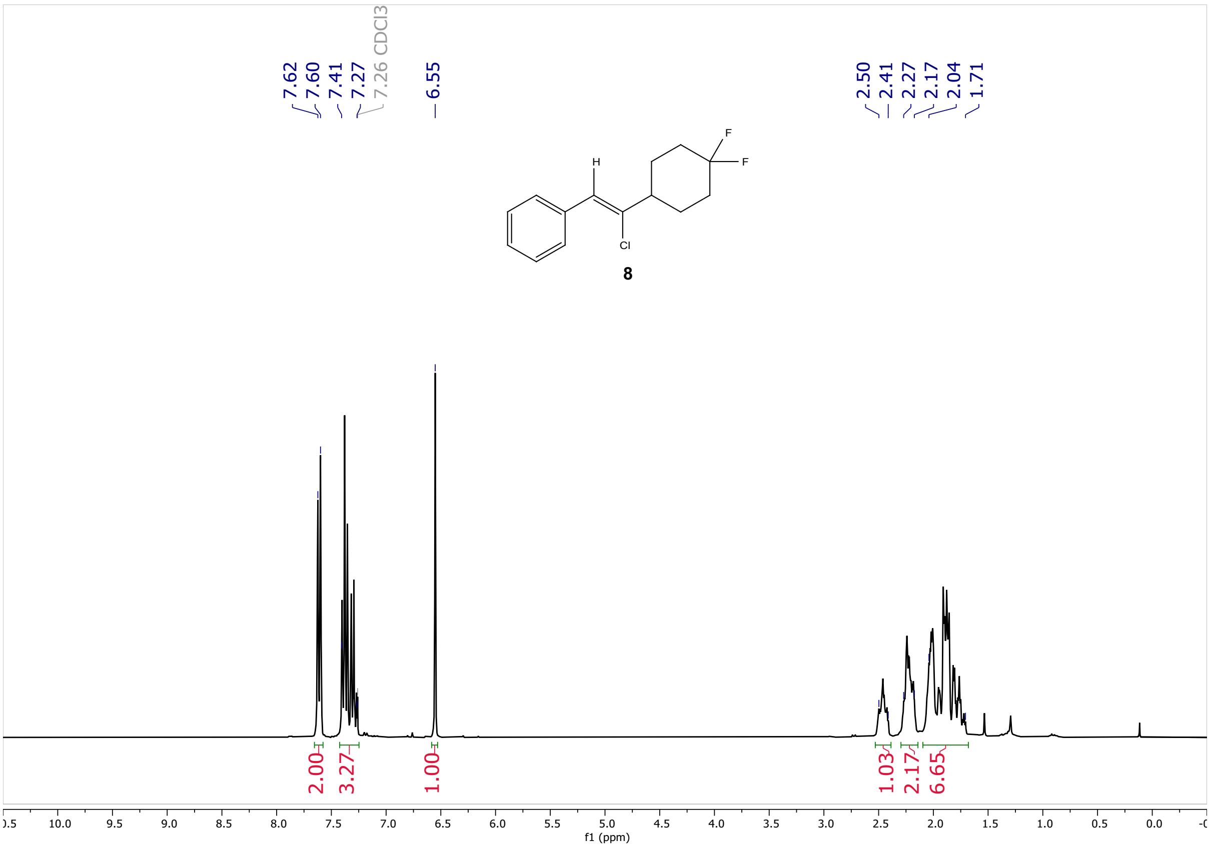

^{1}H-NMR (300 MHz, $CDCl_3$) of **8**, *Z* isomer.

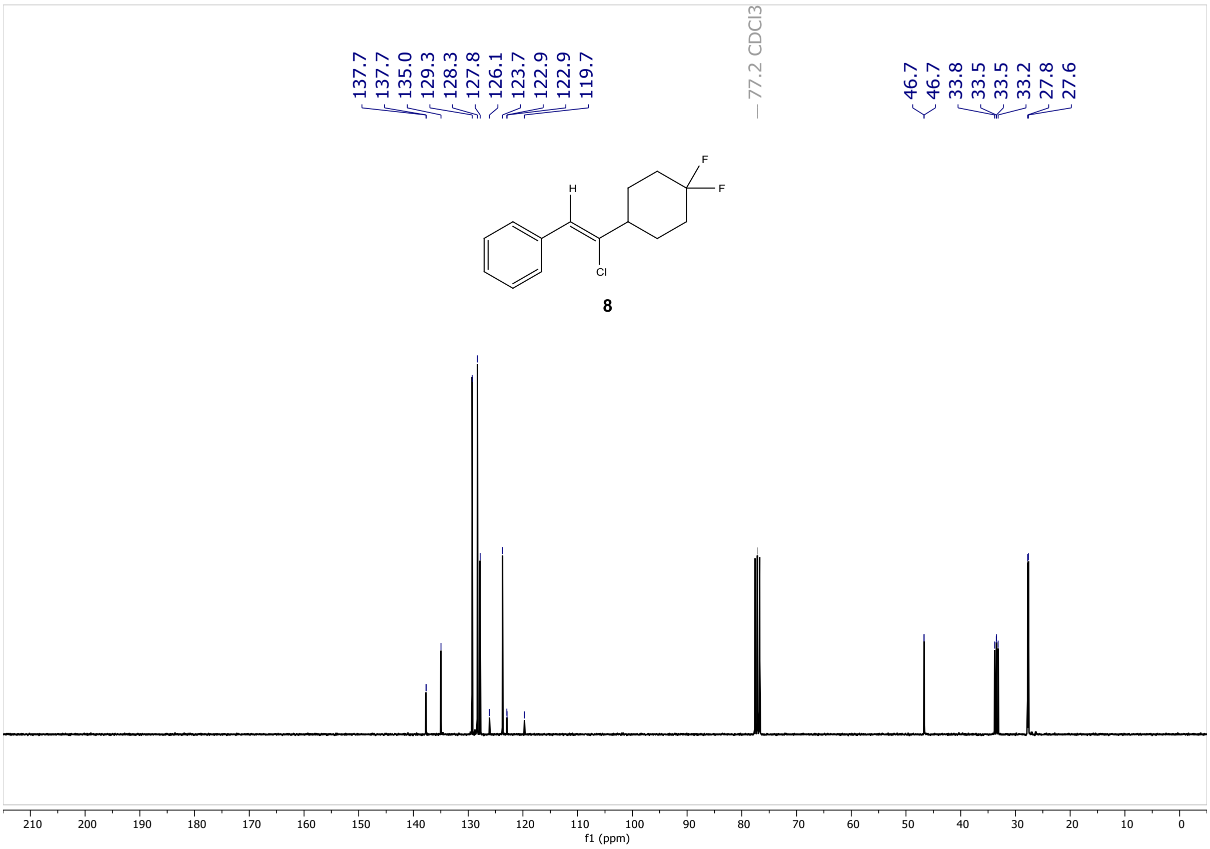

^{13}C-NMR (75 MHz, $CDCl_3$) of **8**, *Z* isomer.

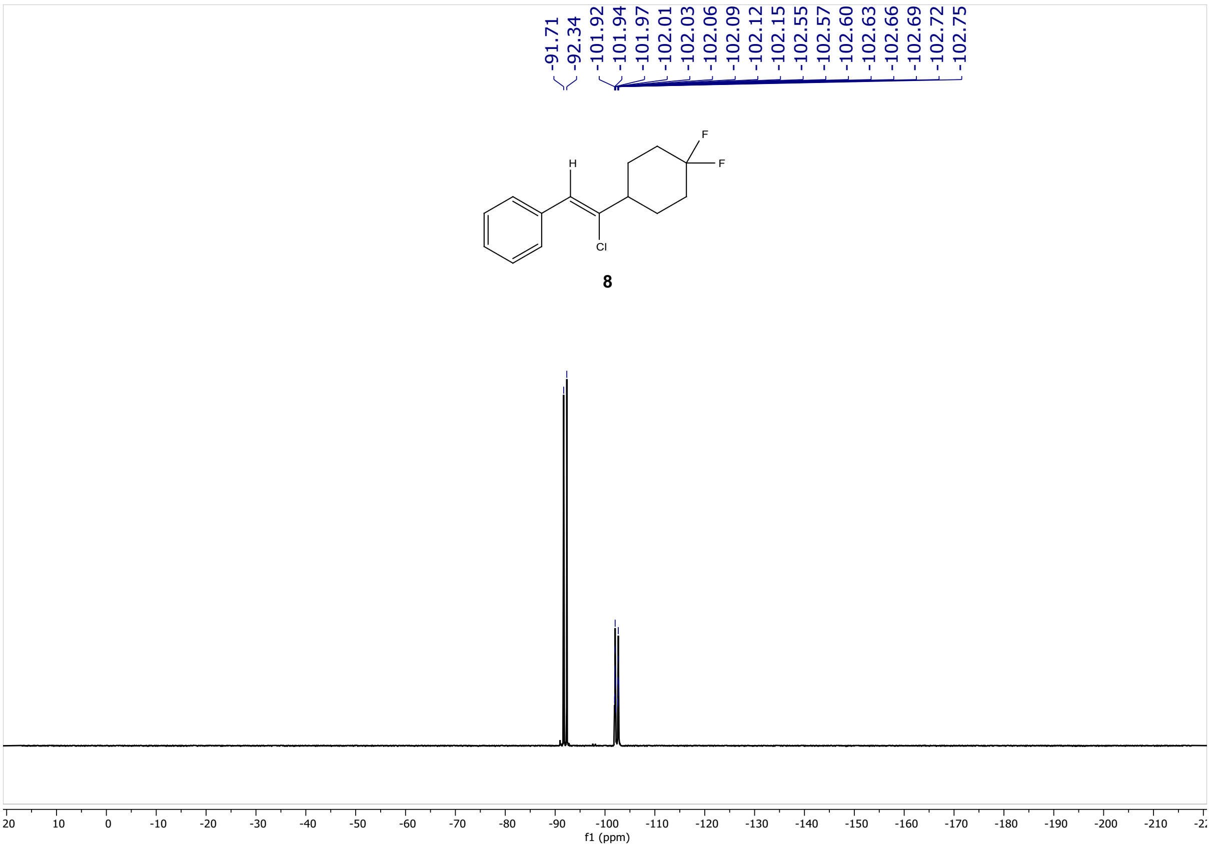

^{19}F-NMR (376 MHz, $CDCl_3$) of **8**, *Z* isomer.

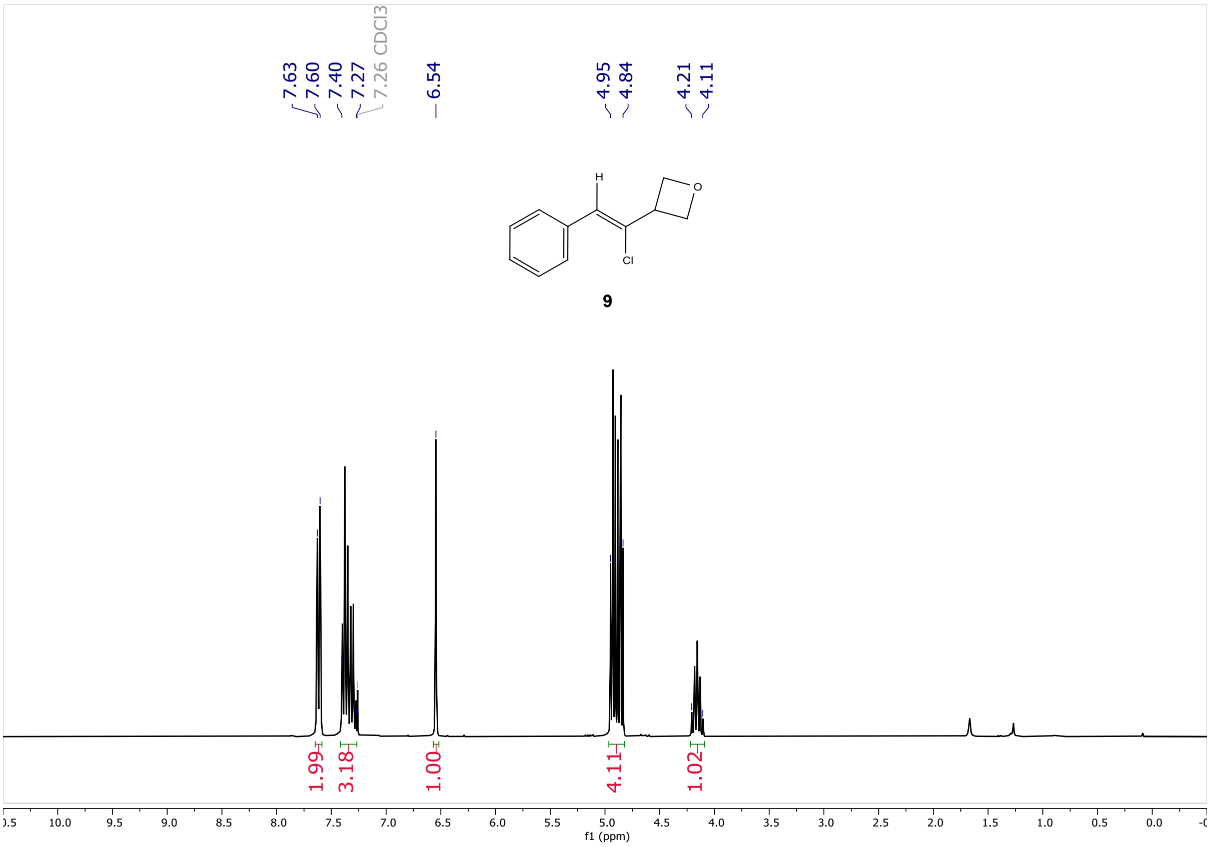

^{1}H-NMR (300 MHz, $CDCl_3$) of **9**, *Z* isomer.

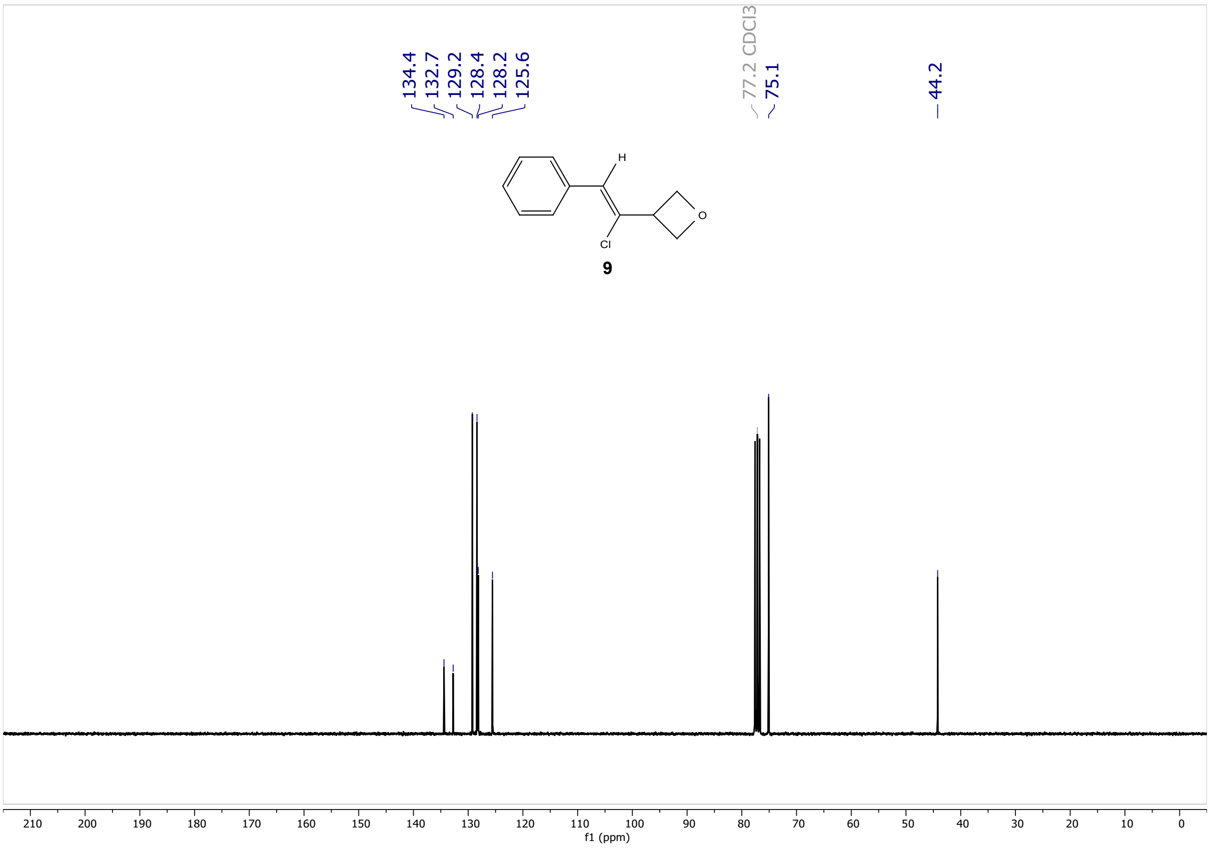

^{13}C-NMR (75 MHz, $CDCl_3$) of **9**, *Z* isomer.

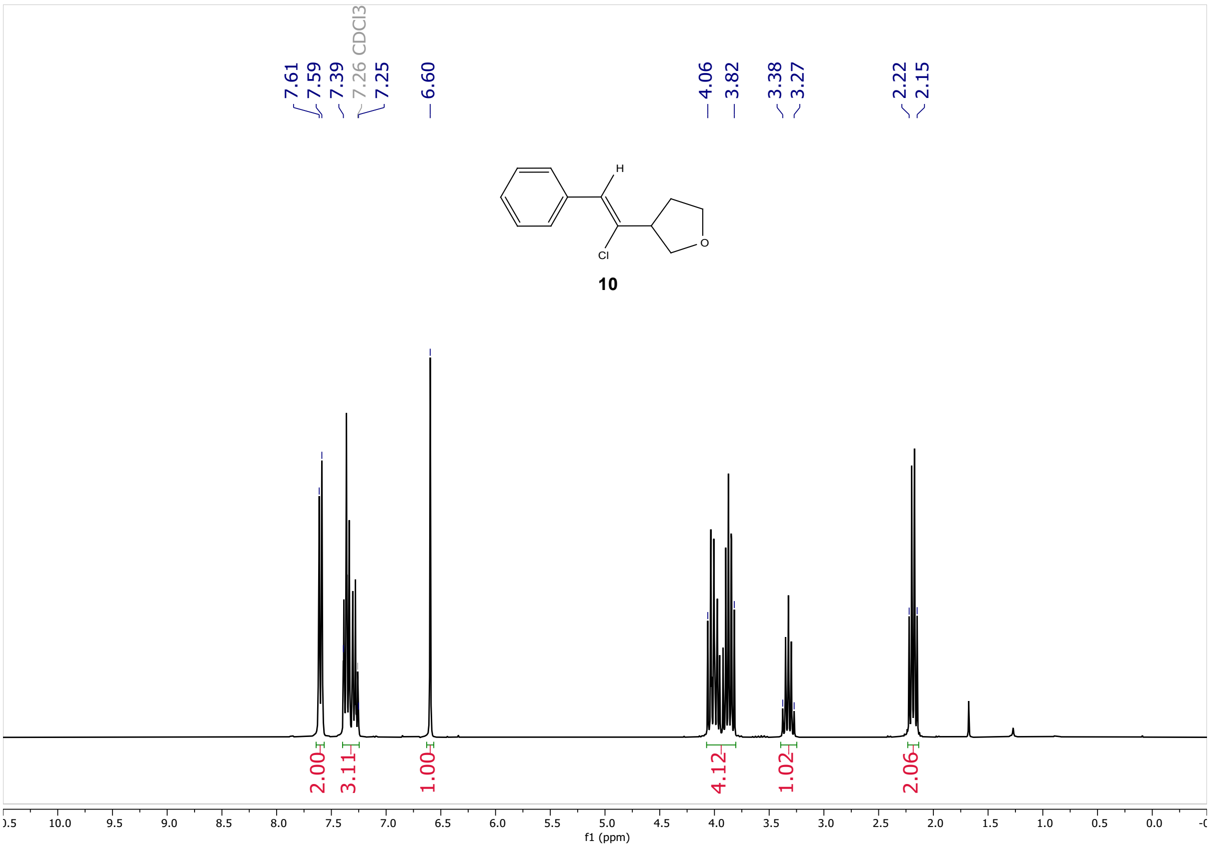

^{1}H-NMR (300 MHz, $CDCl_3$) of **10**, *Z* isomer.

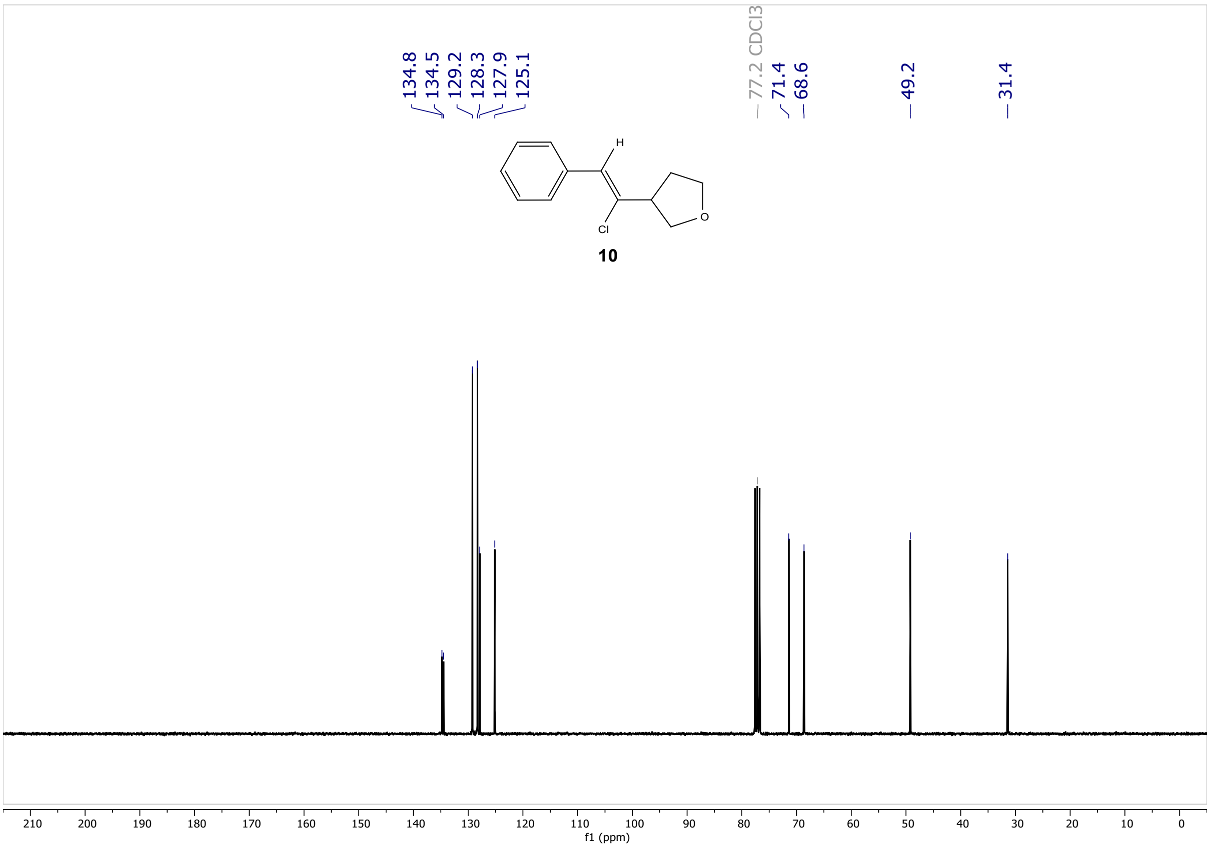

^{13}C-NMR (75 MHz, $CDCl_3$) of **10**, *Z* isomer.

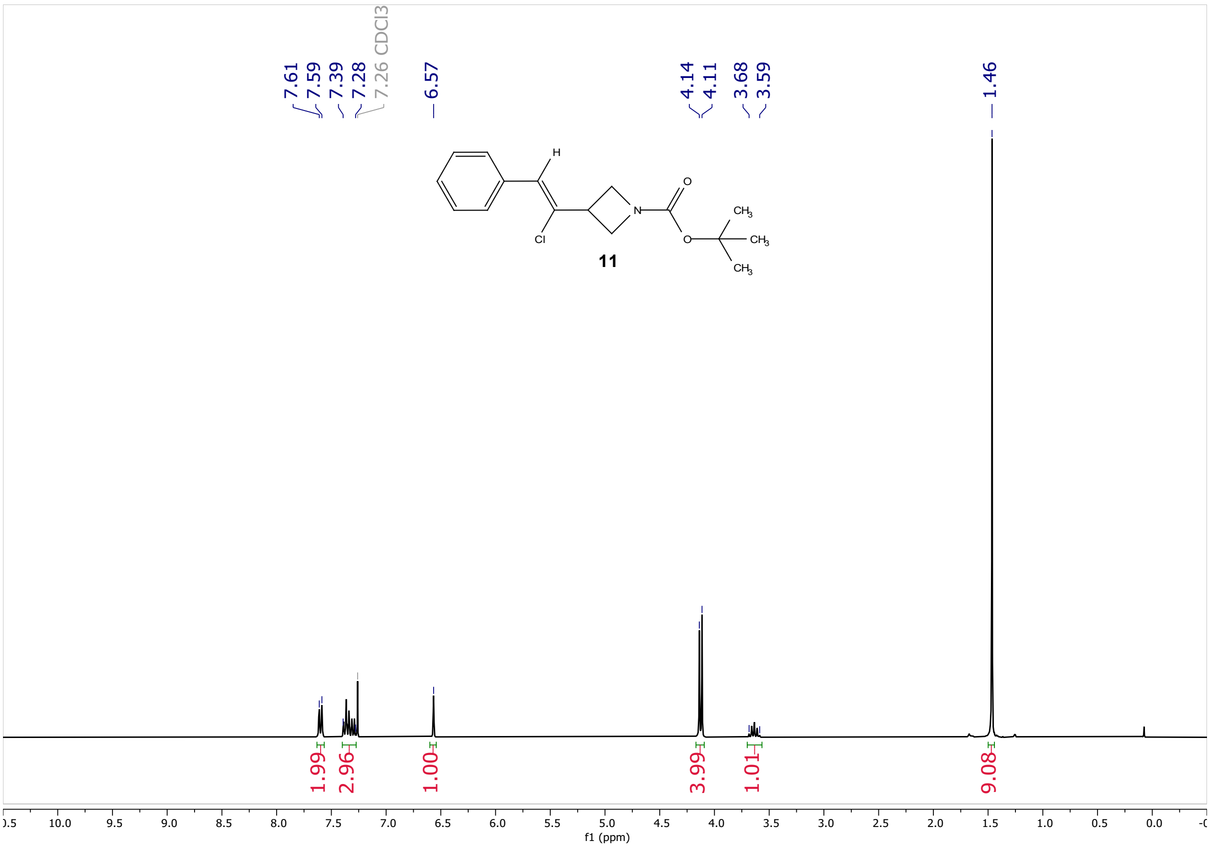

^{1}H-NMR (300 MHz, $CDCl_3$) of **11**, *Z* isomer.

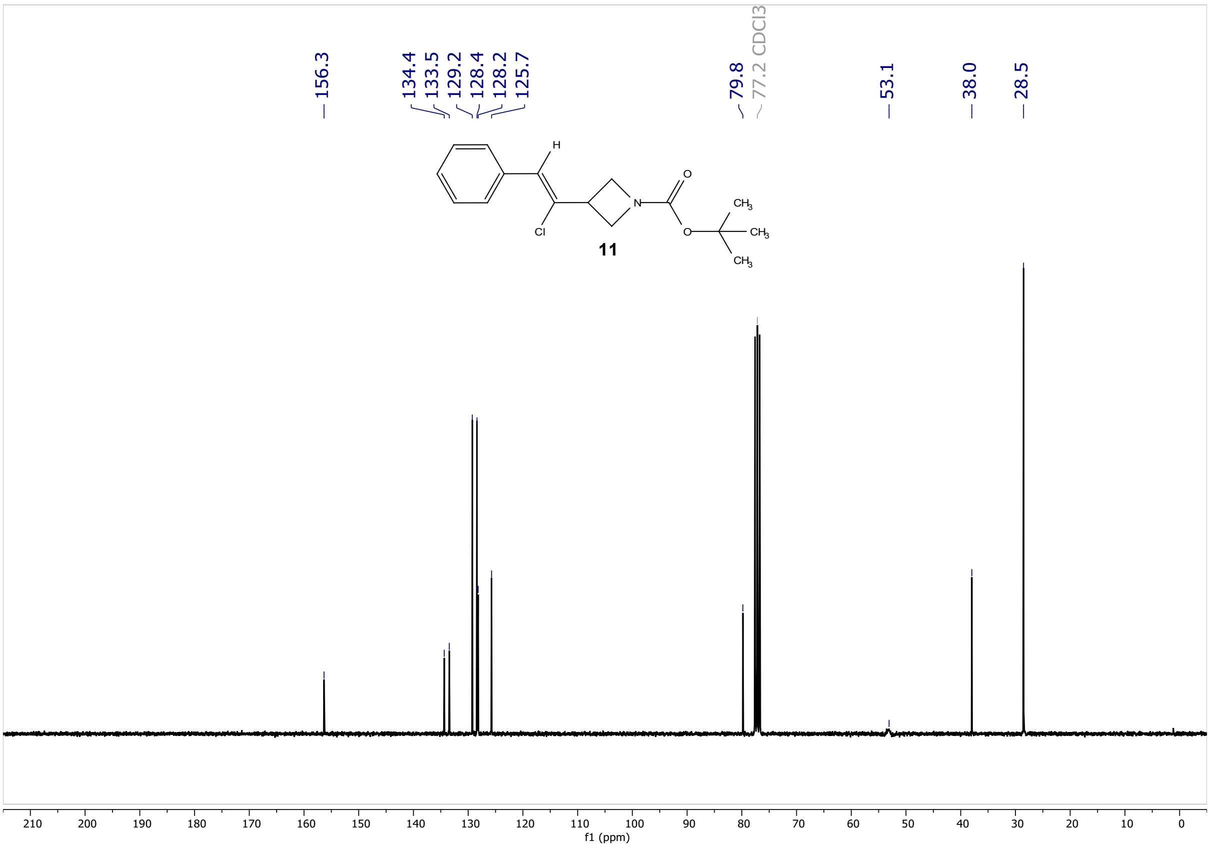

^{13}C-NMR (75 MHz, $CDCl_3$) of **11**, *Z* isomer.

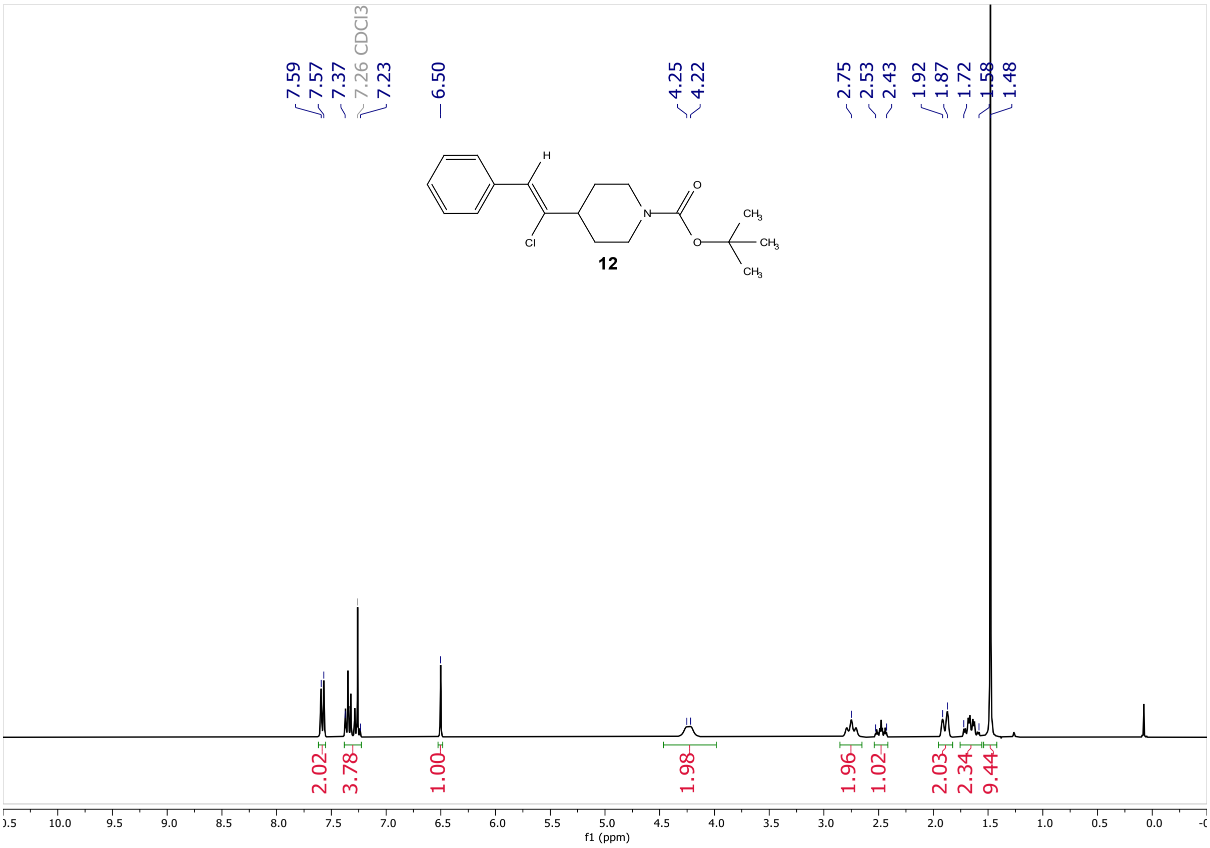

^{1}H-NMR (300 MHz, $CDCl_3$) of **12**, *Z* isomer.

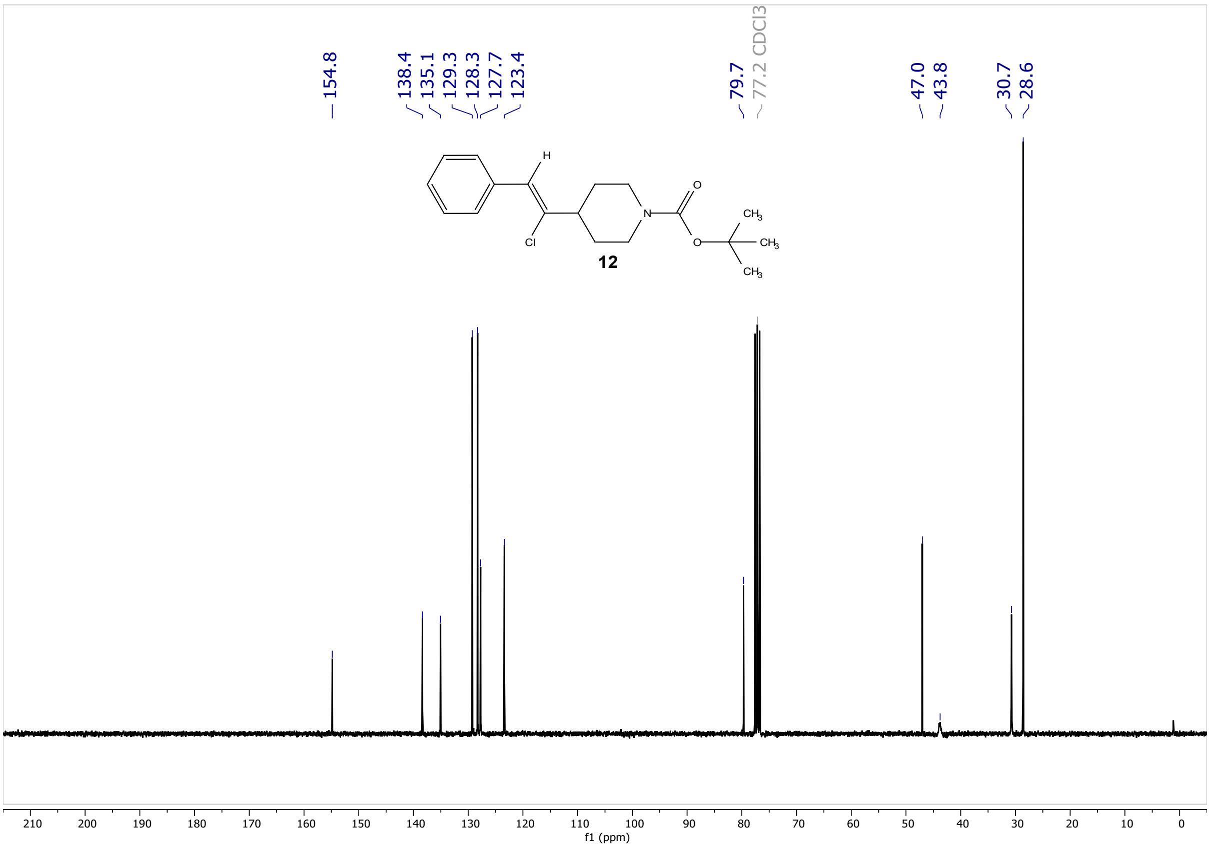

^{13}C-NMR (75 MHz, $CDCl_3$) of **12**, *Z* isomer.

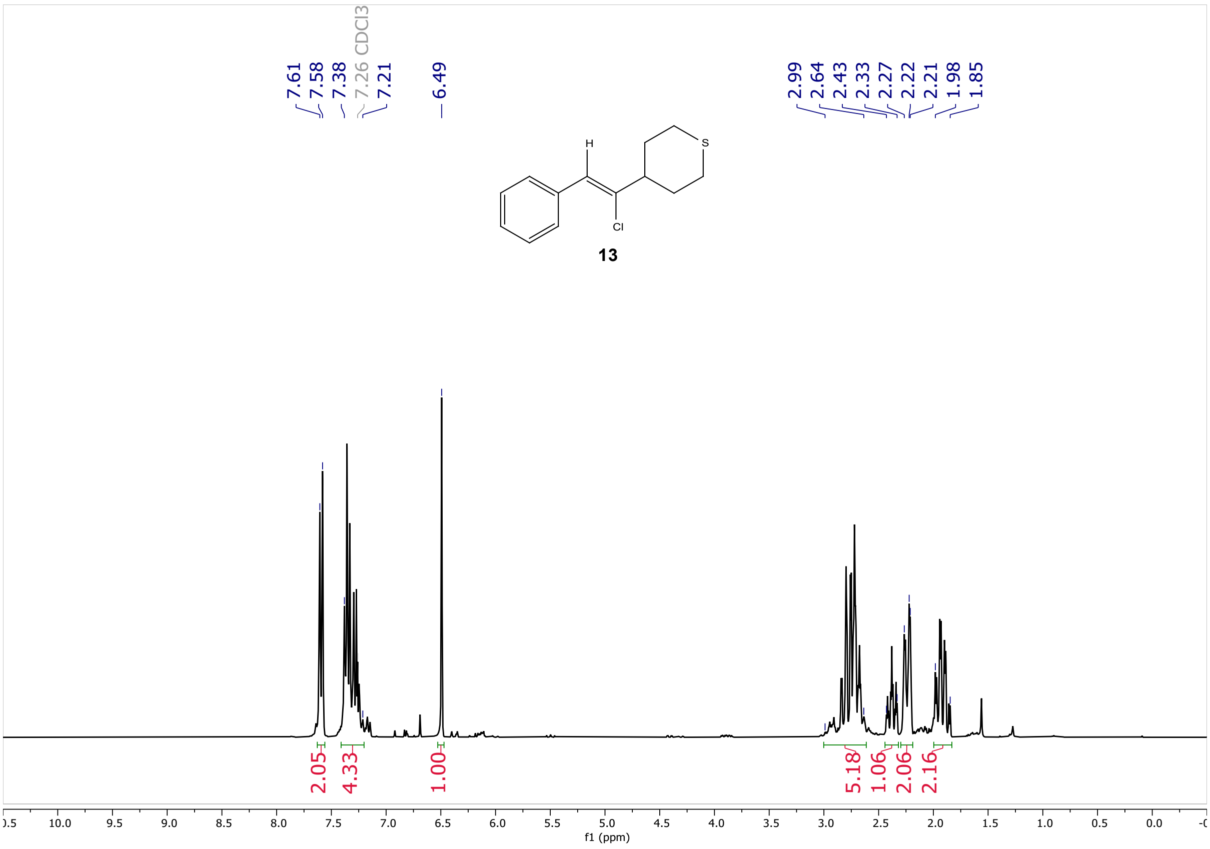

^{1}H-NMR (300 MHz, $CDCl_3$) of **13**, *Z* isomer.

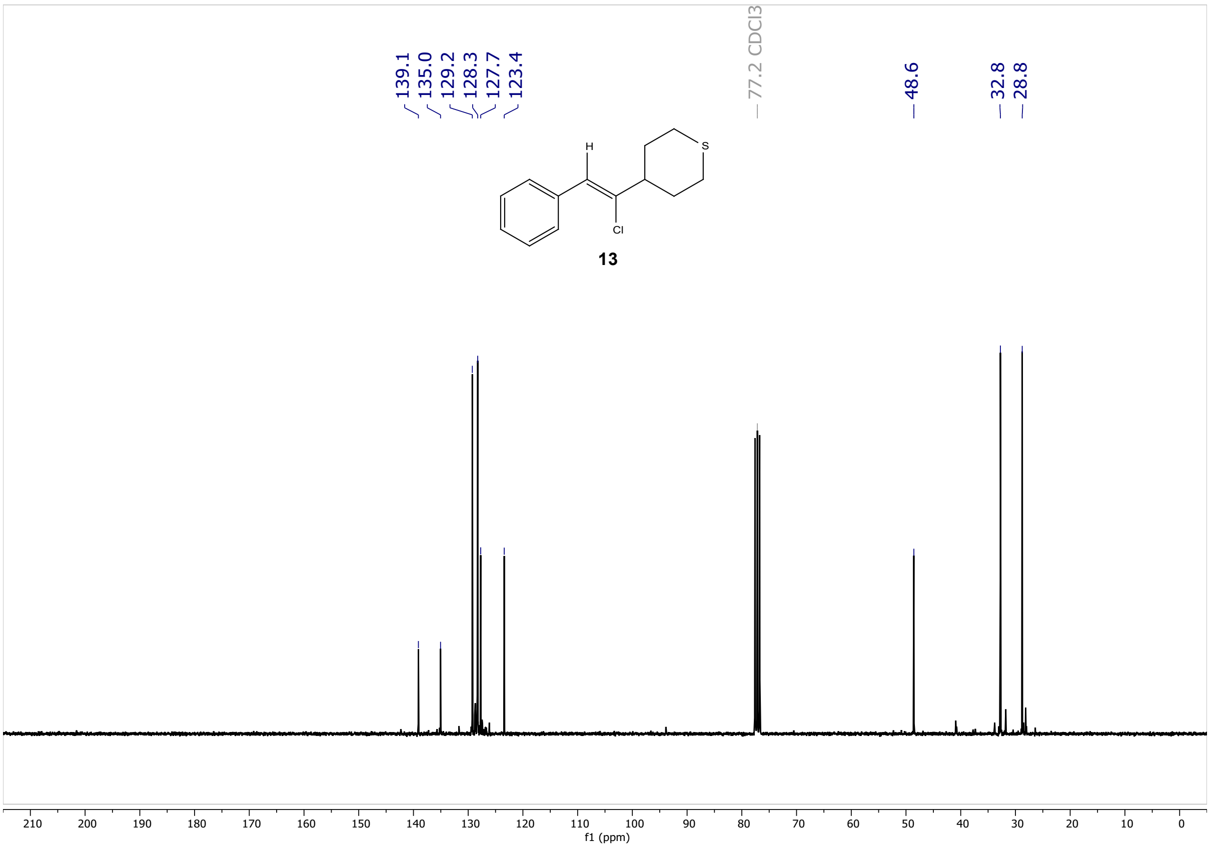

^{13}C-NMR (75 MHz, $CDCl_3$) of **13**, *Z* isomer.

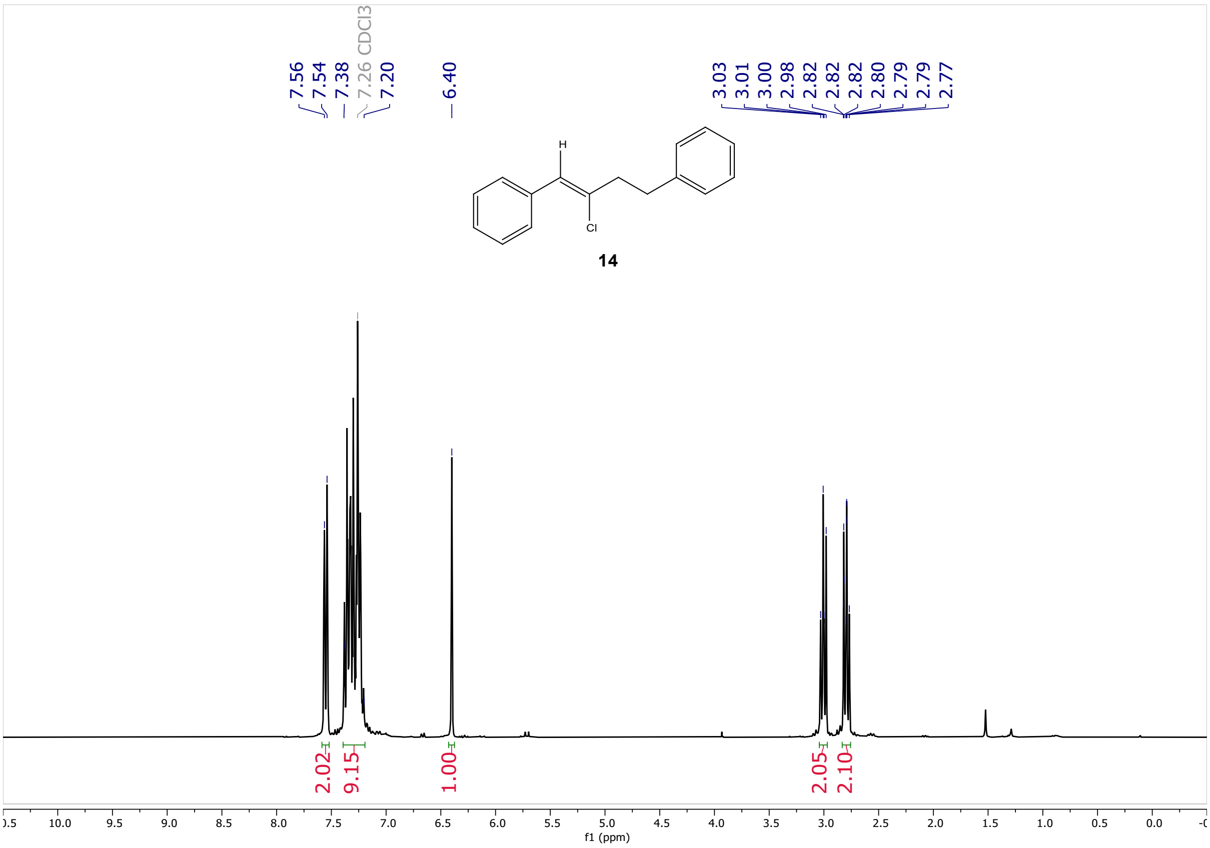

^{1}H-NMR (300 MHz, $CDCl_3$) of **14**, *Z* isomer.

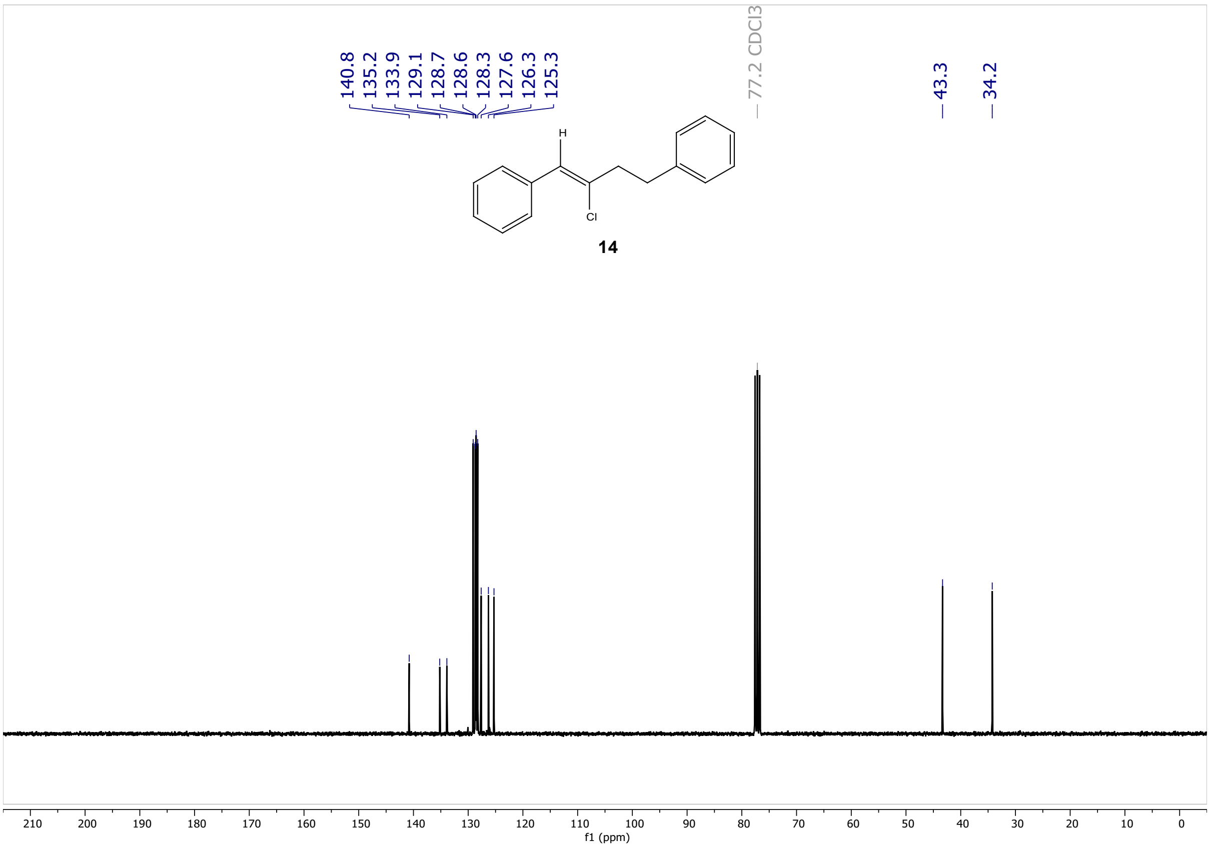

^{13}C-NMR (75 MHz, $CDCl_3$) of **14**, *Z* isomer.

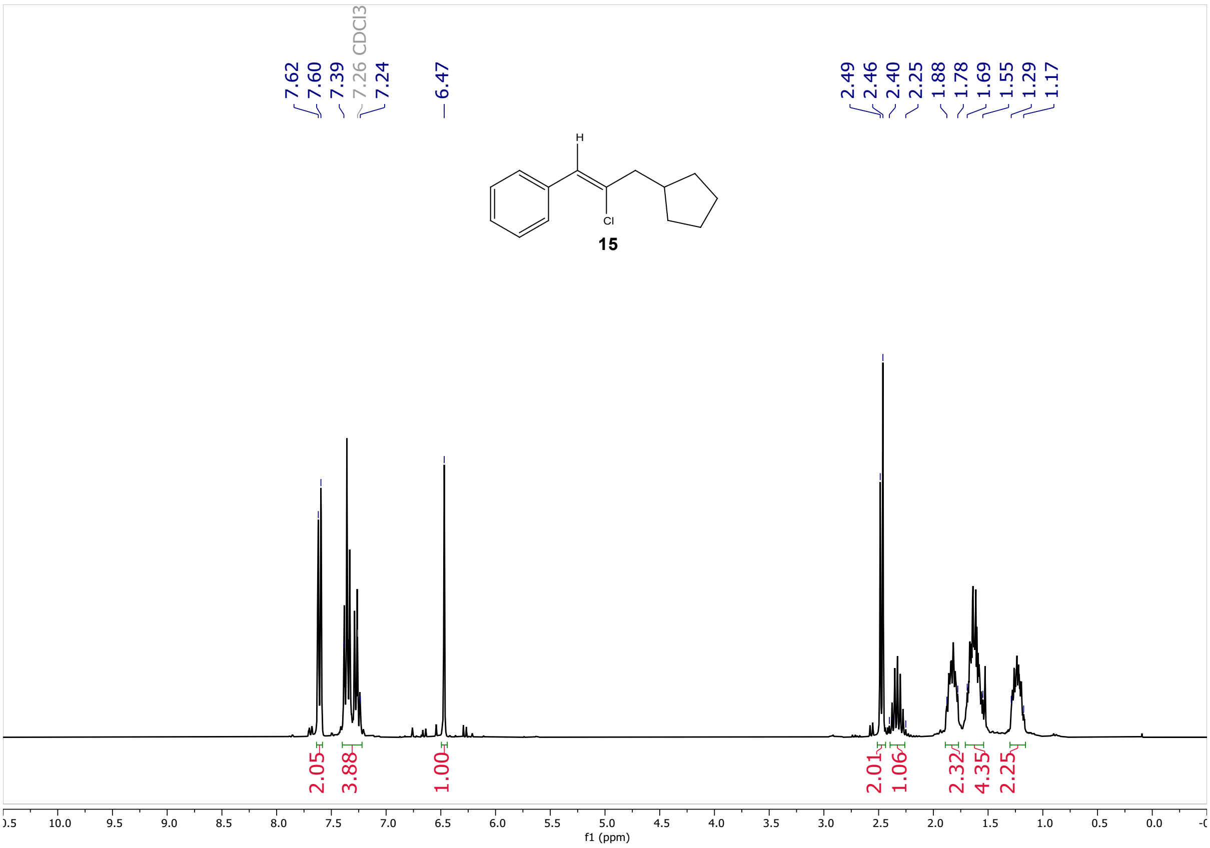

^{1}H-NMR (300 MHz, $CDCl_3$) of **15**, *Z* isomer.

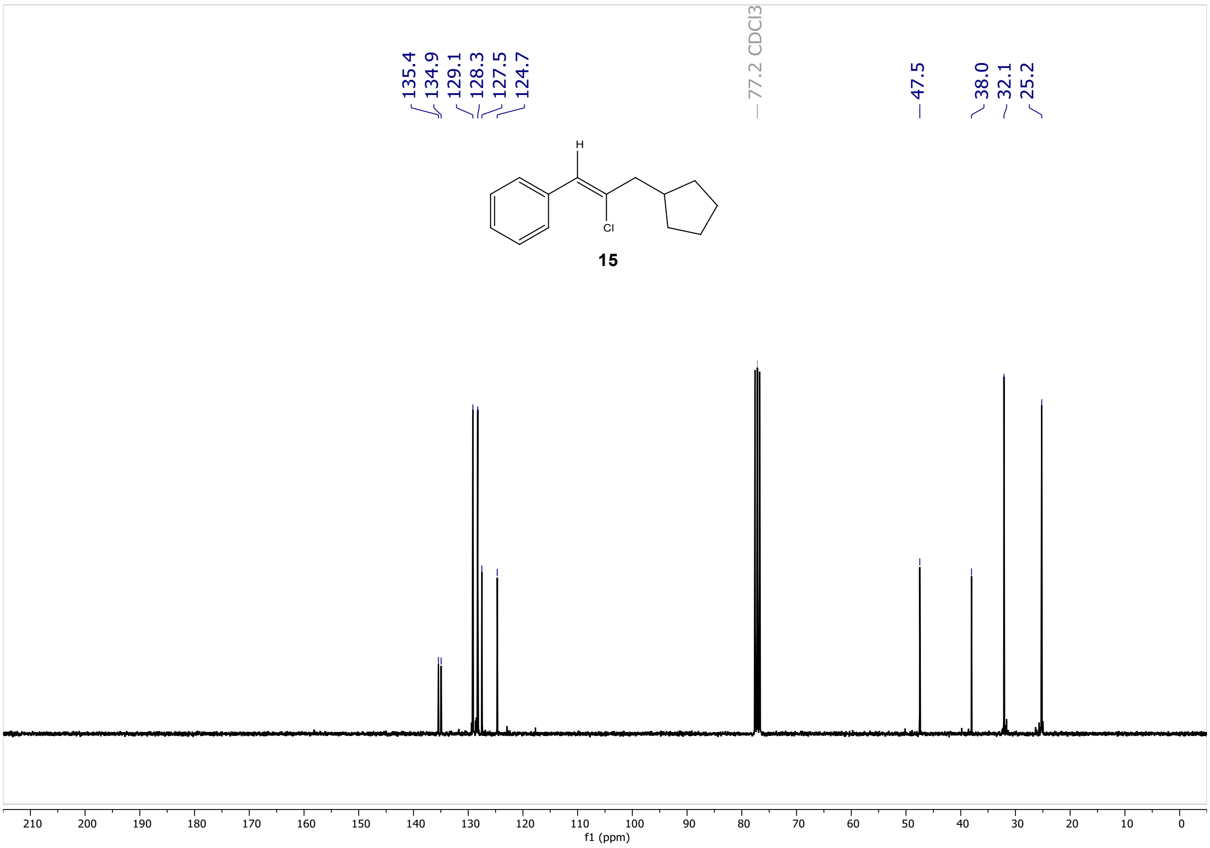

^{13}C-NMR (75 MHz, $CDCl_3$) of **15**, *Z* isomer.

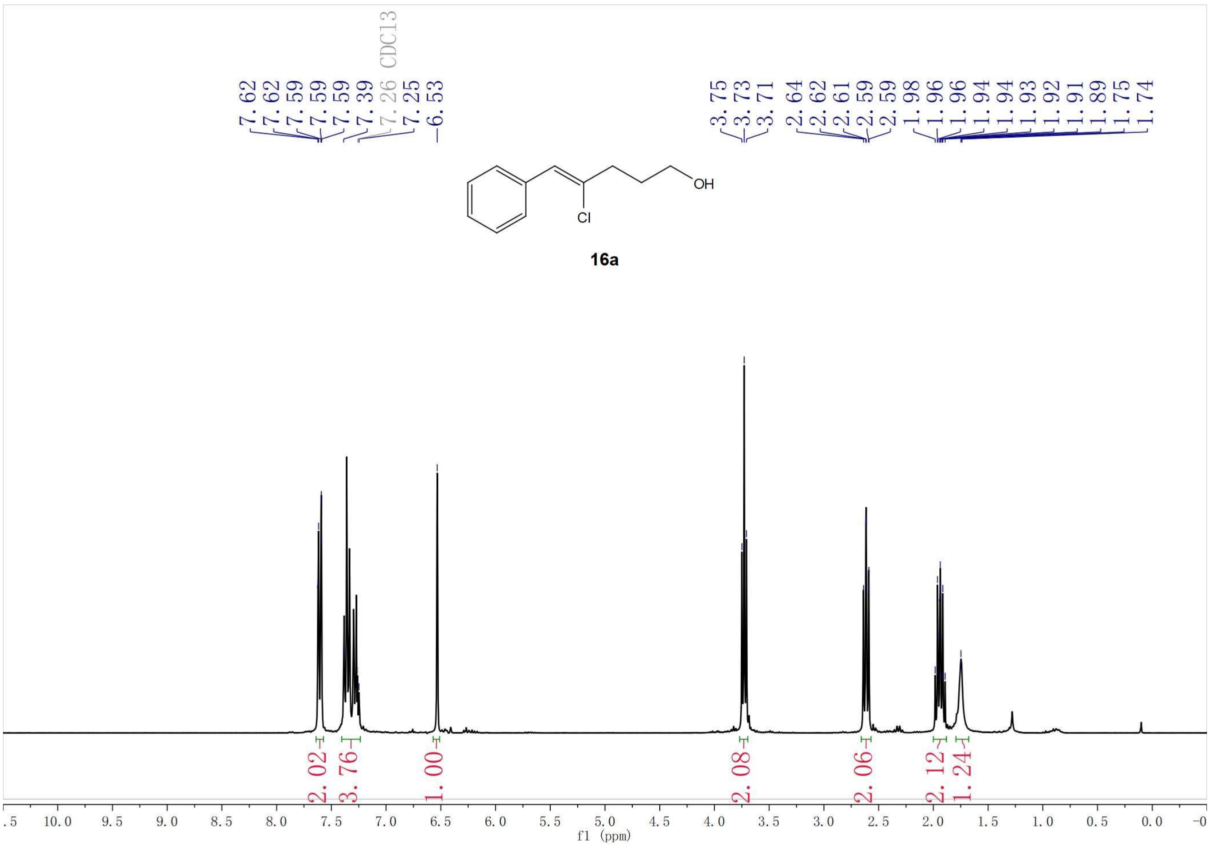

^{1}H-NMR (300 MHz, $CDCl_3$) of **16a**, *Z* isomer.

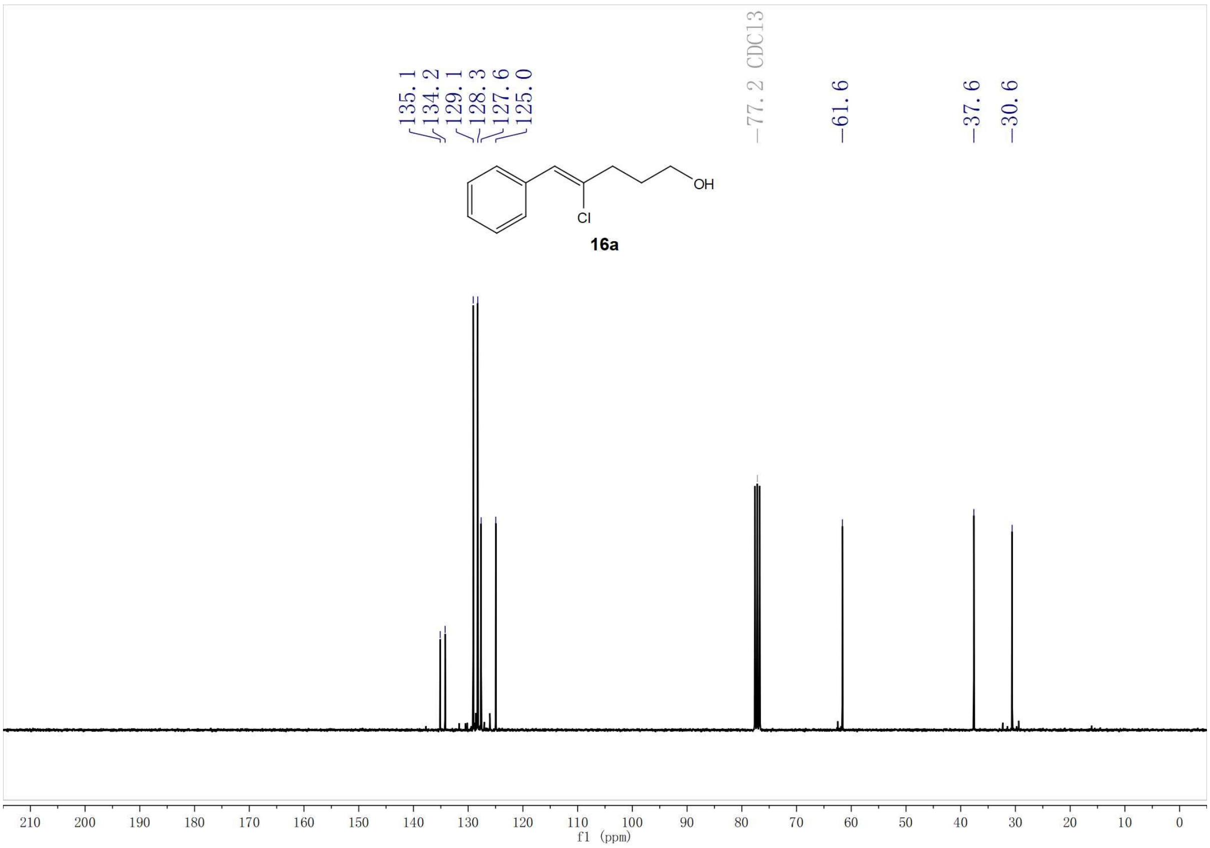

^{13}C-NMR (75 MHz, $CDCl_3$) of **16a**, *Z* isomer.

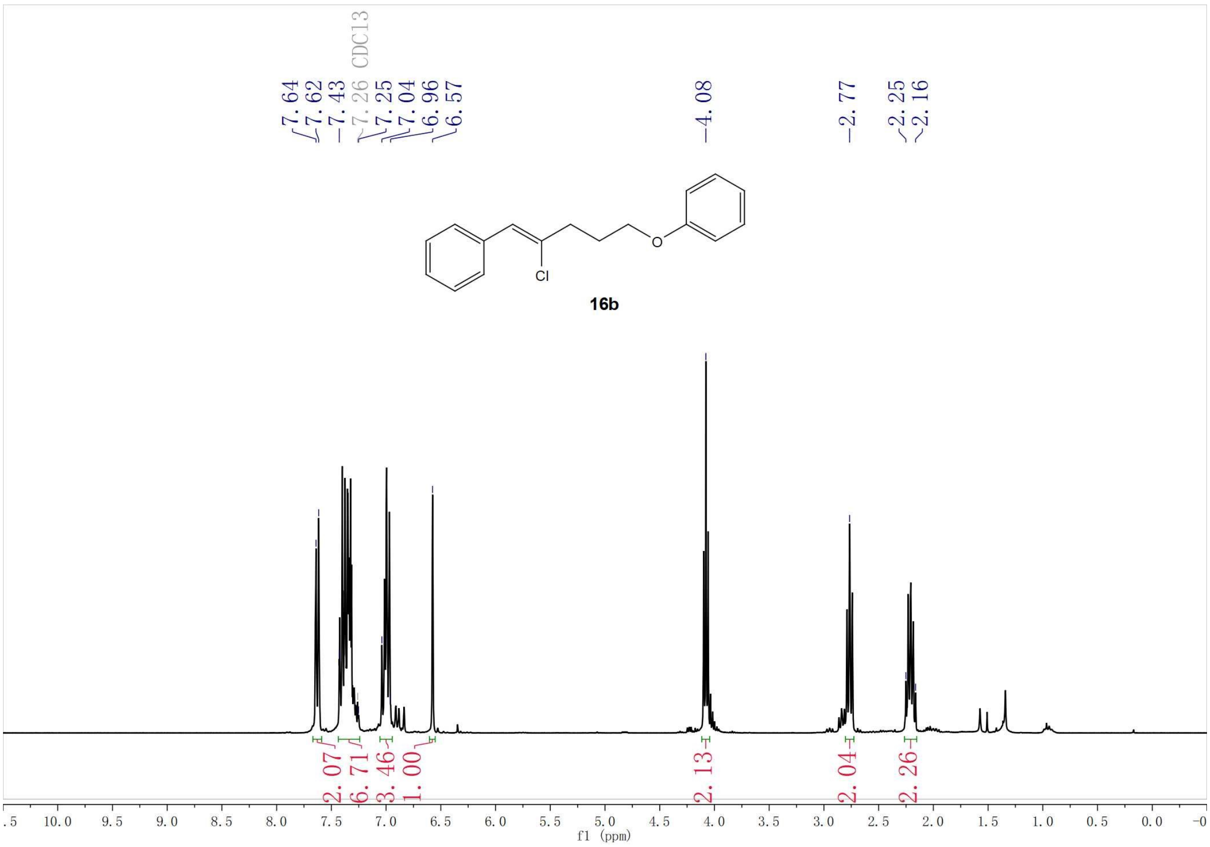

^{1}H-NMR (300 MHz, $CDCl_3$) of **16b**, *Z* isomer.

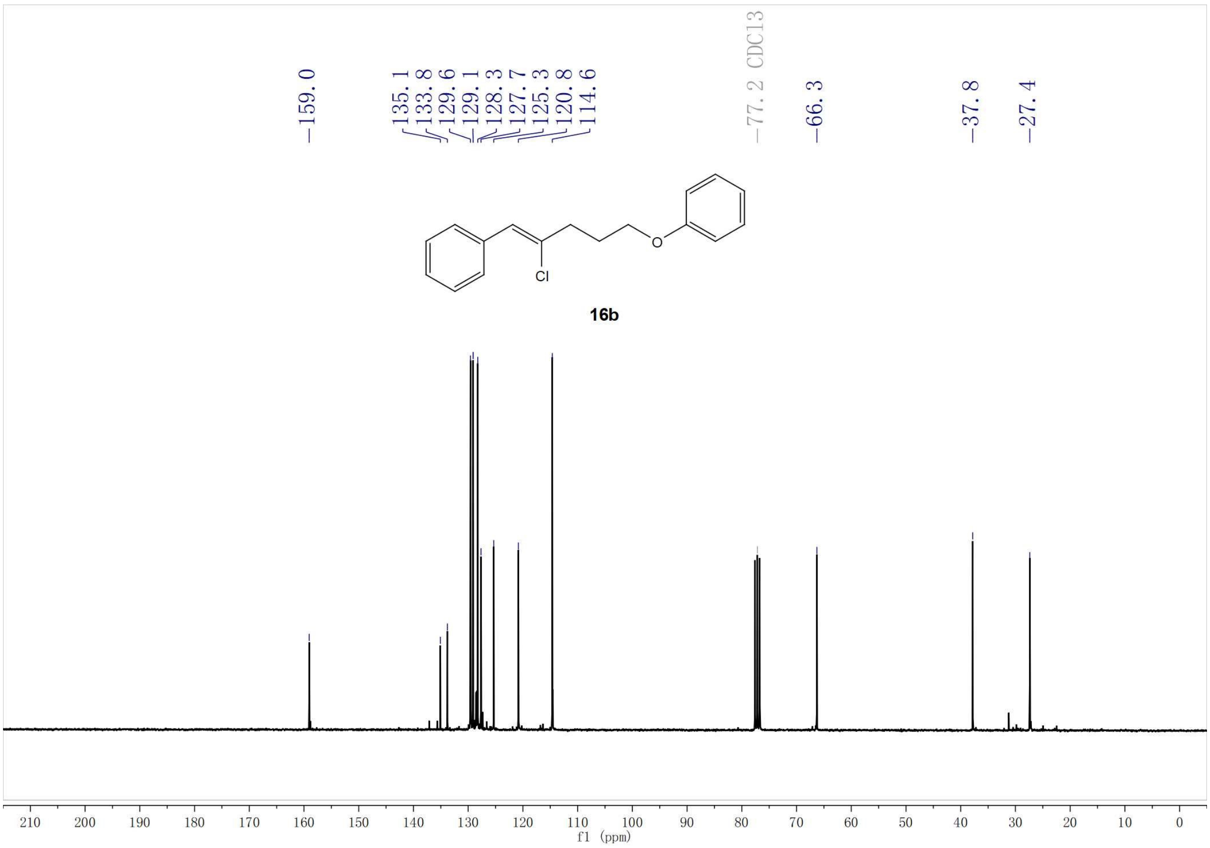

^{13}C-NMR (75 MHz, $CDCl_3$) of **16b**, *Z* isomer.

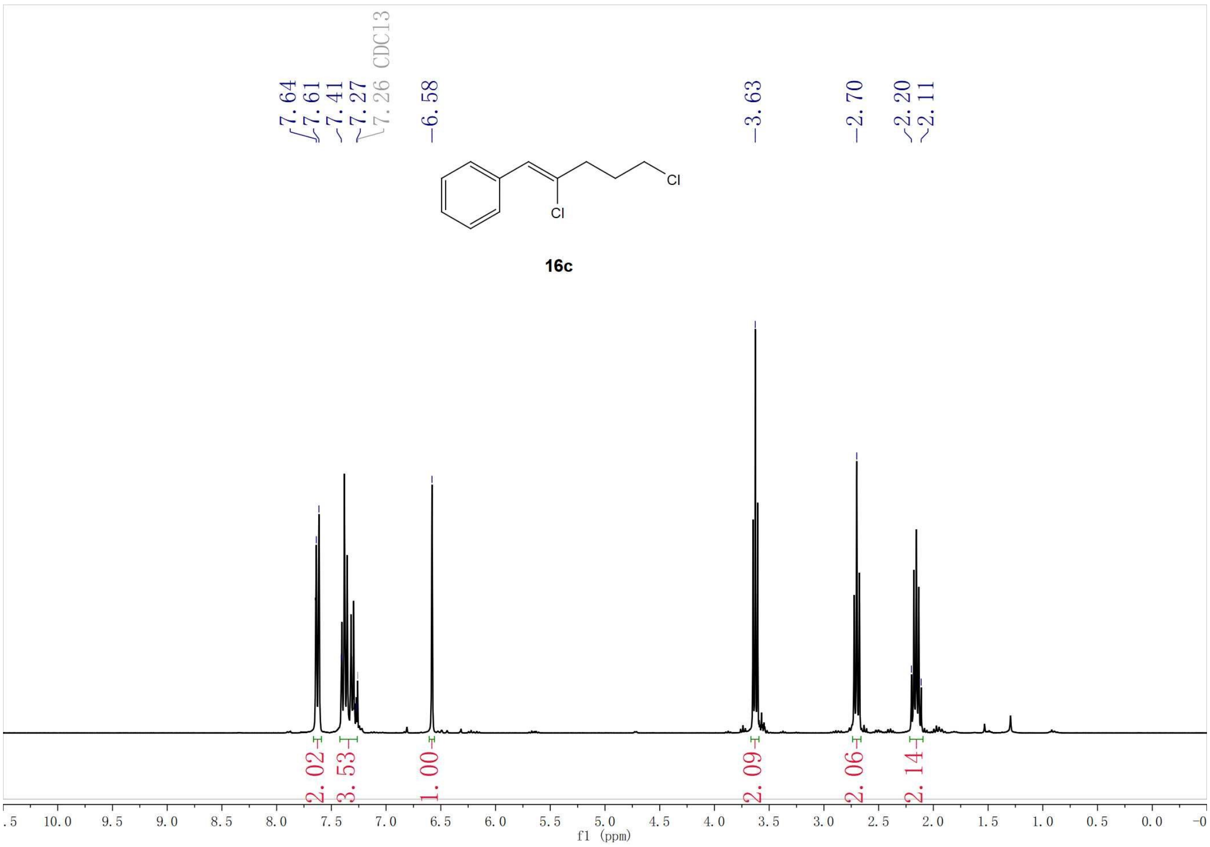

^{1}H-NMR (300 MHz, $CDCl_3$) of **16c**, *Z* isomer.

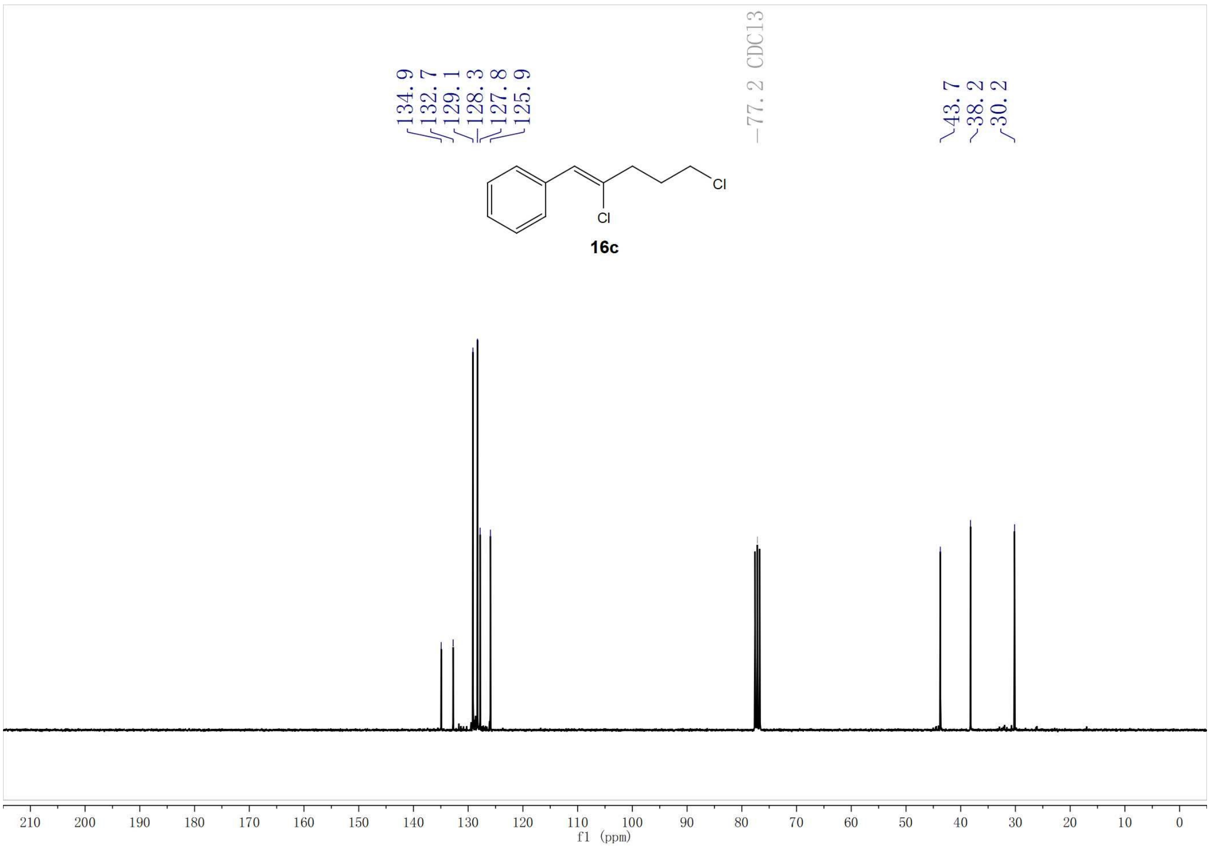

^{13}C-NMR (75 MHz, $CDCl_3$) of **16c**, *Z* isomer.

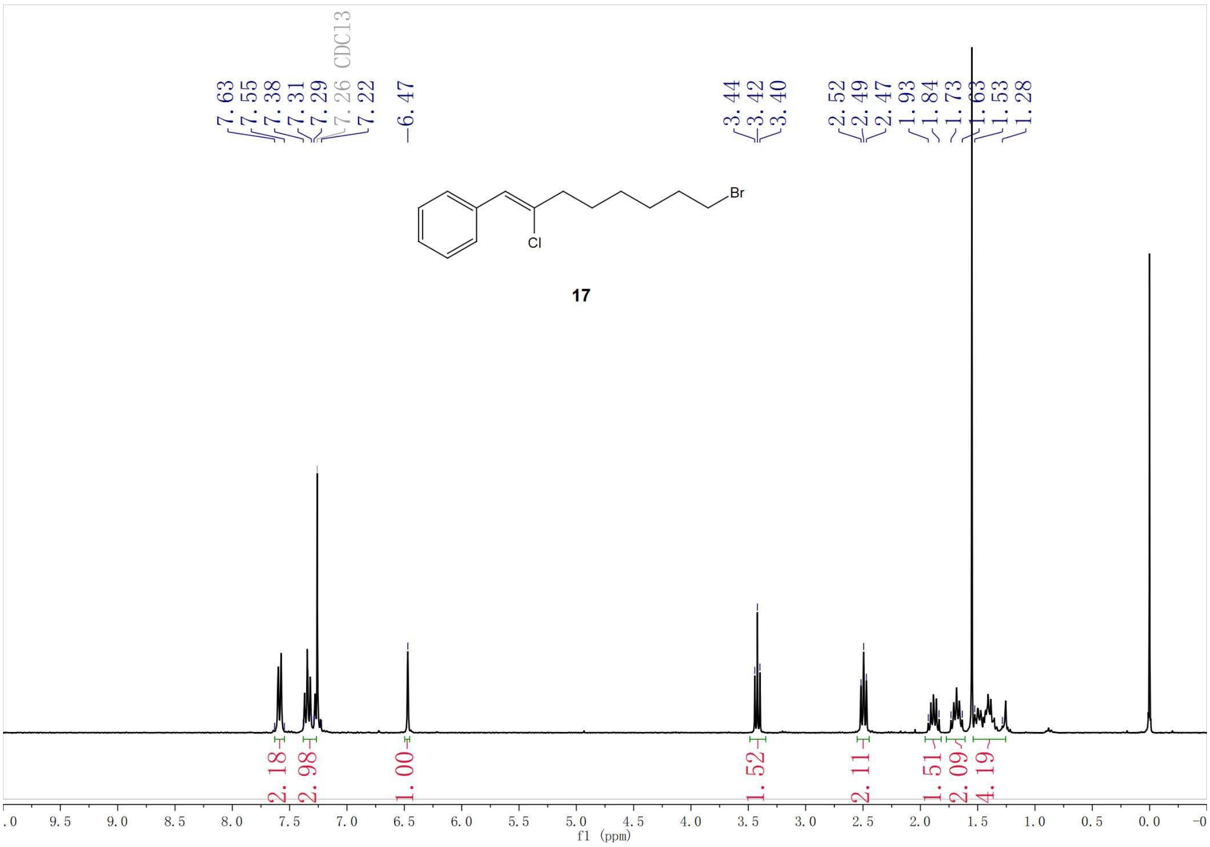

^{1}H-NMR (300 MHz, $CDCl_3$) of **17**, *Z* isomer.

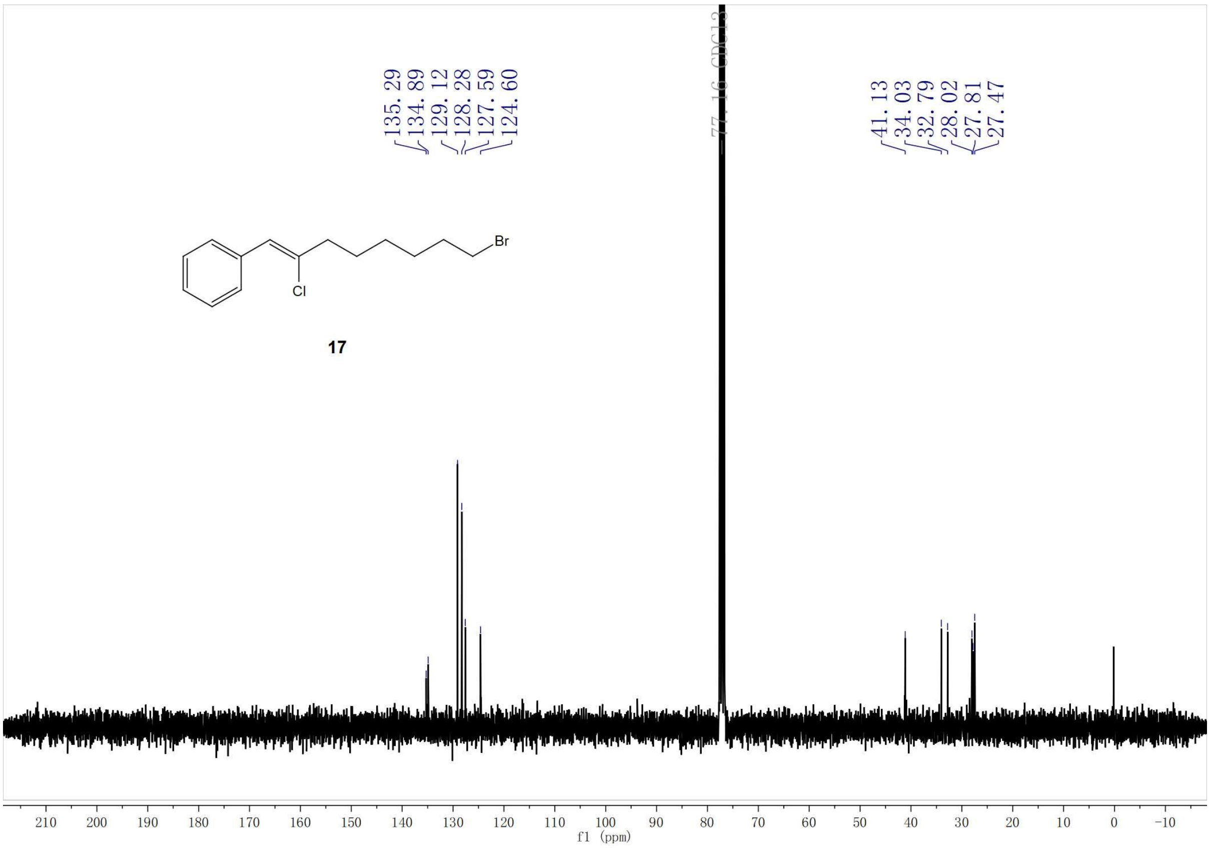

^{13}C-NMR (75 MHz, $CDCl_3$) of **17**, *Z* isomer.

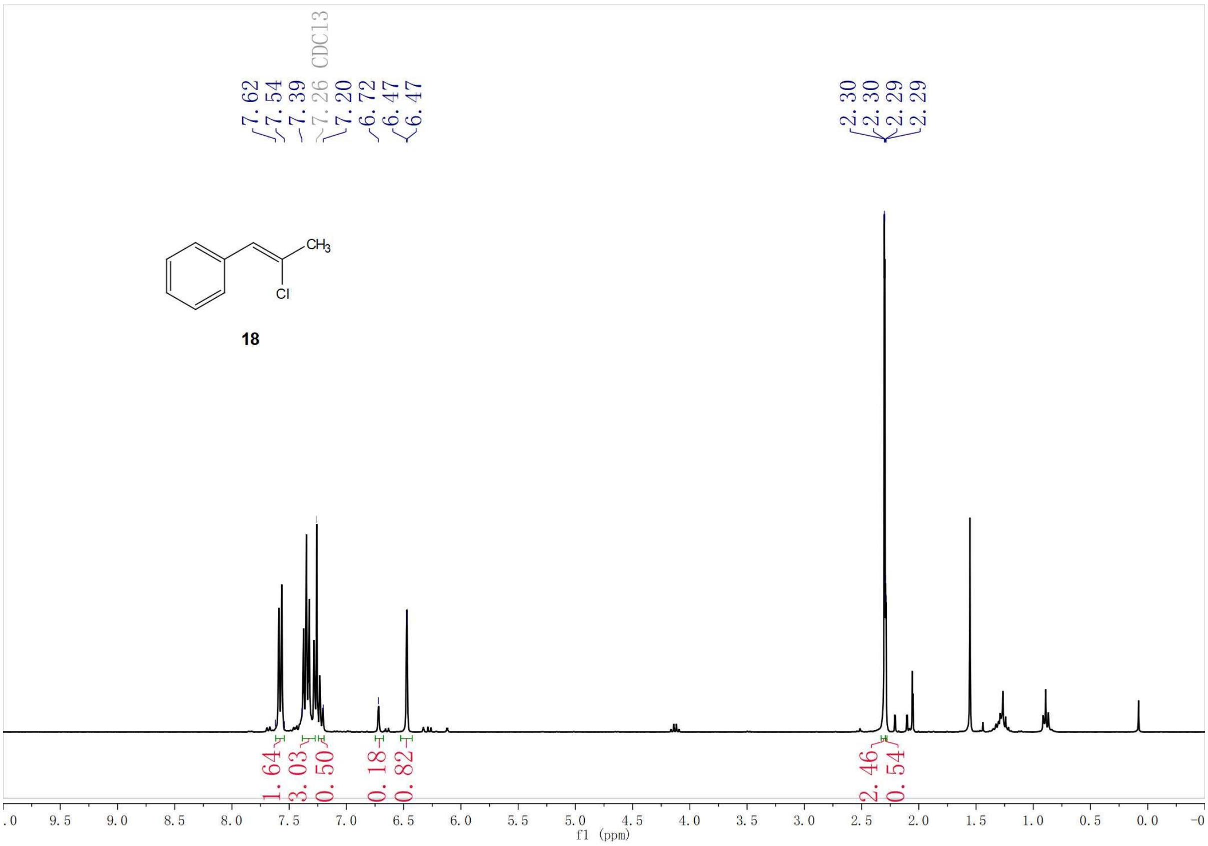

^{1}H-NMR (300 MHz, $CDCl_3$) of **18**, *Z/E* mixture.

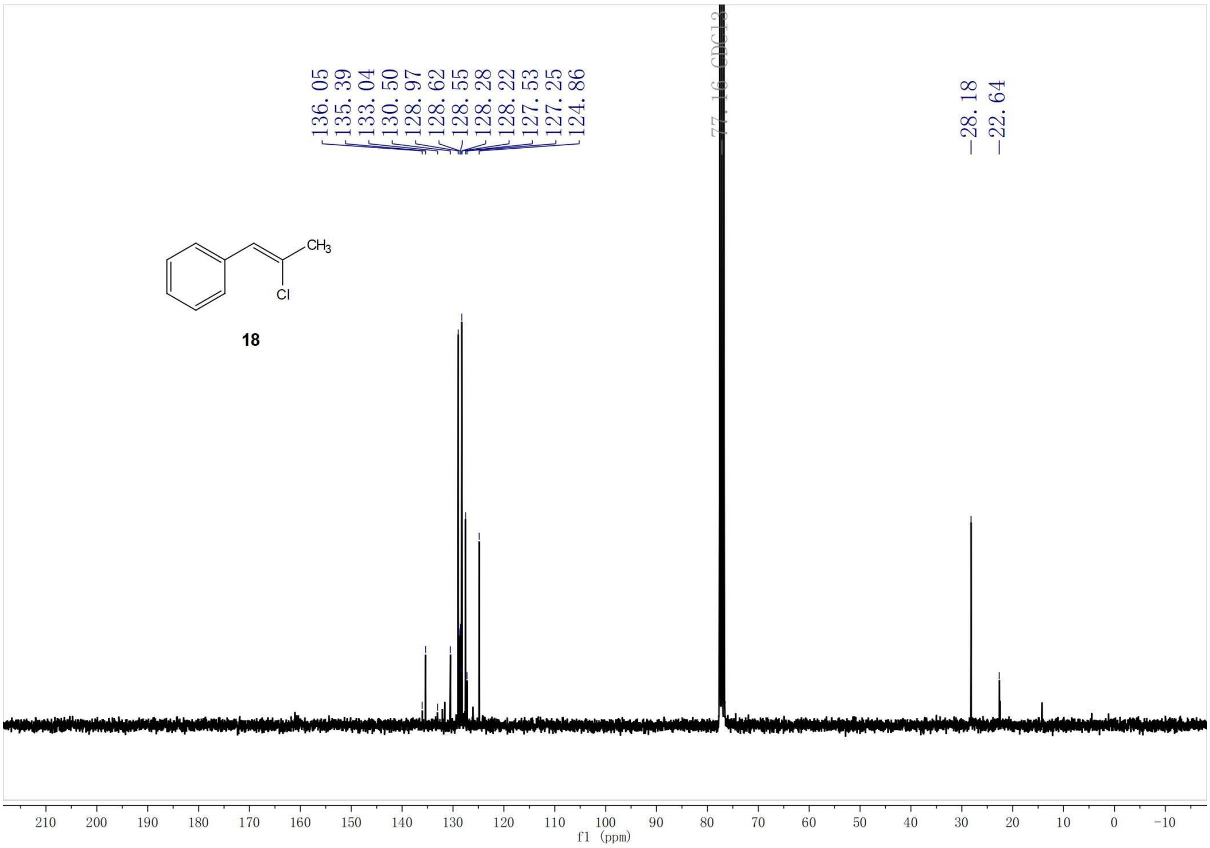

^{13}C-NMR (75 MHz, $CDCl_3$) of **18**, *Z/E* mixture.

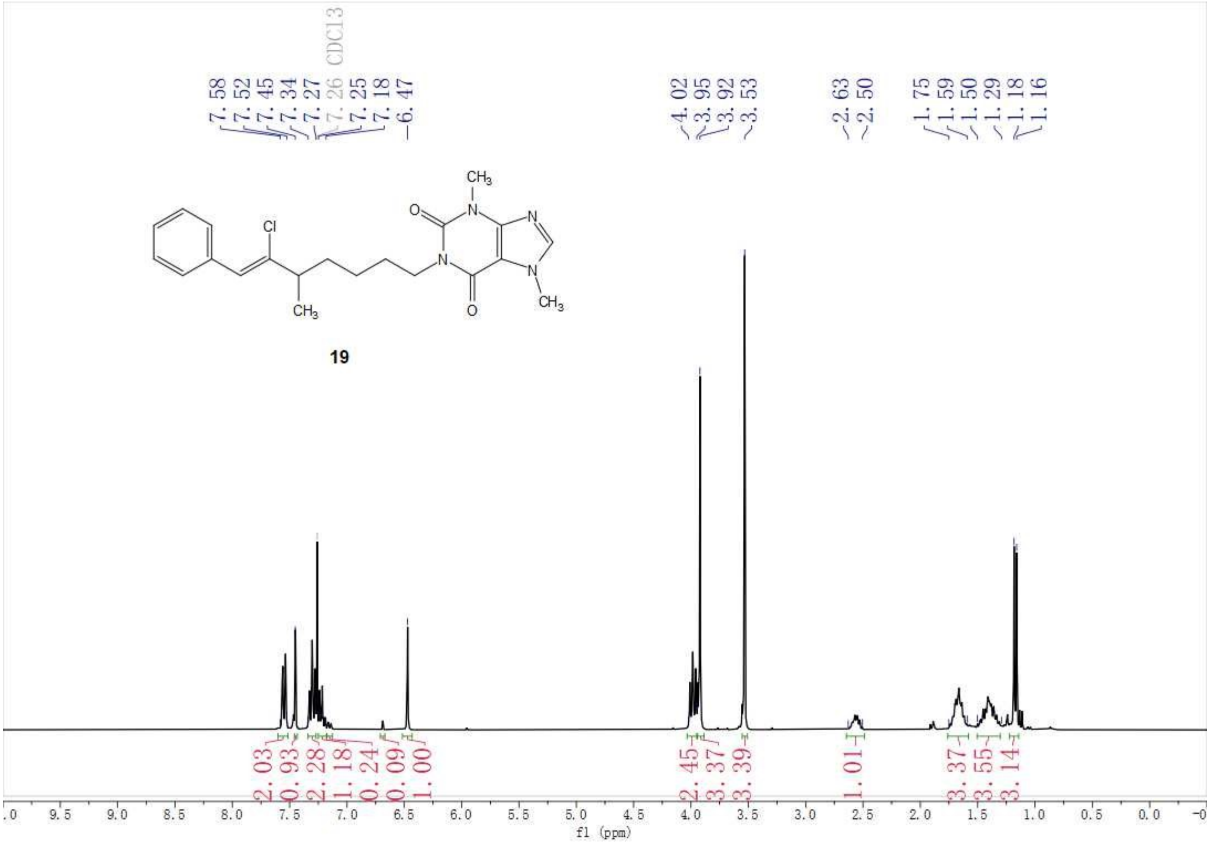

^{1}H-NMR (300 MHz, $CDCl_3$) of **19**, *Z* isomer.

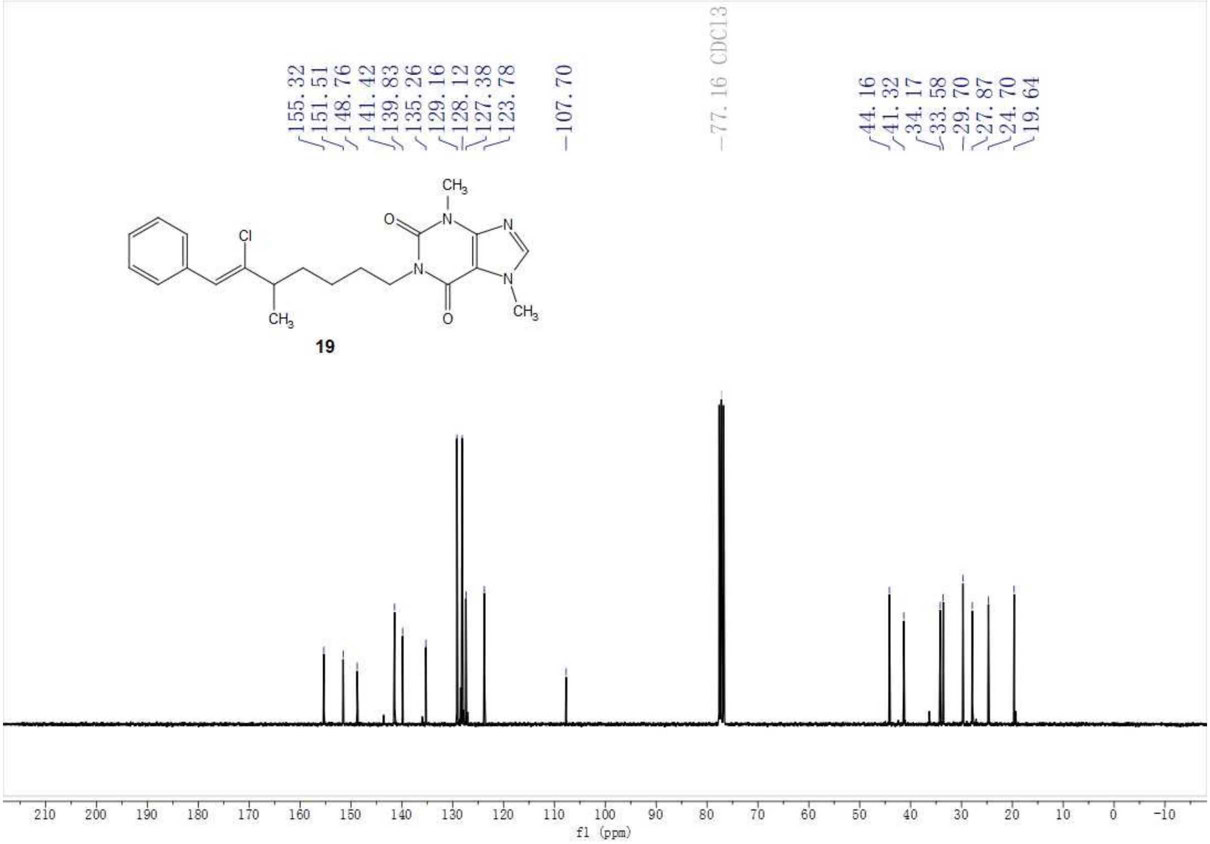

^{13}C-NMR (75 MHz, $CDCl_3$) of **19**, *Z* isomer.

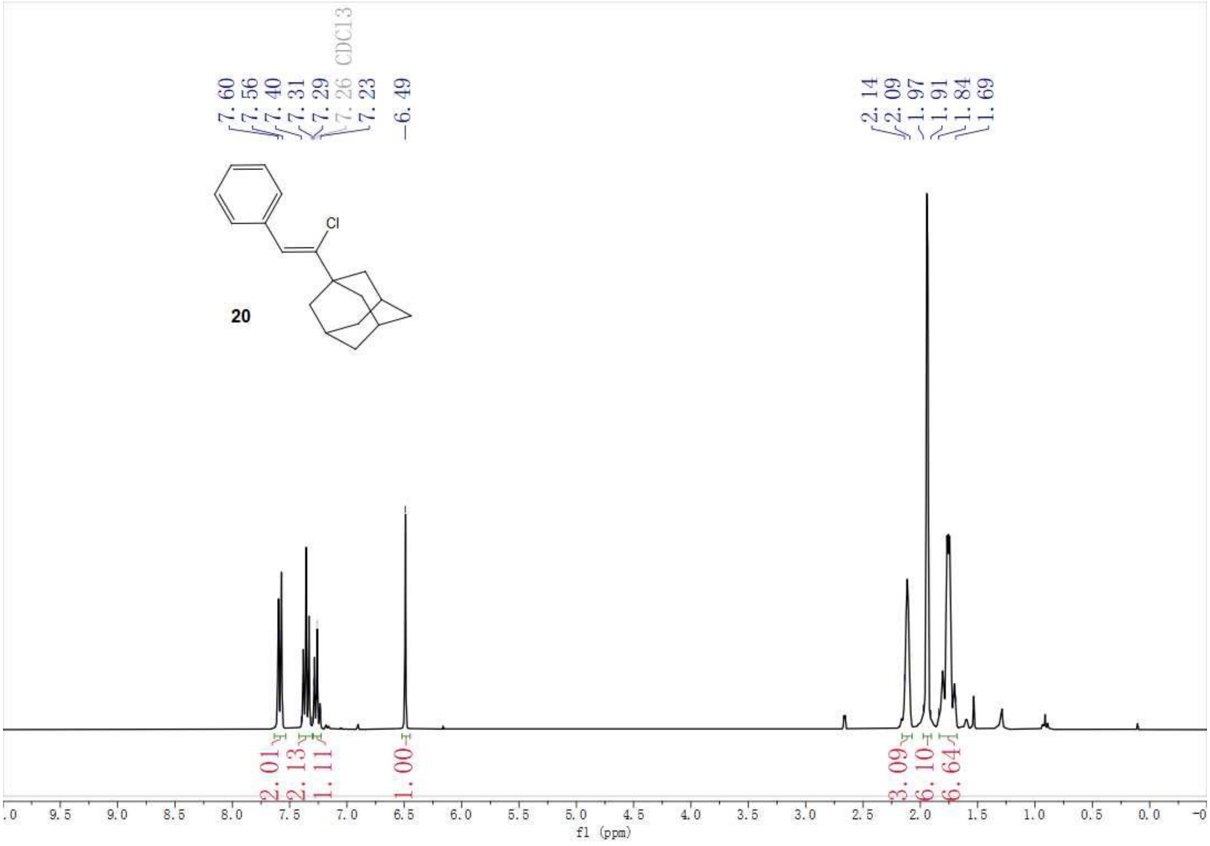

^{1}H-NMR (300 MHz, $CDCl_3$) of **20**, *Z* isomer.

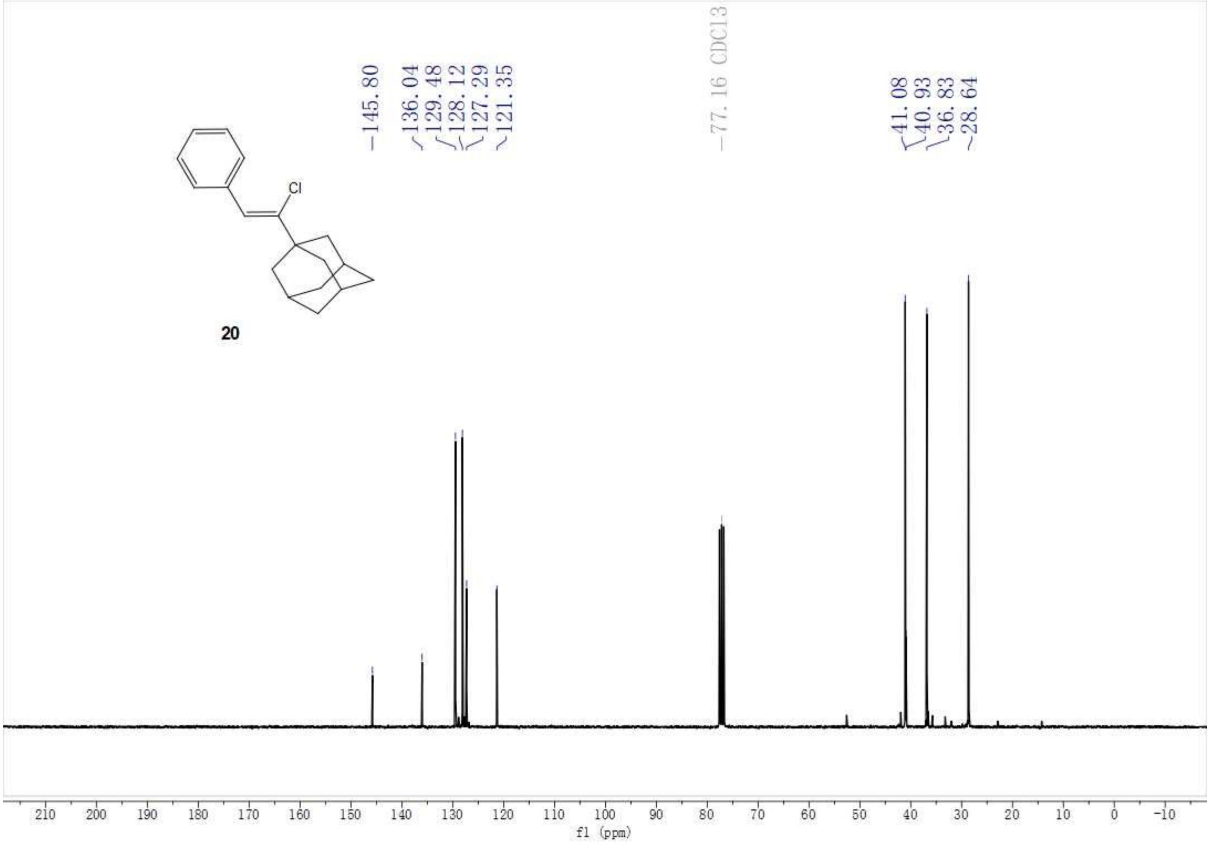

^{13}C-NMR (75 MHz, $CDCl_3$) of **20**, *Z* isomer.

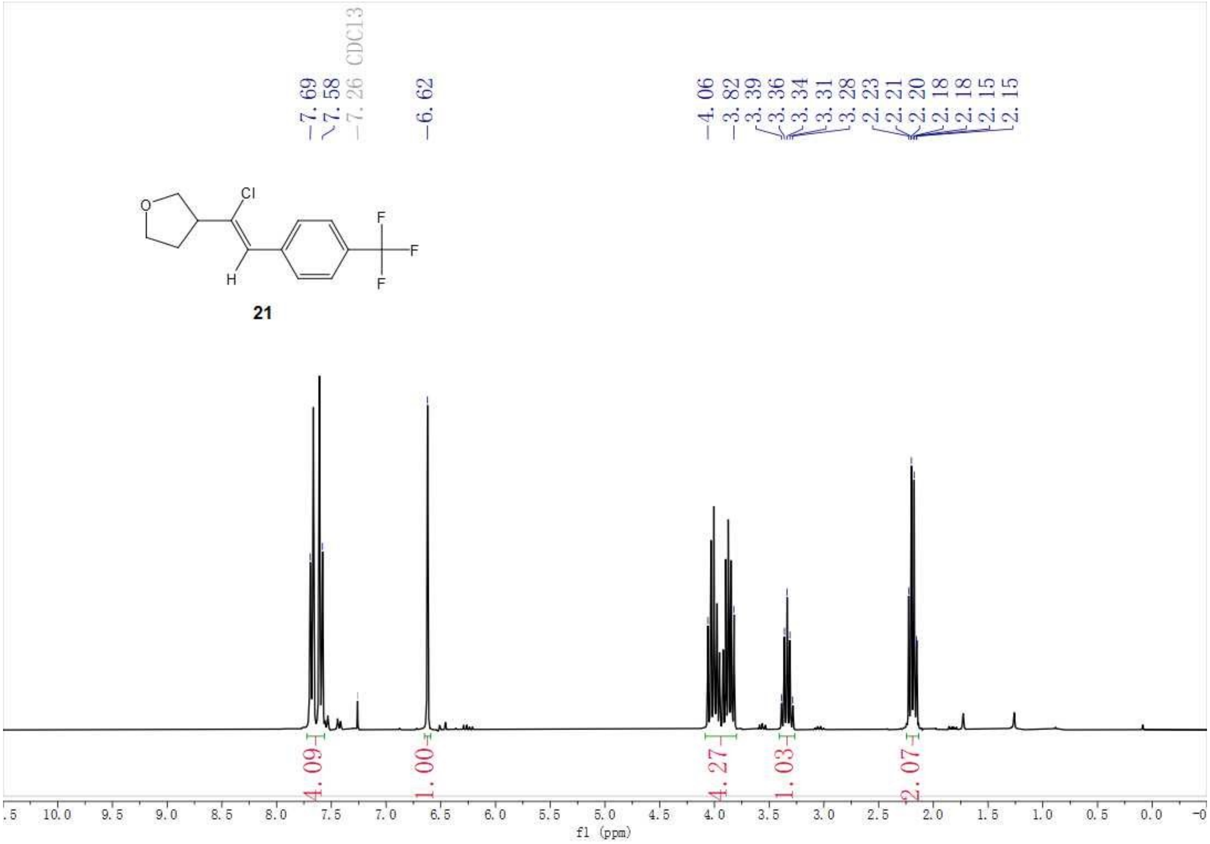

^{1}H-NMR (300 MHz, $CDCl_3$) of **21**, *Z* isomer.

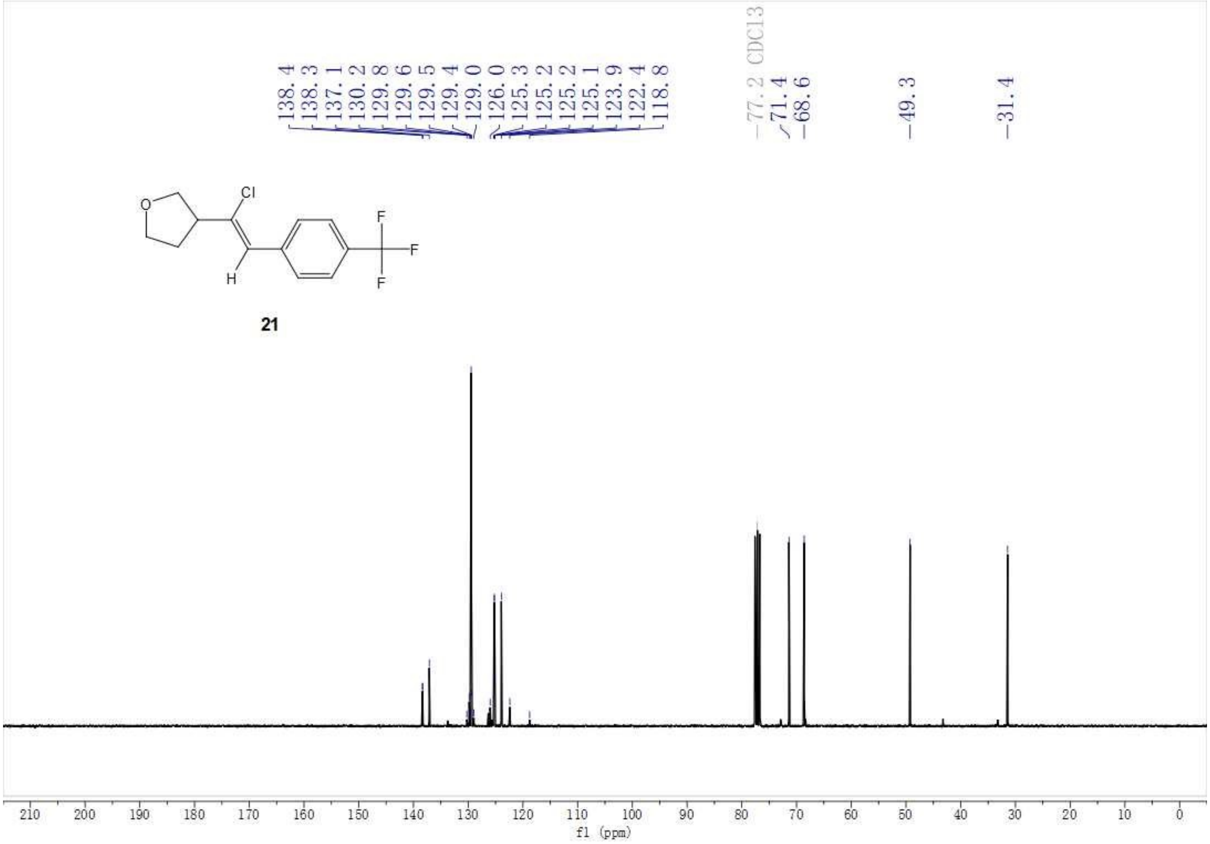

^{13}C-NMR (75 MHz, $CDCl_3$) of **21**, *Z* isomer.

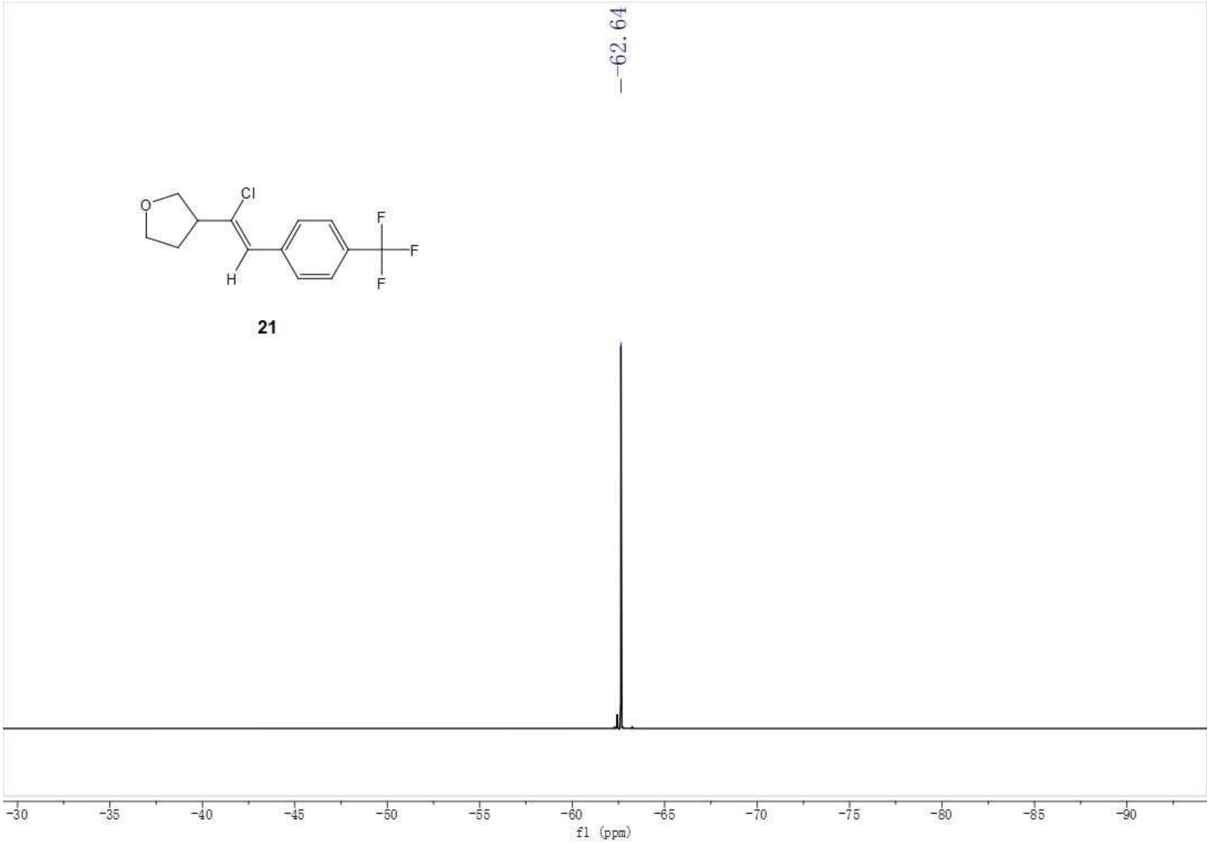

^{19}F-NMR (282 MHz, $CDCl_3$) of **21**, *Z* isomer.

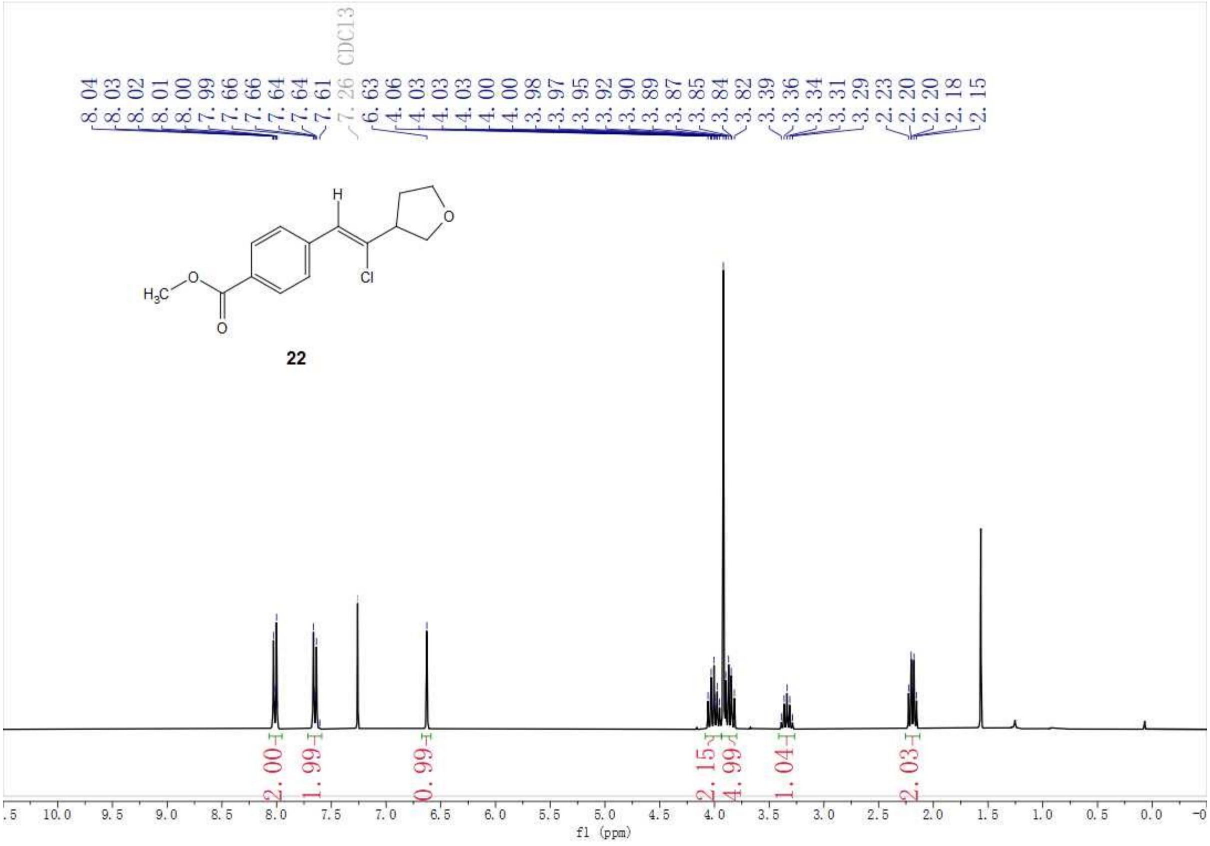

^{1}H-NMR (300 MHz, $CDCl_3$) of **22**, *Z* isomer.

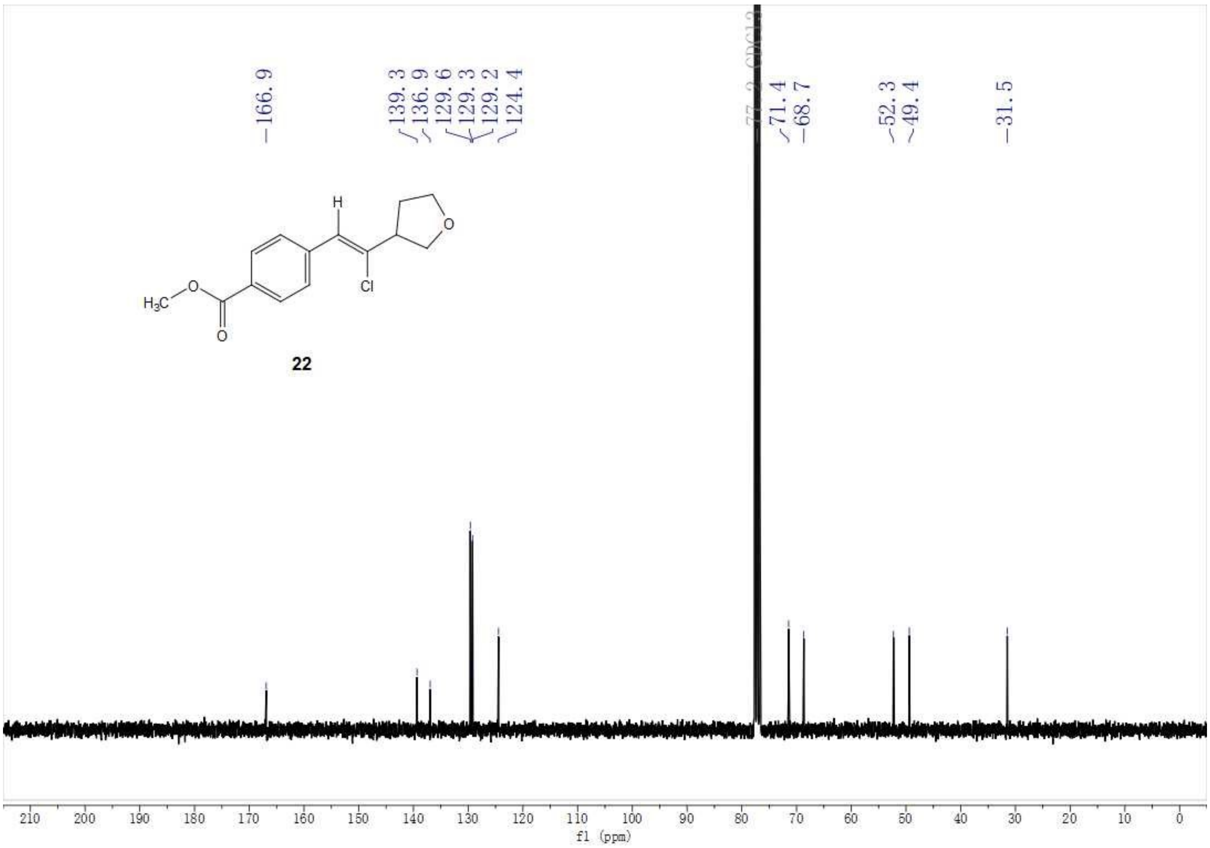

^{13}C-NMR (75 MHz, $CDCl_3$) of **22**, *Z* isomer.

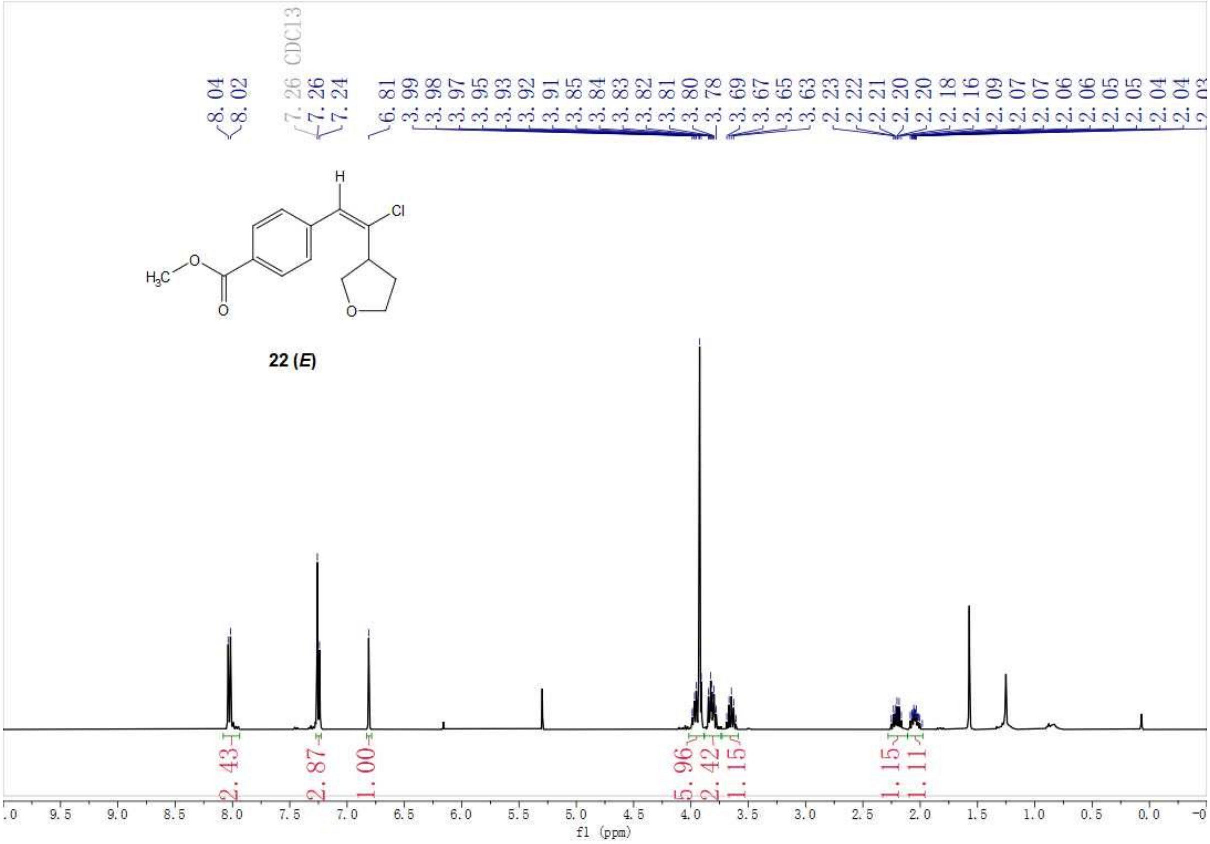

^{1}H-NMR (400 MHz, $CDCl_3$) of **22**, *E* isomer.

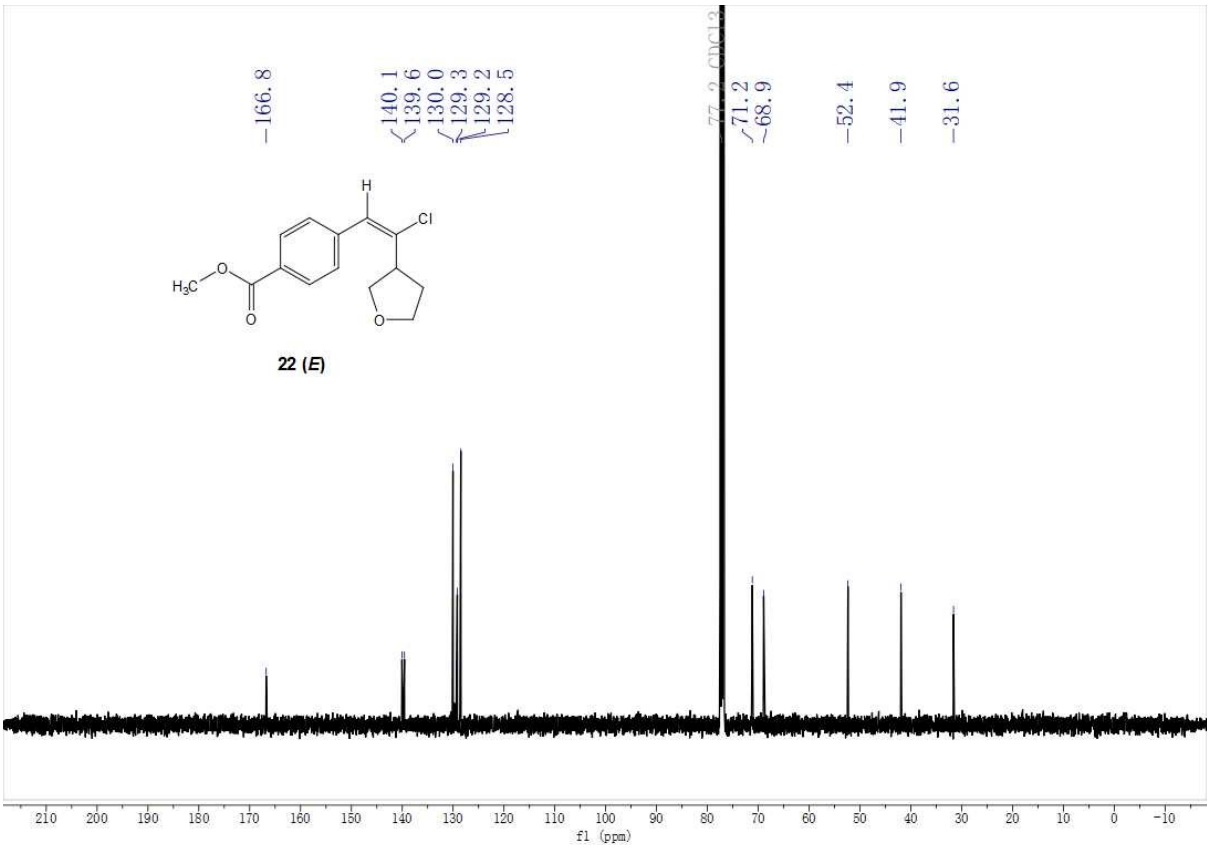

^{13}C-NMR (101 MHz, $CDCl_3$) of **22**, *E* isomer.

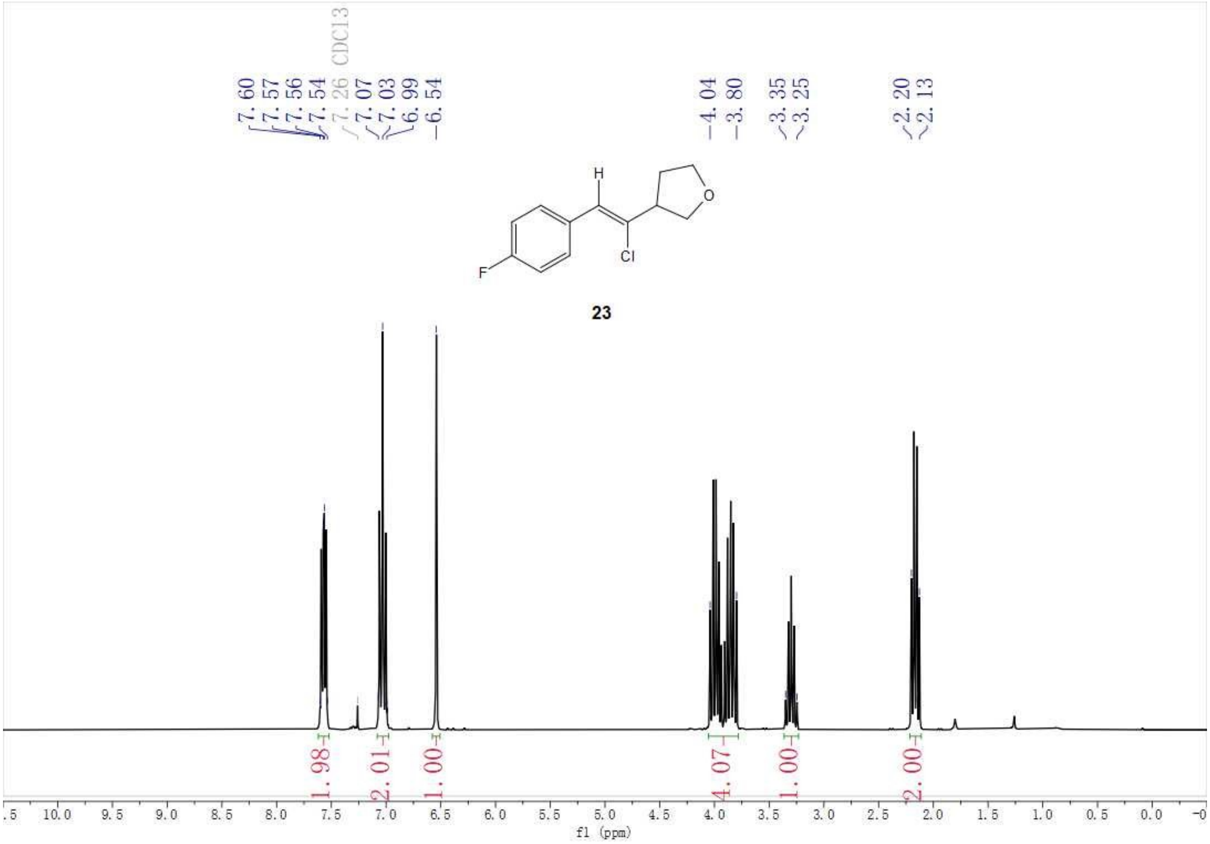

^{1}H-NMR (300 MHz, $CDCl_3$) of **23**, *Z* isomer.

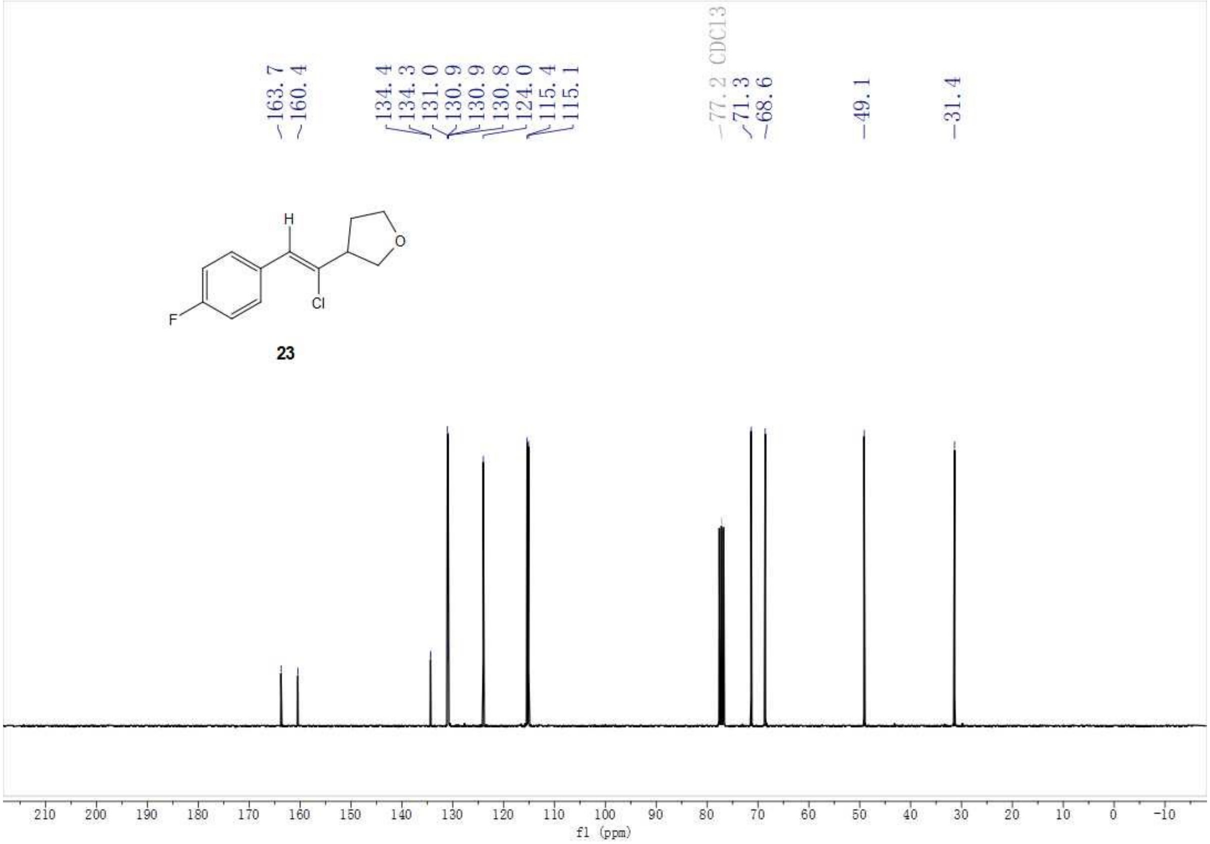

^{13}C-NMR (75 MHz, $CDCl_3$) of **23**, *Z* isomer.

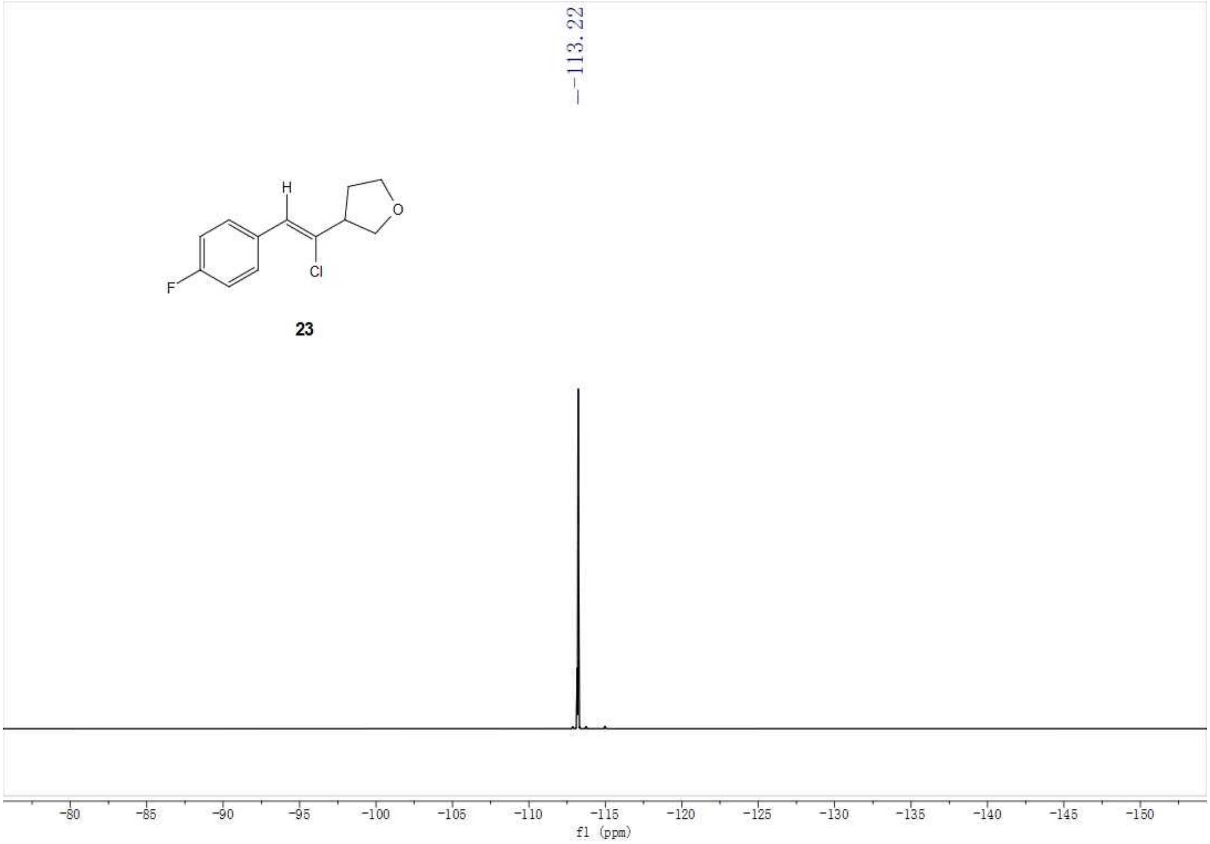

^{19}F-NMR (282 MHz, $CDCl_3$) of **23**, *Z* isomer.

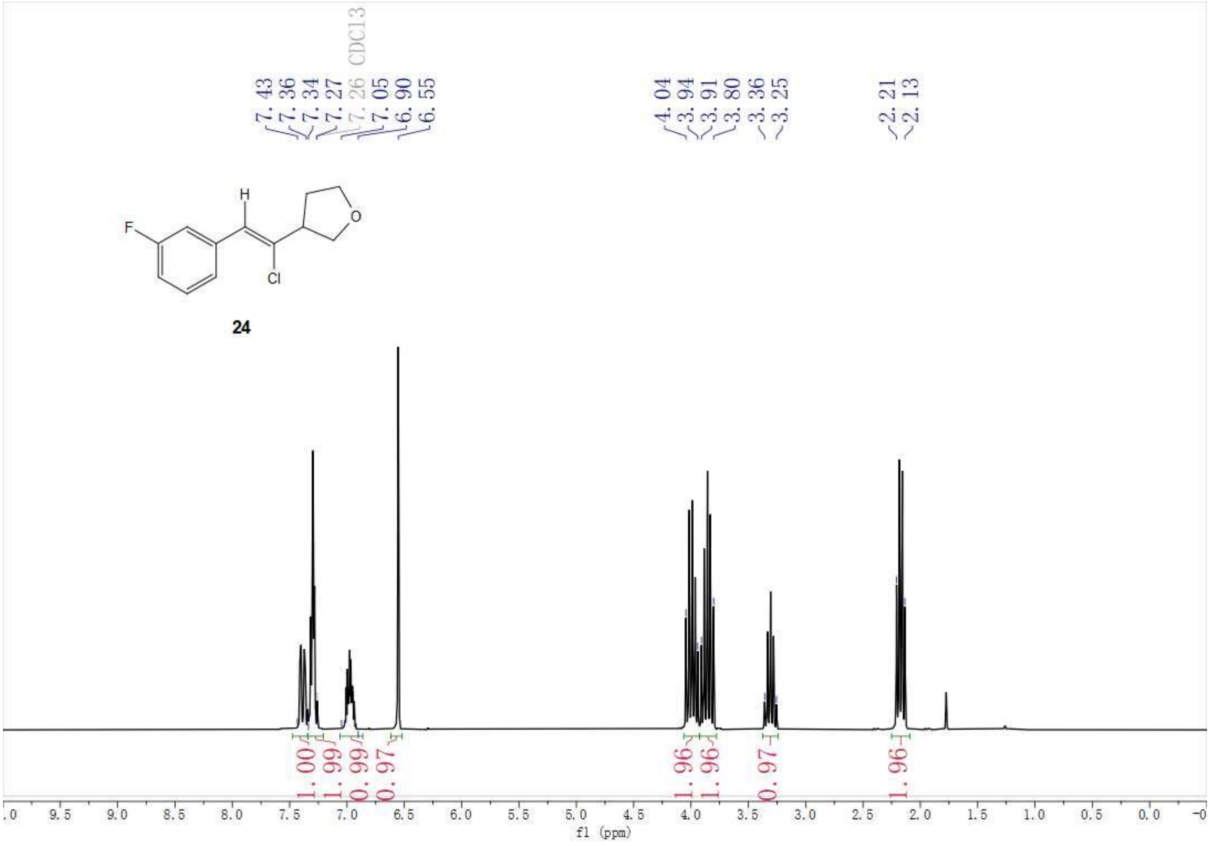

^{1}H-NMR (300 MHz, CDCl$_3$) of **24**, *Z* isomer.

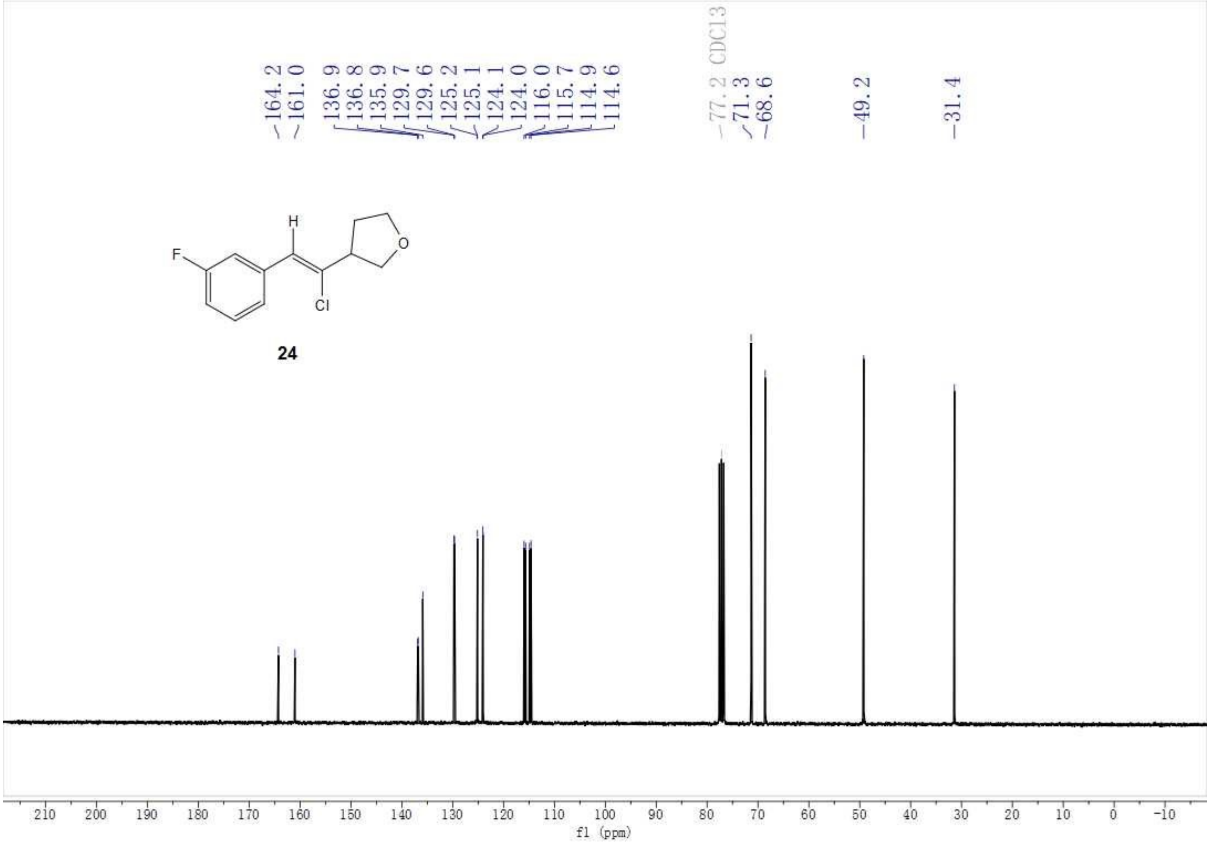

^{13}C-NMR (75 MHz, CDCl$_3$) of **24**, *Z* isomer.

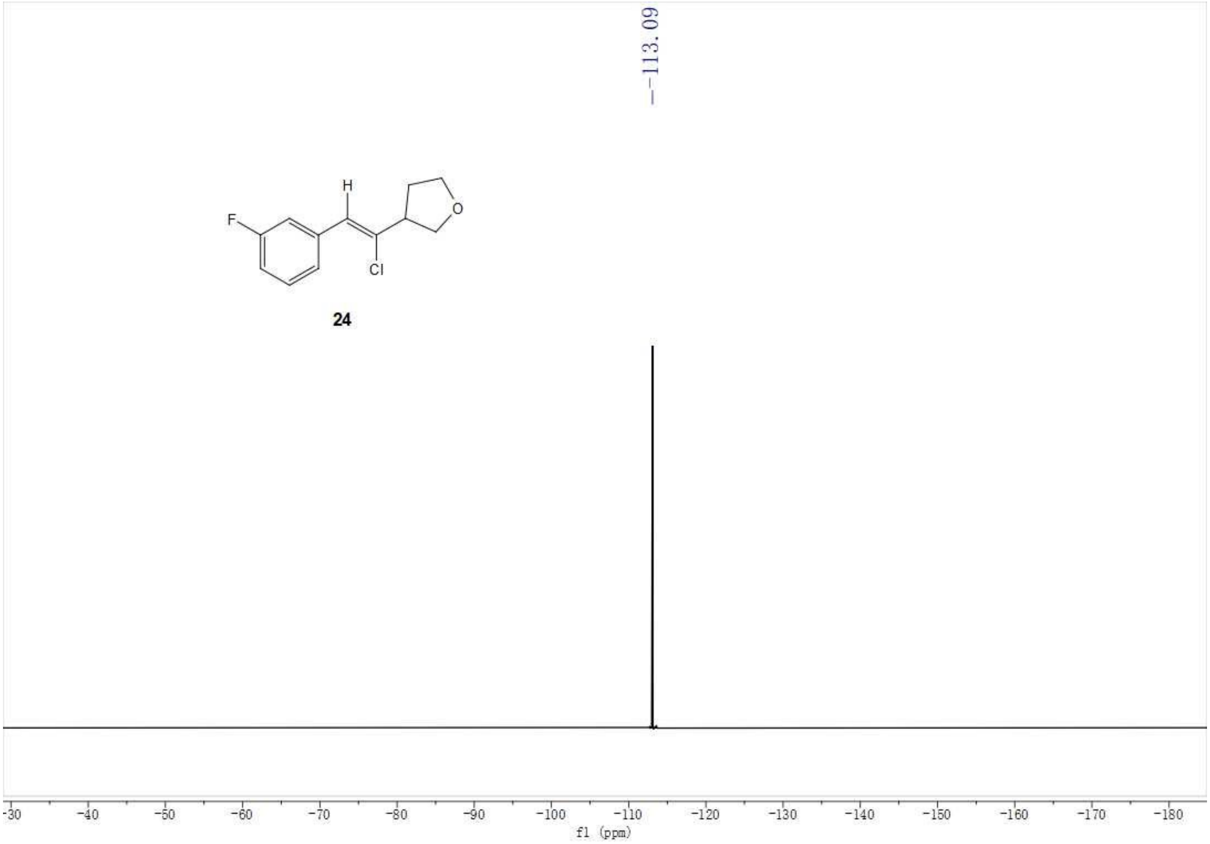

^{19}F-NMR (282 MHz, $CDCl_3$) of **24**, *Z* isomer.

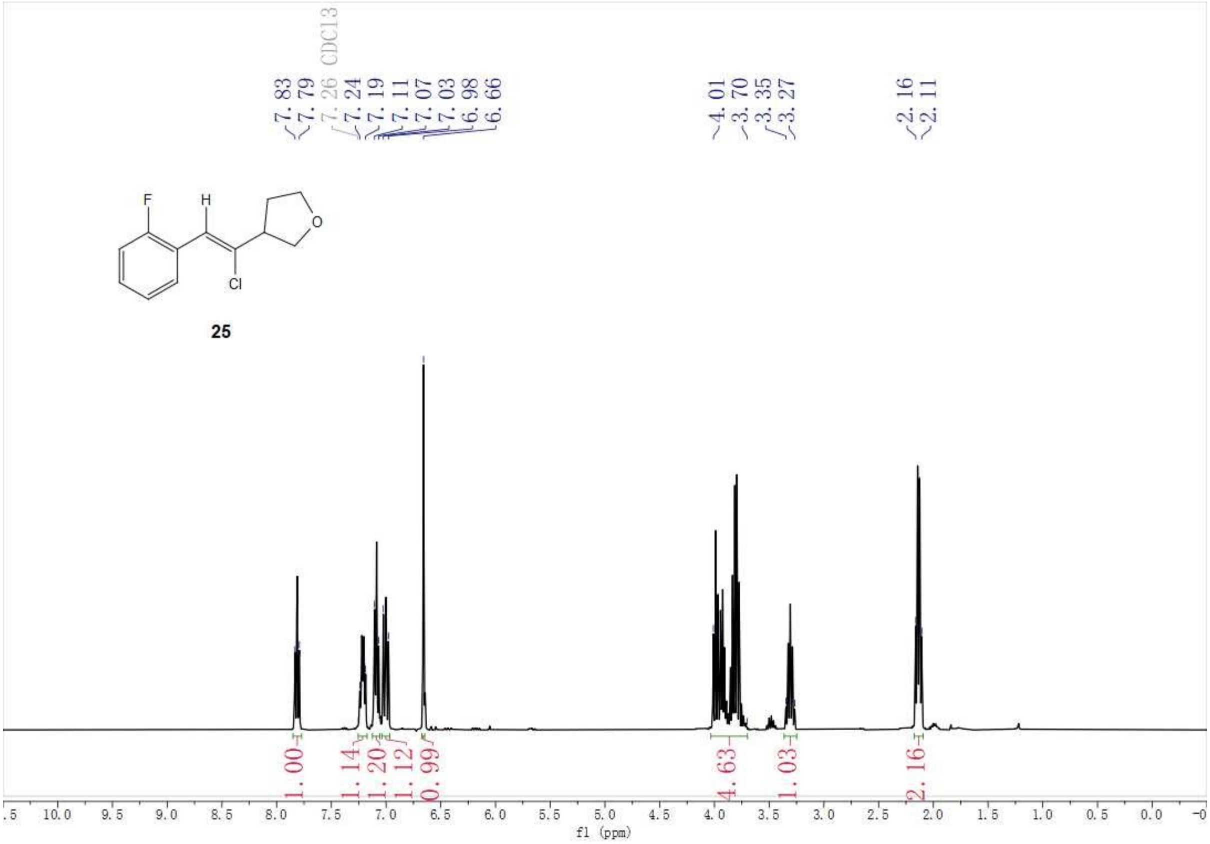

^{1}H-NMR (400 MHz, $CDCl_3$) of **25**, *Z* isomer.

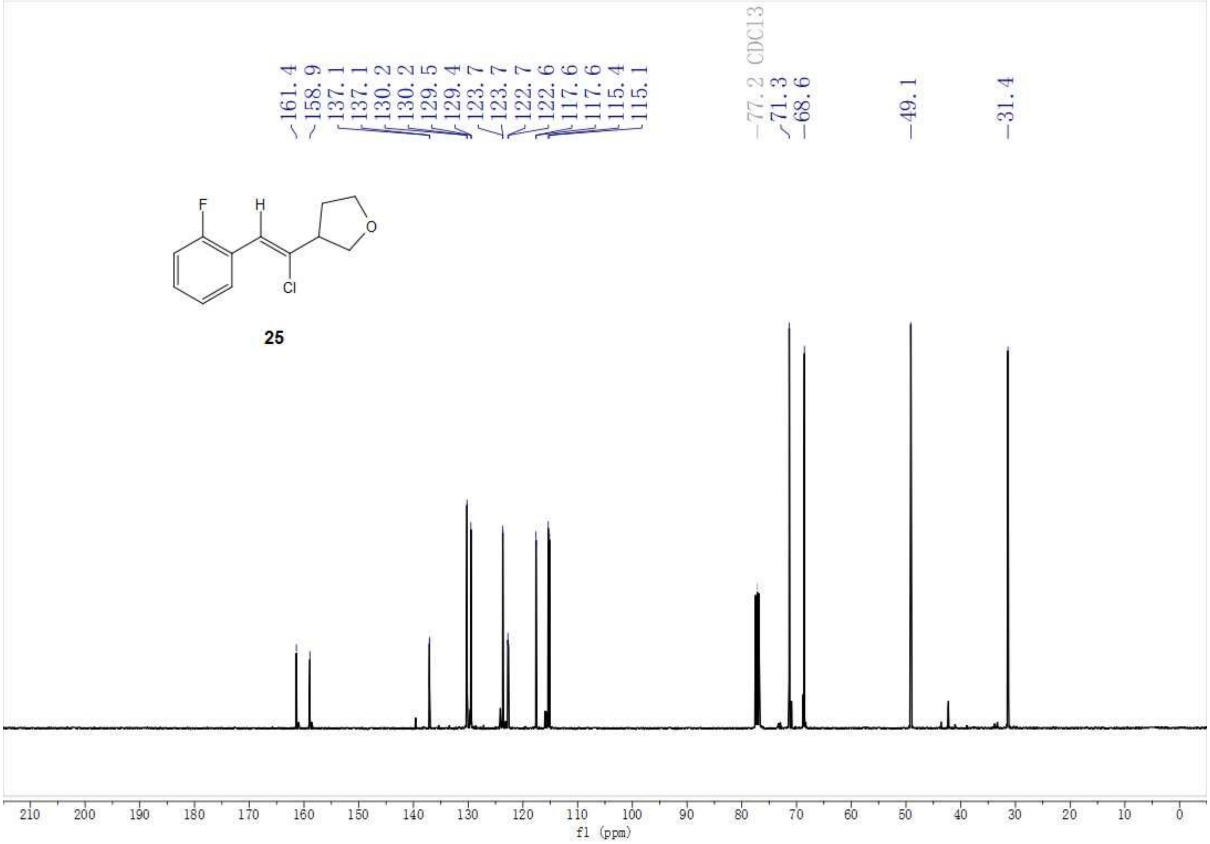

^{13}C-NMR (101 MHz, $CDCl_3$) of **25**, *Z* isomer.

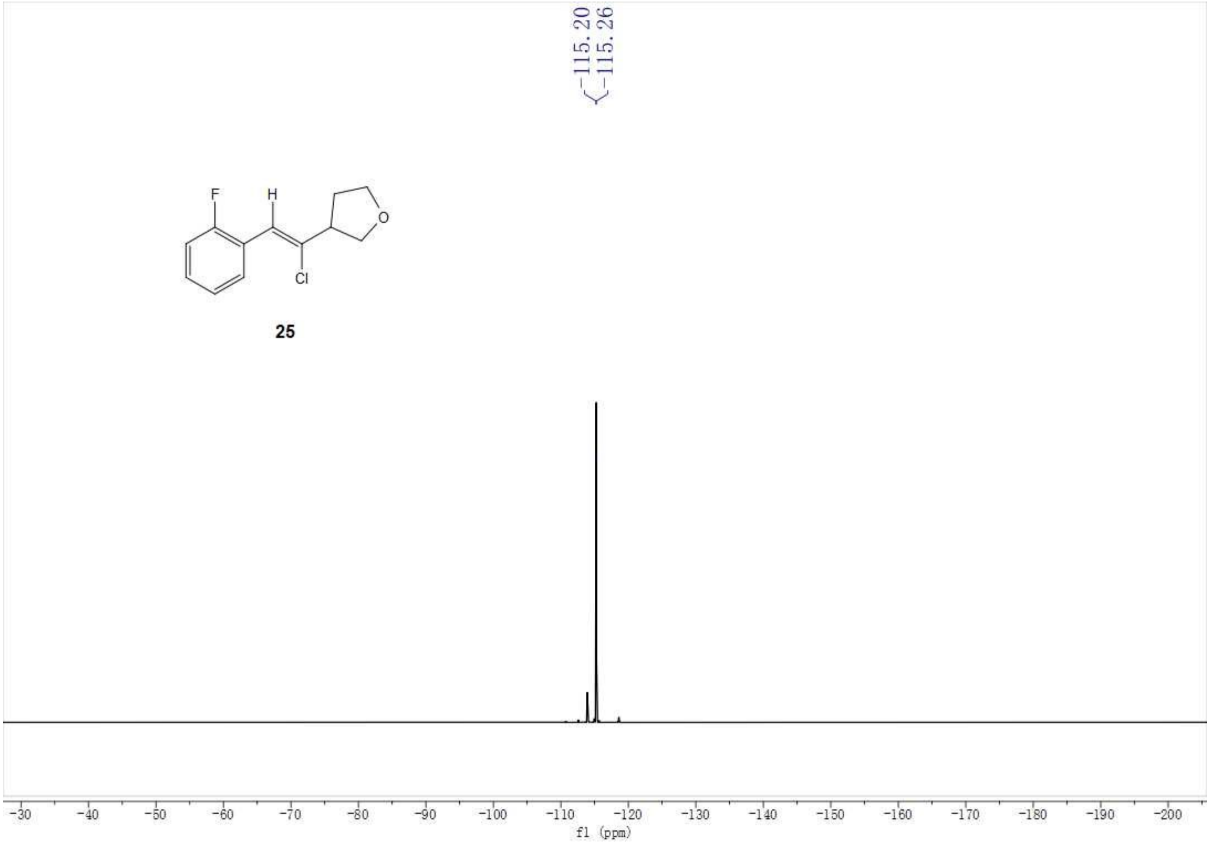

^{19}F-NMR (376 MHz, $CDCl_3$) of **25**, *Z* isomer.

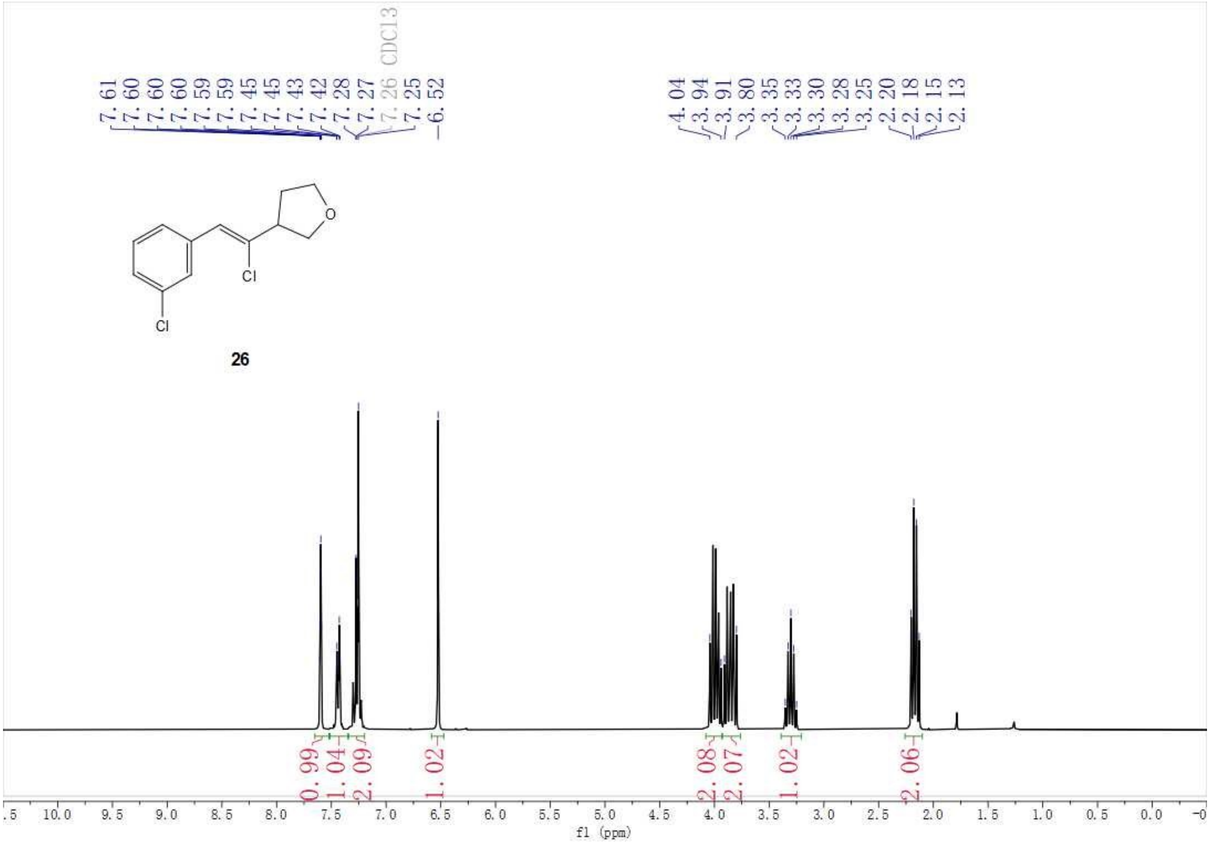

^{1}H-NMR (300 MHz, $CDCl_3$) of **26**, *Z* isomer.

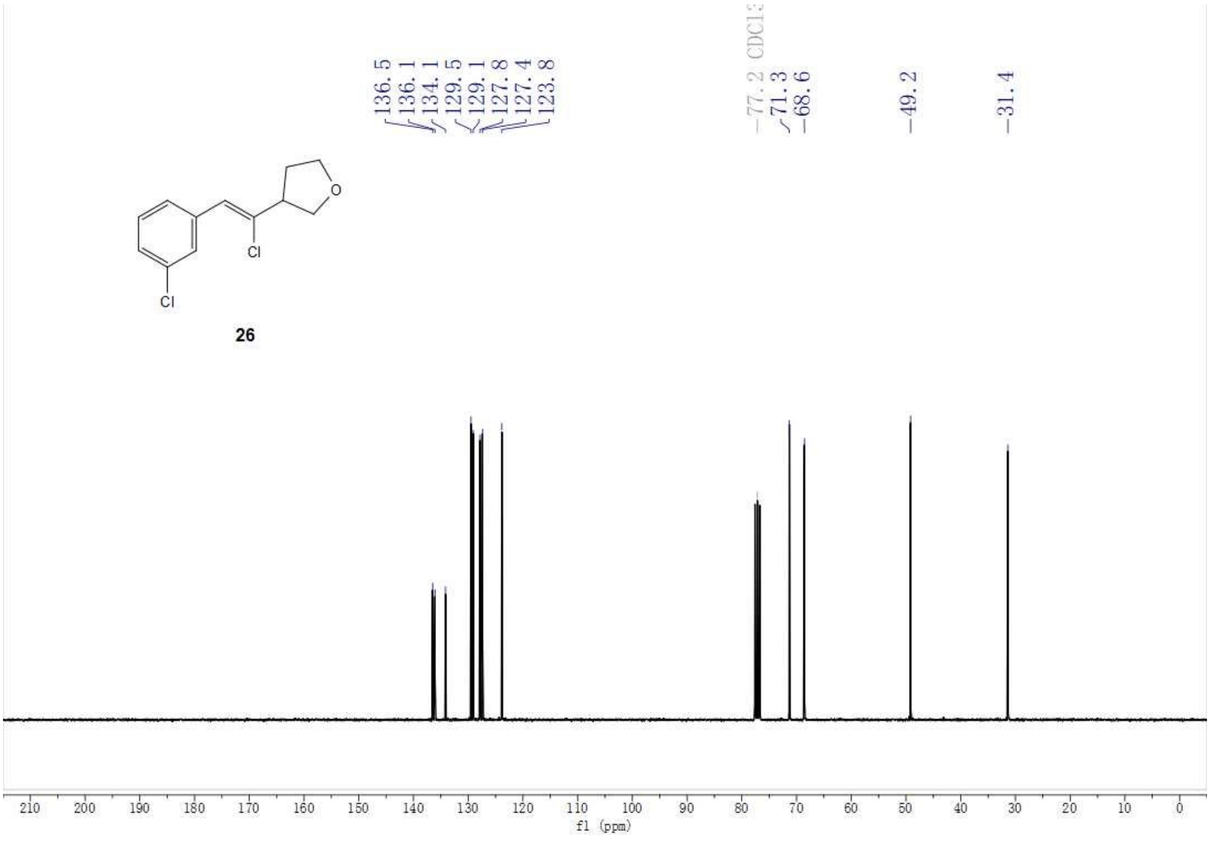

^{13}C-NMR (75 MHz, $CDCl_3$) of **26**, *Z* isomer.

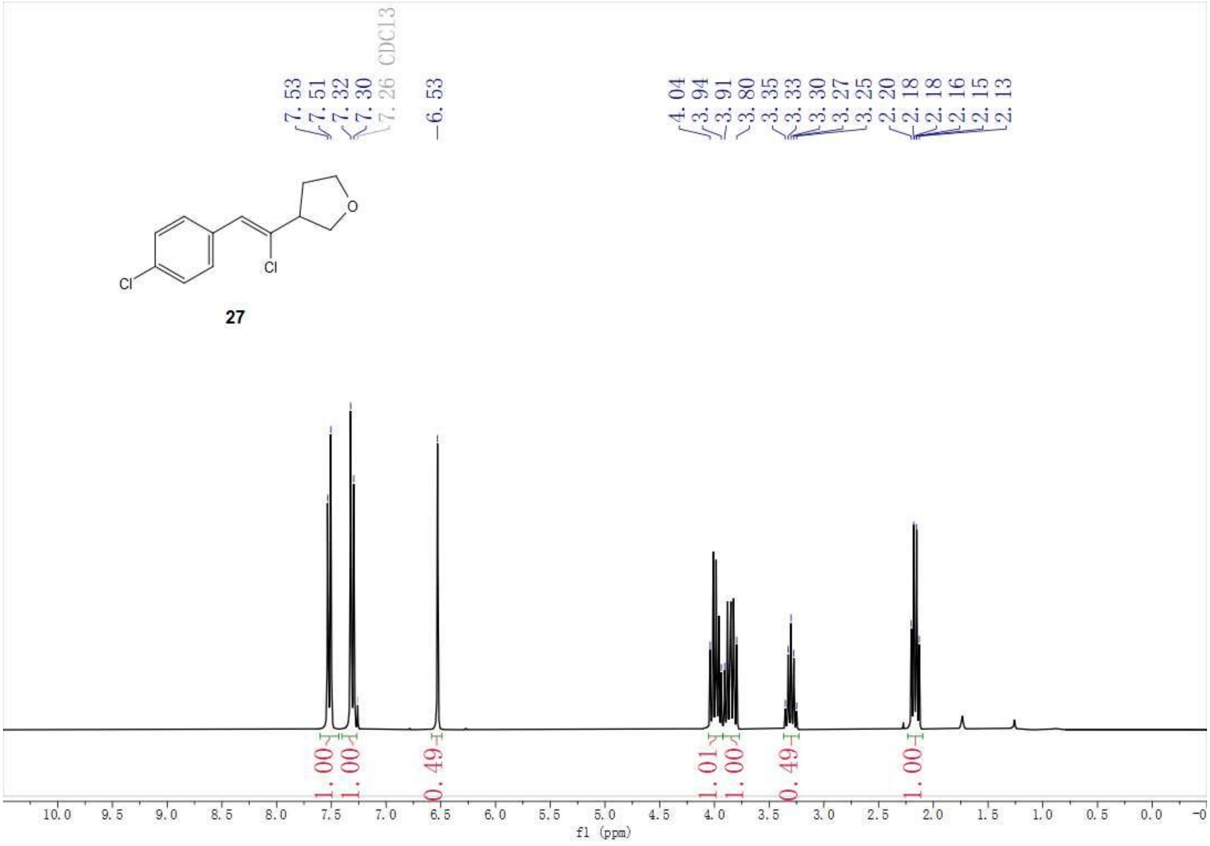

^{1}H-NMR (300 MHz, $CDCl_3$) of **27**, *Z* isomer.

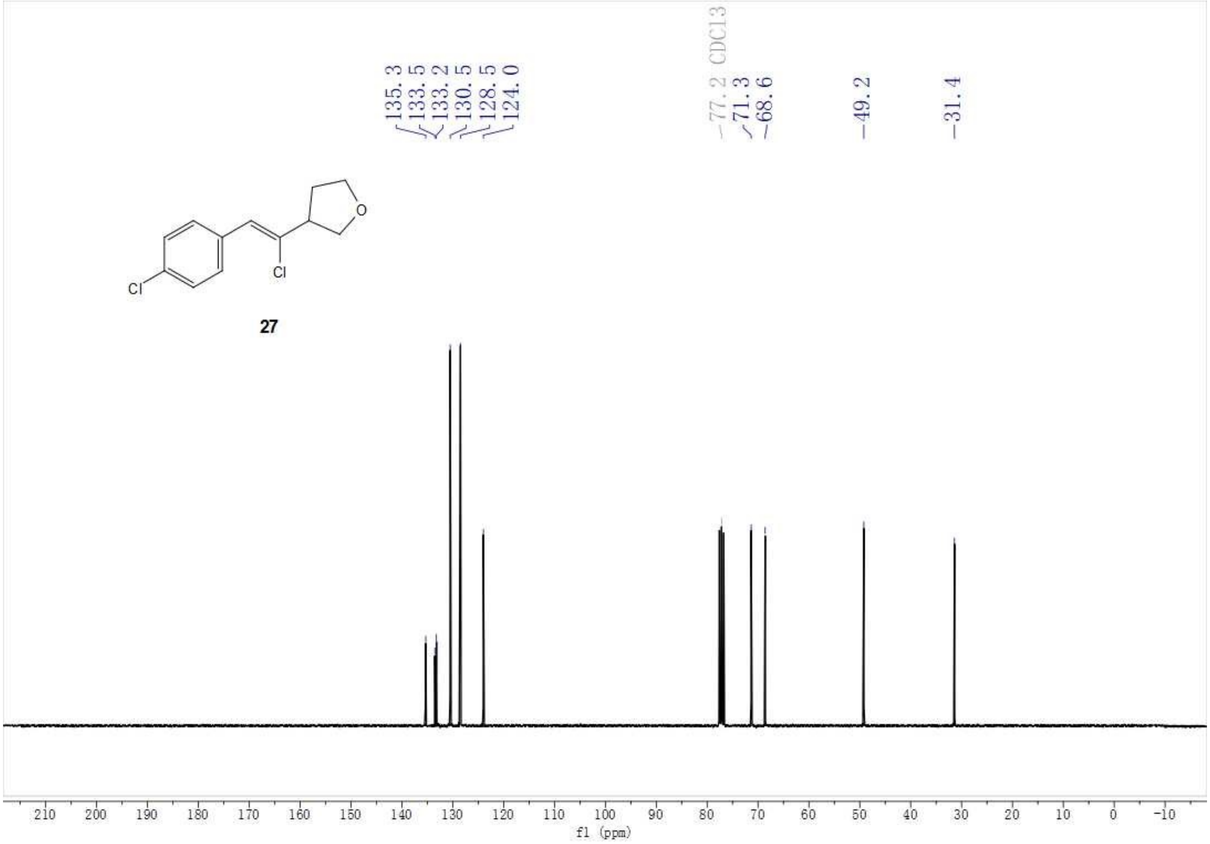

^{13}C-NMR (75 MHz, $CDCl_3$) of **27**, *Z* isomer.

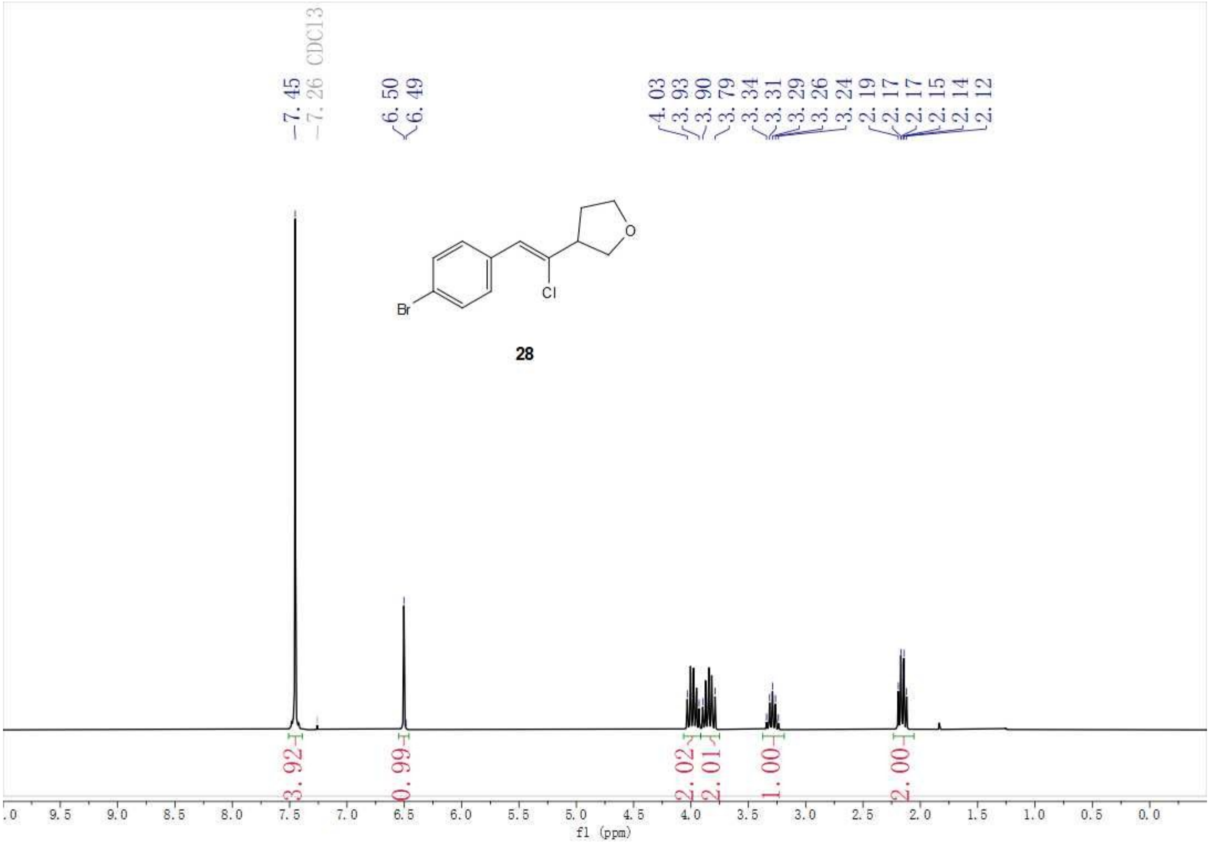

^{1}H-NMR (300 MHz, $CDCl_3$) of **28**, *Z* isomer.

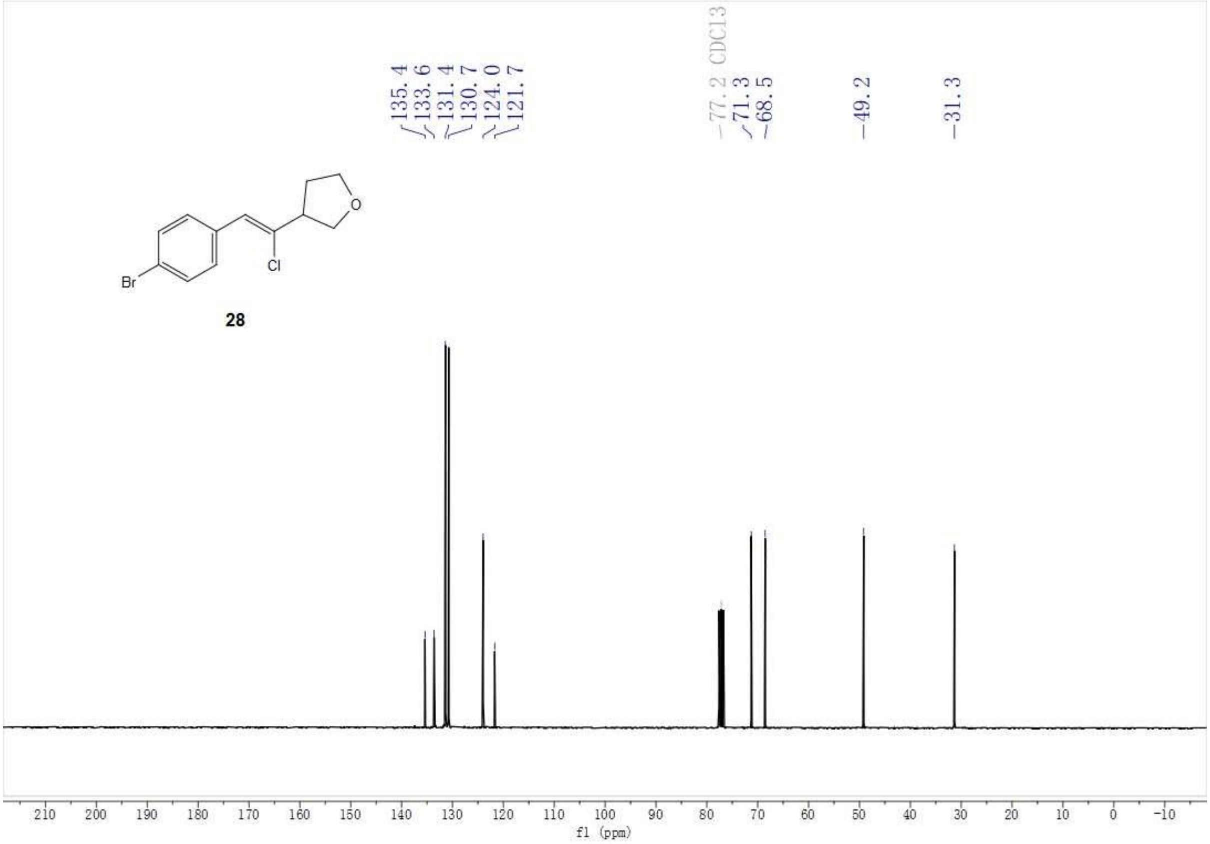

^{13}C-NMR (75 MHz, $CDCl_3$) of **28**, *Z* isomer.

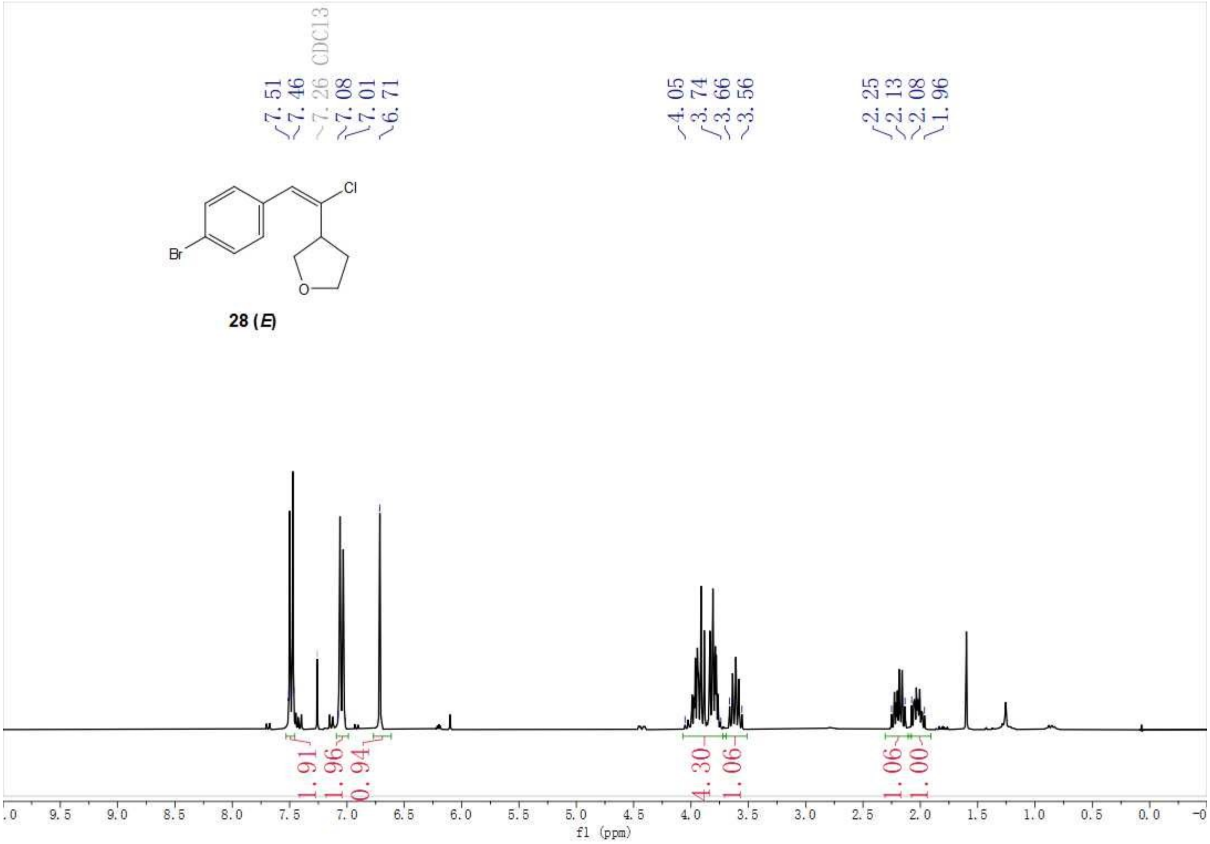

^{1}H-NMR (300 MHz, $CDCl_3$) of **28**, *E* isomer.

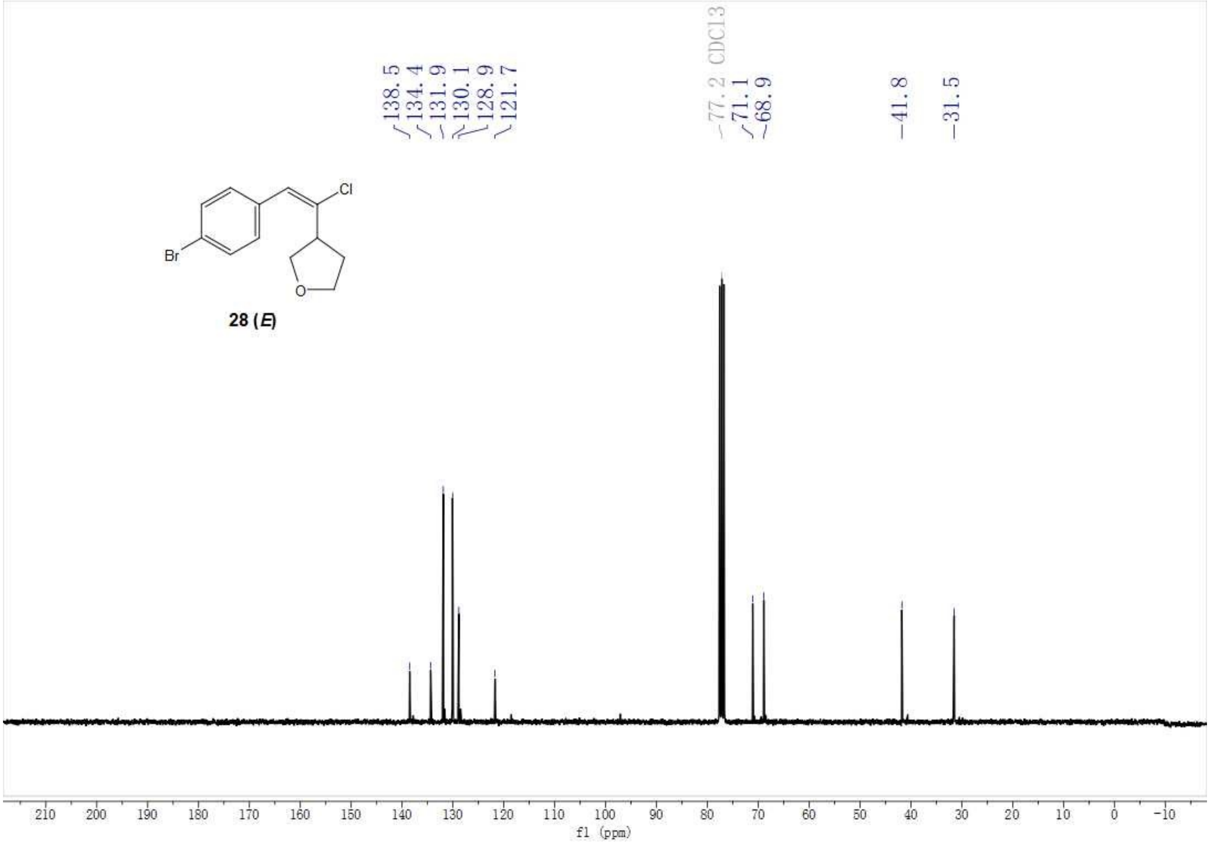

^{13}C-NMR (75 MHz, $CDCl_3$) of **28**, *E* isomer.

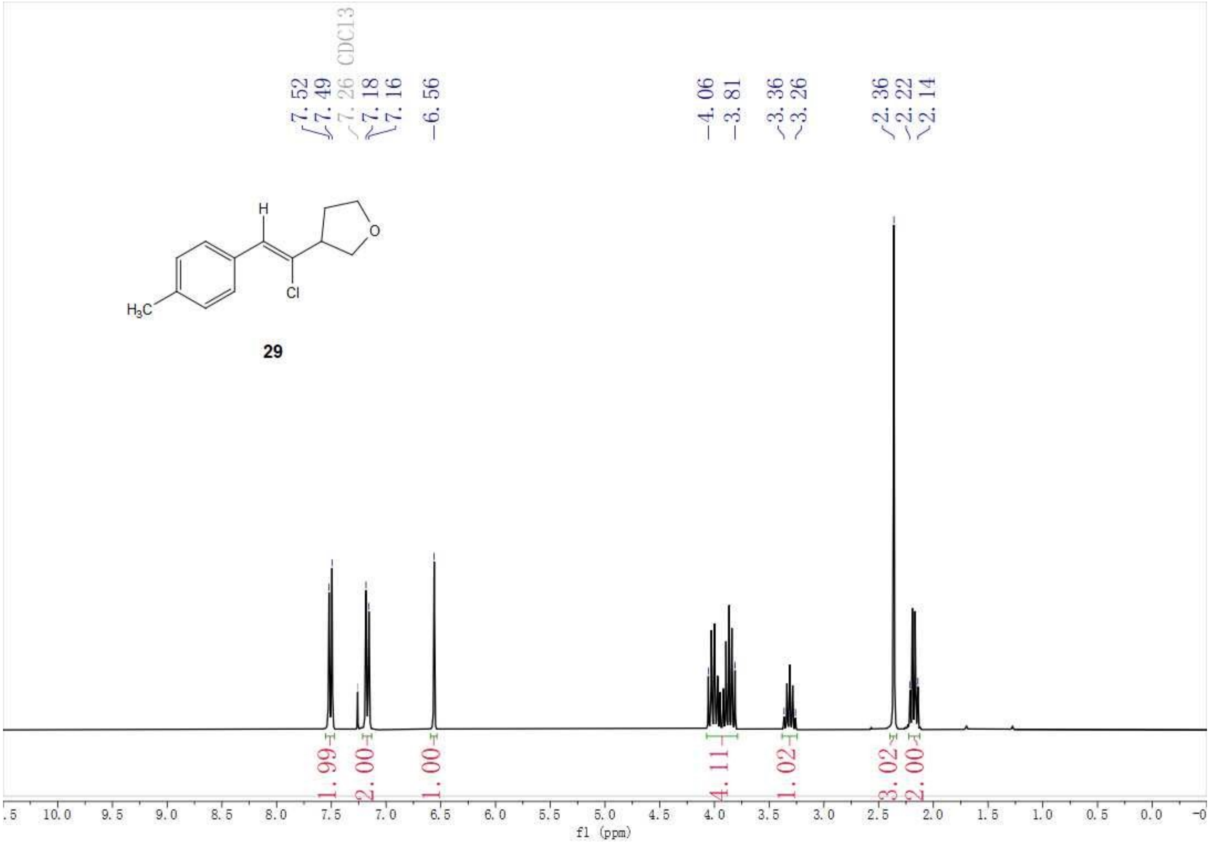

^{1}H-NMR (300 MHz, $CDCl_3$) of **29**, *Z* isomer.

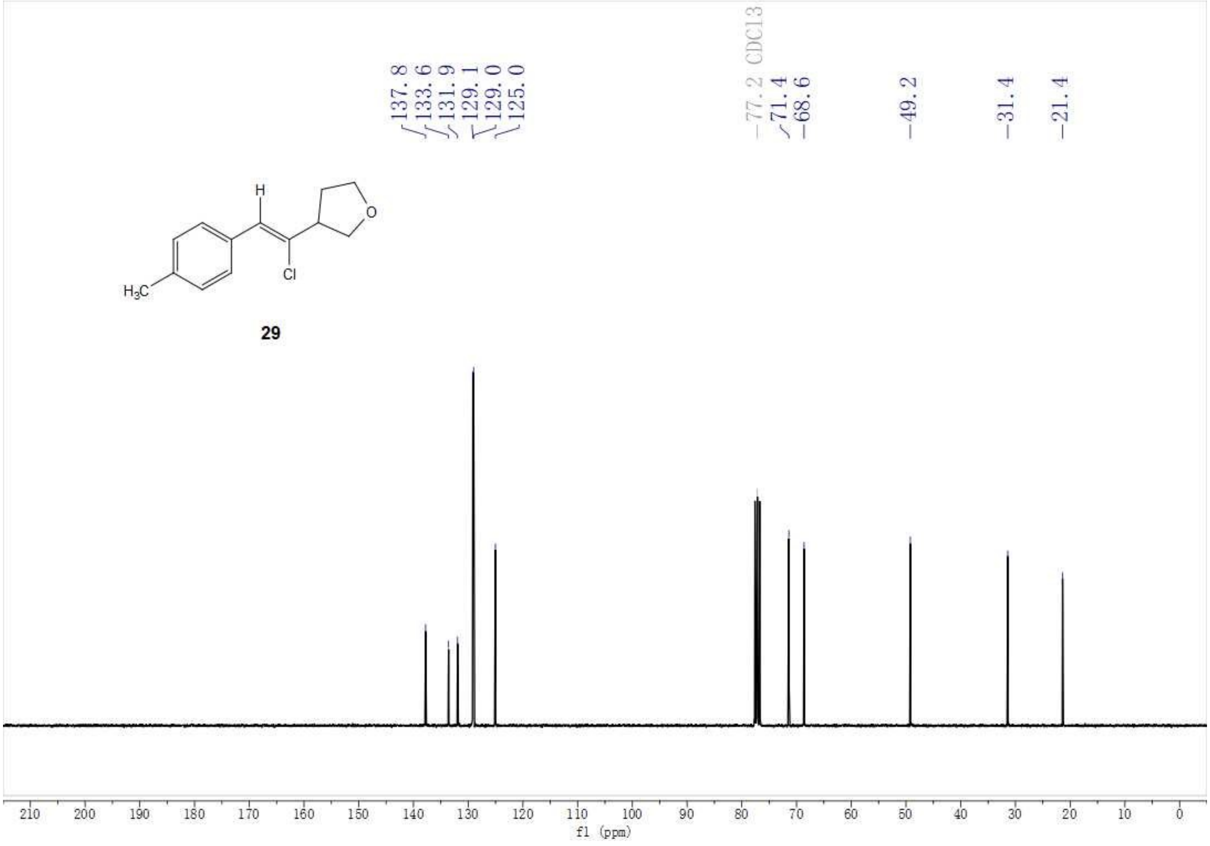

^{13}C-NMR (75 MHz, $CDCl_3$) of **29**, *Z* isomer.

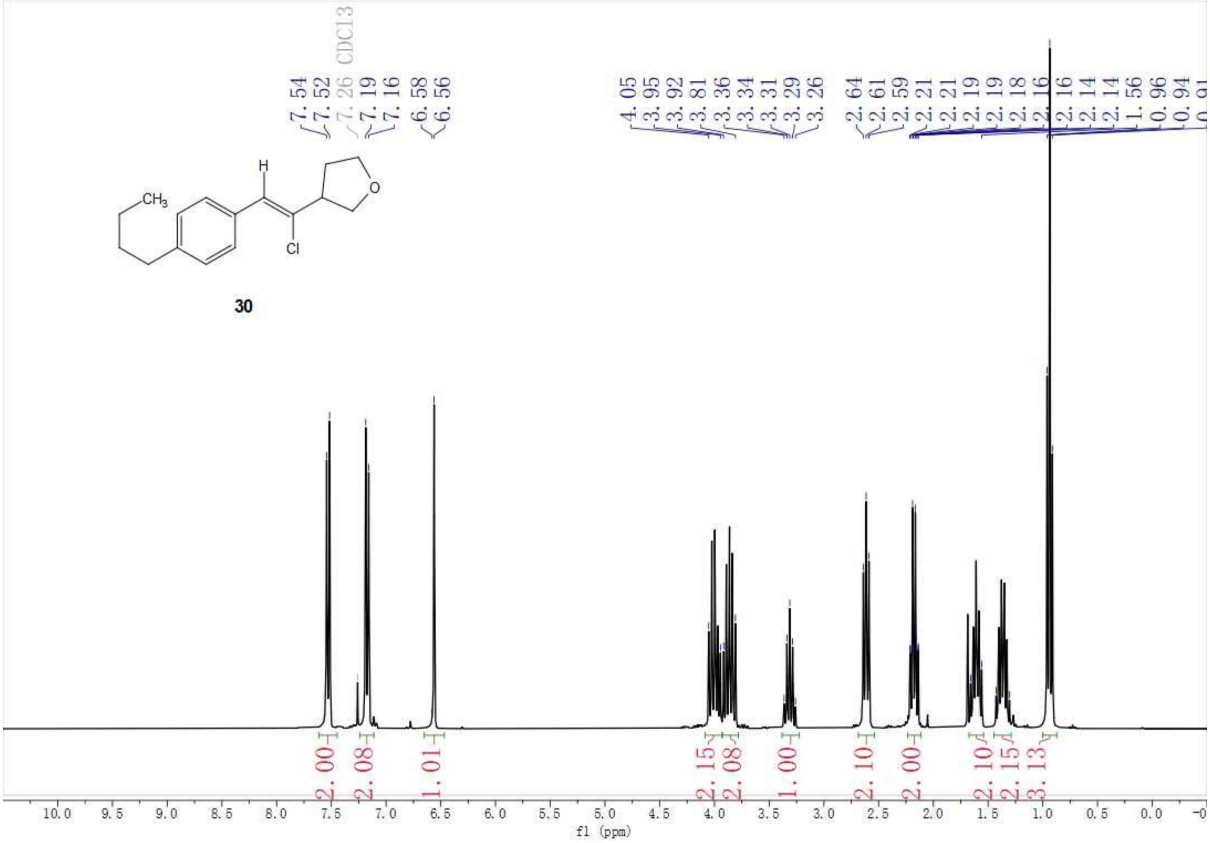

^{1}H-NMR (300 MHz, $CDCl_3$) of **30**, *Z* isomer.

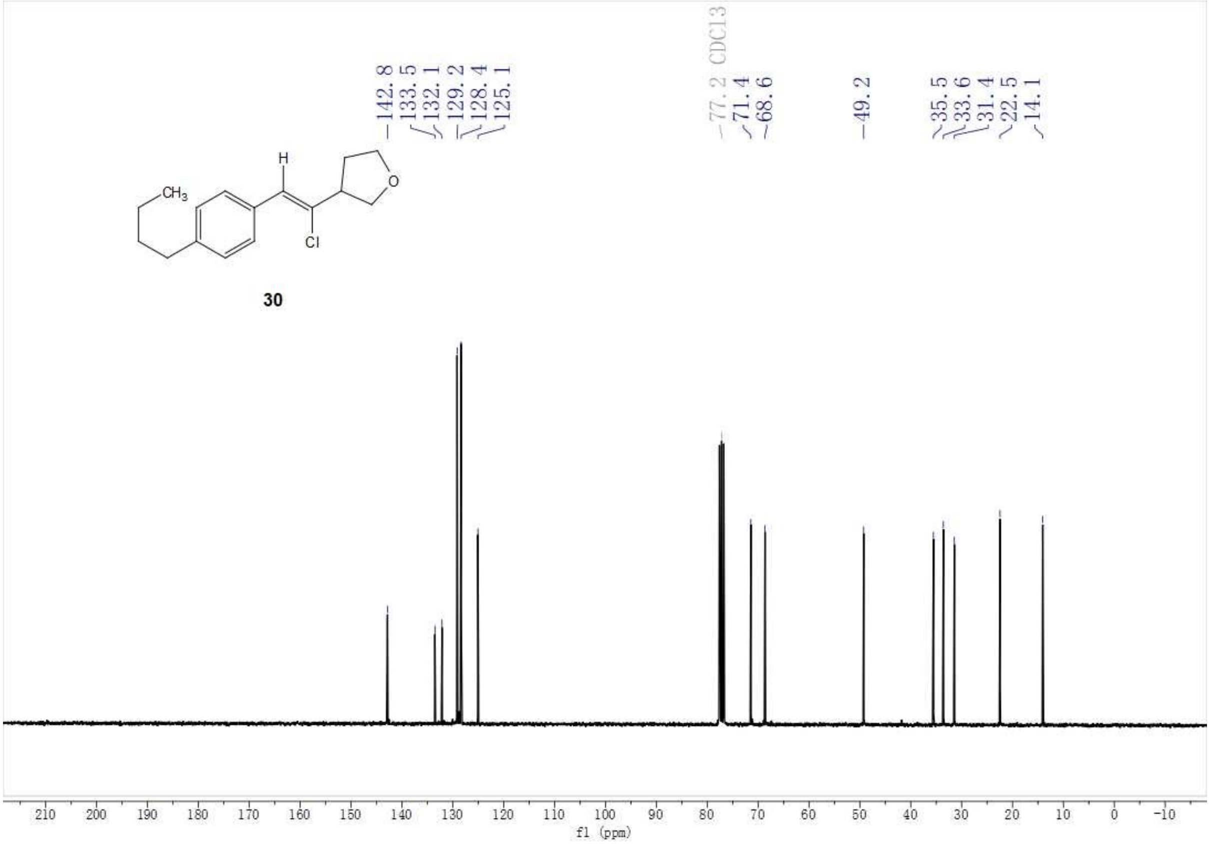

^{13}C-NMR (75 MHz, $CDCl_3$) of **30**, *Z* isomer.

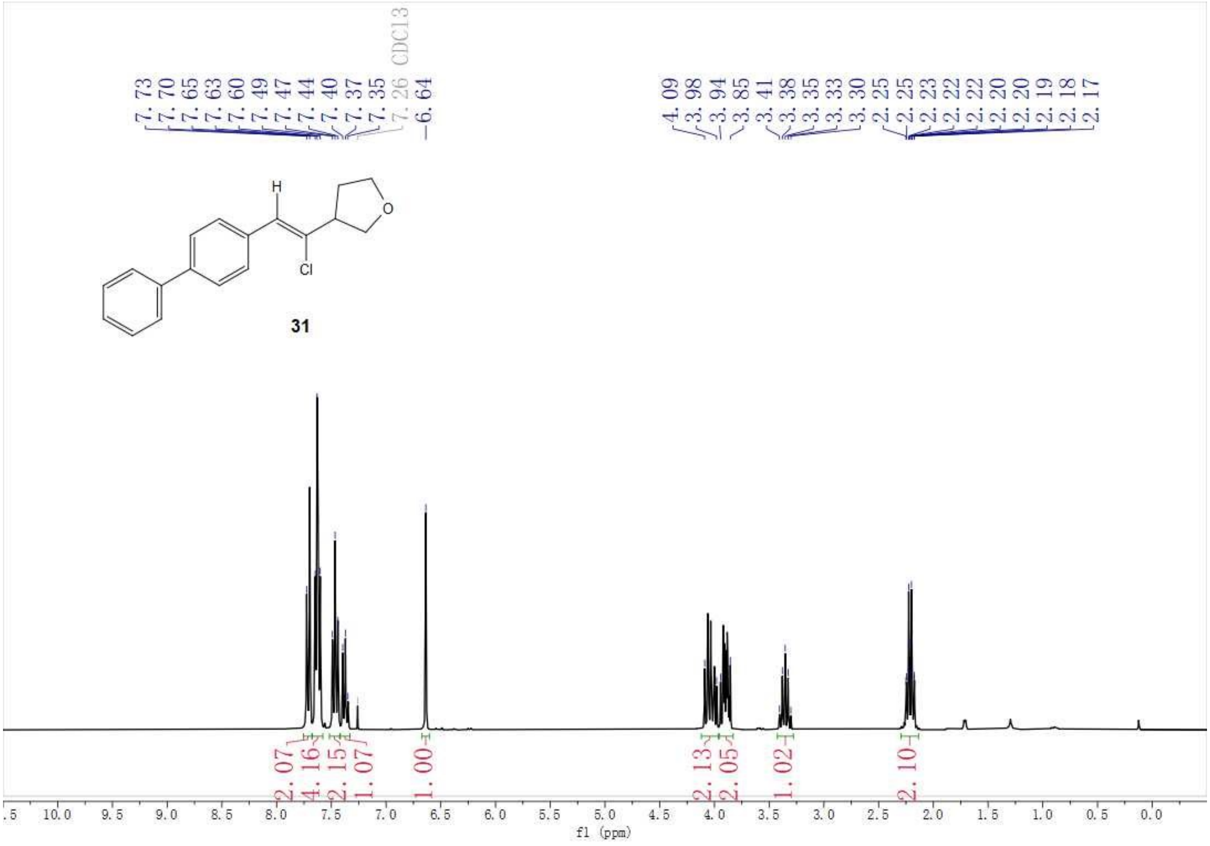

^{1}H-NMR (300 MHz, $CDCl_3$) of **31**, *Z* isomer.

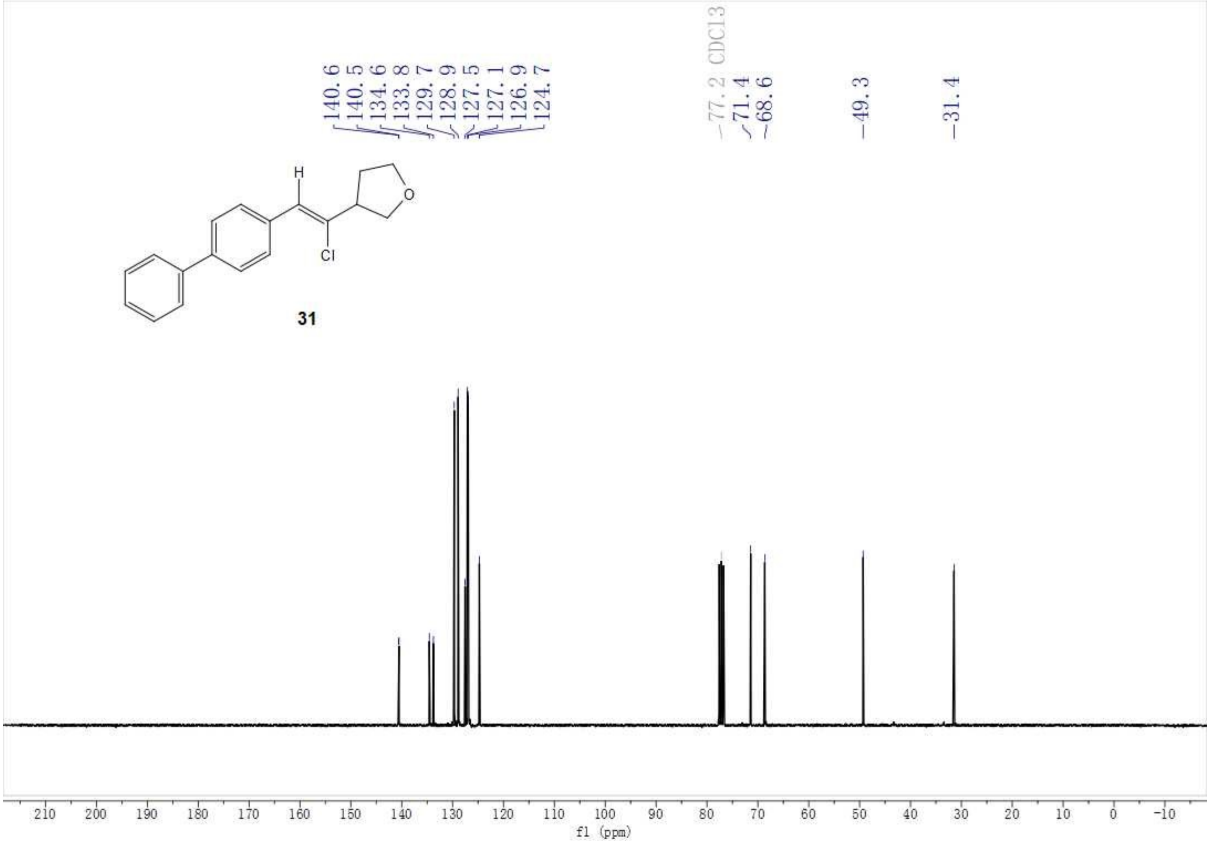

^{13}C-NMR (75 MHz, $CDCl_3$) of **31**, *Z* isomer.

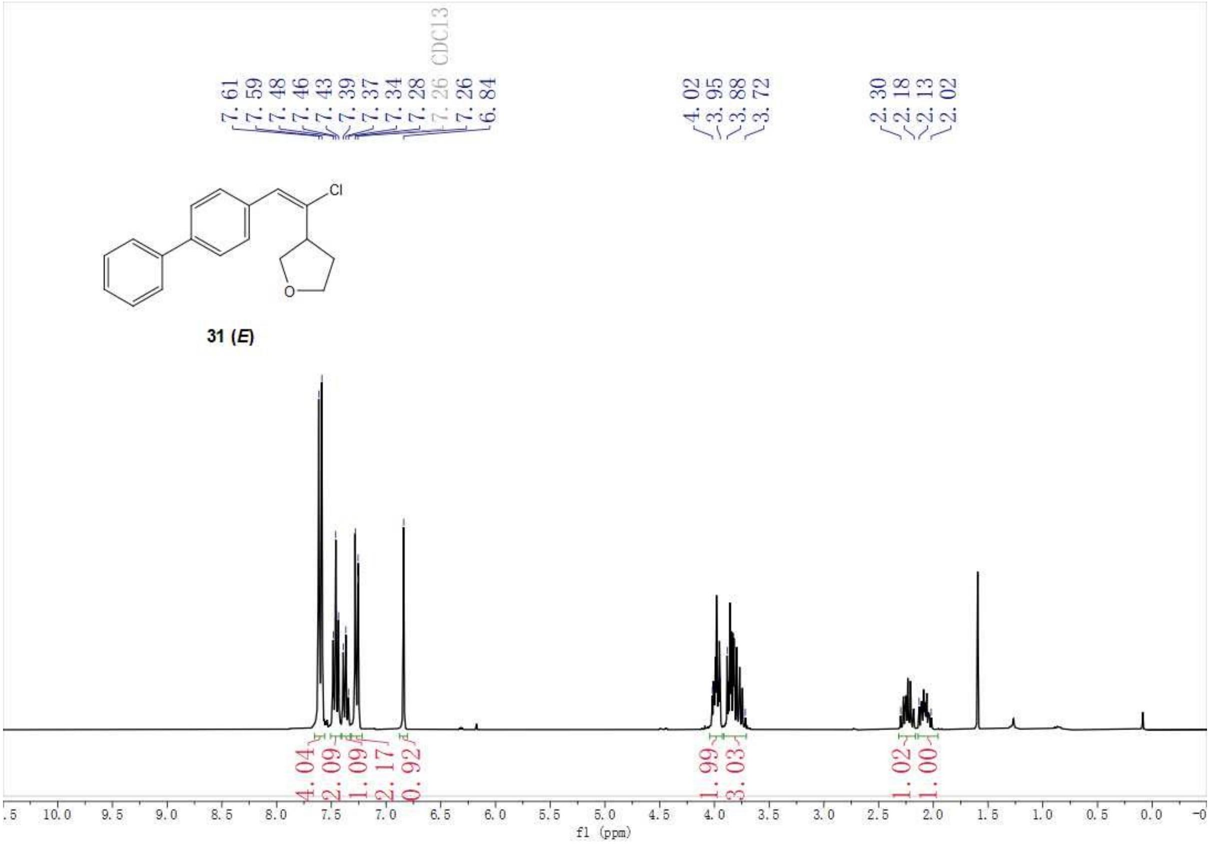

^{1}H-NMR (300 MHz, $CDCl_3$) of **31**, *E* isomer.

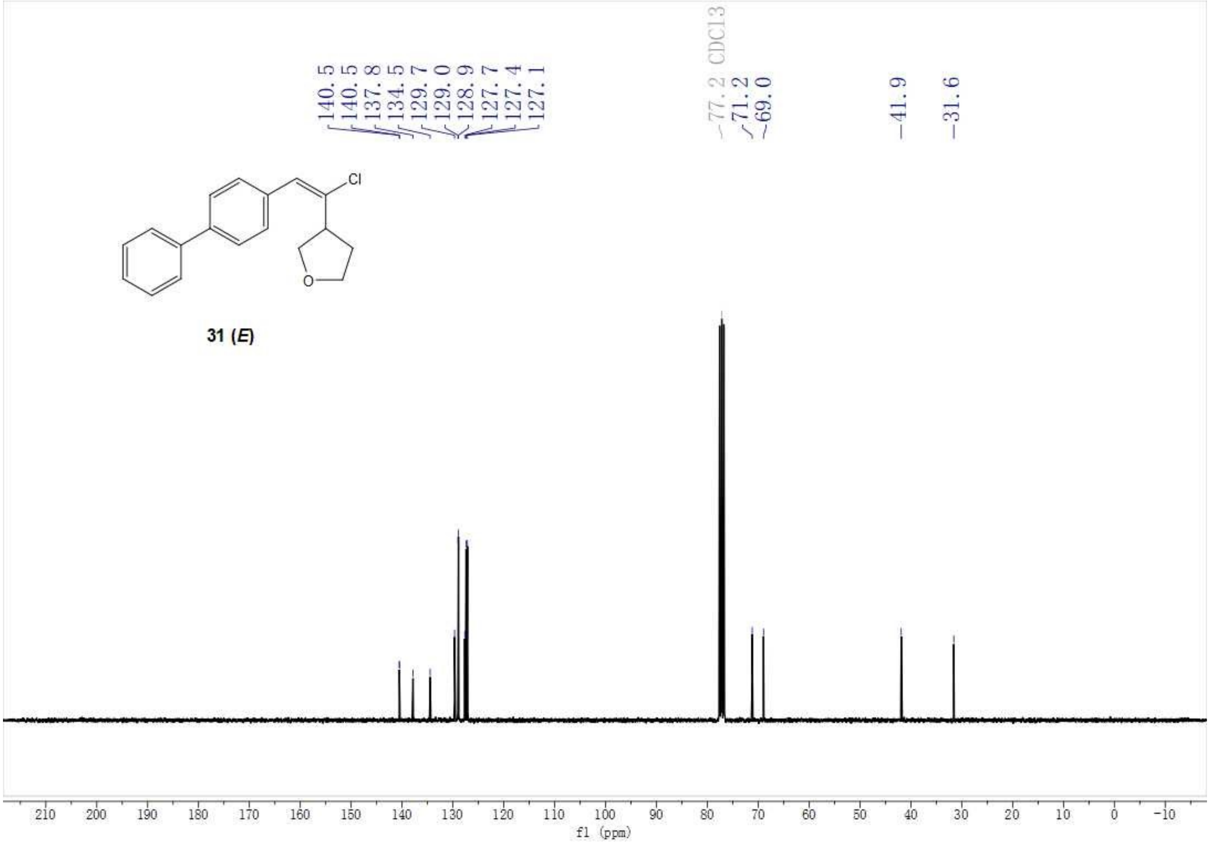

^{13}C-NMR (75 MHz, $CDCl_3$) of **31**, *E* isomer.

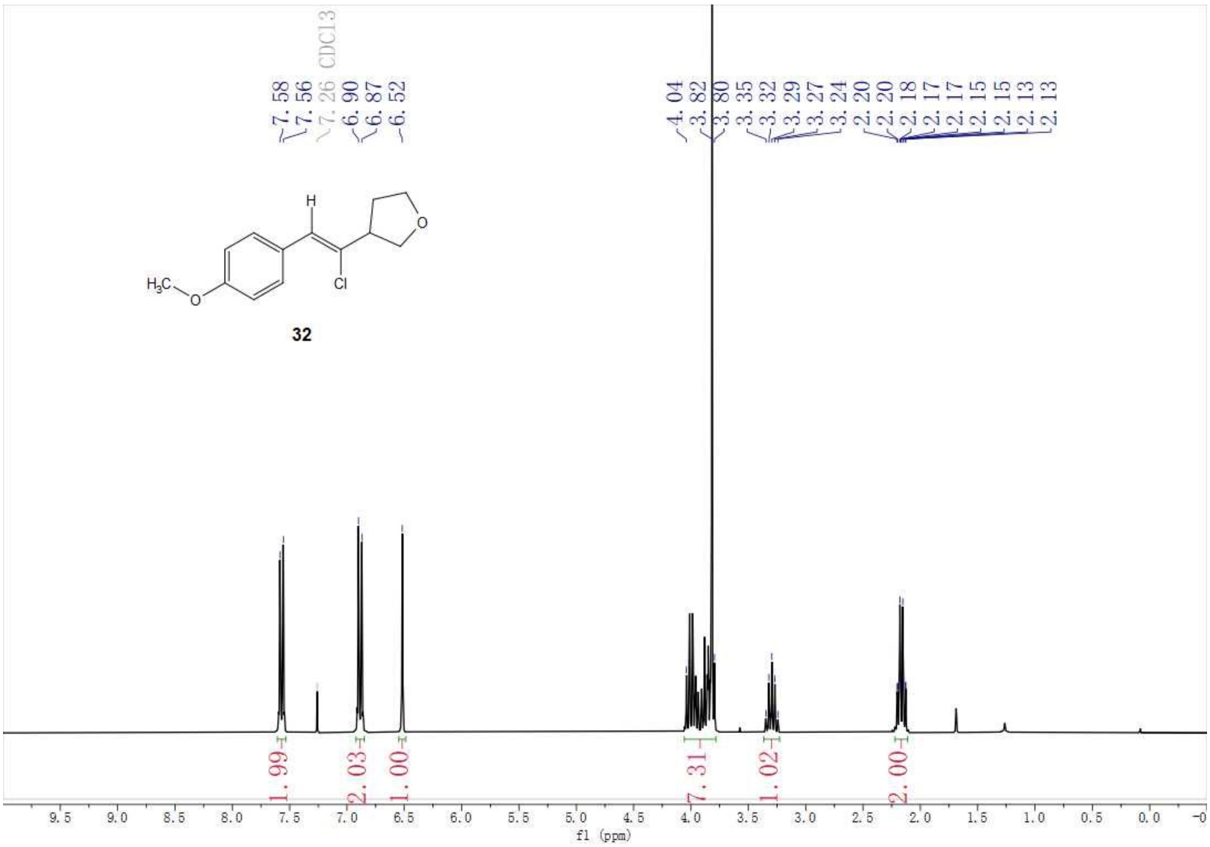

^{1}H-NMR (300 MHz, $CDCl_3$) of **32**, *Z* isomer.

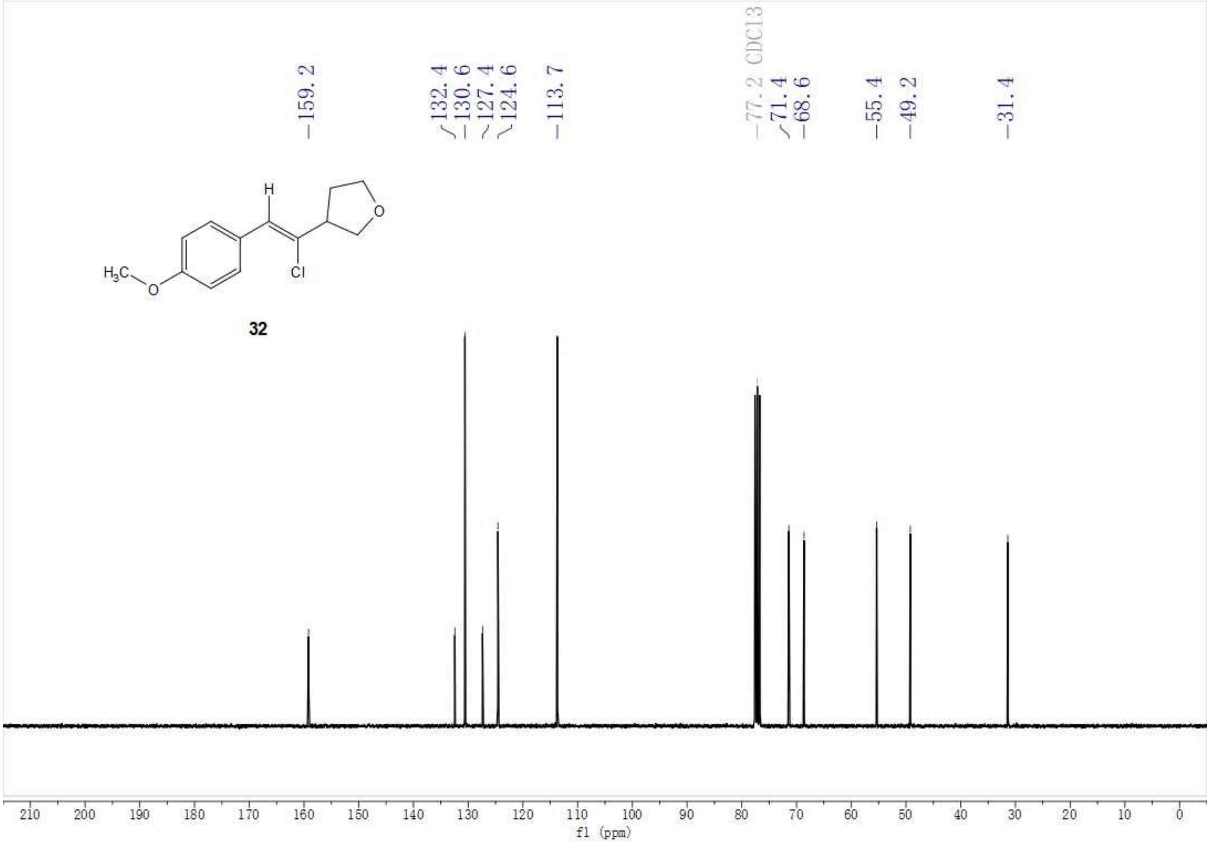

^{13}C-NMR (75 MHz, $CDCl_3$) of **32**, *Z* isomer.

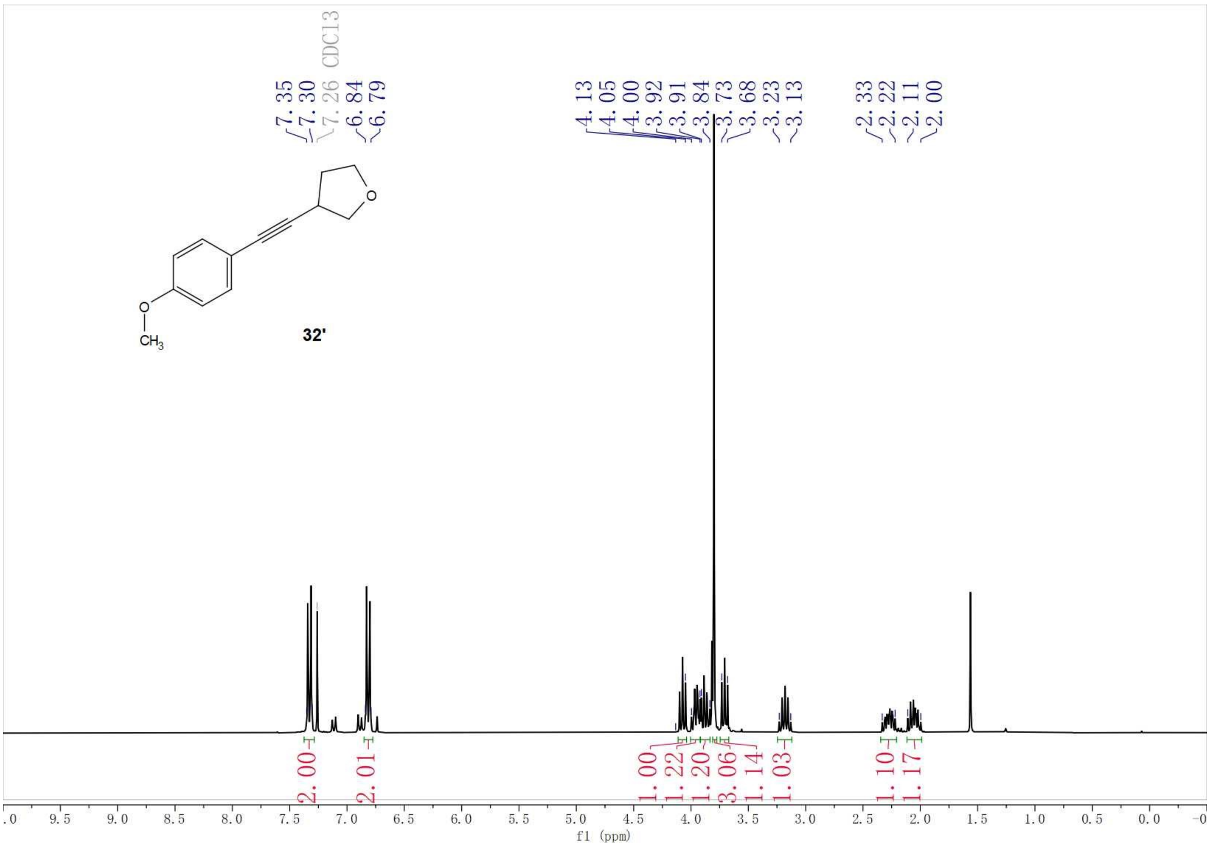

^{1}H-NMR (300 MHz, $CDCl_3$) of **32′**

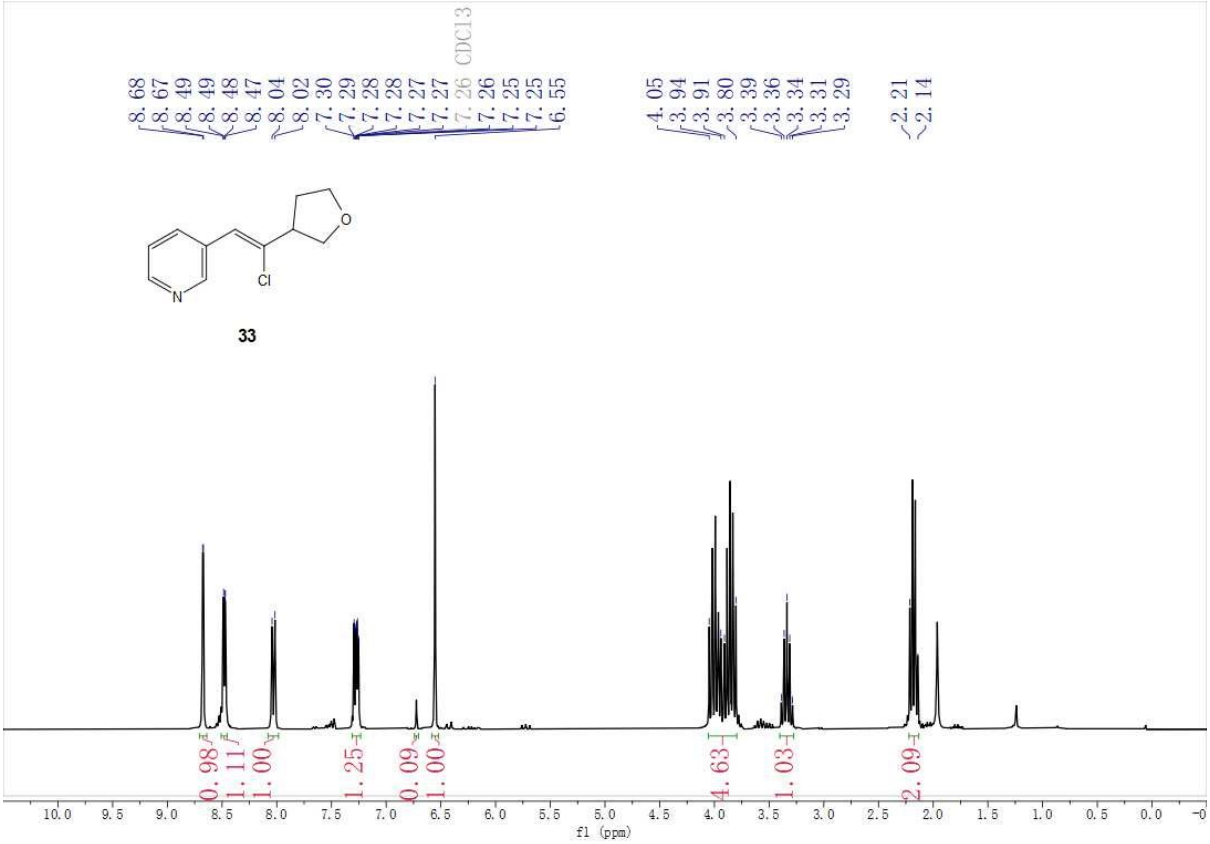

^{1}H-NMR (300 MHz, $CDCl_3$) of **33**, *Z* isomer.

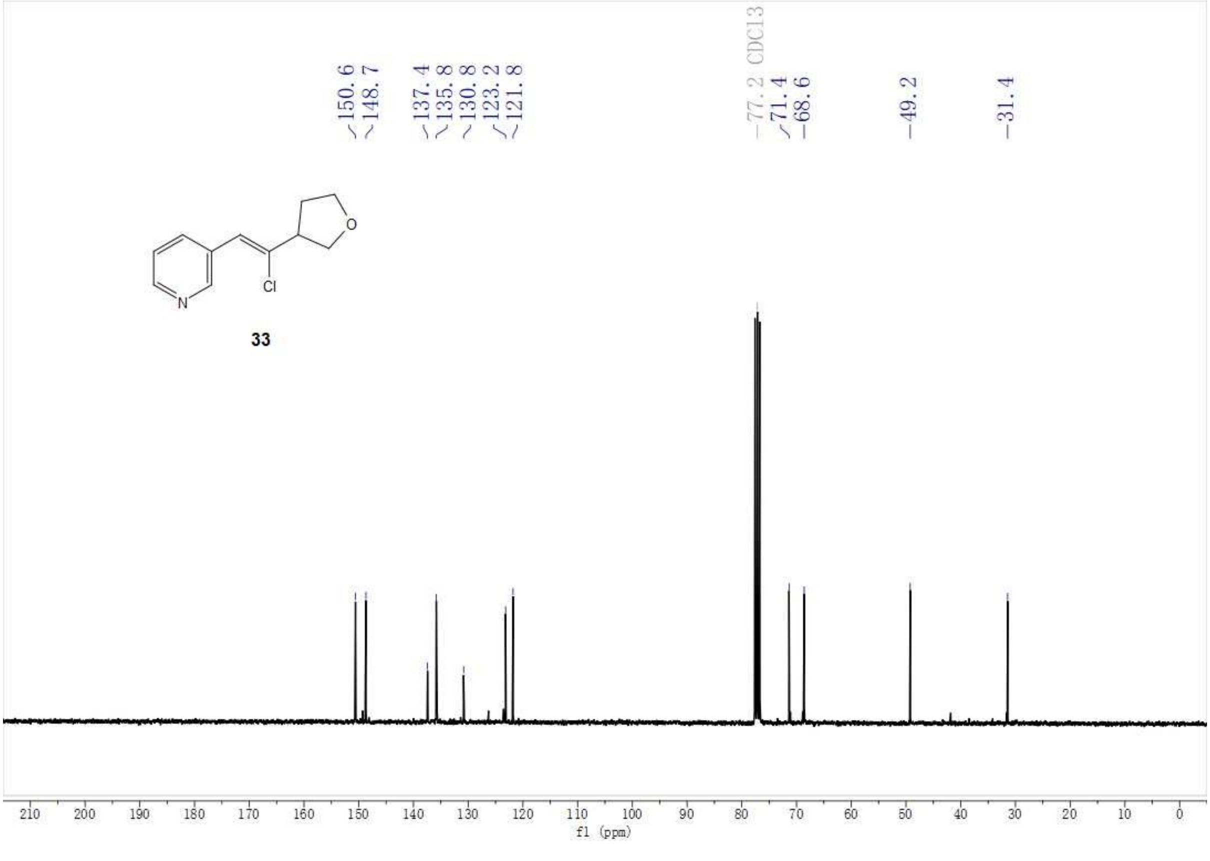

^{13}C-NMR (75 MHz, $CDCl_3$) of **33**, *Z* isomer.

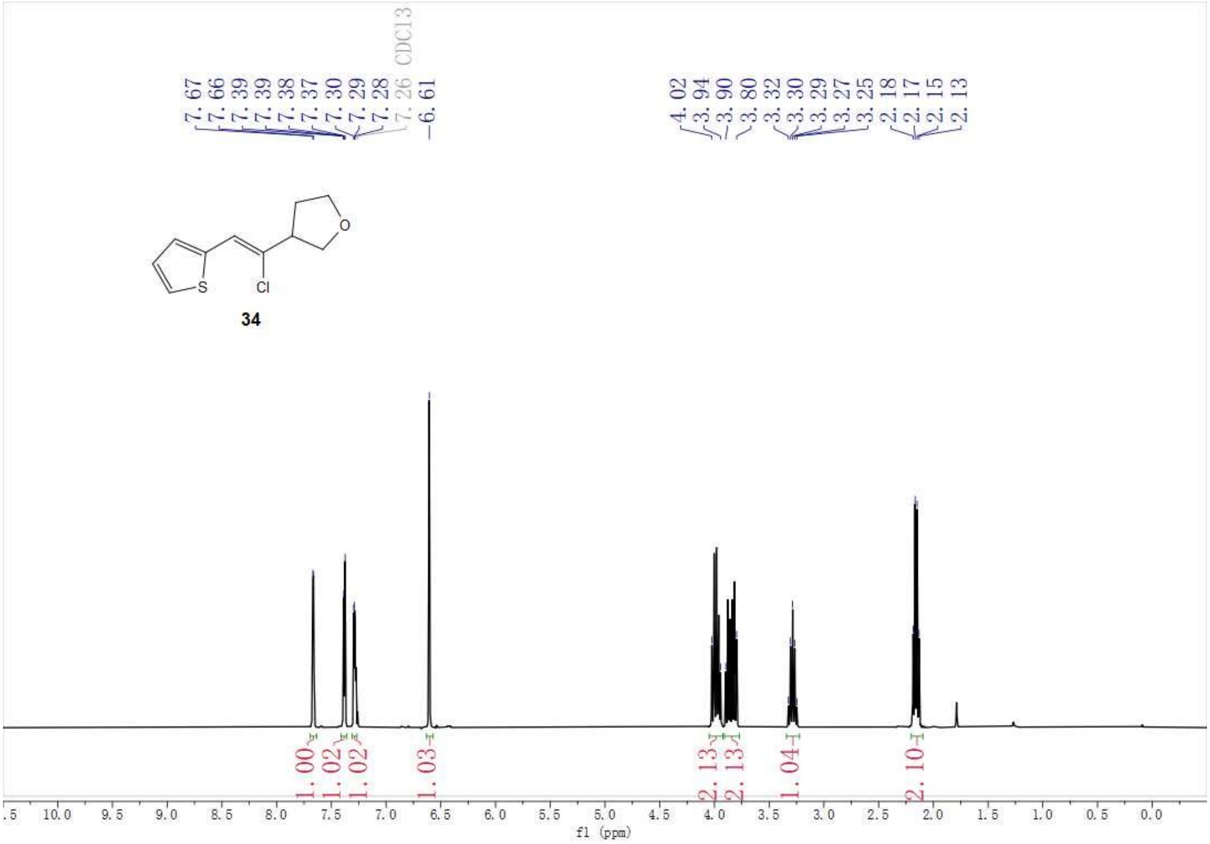

^{1}H-NMR (400 MHz, $CDCl_3$) of **34**, *Z* isomer.

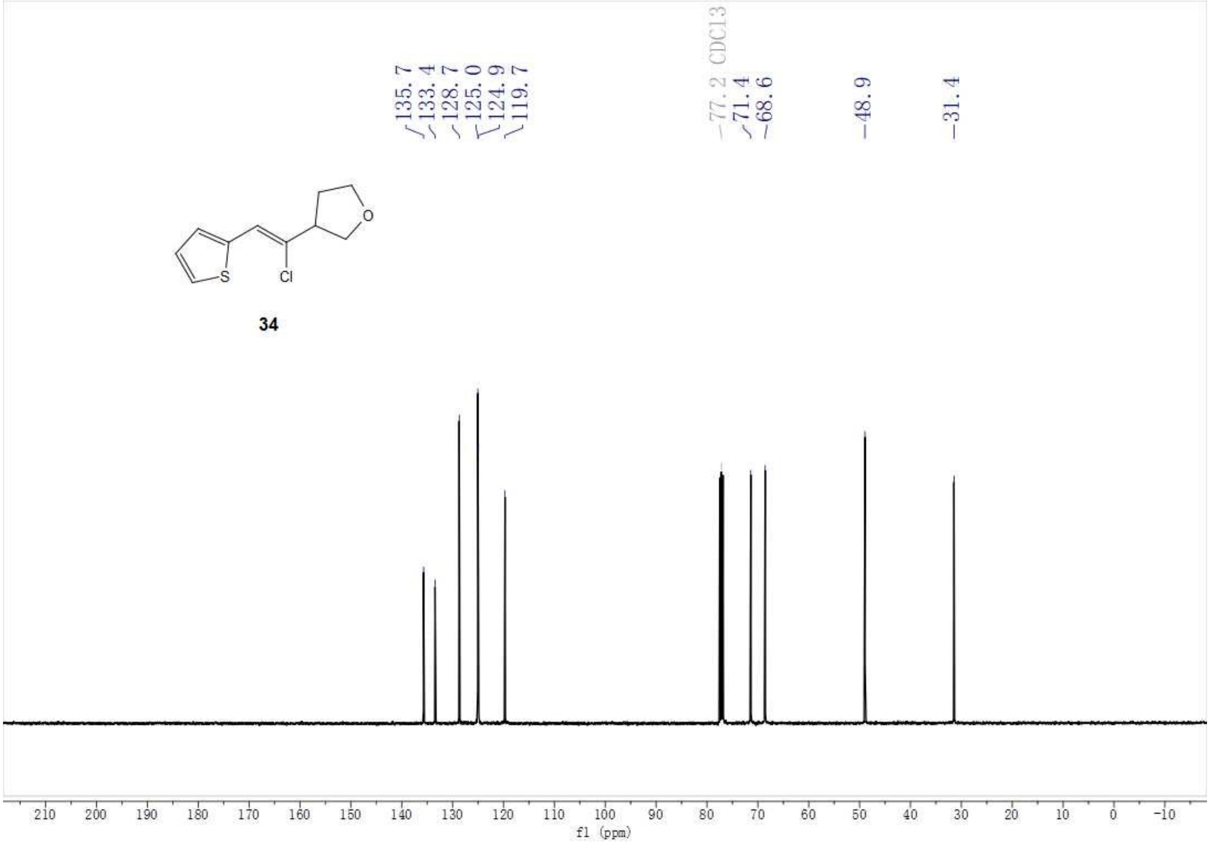

^{13}C-NMR (101 MHz, $CDCl_3$) of **34**, *Z* isomer.

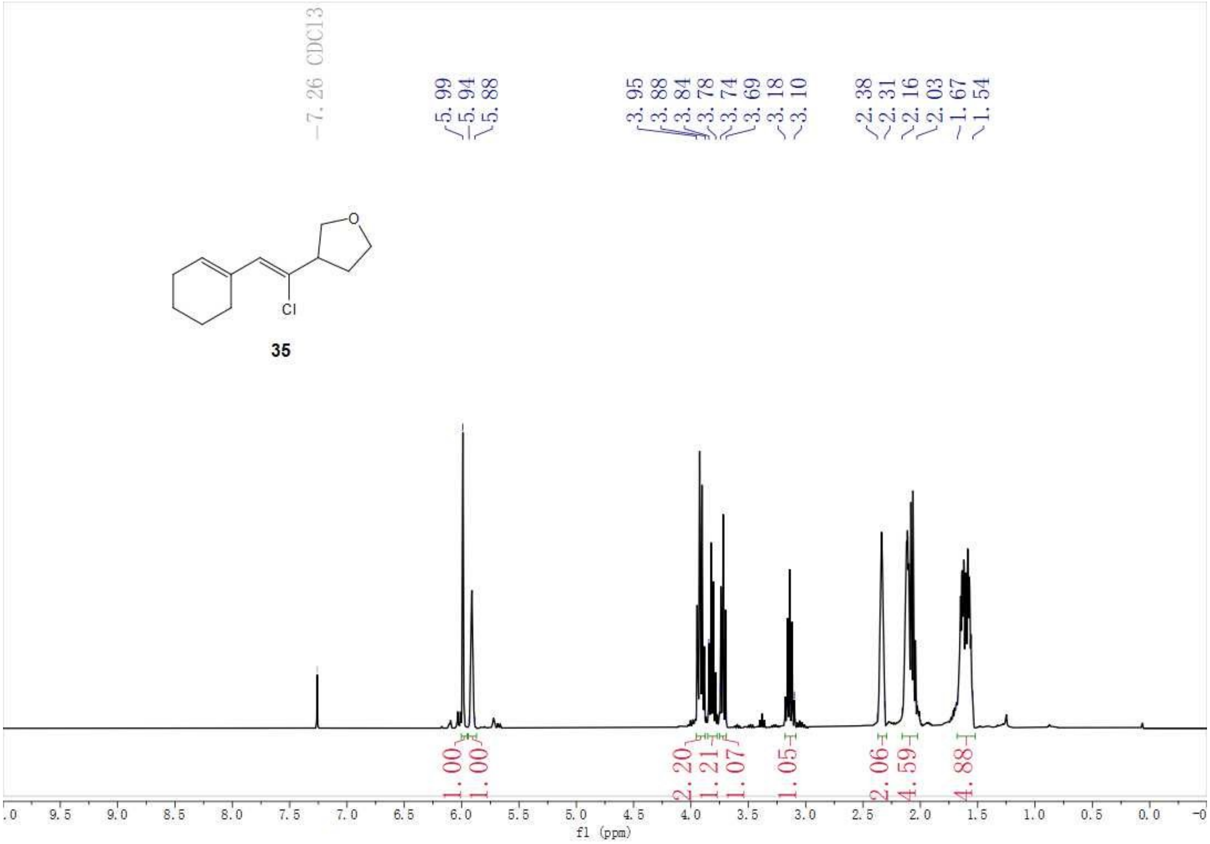

^{1}H-NMR (400 MHz, $CDCl_3$) of **35**, *Z* isomer.

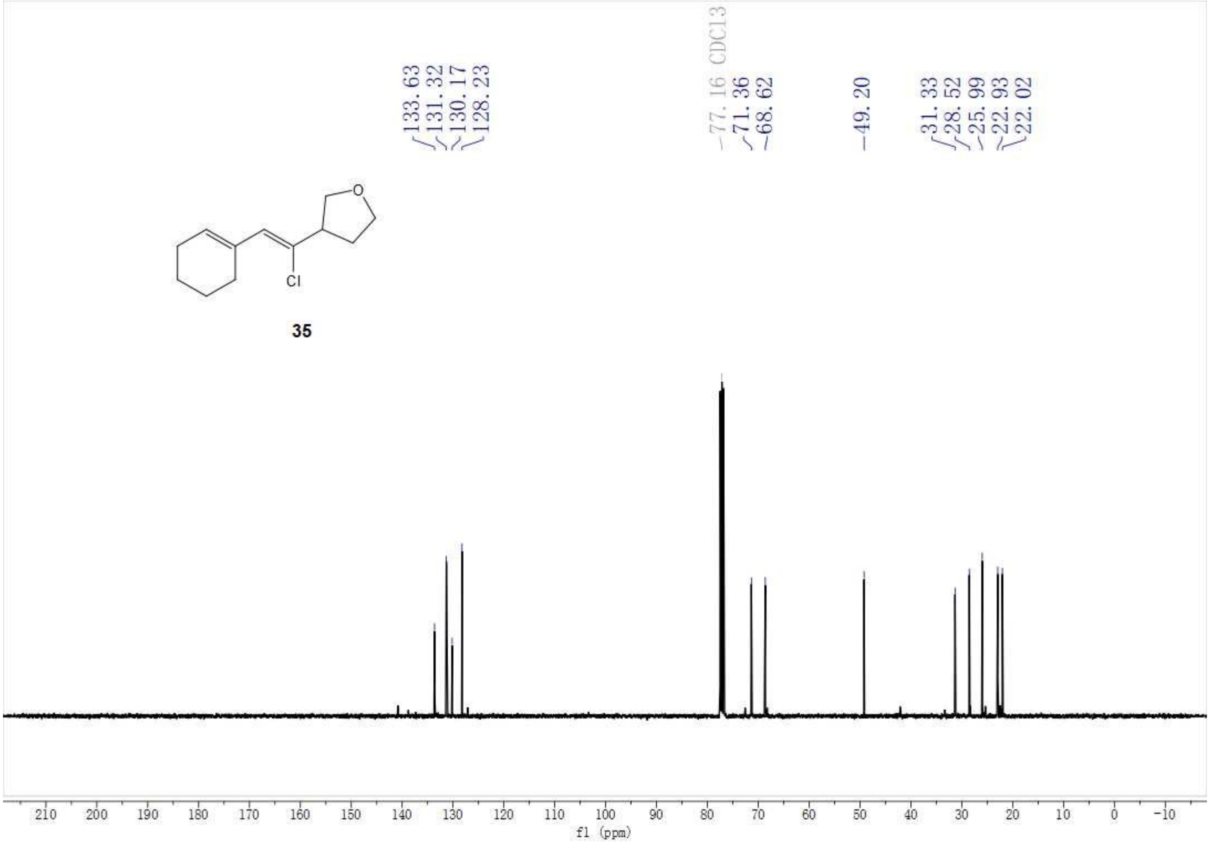

^{13}C-NMR (101 MHz, $CDCl_3$) of **35**, *Z* isomer.

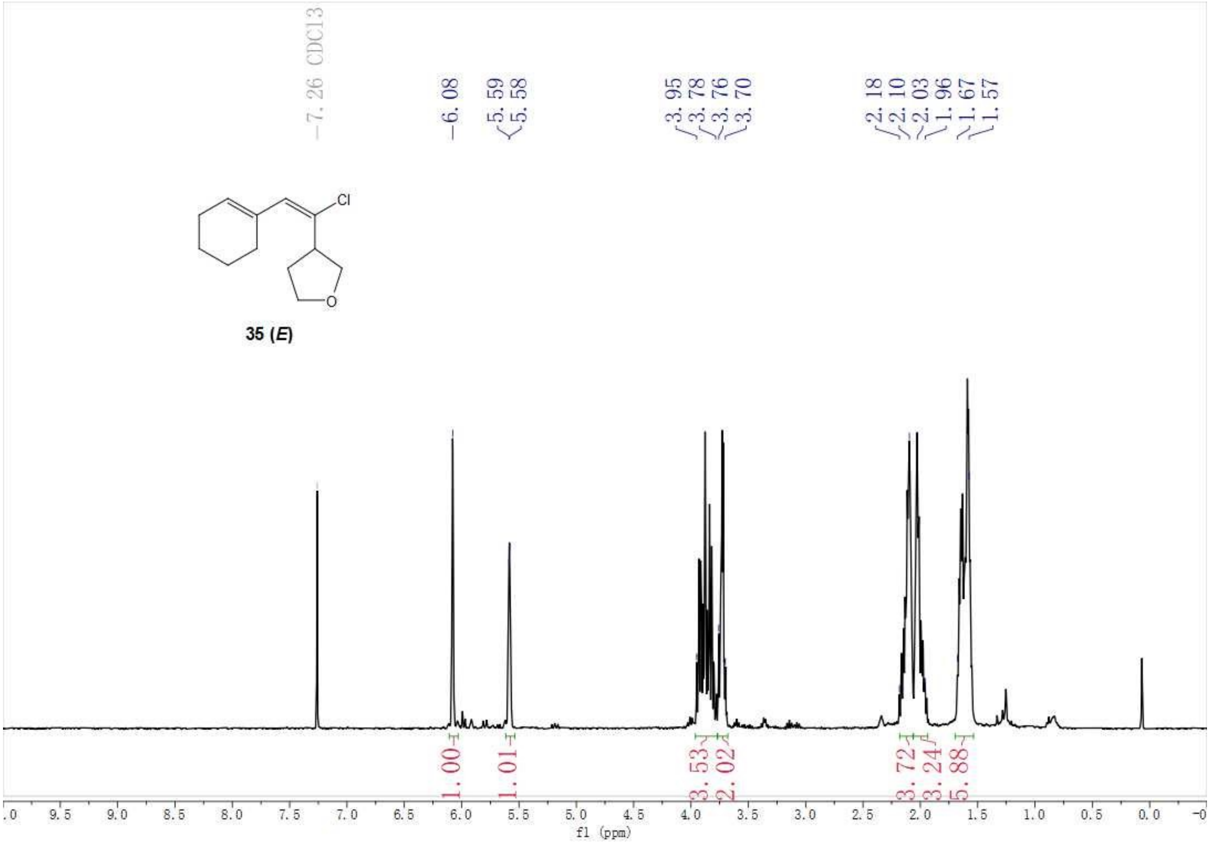

^{1}H-NMR (400 MHz, $CDCl_3$) of **35**, *E* isomer.

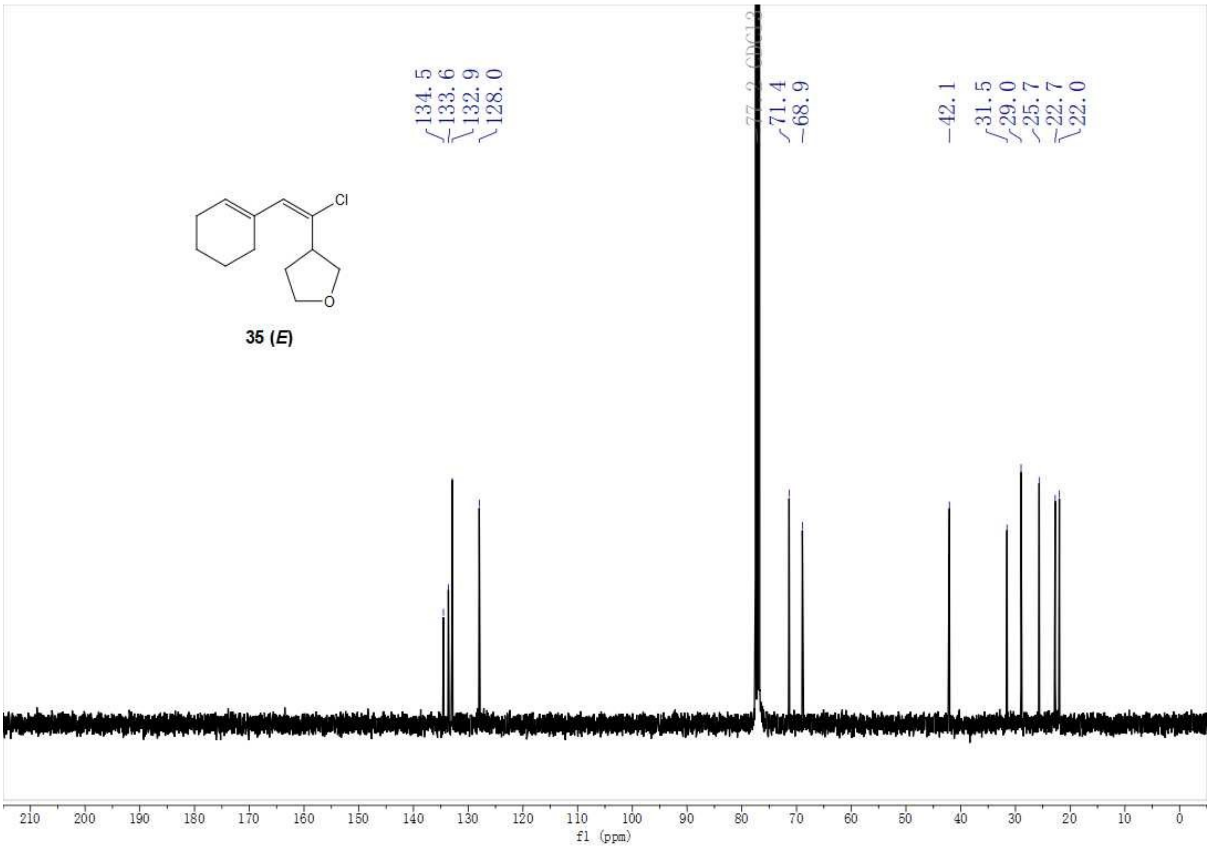

^{13}C-NMR (101 MHz, $CDCl_3$) of **35**, *E* isomer.

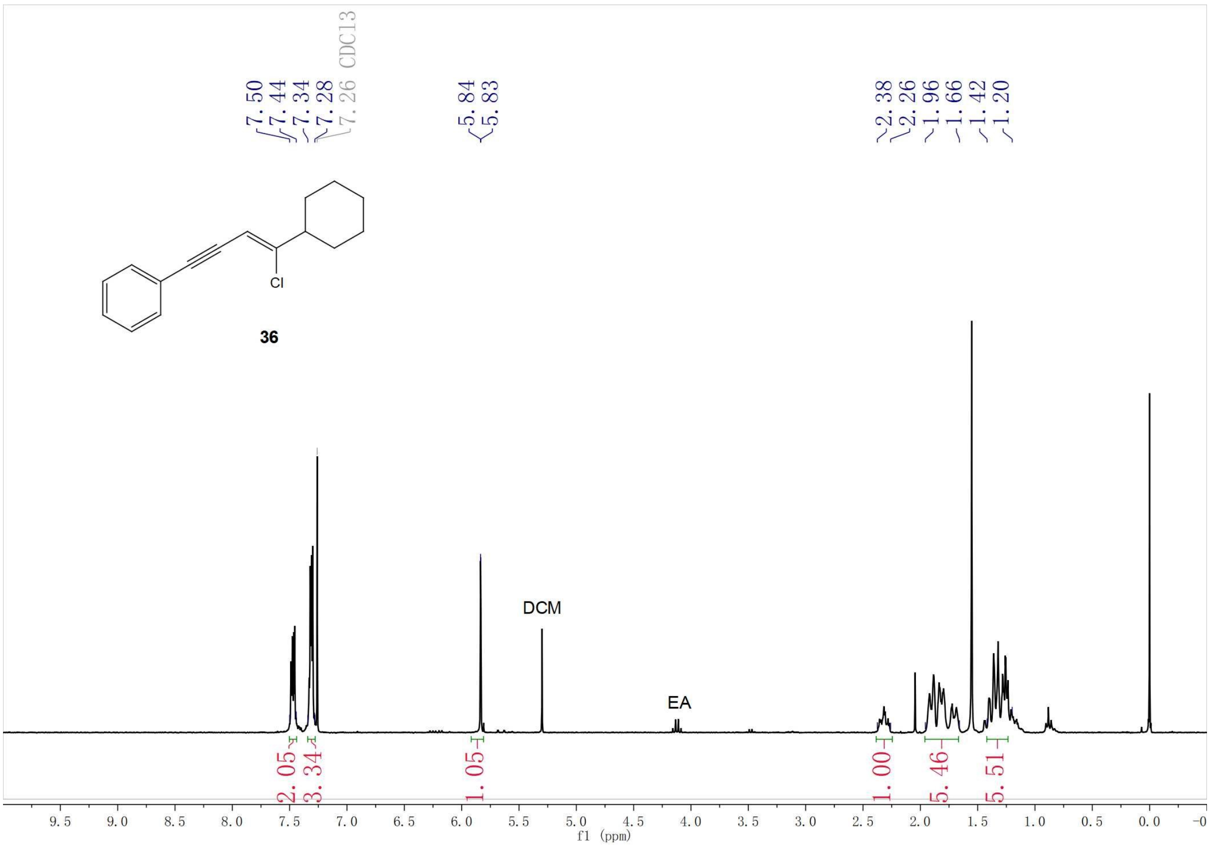

^{1}H-NMR (300 MHz, $CDCl_3$) of **36**, *Z* isomer.

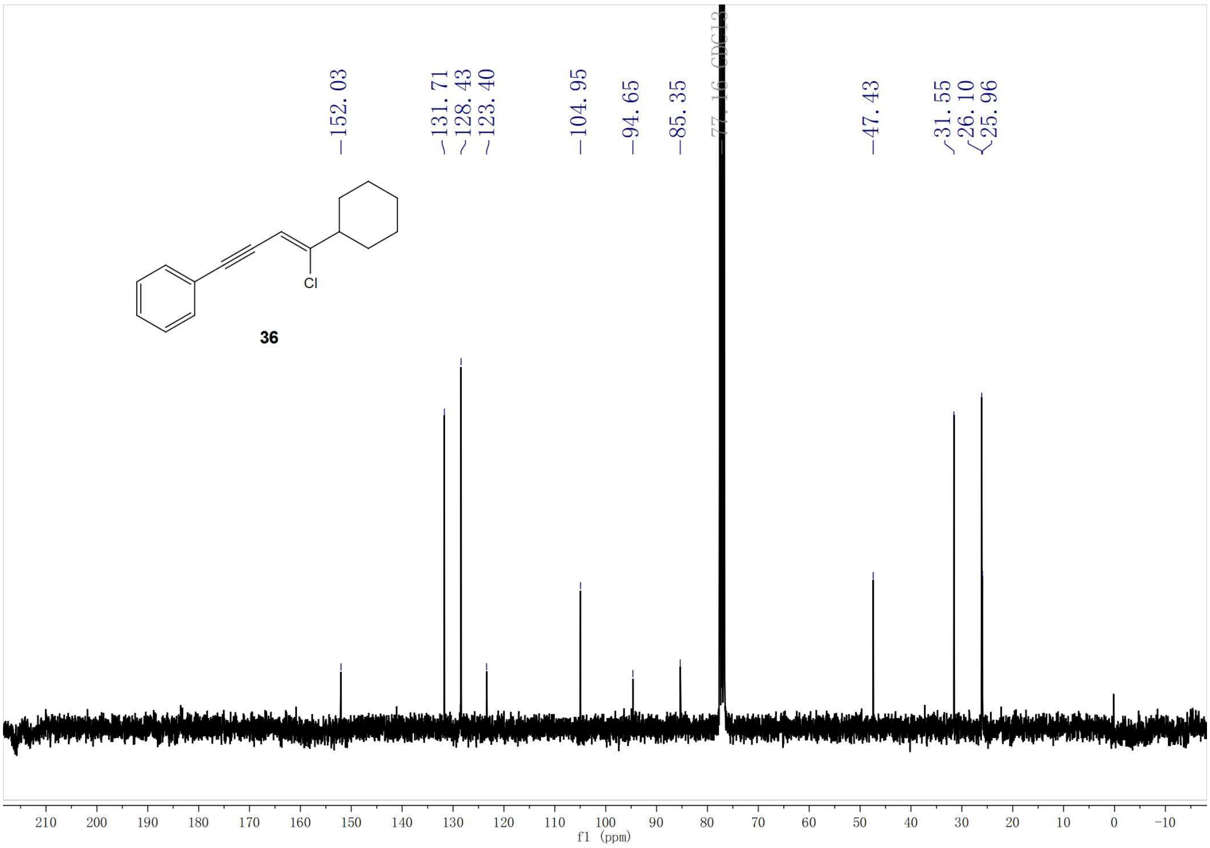

^{13}C-NMR (101 MHz, $CDCl_3$) of **36**, *Z* isomer.

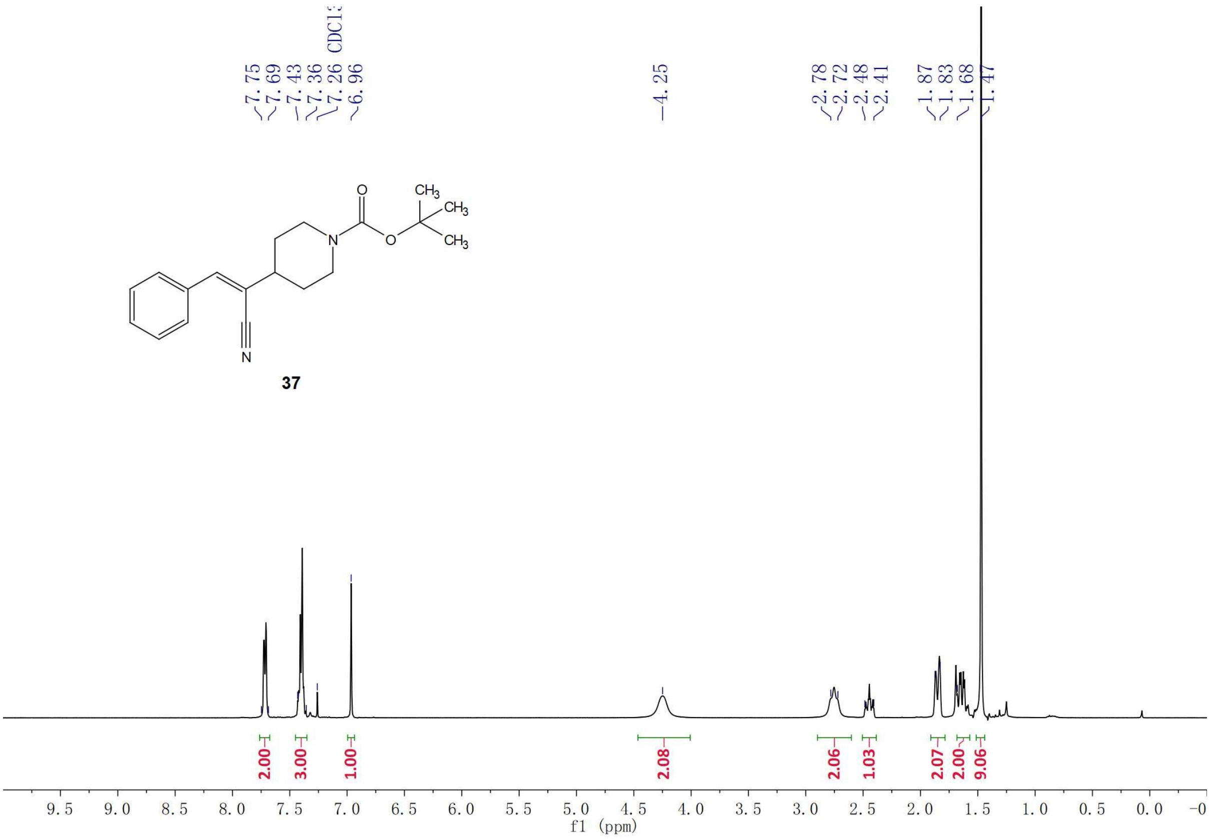

^{1}H-NMR (400 MHz, $CDCl_3$) of **37**.

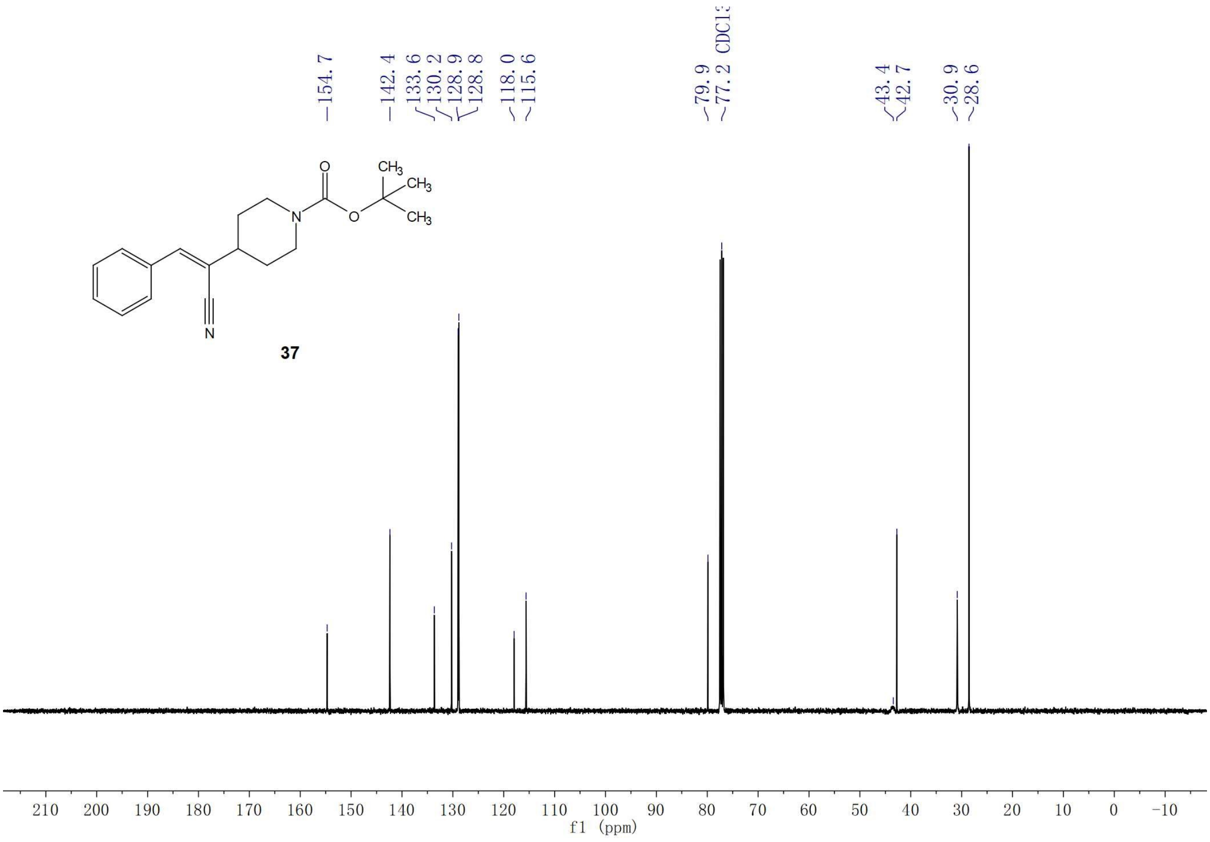

^{13}C-NMR (101 MHz, $CDCl_3$) of **37**.

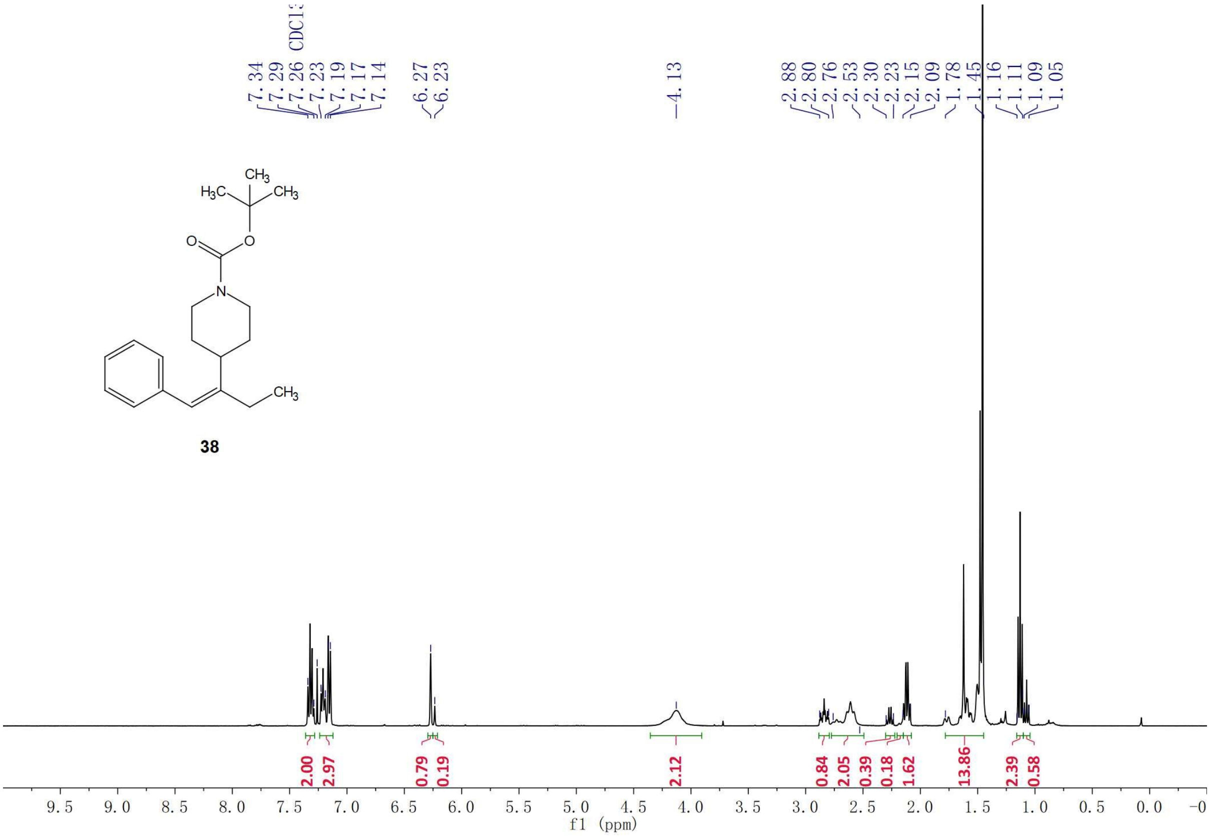

^{1}H-NMR (400 MHz, $CDCl_3$) of **38**.

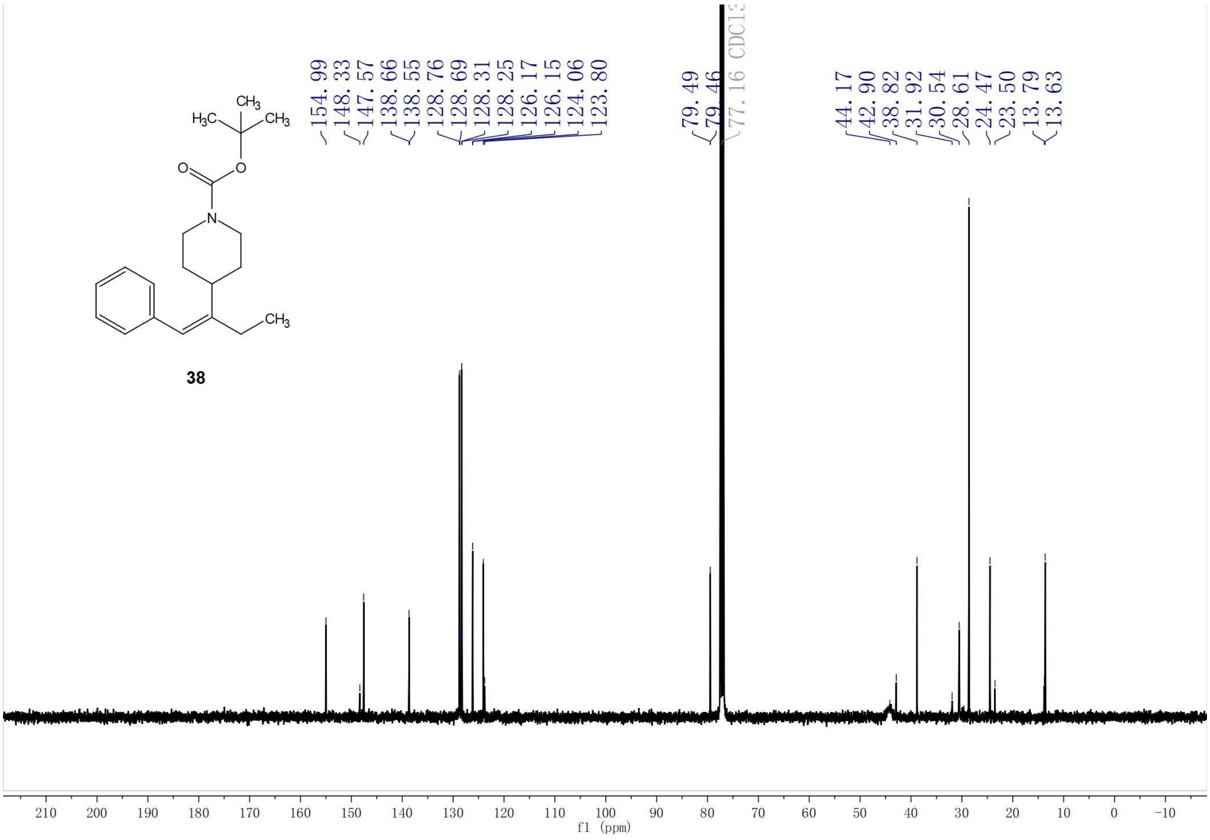

^{13}C-NMR (101 MHz, $CDCl_3$) of **38**.

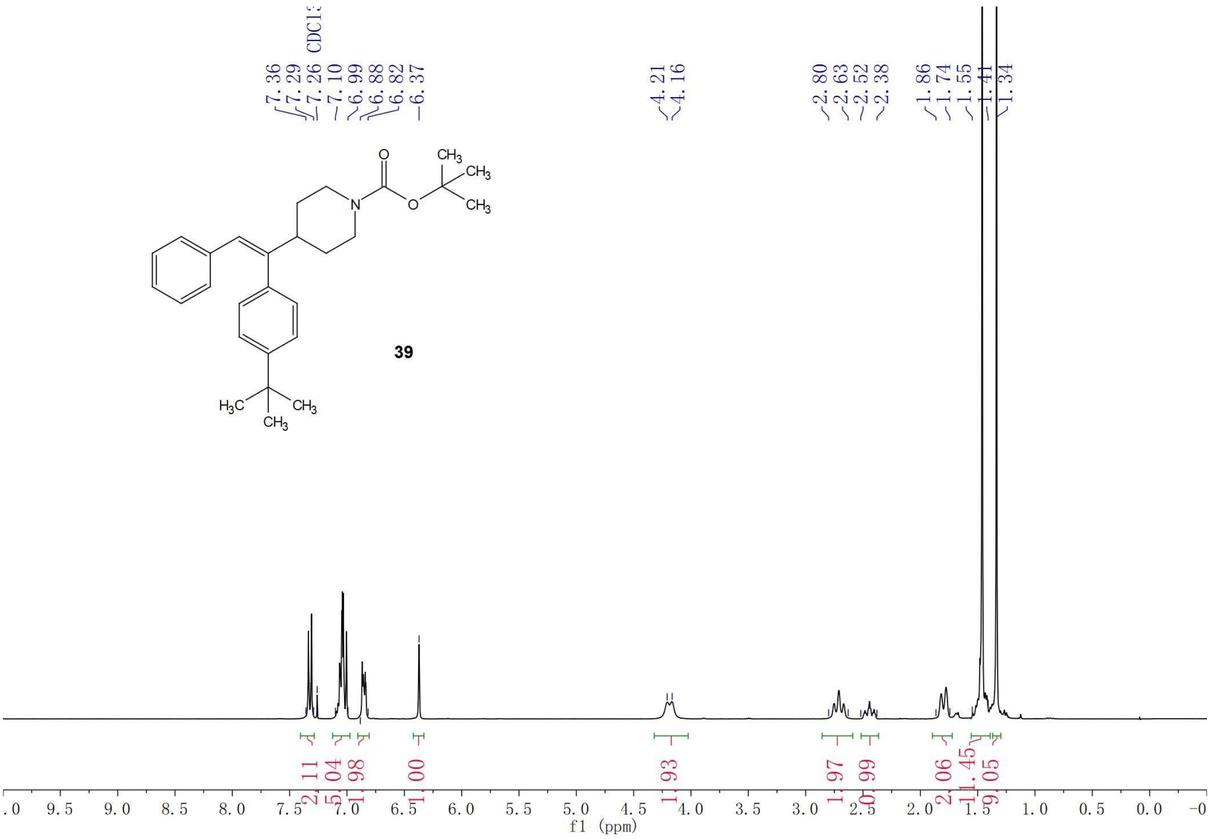

^{1}H-NMR (300 MHz, $CDCl_3$) of **39**.

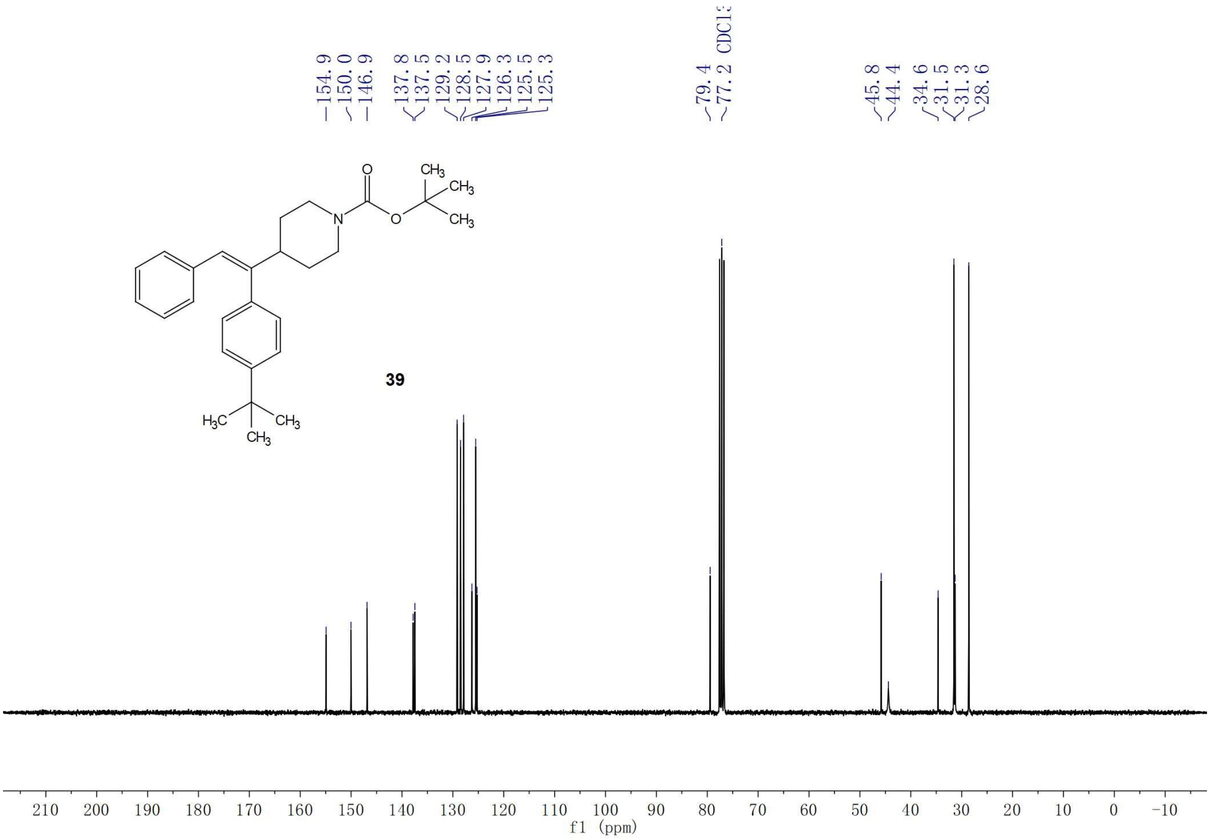

^{13}C-NMR (101 MHz, $CDCl_3$) of **39**.
